# Mechanisms of Surface Antigenic Variation in the Human Pathogenic Fungus *Pneumocystis jirovecii*

**DOI:** 10.1128/mBio.01470-17

**Published:** 2017-11-07

**Authors:** Emanuel Schmid-Siegert, Sophie Richard, Amanda Luraschi, Konrad Mühlethaler, Marco Pagni, Philippe M. Hauser

**Affiliations:** a Vital-IT Group, SIB Swiss Institute of Bioinformatics, Lausanne, Switzerland; b Institute of Microbiology, Lausanne University Hospital, Lausanne, Switzerland; c Institut für Infektionskrankheiten, Universität Bern, Bern, Switzerland; Albert Einstein College of Medicine

**Keywords:** PCP, PacBio sequencing, *Pneumocystis jirovecii*, *Pneumocystis carinii*, adhesin, gene exchange, major surface glycoprotein, mosaicism, subtelomere, telomere exchange

## Abstract

Microbial pathogens commonly escape the human immune system by varying surface proteins. We investigated the mechanisms used for that purpose by *Pneumocystis jirovecii*. This uncultivable fungus is an obligate pulmonary pathogen that in immunocompromised individuals causes pneumonia, a major life-threatening infection. Long-read PacBio sequencing was used to assemble a core of subtelomeres of a single *P. jirovecii* strain from a bronchoalveolar lavage fluid specimen from a single patient. A total of 113 genes encoding surface proteins were identified, including 28 pseudogenes. These genes formed a subtelomeric gene superfamily, which included five families encoding adhesive glycosylphosphatidylinositol (GPI)-anchored glycoproteins and one family encoding excreted glycoproteins. Numerical analyses suggested that diversification of the glycoproteins relies on mosaic genes created by ectopic recombination and occurs only within each family. DNA motifs suggested that all genes are expressed independently, except those of the family encoding the most abundant surface glycoproteins, which are subject to mutually exclusive expression. PCR analyses showed that exchange of the expressed gene of the latter family occurs frequently, possibly favored by the location of the genes proximal to the telomere because this allows concomitant telomere exchange. Our observations suggest that (i) the *P. jirovecii* cell surface is made of a complex mixture of different surface proteins, with a majority of a single isoform of the most abundant glycoprotein, (ii) genetic mosaicism within each family ensures variation of the glycoproteins, and (iii) the strategy of the fungus consists of the continuous production of new subpopulations composed of cells that are antigenically different.

## IMPORTANCE

*Pneumocystis jirovecii* is a fungus causing severe pneumonia in immunocompromised individuals. It is the second most frequent life-threatening invasive fungal infection. We have studied the mechanisms of antigenic variation used by this pathogen to escape the human immune system, a strategy commonly used by pathogenic microorganisms. Using a new DNA sequencing technology generating long reads, we could characterize the highly repetitive gene families encoding the proteins that are present on the cellular surface of this pest. These gene families are localized in the regions close to the ends of all chromosomes, the subtelomeres. Such chromosomal localization was found to favor genetic recombinations between members of each gene family and to allow diversification of these proteins continuously over time. This pathogen seems to use a strategy of antigenic variation consisting of the continuous production of new subpopulations composed of cells that are antigenically different. Such a strategy is unique among human pathogens.

## INTRODUCTION

*Pneumocystis jirovecii* is a fungus colonizing specifically human lungs. It has developed strategies to survive in healthy human lungs, at least transiently, and can turn into a deadly pathogen causing pneumonia in individuals with debilitated immune system (1–4). This disease is the second-most-frequent life-threatening invasive fungal infection, with ca. 400,000 cases per year worldwide (5). However, the biology of this pest remains difficult to study in the lab because of the lack of any established methods for *in vitro* culture. Recent progress in understanding *P. jirovecii* biology strongly benefitted from the publication of two assemblies of its genome from two different clinical samples (4, 6).

In contrast to other pathogenic fungi, the cells of *P. jirovecii* lack chitin as well as glucans during part of the cell cycle, which may avoid eliciting innate and acquired immune responses (4). Moreover, a mechanism of surface antigenic variation, to which ca. 5% of the genome is dedicated, seems crucial to escape from the human immune system during colonization, although the details have not been understood so far. Surface antigenic variation is a common strategy among major microbial human pathogens: for example, *Plasmodium*, *Trypanosoma*, *Candida*, *Neisseria*, and *Borrelia*. It relies on various genetic and/or epigenetic mechanisms aimed at expressing only one or few of them at once (7). Such systems often involve gene families encoding surface antigens localized at subtelomeres, presumably because these regions of the genome are prone to gene silencing, which is used for mutually exclusive expression, and possibly enhanced mutagenesis (8). Moreover, the formation of clusters of telomeres at the nuclear periphery may favor ectopic recombinations (8), which can be responsible for the generation of new mosaic antigens.

Surface antigenic variation has been previously studied on a limited set of genes in *Pneumocystis carinii* infecting specifically rats. The molecular mechanism was then assumed to be also active in *P. jirovecii*, as suggested by studies using PCR-based technologies. Antigen diversity was believed to be generated by recombination between members of a single family of ca. 80 subtelomeric genes encoding isoforms of the major surface glycoprotein (*msg*) (9–11). A single copy of these isoforms would be expressed in each cell thanks to its localization downstream of a subtelomeric expression site, the upstream conserved element (UCS) present at a single copy in the genome. The UCS includes the promoter of transcription, the protein start, and the leader sequence responsible for translocation of the protein into the endoplasmic reticulum for final incorporation into the cell wall (12, 13). The mechanism for exchange of the expressed *msg* gene is thought to be by recombination at a 33-bp-long sequence that is present at both the end of the UCS and the beginning of each *msg* (the conserved recombination junction element [CRJE]). The exchange of the expressed gene seems relatively frequent and would explain how different *msg* genes can be expressed in each population (12). The CRJE sequence encodes at its end a potential lysine-arginine recognition site for kexin endoprotease, which might be involved in the maturation of the antigen. Kutty et al. (14) provided evidence for frequent recombinations among *msg* genes creating potentially mosaic genes. All of these observations were made using conventional cloning procedures and PCRs, and these mechanisms have yet to be understood in a more extensive genomic context.

The first genome sequence of *P. jirovecii* released was obtained using technologies generating short reads, which prevented assembly of long repetitive sequences such as centromeres, telomeres, and subtelomeres, including *msg* genes (6). A second study used a mixture of techniques that generated nearly complete chromosomes of *P. jirovecii*, *P. carinii*, and *Pneumocystis murina* (infecting specifically mice) (4). These latter authors used PCRs to reconstruct the subtelomeres, which allowed the discovery of new subtelomeric gene families related to *msg*. However, they did not investigate the function of these proteins, the mechanisms involved in their expression and gene variation, or the global strategy of antigenic variation of these fungi.

The aim of the present study was to analyze in detail the mechanisms of surface antigenic variation in *P. jirovecii*. To that purpose, we used the PacBio sequencing technology generating long DNA reads to assemble a set of subtelomeres of a single *P. jirovecii* strain from a bronchoalveolar lavage fluid (BALF) specimen from a single patient. The analysis of this data set and laboratory experiments permit a new classification and the characterization of six subtelomeric *msg* families, demonstrate the presence of pseudogenes, and provide important new insights into the molecular mechanisms responsible for antigenic variation. Moreover, our observations suggest a unique strategy of antigenic variation consisting of the continuous production of new subpopulations composed of cells that are antigenically different. This strategy may be associated with the particular nonsterile niche within lungs.

## RESULTS

Most if not all *P. jirovecii* infections are polyclonal (15). In order to facilitate the study of the mechanisms of antigenic variation, one patient infected with a vastly dominant strain was selected by multitarget genotyping. The genome of a single *P. jirovecii* strain was assembled into 219 contigs using PacBio sequencing and a dedicated bioinformatics strategy for read processing.

### Identification of subtelomeric *msg* genes and pseudogenes.

Automated gene prediction performed poorly in the subtelomeric regions compared to the core of the genome, due to abundant stretches of low-complexity DNA, numerous pseudogenes, residual assembly errors in homopolymers, and the lack of a start codon in many *msg* genes. The *msg* genes were detected by sequence homology using generalized profiles (16) derived from previously published sequences. A total of 113 *msg* genes with sizes ranging from 331 to 3,337 bp were found on 37 different contigs, only two genes being perfectly identical (*msg52* and *msg61* [see Table S1 in the supplemental material]). Most of them (*n =* 85) contained a single large exon and zero to two small exons at their 5′ end. The remaining 28 genes harbored stop codons in all frames and were considered pseudogenes (see note 1 in Text S1 in the supplemental material).

10.1128/mBio.01470-17.9 TABLE S1  Features of the 113 *msg* genes identified in the *P. jirovecii* isolate. Download TABLE S1, XLSX file, 0.1 MB. Copyright © 2017 Schmid-Siegert et al. 2017 Schmid-Siegert et al. This content is distributed under the terms of the Creative Commons Attribution 4.0 International license.

10.1128/mBio.01470-17.1 TEXT S1  Seven supplementary notes. Download TEXT S1, DOCX file, 0.1 MB. Copyright © 2017 Schmid-Siegert et al. 2017 Schmid-Siegert et al. This content is distributed under the terms of the Creative Commons Attribution 4.0 International license.

### Characterization of the *msg* gene families.

We are proposing a classification of the *msg* genes into six families (Table 1) based on the integration of four independent lines of evidence: sequence homology, gene structure, protein property, and recombination events. The global picture that emerged is coherent, and the details on the different points are presented below.

**TABLE 1:**
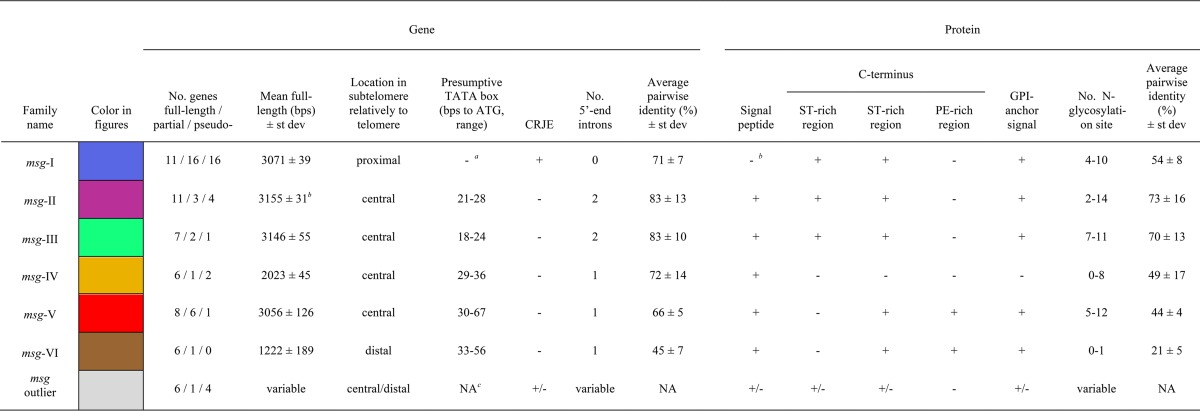
Characteristics of the *msg* families identified in *P. jirovecii*

a The promoter including the signal peptide for family I is within the UCS present at a single copy per genome.

b The *msg3* gene was not used to calculate this value because it is ca. 900 bp shorter than the other genes of the family, although it presents all features of the family (see alignment in Fig. S2).

c NA, not applicable.

**FIG 1:**
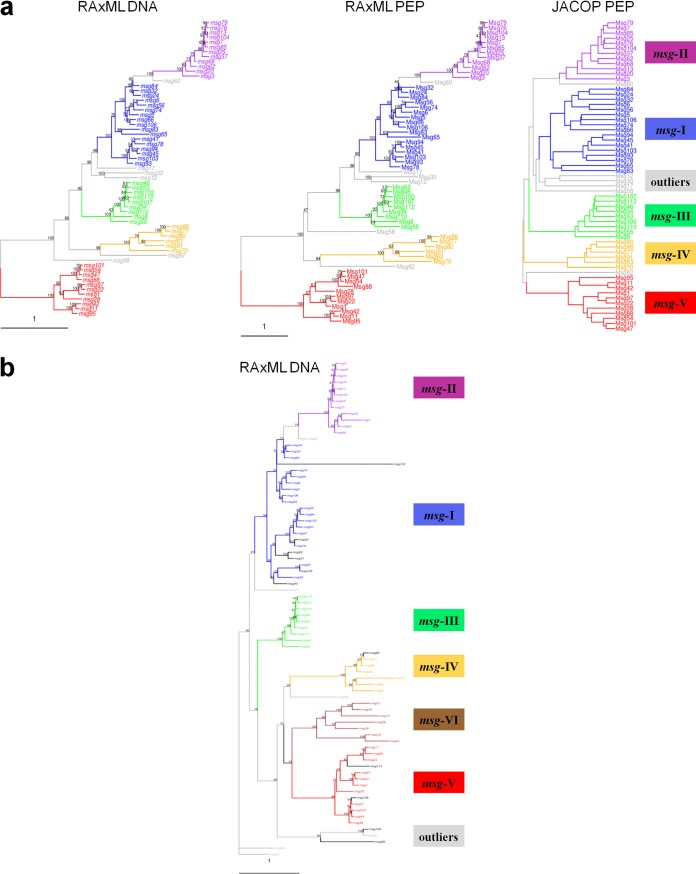
Classification trees of *P. jirovecii msg* genes and Msg proteins. The different families are represented by color, and their characteristics are summarized in Table 1. A few unclassified outliers are in gray. The scale represents the number of mean substitutions per site. (a) RAxML DNA and PEP are maximum likelihood trees of nucleotide and amino acid sequences of the 61 genes with an exon larger than 1.6 kb. Members of family V were defined as the out-group (1,000 bootstraps). JACOP PEP is a hierarchical classification based on local sequence similarity, a method that does not rely on a particular multiple sequence alignment. (b) Maximum likelihood tree of the 61 genes with an exon larger than 1.6 kb plus 18 genes with an exon smaller than 1.6 kb. The sequences were trimmed from position 1540 of the first alignment up to their end and realigned to construct the tree (1,000 bootstraps). Seven of the 18 genes with an exon smaller than 1.6 kb constitute the *msg* family VI shown in brown, whereas the remaining 11 shown in black belong to the other *msg* families.

Figure 1a shows the results of the analysis of 61 *msg* genes containing an exon of ≥1.6 kb. Based on the multiple sequence alignments (MSAs) of the coding sequences (CDSs) and their predicted proteins, two phylogenetic trees were computed using RAxML. The different gene families are clearly individualized as clades, with the exceptions of (i) *msg-II*, which appears as a subclade of *msg-I*, and (ii) *msg-I*, which seems to include two subclades. Using an alternative classification method that does not rely on a single particular MSA (JACOP [Fig. 1a]), the placement of *msg-II* as a subclade of *msg-I* was not confirmed, whereas the subclades of *msg-I* were. Due to the differences in the gene structures and the recombination events reported below, we believe that (i) *msg-I* and *msg-II* should be treated separately, and (ii) *msg-I* should be considered a single family, including two subclades. Figure 1b shows the analysis of trimmed CDS sequences allowing the placement of the *msg-VI* family, which appeared as a clade on its own, while the classification of the other families remained essentially unchanged. Figure S1 in the supplemental material shows that most pseudogenes could be attributed to one of the six *msg* families, and their often longer branches further account for their pseudogenic nature.

10.1128/mBio.01470-17.2 FIG S1  Maximum likelihood phylogenetic tree, including pseudogenes. Shown is analysis of 61 *P. jirovecii msg* genes with an exon larger than 1.6 kb plus 26 pseudogenes larger than 1.6 kb, shown in black. The 26 sequences have been added to the tree of Fig. 1a using the evolutionary placement algorithm (EPA) from RAxML (no new bootstrap values). The scale represents the mean number of substitutions per site. Download FIG S1, TIF file, 1 MB. Copyright © 2017 Schmid-Siegert et al. 2017 Schmid-Siegert et al. This content is distributed under the terms of the Creative Commons Attribution 4.0 International license.

**FIG 2:**
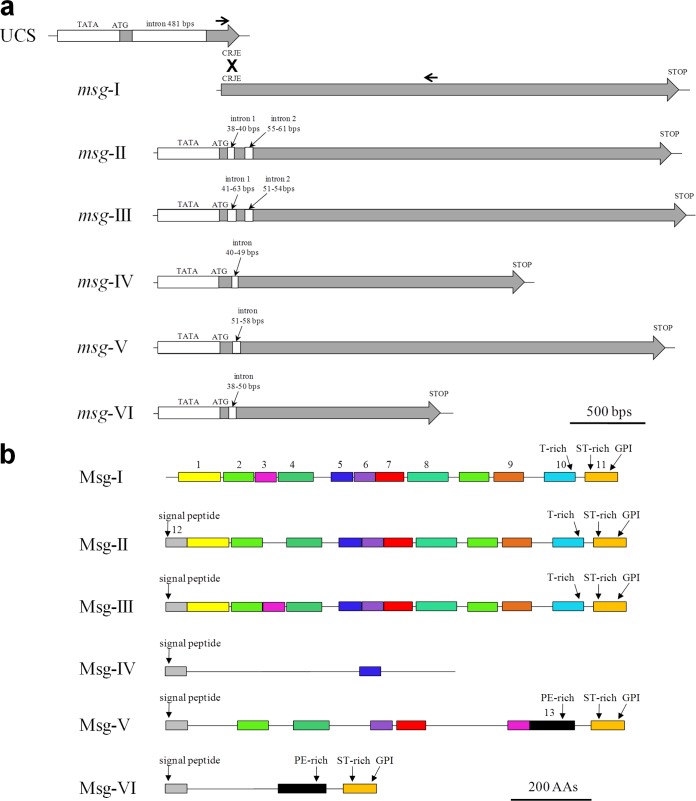
Diagrams of the structure of *P. jirovecii msg* genes and Msg proteins belonging to families I to VI. (a) Features of the *msg* genes of each family derived from the analysis of the full-length genes. The UCS and recombination between CRJE sequences are shown for family I. The approximate positions of PCR primers used for identification of the *msg-I* expressed genes linked to the UCS are shown by arrows (see note 4 in Text S1). (b) Features of Msg proteins of each family derived from the analyses of the full-length proteins. The 13 domains identified by MEME analysis are shown. The logos of these domains are shown in Fig. S4.

Manual curation of the *msg* genes led to their classification as full-length, partial, and pseudogenes (Table S1). Table 1 shows the characteristics of each family identified by the analysis of the sequences of the full-length genes, as well as their alignments (see Fig. S2 in the supplemental material). Except those of the family *msg-I*, each *msg* gene presented one or two introns at its 5′ end, as well as a presumptive TATA box upstream of the ATG and an initiator motif (Cap signal) at presumptive sites of initiation transcription (Fig. 2a; see Fig. S2 in the supplemental material). The members of family I had only the conserved recombination junction element (CRJE) at the beginning of their single exon. These observations suggested that members of family I can be expressed only upon recombination of their CRJE with that of the single-copy UCS, which encompasses a promoter, whereas all members of the other five families are expressed independently. Three of the six full-length outlier genes seemed not to be expressed since they had no CRJE and missed a TATA box (Table S1). Twenty-six partial genes were truncated by the end of the contig so that only three bona fide partial genes were identified, which, however, missed the TATA box, signal peptide, and/or GPI anchor signal, and thus were probably not expressed or not correctly processed (*msg44*, *msg89*, and *msg99*).

10.1128/mBio.01470-17.3 FIG S2  Alignment of the full-length *msg* genes of the six families. The 300 bp upstream of the genes were also aligned to detect eventual upstream potential TATA boxes. Download FIG S2, DOCX file, 0.2 MB. Copyright © 2017 Schmid-Siegert et al. 2017 Schmid-Siegert et al. This content is distributed under the terms of the Creative Commons Attribution 4.0 International license.

### Characterization of the Msg protein families.

Analysis of the sequences and alignments (see Fig. S3 in the supplemental material) of the full-length proteins of each family revealed that each Msg protein, except those of family I, presented a signal peptide at its N terminus (Fig. 2b). Proteins of family I probably acquire a signal peptide upon fusion of their encoding gene with the UCS. Except those of family IV, each Msg protein presented a GPI anchor signal at its C terminus. These observations suggested that all Msg proteins are attached externally to the cell wall, except those of family IV, which would be secreted in the environment or attached to the cell wall through another mechanism than GPI.

10.1128/mBio.01470-17.4 FIG S3  Alignment of the full-length Msg proteins of the six families. Download FIG S3, DOCX file, 0.1 MB. Copyright © 2017 Schmid-Siegert et al. 2017 Schmid-Siegert et al. This content is distributed under the terms of the Creative Commons Attribution 4.0 International license.

The possible conservation of motifs among the proteins of the six families was investigated using MEME (multiple expectation-maximization for motif elicitation) analysis (17). Thirteen conserved motifs were identified, the arrangement of which was fairly diagnostic within each family (Fig. 2b). Most motifs included several conserved cysteines and leucines, which resembled the previously identified Pfam MSG domain (see Fig. S4 in the supplemental material). Interestingly, conserved leucines were often separated by two to six residues. The beginning of motif 10 corresponded to the end of the previously identified Pfam Msg2_C domain. Accordingly, Pfam predictions identified one to five MSG domains (often partial) per protein of all families and a single Msg2_C domain in each Msg-I protein (see Fig. S5 and Table S2 in the supplemental material). The Msg2_C domain was not predicted in families II and III, although they harbored the corresponding motif 10, suggesting that this domain is divergent in these families. The Ncoils predictor revealed three to five coiled-coil motifs spread along members of families I, II, and III, whereas unstructured regions were predicted at the C terminus of Msg proteins of families I, III, V, and VI (Fig. S5).

10.1128/mBio.01470-17.5 FIG S4  Logos of the MEME domains identified among Msg proteins. The distribution of the 13 domains within the Msg proteins of the six families is shown in Fig. 2b. The two Msg domains present in the Pfam database are shown at the bottom. Download FIG S4, DOCX file, 1.4 MB. Copyright © 2017 Schmid-Siegert et al. 2017 Schmid-Siegert et al. This content is distributed under the terms of the Creative Commons Attribution 4.0 International license.

10.1128/mBio.01470-17.6 FIG S5  Representative examples of predicted domains in *P. jirovecii* Msg proteins. HMMER with embedded predictors was used. The Pfam predictions of all Msg proteins are given in Table S2. Download FIG S5, TIF file, 0.7 MB. Copyright © 2017 Schmid-Siegert et al. 2017 Schmid-Siegert et al. This content is distributed under the terms of the Creative Commons Attribution 4.0 International license.

10.1128/mBio.01470-17.10 TABLE S2  Pfam predictions of full-length *P. jirovecii* Msg proteins using HMMER (57) (biosequence analysis using profile hidden Markov models). Download TABLE S2, XLSX file, 0.1 MB. Copyright © 2017 Schmid-Siegert et al. 2017 Schmid-Siegert et al. This content is distributed under the terms of the Creative Commons Attribution 4.0 International license.

Except those of family IV, each Msg protein harbored at its C terminus two MEME motifs, which included a region enriched in specific residues: threonine (T rich [motif 10]), serine and threonine (ST rich [motif 11]), or proline and glutamine (PE rich [motif 13]) (Fig. 2b; Table 1). The T-rich region in family I included generally a stretch of 9 to 15 Ts, which was not present in families II and III (Fig. S3). The PE-rich region in family V was enriched in proline residues relatively to that present in family VI (Fig. S3). Four to 14 potential sites of nitrogen-linked glycosylation of asparagines were predicted to be present in each Msg protein, except in family VI, which presented no or only one such site (Table 1; Fig. S3). The localization of these glycosylation sites was widespread along the protein and fairly conserved within each family (Fig. S3).

### Arrangement of the *msg* families within the subtelomeres.

**FIG 3:**
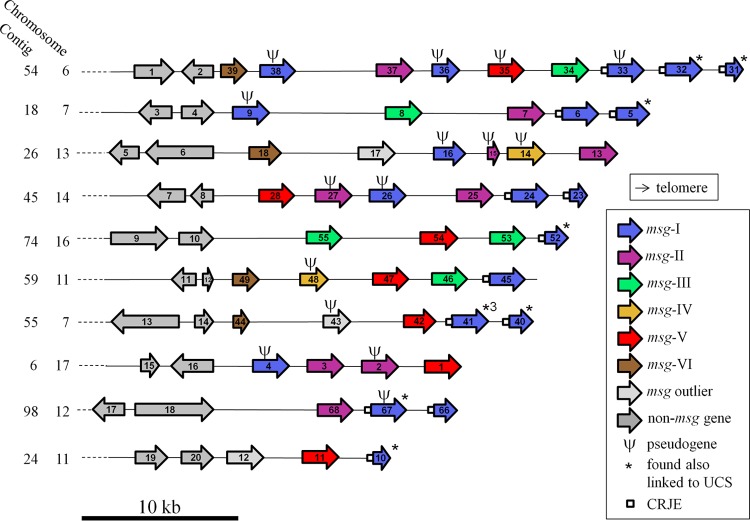
Diagrams of 10 representative *P. jirovecii* assembled subtelomeres. The other 27 assembled subtelomeres are shown in Fig. S6. The *msg* genes are described in detail in Table S1. The attribution of the contigs to the chromosomes previously described using flanking non-*msg* genes is described in Table S3 at http://www.chuv.ch/microbiologie/en/imu_home/imu-recherche/imu-research-groups/imu-research-phauser/imu-supplementary_data.htm. The number of subclones obtained from the generic PCR amplifying *msg-I* genes linked to the UCS is indicated close to the asterisk of one *msg-I* gene of contig 55 (see note 4 in Text S1). The functions/products of 4 of the 20 non-*msg* genes are known (see Table S3): gene 6, thiamine pyrophosphokinase; gene 9, amidophosphoribosyltransferase; gene 15, 60S ribosomal protein L28; and gene 16, potassium-sodium efflux P-type ATPase.

Consistent with a subtelomeric localization, the *msg* genes were grouped at one end of their contig when flanking non-*msg* genes were also present (in 20 of 37 contigs [Fig. 3; see Fig. S6a in the supplemental material]). All *msg* genes identified were oriented toward one end of the contig (i.e., presumably toward the telomere). (No telomeric repeats were identified for an unknown reason [see note 2 in Text S1].) Except for pseudogenes, which were dispersed all over the subtelomeres, all members of family I with a CRJE were the closest to the end of their contig (i.e., proximal to the telomere) (Fig. 3; Fig. S6). In contrast, all members of family VI were the closest to the flanking non-*msg* genes present on their contig (i.e., distal to the telomere). Members of the four remaining families were localized centrally in the subtelomeres, between those of families I and VI. There were up to three *msg-I* genes grouped at the end of 19 contigs. Members of the other five families did not show any clear grouping patterns.

10.1128/mBio.01470-17.7 FIG S6  Diagrams of the 27 supplementary *P. jirovecii* assembled subtelomeres. with (a) or without (b) flanking non-*msg* genes. The attribution of the subtelomeres to the 20 chromosomes described by Ma et al. (4) using the flanking non-*msg* genes is described in Table S3. Contig 72 to which the UCS has been linked using PCR amplifications is shown at the bottom of panel a. In panel b, the number of subclones obtained from the generic PCR amplifying *msg-I* genes linked to the UCS is indicated close to the asterisk of one *msg-I* gene of contigs 95, 110, and 148 (see note 4 in Text S1). The functions/products of 6 of the 22 non-*msg* genes are known (see Table S3 at http://www.chuv.ch/microbiologie/en/imu_home/imu-recherche/imu-research-groups/imu-research-phauser/imu-supplementary_data.htm): gene 32, mevalonate kinase; gene 34, rRNA adenine *N*(6)-methyltransferase; gene 37, metalloendopeptidase; gene 38, ferrochelatase; gene 39, nuclear distribution protein PAC1; gene 41, transketolase. Download FIG S6, PDF file, 0.4 MB. Copyright © 2017 Schmid-Siegert et al. 2017 Schmid-Siegert et al. This content is distributed under the terms of the Creative Commons Attribution 4.0 International license.

### Identification of the expression site of *msg-I* genes and of the genes linked to it.

Each infection by *P. jirovecii* is believed to involve a mixture of cells expressing different *msg-I* genes under the control of the expression site (i.e., the UCS, which is present at a single copy per genome) (12). Consequently, the UCS was expected to be linked to different *msg-I* genes in our DNA sample and thus cannot be unequivocally assembled, which plausibly explains its absence from the PacBio assembly. A single UCS was retrieved from our DNA sample using PCRs based on published sequences, and it could be linked to one of the PacBio contigs (see note 3 [4, 61] in Text S1). The UCS retrieved from our sample was identical to that of Ma et al. (4), except for a few small changes not modifying the encoded protein (see Fig. S7 in the supplemental material). Interestingly, the CRJE sequence at the end of the UCS and the beginning of each *msg-I* gene presented an imperfect inverted repeat that had never been pointed out so far (Fig. S7).

10.1128/mBio.01470-17.8 FIG S7  Nucleotide sequence of the UCS (upstream conserved element). The encoded protein is shown, as well as relevant features. The polymorphisms relatively to sequence previously published by Ma et al. (4) are indicated. Relevant motifs described by Kutty et al. (12) are shown. Download FIG S7, DOCX file, 0.1 MB. Copyright © 2017 Schmid-Siegert et al. 2017 Schmid-Siegert et al. This content is distributed under the terms of the Creative Commons Attribution 4.0 International license.

In order to identify the *msg-I* genes linked to the UCS in our sample, we amplified by PCR the junction between these elements using one primer within the UCS and either one primer generic for many *msg-I* genes (12) or one primer specific to a given *msg-I* gene of the PacBio assembly (Fig. 2a; see note 4 in Text S1). Eighteen different *msg-I* genes were found fused in frame to the UCS at the CRJE sequence, two being pseudogenes of the family I with an upstream CRJE sequence and four being new *msg-I* sequences not present in the PacBio assembly. The 12 *msg-I* genes found linked to the UCS that were present in the PacBio assembly are identified in Fig. 3 and Fig. S6 by asterisks. Three specific *msg-I* genes linked to the UCS represented 74% of the subclones of the generic PCR analyzed, suggesting that subpopulations of cells expressing given *msg-I* genes were of different sizes in our sample (see note 4 in Text S1). These observations suggested that recombination between the CRJE sequence of the UCS and that of different *msg-I* genes occurred at a high frequency in the single *P. jirovecii* population studied here.

### Set of assembled subtelomeres.

The flanking non-*msg* genes allowed attribution of 20 of our 37 contigs (Fig. 3 and Fig. S6a) to 15 of the 20 full-length chromosomes described by Ma et al. (4) because they were also present in the latter assembly (see Table S3 at http://www.chuv.ch/microbiologie/en/imu_home/imu-recherche/imu-research-groups/imu-research-phauser/imu-supplementary_data.htm). All of the remaining 17 contigs without flanking non-*msg* genes (Fig. S6b) could have been assembled from the same subtelomeres as the other contigs. Thus, we assembled at least 20 subtelomeres out of the 40 potentially present in each cell. Given the presence of a large number of subpopulations expressing different *msg-I* genes in our sample, the set of subtelomeres present in each cell varied considerably. It is likely that the set we assembled corresponded to a core of subtelomeres that was present in a majority of cells of the population so that it could be assembled unequivocally.

### Recombination between *msg* genes.

**FIG 4:**
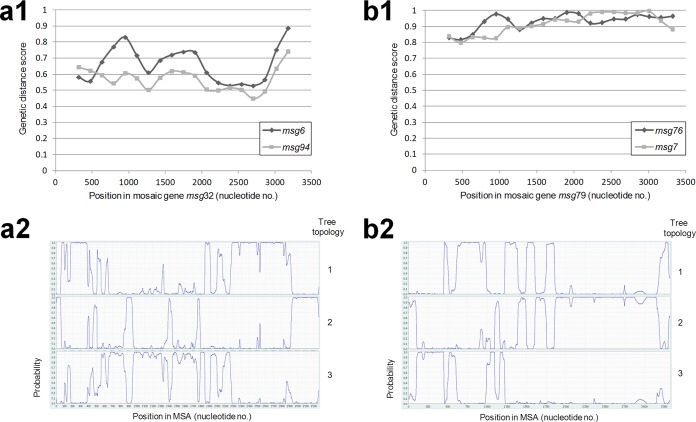
Examples of detection of potential mosaic genes. (a) Mosaic gene *msg32*. (a1) The set of 11 full-length *msg-I* genes was analyzed using the Recombination Analysis Tool. This method measures genetic distances in windows sliding along the MSA. The genetic distance scores of the putative parent genes at the middle of each window are plotted against the position in the mosaic gene. The predicted recombination site is at position ca. 600, at the crossover of the curves. The second screening method, Bellerophon, which is based on a similar analysis, identified a recombination event at position 392. (a2) Analysis of the mosaic gene *msg32* with its putative parent genes together with the randomly chosen gene *msg84* of the same family using the more sensitive method TOPALi, based on the hidden Markov model. This method analyzes only four sequences at a time and calculates the probabilities of the three possible tree topologies at each residue of the MSA. A recombination event is also detected at positions ca. 400 to 600, but many other recombination events are predicted. (b) Mosaic gene *msg79*. This gene shares an almost identical fragment of 947 bp with its putative parent, *msg7* (see alignment in Fig. S8c). (b1) The set of 11 full-length *msg-II* genes was analyzed using the Recombination Analysis Tool. The predicted recombination sites are at positions ca. 400, 1300, 2100, and 3100. The Bellerophon method did not identify this mosaic gene. (b2) Analysis of the mosaic gene *msg79* with its putative parent genes together with the randomly chosen gene *msg85* of the same family using TOPALi based on the hidden Markov model. Recombination events are also detected at positions ca. 400, 1500, and 3100, but not at 2100, and other recombination events are predicted.

Evidence of recombination events between *msg-I* genes was previously provided (14). We investigated this issue among the different *msg* families using three different numerical methods: two allowing analyses of large sets of genes for screening, and one analyzing only four genes at a time for more sensitive analysis. Two to 18 potential mosaic genes and their putative parent genes were detected within each of families I to IV, involving sometimes partial genes or pseudogenes (Fig. 4 and Table 2). On the other hand, only one potential mosaic gene was identified in family V and none in family VI (*P* = 0.06). Eight of the 30 mosaic genes detected shared with one parent a perfectly or almost perfectly identical fragment of ca. 100 to 1,000 bp, often close to the site of the predicted recombination events (Fig. 4b; see Fig. S8 at http://www.chuv.ch/microbiologie/en/imu_home/imu-recherche/imu-research-groups/imu-research-phauser/imu-supplementary_data.htm). These latter cases suggested very recent recombination events. BLAST comparison revealed that the regions between the *msg* genes were more homologous to intergenic regions on other contigs close to *msg* genes of the same family than those of the other families. These homologous regions sometimes shared identical sequences of 100 to 300 bp. This suggested that recombinations between *msg* genes may sometimes also involve the flanking intergenic regions. The putative parent genes of mosaic genes were randomly distributed among the two subclades of family I, suggesting that this family must be considered a single entity (see note 5 in Text S1).

**TABLE 2:** Potential mosaic genes detected within each *msg* family^a^

*msg* family	No. of *msg* genes					No. of potential *msg* mosaic genes				% mosaic
	Full length	Partial	Pseudogenes	Total	Nonmosaic^b^	Full length^c^	Partial	Pseudogenes^d^	Total^b^	
I	11	16	16	43	25	8	1	9	18	42
II	11	3	4	18	13	4	1	0	5	28
III	7	2	1	10	6	3	0	1	4	40
IV	6	1	2	9	7	1	0	1	2	22
V^e^	8	6	1	15	14	1	0	0	1	7
VI^e^	6	1	0	7	7	0	0	0	0	0

a Detected using the Recombination Analysis Tool and/or Bellerophon numerical screening methods among three different sets of genes of each *msg* family: full-length, full-length plus partial genes, or full-length plus pseudogenes.

b The number of potential mosaic genes among the *msg* families was almost significantly different (*P* = 0.06, chi-square test).

c Six full-length mosaic genes were detected twice but with different pairs of putative full-length parent genes according to the set of genes analyzed (four of family I, one of family II, and one of family III). One mosaic gene of family I was detected twice: once with one full-length gene and one pseudogene as parents and once with two partial genes as parents. All 10 remaining genes were detected only once with a pair of full-length parents.

d Six mosaic pseudogenes of family I had two pseudogenes as parents. Two of family I had one full-length gene and one a pseudogene as parents. The three remaining had a pair of full-length parents.

e Several potential recombination events were detected for these two families using the more sensitive method TOPALi based on the hidden Markov model (see Fig. S9 at http://www.chuv.ch/microbiologie/en/imu_home/imu-recherche/imu-research-groups/imu-research-phauser/imu-supplementary_data.htm).

One to four potential recombination events per mosaic gene were generally identified by the two screening methods. These events were most often confirmed by the more sensitive method, which, however, detected many other potential recombination events (Fig. 4; see Fig. S8 at http://www.chuv.ch/microbiologie/en/imu_home/imu-recherche/imu-research-groups/imu-research-phauser/imu-supplementary_data.htm). Consistent with the single mosaic gene detected in families V and VI, the frequency of recombination events appeared lower in these families than in the others (see Fig. S9 at http://www.chuv.ch/microbiologie/en/imu_home/imu-recherche/imu-research-groups/imu-research-phauser/imu-supplementary_data.htm). This correlated with an average pairwise identity lower within each of these two families than within the others (45 to 66% versus 71 to 83% [Table 1]). The predicted sites of the recombinations reported by all three methods were distributed randomly along the *msg* genes for all families and did not contain any specific DNA sequence motifs (Fig. 4; see Fig. S8 and S9). This suggested homologous rather than site-specific recombination events.

In contrast, we were unable to detect recombination events between different *msg* families, even using the more sensitive method (see Fig. S10 at http://www.chuv.ch/microbiologie/en/imu_home/imu-recherche/imu-research-groups/imu-research-phauser/imu-supplementary_data.htm).

### Comparison to the *msg* superfamily previously proposed.

The 146 *P. jirovecii msg* genes larger than 1.6 kb reported by Ma et al. (4), out of a total of 179, were added to our DNA phylogenetic tree. They all clustered within our families, except 11 outliers clustering with our outliers (see Fig. S11 at http://www.chuv.ch/microbiologie/en/imu_home/imu-recherche/imu-research-groups/imu-research-phauser/imu-supplementary_data.htm). The correspondence between the two sets of families and the comparison of the two studies are detailed in note 6 in Text S1.

## DISCUSSION

Antigenic surface variation plays a crucial role in escaping the human immune system and adhering to host cells for important microbial pathogens. In the present study, we investigated the mechanisms believed to be used by the fungus *P. jirovecii* for this purpose. Our observations show that its surface glycoproteins diversified during the evolution into a superfamily, including six families each with its own structure, function, independent mosaicism, and expression mode.

### Structure and function of Msg glycoproteins.

Members of Msg family I were previously demonstrated to adhere to the human epithelial cell through binding to fibronectin and vitronectin (18, 19). The ST-rich regions present in *P. jirovecii* Msg glycoproteins (except those of family IV) are sites of oxygen-linked glycosylation commonly involved in cell to cell adhesion (20). Moreover, most of these glycoproteins were predicted to be adhesins (see note 7 [62] in Text S1). Consistently, their structure fits the model of modular organization of fungal adhesins with ST-rich regions at the C terminus and a ligand binding domain at the N terminus (20, 21). Linder and Gustafsson (21) proposed that, in addition to their role in adhesion, the oxygen-linked glycosylations of the ST-rich region confer rigidity to the protein in order to present outward the ligand domain. Thus, the N-terminus regions of the *P. jirovecii* adhesins may correspond to ligand binding domains. The fate and function of the glycoproteins of family IV remain enigmatic since they lack the ST-rich region, are only weakly predicted as adhesins (see note 7 in Text S1 and Table S4 at http://www.chuv.ch/microbiologie/en/imu_home/imu-recherche/imu-research-groups/imu-research-phauser/imu-supplementary_data.htm), and may not be attached to the cell wall in the absence of a GPI anchor signal. The conserved leucines separated by two to six residues present in all *msg* families are similar to leucine zipper motifs, which are often involved in protein-protein nonspecific binding and protein dimerization (22). The latter function is also carried out by the PE-rich region present in *msg* families V and VI (23). The conserved coiled-coil domains discovered in Msg families I to III are often involved in the formation of heteromultimers and protein complexes (24, 25). On the other hand, the unstructured regions at the C terminus present in four Msg families are not informative because these regions can have several different functions (26). These observations suggest that the Msg adhesins may form homo- or hetero-oligomers at the cell surface, possibly implying a further level of antigen variation that has never been envisaged so far.

### Mosaicism of *msg* genes.

Our observations suggest that a continuous and random creation of mosaic genes by homologous recombinations occurs mostly, if not exclusively, within each *msg* family. Very interestingly within the scope of protein annotation, this mechanism permits by itself definition of the members of a protein family without having to rely upon the cutting of a phylogenetic tree at an arbitrary height. The frequency of these recombinations remains to be quantified precisely, but it is likely to be reduced in *msg* families V and VI. The genetic mechanisms involved in the creation of mosaic genes may include a single homologous recombination leading to a telomere exchange or two homologous recombinations leading to a gene fragment conversion or exchange (models are shown in Fig. S12 at http://www.chuv.ch/microbiologie/en/imu_home/imu-recherche/imu-research-groups/imu-research-phauser/imu-supplementary_data.htm). Such recombinations could also produce partial genes if they occur between homologous regions that are not located at the same position along the recombining genes. Our results suggest that this is rare because we identified only three partial *msg* genes out of 113. This conclusion is also consistent with the fact that different motifs are conserved along the sequence of the Msg proteins of each family. Our data suggest that pseudogenes might also be involved in the generation of mosaic genes and thus might constitute a reservoir of sequences that can be integrated into functional antigens. The pseudogenes may result from accumulation of mutations in the absence of expression and thus of selective pressure. This phenomenon could be enhanced by mutation and recombination rates within the subtelomeric gene families higher than those in the rest of the genome (8). The presence of the pseudogenes in the subtelomeres might simply correspond to the state between their birth and their future decay. However, they could also be maintained within the subtelomeres through indirect selective pressure because of their role as a reservoir of fragments for the creation of mosaic genes.

### Mutually exclusive expression of *msg-I* genes.

**FIG 5:**
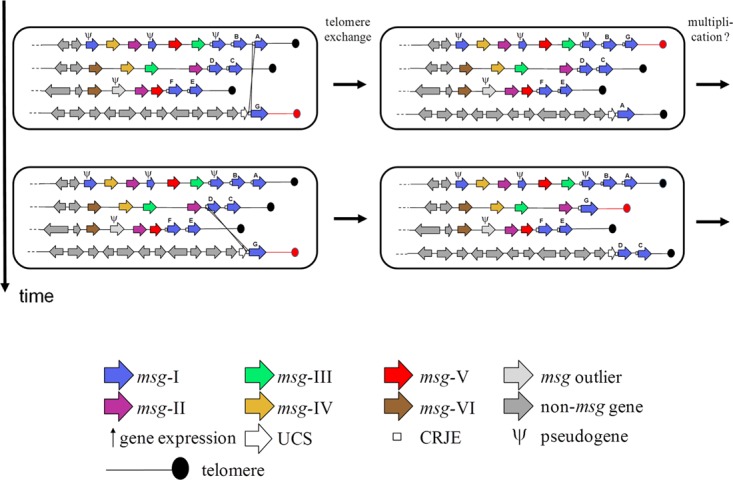
Telomere exchange model for swapping the *msg-I* expressed gene through a single recombination between CRJE sequences. One exchanged telomere is shown in red. Subpopulations of cells expressing a potentially new mosaic *msg-I* gene are generated over time and may then multiply. Polycistronic expression of two *msg-I* genes is shown in the second subpopulation generated (see the text).

Our conclusions concerning the mutually exclusive expression of the *msg-I* genes are in agreement with those of previous studies but bring support for the involvement of telomere exchange, which has been previously hypothesized (27). The exchange of the single expressed gene by recombination at the CRJE sequences might be facilitated by the localization of the *msg*-I genes closest to the telomeres, because this may in turn facilitate telomere exchanges (a model is shown in Fig. 5). These recombinations could be homologous in nature because the full identity over 33 bp might be sufficient as is the case in fungal cousins (28). However, they could also be site specific because the imperfect inverted repeat present in the CRJE is a common motif used by site-specific recombinases (29). Up to three *msg-I* genes were present at the end of the subtelomeres. There is no reason to exclude that transfer of more than one *msg-I* gene to the expression site at once also occurs, followed by polycistronic expression. The polypeptide produced could then be chopped by the endoprotease Kex-1 at the end of each CRJE and each Msg-I anchored to the cell wall separately through its own GPI signal. Interestingly, we detected *msg-I* pseudogenes linked to the UCS by using PCR in our sample. The cells expressing such truncated antigens may not be selected over time during the infection because of their likely deficiency in adhesion to host cells. They might constitute a cost inherent in such a system of antigenic variation based on frequent recombination events.

### Expression of *msg* families.

Transcriptome sequencing (RNA-seq) analyses suggested that the vast majority of the *msg* genes of all families were expressed in *P. carinii* and *P. murina* populations (4). As far as *P. jirovecii* is concerned, alignment of our previous RNA-seq data (6) with the subtelomeres assembled in the present study was compatible with the same conclusion, although the data were from different clinical isolates (results not shown). Expression of most *msg-I* genes at the population level is consistent with the numerous subpopulations of cells expressing different *msg-I* genes that we observed. As far as *msg* families II to VI are concerned, the RNA-seq data are compatible with constitutive or temporally regulated expression of all genes in each cell driven by the promoter present upstream of each of these genes. However, they are also compatible with mutually exclusive or partially exclusive expression of these genes thanks to silencing of promoters or through another unknown mechanism.

### Cell surface structure.

The UCS is a strong promoter (13), probably leading to the majority of adhesive Msg-I antigens on the cellular surface being represented by a single isoform. This is consistent with the fact that Msg-I proteins are the most abundant at the cell surface (13). The surface of *P. carinii* trophic cells was shown to harbor also the surface protein INT1 participating in adhesion (30). Recently, a transcription factor responsible for expression of a still unidentified adhesive surface protein (or proteins) has been reported in *P. carinii* trophic cells (31). Genes encoding orthologs of these two latter proteins are also present in the *P. jirovecii* genome (results not shown). Moreover, Kottom and Limper (31) mentioned that other uncharacterized genes that are important in binding to mammalian hosts are present in the *P. carinii* genome. Thus, the structure of the *P. jirovecii* cell surface is made of a complex mixture of different proteins.

### Strategy of antigenic variation.

The exchange of the *msg-I* isoform expressed and the generation of new mosaic genes of all *msg* families probably lead to a continuous segregation of subpopulations with a new mixture of glycoproteins at the cell surface. Thus, the strategy of the fungus would consist of the continuous generation of cells that are antigenically different. This strategy is further suggested by other characteristics of *Pneumocystis* spp. First, there is a high variability of the subtelomeres between *P. jirovecii* isolates (4), which is consistent with frequent subtelomeric recombinations. The subtelomeres of the isolate we studied here also differed greatly from those of the same chromosomes reported by Ma et al. (4) (see note 6 in Text S1). Second, sexuality could be obligatory in the cell cycle (2, 3) because ectopic recombinations between subtelomeres occur during meiosis, within the bouquet of telomeres formed (8). The likely primary homothallic sexuality of *Pneumocystis* spp. (32) avoids the need to find a compatible partner and thus increases mating frequency, which is believed to favor genetic diversity (33). Moreover, the genetic diversity might be enhanced by mating between the numerous coinfecting strains that are generally present in *P. jirovecii* infections (15). Third, the presence of several *msg* families may allow the formation of the Msg hetero-oligomers that we envisage above, which could further enhance the cell surface complexity.

### Strategies of antigenic variation in different human pathogens.

The mechanisms and hypothesized strategy of antigenic variation unraveled here appear unique among human pathogens. *Candida glabrata* contain one subtelomeric family of ca. 20 adhesins (7). *Trypanosoma brucei* presents a large reservoir of sequences used to create mosaic genes of a single surface antigen family made up of about 1,000 genes located in subtelomeres as well as on minichromosomes (7). In the latter organism, pseudogenes provide segments to mosaic functional antigens (34), a phenomenon that might also occur in *P. jirovecii*. *Plasmodium falciparum* harbors one subtelomeric antigen family of ca. 60 members (7). These three organisms present a single gene family subject to mutually exclusive expression involving silencing in several cases. Thus, their populations are homogenous antigenically but may vary over time when the expressed gene is exchanged. Such a strategy might be imposed by sterile niches such as blood and the urinary tract. This contrasts sharply with the putative strategy of antigenic variation of *P. jirovecii* consisting of the continuous production of a mixture of cells antigenically different. The latter strategy may be associated with the particular niche within lungs since this niche tolerates the presence of low abundance fungi as members of the natural lung microbiota. This strategy might allow presenting most cells as different organisms to the immune system and thus having them tolerated during colonization. A similar strategy might be used by *Candida albicans* living in nonsterile mucosal niches. Indeed, its unique adhesin family presents a high number of serine CUG codons that are ambiguously translated into serine or leucine, thus creating variability from individual genes (35).

*Trypanosoma* and *Plasmodium* also differ from *Pneumocystis* spp. in that they infect two different hosts rather than one. This undoubtedly exerts a different selective pressure on their antigenic variation system. The *Pneumocystis* spp. differ considerably in their *msg* families (4), as well as in the fine structure of the Msg adhesins (36). It is likely that these differences are involved in the strict host species specificity of these fungi. Further work aiming at understanding the relation between structure and function of the different Msg glycoproteins is needed to further decipher both the antigenic variation and host specificity of these fungi.

### Conclusions.

Several conclusions can be drawn from our observations. First, the *P. jirovecii* cell surface appears to be made of a complex mixture of different surface proteins, with the majority represented by a single isoform of the most abundant *msg-I* glycoprotein. Second, genetic mosaicism within each *msg* family probably ensures variation of the surface glycoproteins. Third, the strategy of the fungus seems to consist of the continuous production of new subpopulations composed of cells that are antigenically different. Such a strategy is unique among human pathogens and might be associated with the particular niche within host lungs. Given the role of surface antigen variation and regulation in immune avoidance in other human pathogenic organisms, we postulate that antigenic variation may provide a similar role in the life cycle of *P. jirovecii*.

## MATERIALS AND METHODS

### Ethics approval and consent to participate.

The protocol was approved by the institutional review board (Commission Cantonale d’Éthique de la Recherche sur l’Être Humain). All patients provided informed written consent, which was part of the procedure for admittance in the hospital. The admittance paperwork included the possibility to ask that their samples not be used for research. The samples were treated anonymously and were collected through a routine procedure at the hospital.

### Bronchoalveolar lavage fluid specimens.

Fresh BALFs positive for *P. jirovecii* using methenamine-silver nitrate staining (37) were supplemented with 15% (vol/vol) glycerol, frozen in liquid nitrogen, and stored at −80°C. Only those with more than 1 ml available and a heavy fungal load were stored. Seventeen specimens were stored between 2012 and 2014 and used for the selection procedure described here below.

### DNA extraction and identification of an infection with a single *P. jirovecii* strain.

Genomic DNA was extracted from 0.2 to 0.4 ml of BALF specimen using the QIAamp DNA minikit (Qiagen) and resuspended in 50 μl of elution buffer. Four genomic regions were amplified by PCR from genomic DNA extracted as described previously (38). Each PCR product was cloned into the plasmid pCR4-TOPO using the TOPO TA Cloning kit for sequencing (Life Technologies, Inc.). Both strands of the insert of 15 clones for each genomic region were sequenced with M13 primers using the BigDye Terminator kit and the ABI Prism 3100 automated sequencer (both from PerkinElmer Biosystems). Among the 17 clinical specimens collected, only one generated identical sequences for all clones of all genomic regions. Since ca. 15 clones per genomic region were analyzed, a second eventual coinfecting strain in this specimen should not represent more than ca. 7% of the *P. jirovecii* population. This specimen was selected for all experiments performed in the present study. It was from an HIV-infected patient. The genotype of the *P. jirovecii* strain present in this specimen was as follows (the nomenclature refers to that used by Hauser et al. [38]): allele B of internal transcribed spacer 1 of the nuclear rRNA gene operon (T at position 2, 2× T at positions 8 to 10, A at position 11, T at position 17, T at position 22, TC at positions 46 to 47, 10× T at positions 54 to 62, GAGG at positions 71 to 72, and TTA at positions 111 to 113), allele 8 of the variable region of the mitochondrial 26S rRNA gene (4× A at positions 54 to 57, T at position 85, C at position 248, and G at position 288), the reference allele of the intron of the nuclear 26S rRNA gene (GenBank accession no. L13615 [A at positions 3, 78, and 212, T at position 296, and C at position 305]), and the reference allele of the β-tubulin intron 6 region (A at position 24 and G at position 282).

### Enrichment in *P. jirovecii* DNA and random amplification.

The DNA of the selected specimen was enriched in *P. jirovecii* DNA using the NEBNext microbiome DNA enrichment kit based on the absence of CpG methylation (Biolabs), purified by ethanol precipitation in the presence of 10 μg glycogen (Thermo Fisher Scientific), and resuspended in 50 μl of 1× Tris-EDTA (TE) buffer. This enrichment raised the proportion of *P. jirovecii* DNA from a few percent to ca. 55% as determined *a posteriori* by high-throughput sequencing. Because only small amounts of DNA are recoverable from a clinical specimen and in absence of an *in vitro* culture system, a sufficient amount of DNA for high-throughput PacBio sequencing was obtained by random amplification. Five microliters of DNA was randomly amplified in a 50-μl reaction using the Illustra GenomiPhi HY DNA amplification kit (GE Healthcare). This amplification proved to create artificial molecules made of inverted repeats of several kilobases, which were revealed by PacBio sequencing. The reads from these molecules were eliminated by bioinformatics (described below). DNA was then purified using the QIAamp DNA blood minikit (Qiagen) followed by ethanol precipitation in the presence of 10 μg glycogen. Amplified DNA fragments were sized (mean of 8.6 kb) and quantified using a fragment analyzer (Advanced Analytical).

### High-throughput PacBio sequencing.

Five micrograms of amplified DNA was used to prepare an SMRTbell library with the PacBio SMRTbell Template Prep kit 1 according to the manufacturer’s recommendations (Pacific Biosciences). The resulting library was size selected on a BluePippin system (Sage Science) for molecules larger than 5 kb. The recovered library was sequenced on one SMRT cell with P6/C4 chemistry and MagBeads on a PacBio RSII system (Pacific Biosciences) at a movie length of 240 min.

### Read filtering and *P. jirovecii* genome assembly.

The flow chart of the filtering and assembly procedure is shown in Fig. S13a at http://www.chuv.ch/microbiologie/en/imu_home/imu-recherche/imu-research-groups/imu-research-phauser/imu-supplementary_data.htm, and the details for each step are described here. PacBio subreads were extracted from the raw h5 files using DEXTRACTOR (https://github.com/thegenemyers/DEXTRACTOR/). The average length of the extracted subreads was 5.2 kb, with a maximum length of 42 kb. We removed human-derived reads by mapping them against the human reference genome using BLASR (smrtpipe 2.3 [cutoff corrected score of <55,000). Reverse-complementary artificial reads created by the random amplification were next filtered out (cutoff match length of ≥1,000 bp) after mapping them onto themselves using DALIGNER (https://github.com/thegenemyers/DALIGNER/) (v1.0 [options -A and -I]). The cleaned reads were assembled using the tool FALCON (39) (v0.2 [options length cutoff=8000m and length_cutoff_pr=1000]). PacBio reads were remapped onto the assembly using BLASR and used to evaluate and flag the remaining human contigs. Human-derived contigs were subsequently removed. A total of 2.2 Gb of *P. jirovecii* DNA sequences corresponding to a 200-fold coverage of the genome were gathered. The assembly was polished to remove residual PacBio errors using Quiver (smrtpipe 2.3 [5 iterations]) (40). The final polished genome assembly included 8.1 Mb in 219 gap-free contigs ranging from 234 bp to 386 kb, with an NG_50_ of 108 kb and 57% of the genome in 28 contigs larger than 100 kb. The *P. jirovecii* PacBio assembly obtained in the present study covered 96% of that we previously obtained by other sequencing methods (6) and contained ca. 0.5 Mbp of subtelomeric sequences. The combination of both our assemblies covered 97% of the assembly of Ma et al. (4). Controls consisting of PCR amplification of specific subtelomeric regions from the same DNA sample confirmed the accuracy of the nucleotide sequence of the polished PacBio assembly, although few errors in repetitive homopolymer regions were detected (see note S8 in Text S1).

### Gene predictions and *msg* annotations.

Genes were predicted on the assembly using Augustus (version 2.5.5) (41) and a specifically trained model for *Pneumocystis* (6). In order to detect novel and more distant homologous *msg* genes in the assembly, we chose a generalized profile-based approach (16) (see Fig. S13b at http://www.chuv.ch/microbiologie/en/imu_home/imu-recherche/imu-research-groups/imu-research-phauser/imu-supplementary_data.htm). A DNA profile was generated based on a previously described *msg* gene in *P. carinii* (GenBank accession no. D82031.1) (42) and a protein profile based on Msg-Rucl 21 (European Nucleotide Archive no. ABQ51002.1) using a Smith-Waterman algorithm (43). The profiles were calibrated against the scrambled genome (window approach, size 60). Using pfsearchV3 (44), the assembled genome was searched for homologous matches with the DNA profile. Curated matches were extracted and aligned against each other using MAFFT (version 7.305) (45). After manual curation and trimming, the alignments were divided into five groups based on neighbor joining (percentage of identity) using Jalview (v2.8.1) (46). One representative candidate per group was selected, and a new profile based on its sequence was generated and calibrated as described above. These DNA *msg* profiles were used to find and annotate a first set of 75 *msg* genes in the assembly. A combination of BLASTX, GeneWise, in-house tools, and manual curation was applied using the protein Msg profile to extend and correct these annotations to the set of 113 *msg* genes analyzed in the present study. The *msg* genes reported here were all manually curated with respect to their start, stop, and intron coordinates.

### Construction of phylogenetic trees.

For the DNA- and protein-based phylogenetic analysis, the CDS for each annotated *msg* gene was manually corrected (up to five corrections), extracted, and translated into its protein sequence. Both CDSs and protein sequences were aligned against each other using MAFFT (mafft-linsi—genafpair) (45), and the multiple sequence alignment used to infer a phylogenetic tree with RAxML (PROTGAMMAGTR for proteins and with GTRGAMMA for CDS [1,000 bootstraps]) (47). The *msg* genes of family V were defined as the out-group and the final tree rooted. Proteins were further classified using JACOP (http://myhits.isb-sib.ch/cgi-bin/jacop/) (48). In order to add pseudogenes and published *msg* genes from Ma et al. (4) of ≥1.6 kb, we injected the new sequences into the prior DNA-based multiple alignment using MAFFT (—addfull) (45). They were added to the original tree using the evolutionary placement algorithm (EPA) from RAxML. These trees were converted into a compatible format with the tool guppy from the pplacer suite (v1.1alpha14, tog) (49). Genes with an exon smaller than 1.6 kb were added to the original DNA-based multiple-alignment using MAFFT (—addfragments) (45). The alignment was trimmed and realigned using MAFFT (45). A new tree was then built with RAxML (GTRGAMMA [1,000 bootstraps]). All trees were analyzed and visualized using R (v3.3.2) (50) and GGTREE (v1.6.9) (51).

### Gene and protein sequence analyses.

Alignments of full-length gene or protein sequences were carried out using MAFFT (45). Canonical TATA box and Cap signal (52), as well as canonical donor and acceptor sequences of *Pneumocystis* introns (53, 54), were identified by visual inspection of the alignments and sequences of the genes. Signal peptide and GPI anchor signal were identified using Phobius (http://phobius.sbc.su.se/) (55) and GPI-SOM (http://gpi.unibe.ch/) (56), respectively, with default settings. Canonical potential sites NXS/T of nitrogen-linked glycosylation (21) were identified by visual inspection. Conserved domains were searched using MEME (multiple expectation-maximization for motif elicitation [http://meme-suite.org/tools/meme]) (17). MEME analysis of the 49 full-length Msg proteins of all families except outliers was carried out using default settings, except for the minimum and maximum motif widths of 50 and 100 residues, respectively, the option any number of sites per sequence, and a maximum of 13 motifs searched. HMMER (biosequence analysis using the profile hidden Markov models [HMM; http://www.ebi.ac.uk/Tools/hmmer/search/hmmscan]) (57) was used with default settings on full-length proteins for the following embedded predictions: Pfam, unstructured regions (intrinsically unstructured proteins [IUPRED]), and coiled-coil motifs (Ncoils predictor). Pairwise identities between full-length *msg* genes and Msg proteins were calculated using the multiway alignment type of Clone Manager 9 professional edition software.

### Search for potential mosaic genes.

Two screening methods were first used: Recombination Analysis Tool (RAT [http://cbr.jic.ac.uk/dicks/software/RAT/]) (58) and Bellerophon (http://comp-bio.anu.edu.au/bellerophon/bellerophon.pl) (59). MAFFT (45) alignments of various sets of genes were analyzed by both methods. RAT was used with default settings (i.e., using windows of one-tenth of the length of the alignment and an increment size equal to half of the window size). Bellerophon was used with default settings (i.e., windows of 300 bp and Huber-Hugenholtz correction). RAT can detect several recombination events, whereas Bellerophon reports a single one per mosaic gene. The more sensitive method, TOPALi (v2.5 [http://www.topali.org/]) (60), which is based on a hidden Markov model (HMM), was then applied on the potential mosaic genes, and its putative parent genes were detected by the two screening methods. These three genes were aligned using MAFFT (45), with an additional gene chosen randomly in the same *msg* family since TOPALi requires input of four genes. The efficacy of the three methods to detect mosaic genes was assessed by the analysis of artificial chimeras produced *in silico* with related genes, as well as with sets of orthologous genes from different fungal species (results not shown). Only the RAT method is suitable for the search of recombination events among proteins. The vast majority of the events detected at the protein level corresponded to those detected at the DNA level (results not shown).

### PCR amplification and sequencing.

PCRs were performed in a final volume of 20 μl with 0.35 U of Expand high-fidelity polymerase (Roche Diagnostics), using the buffer provided, each deoxynucleoside triphosphate (dNTP) at a final concentration of 200 μM, and each primer at 0.4 μM. The PCR conditions included an initial denaturation step of 3 min at 94°C, followed by 35 cycles consisting of 30 s at 94°C, 30 s at the annealing temperature, and 1 min per kilobase to be amplified at 72°C. The reaction ended with 5 min of extension at 72°C. The annealing temperature and the MgCl_2_ concentration were optimized for each set of primers and ranged from 51 to 60°C and from 3 to 6 mM, respectively. Sequencing of both strands of the PCR products was performed with the two primers used for PCR amplification, as well as the BigDye Terminator DNA sequencing kit and ABI PRISM 3100 automated sequencer (both from PerkinElmer Biosystems).

### Accession number(s).

PacBio raw reads (accession no. SRR5533719) and the PacBio assembly (accession no. NJFV00000000) have been deposited in the NCBI Sequence Read Archive linked to BioProject accession no. PRJNA382815 and BioSample accession no. SAMN06733346.

### Availability of data and materials.

The data sets generated and analyzed during the present study are available from the corresponding author on reasonable request.
