# Supplementary material for: Mechanisms of Surface Antigenic Variation in the Human Pathogenic Fungus Pneumocystis jirovecii
Source: mBio. 2017 Nov 7;8(6):e01470-17. doi: 10.1128/mBio.01470-17 (PMC5676039; doi:10.1128/mBio.01470-17)
Supplement: TEXT S1 [file mbo005173568s1.docx]

**Text S1**

**Supplementary notes**

**1. Identification of the *msg* pseudogenes**

Classification of the genes as pseudogenes was a difficult task because the stop codons along the genes and PacBio sequencing errors among the contigs were unevenly distributed. Thirteen of the 16 genes of family I with more than five stop codons missed the CRJE sequence. Eleven of the 12 genes of families II to VI with more than five stop codons missed TATA box, signal peptide, and/or GPI anchor signal. The lack of these motifs suggested that the genes were not expressed or not functional. On the basis of these observations and the results of the control PCRs described below (Supplementary note 8), we decided to classify genes with more than five stop codons as pseudogenes. The number of stop codons varied between five and several tens, and there were no motifs present systematically in the sequences at or around the stop codons, such as homopolymers. The sequence of 800 to 1200 bps fragments of four pseudogenes was confirmed by specific PCR amplification followed by sequencing.

**2. Absence of telomeric repeats**

Telomeric TTAGGG repeats previously reported in *P. jirovecii* (4) were not present in the *de novo* PacBio assembly, neither in the raw reads. Similarly, PCR amplification from the DNA sample did not result in their recovery. Our present hypothesis is that the enrichment in *P. jirovecii* DNA which we used based on the absence of CpG methylation (see Methods) may have eliminated the telomeres, although such methylation is not expected in such lower eukaryotes.

**3. Retrieval of the UCS**

Sequence comparison with our PacBio assembly revealed that the sequence upstream of the UCS in the assembly of Ma et al (4) was identical to one end of our PacBio contig 72. Using primers in this end and in the published UCS sequence, we succeeded in amplifying from our DNA sample a linking fragment. Further amplification using a primer just upstream of the CRJE localized at the 3’ end of the UCS allowed the retrieval of the full UCS from our sample. Relatively to the sequence published by Ma et al (4), the UCS from our sample presented a supplementary segment of 21 bps which creates a tandem repeat upstream of the promoter, and a few silent polymorphisms within the ORF (Fig. S7). It harbours two tandem repeats within its intron which are of type “1,1” according to Ma et al (61).

**4. Identification of the expressed *msg*-I genes**

In order to amplify several *msg*-I genes linked to the UCS at the same time, we used the generic PCR amplification described by Kutty et al (12). One of the primers is located close to the 3’ end of the UCS (primer GK163) and the second one at position ca. 1600 in many *msg*-I genes (GK160). The PCR product obtained of ca. 1700 bps from the DNA of the selected specimen (not enriched, not randomly amplified) was subcloned using TOPO TA cloning kit (see Methods), and the insert of each subclone was sequenced using M13-21 and M13 reverse primers, as well as internal primers. The obtained sequences were aligned by BLAST against the PacBio assembly. Out of 31 clones analysed, nine harboured *msg*61, seven *msg*93, six a new *msg*-I gene not present in the PacBio assembly, three *msg*41, two *msg*78, and two pairs a new *msg*-I gene.

For amplifying specific *msg*-I genes present in the PacBio assembly linked to the UCS, we performed 21 PCRs using the primer GK163 located close to the 3’ end of the UCS, and one primer designed in the region between ca. 400 and 1000 within the *msg*-I gene. Both strands of the PCR product were then directly sequenced.

**5. Sub-clades of *msg* family I**

In agreement with our results, frequent recombination events between *P. jirovecii msg*-I genes were previously reported (14). These authors also observed the two *msg*-I sub-clades and analyzed specific regions of sequence alignments of members of these clades. They reported the presence of short segments in all *msg* genes of one sub-clade but in only one or few genes of the other sub-clade, an observation that we also did in our set (results not shown). This suggested that inter sub-clade recombination events occur, but less frequently than intra-sub-clade events. The presence of these two sub-clades might be related to the fact that the *msg*-I genes are located distally within the subtelomeres which could favor the creation of mosaic genes through a single homologous recombination concomitant with a telomere exchange. Kutty et al (14) proposed the alternative hypothesis that these two sub-clades are inherited systematically from sexual reproduction, a process which is thought to be obligatory during the *P. jirovecii* cell cycle (2, 3). However, this latter hypothesis seems less likely because the phenomenon would a priori also concern the other *msg* families, which we did not observe.

**6. Comparison of our results to those of Ma et al (4)**

Our observations are fairly consistent with the different *msg* gene families proposed by Ma et al (4). Our families *msg*-I, -IV, -V, and -VI correspond respectively to their families *msg*-A1, -B, -D, and –E. Our families II and III correspond both to their subfamily A3. Nevertheless, their subfamily A3 clearly encompasses two clusters in the phylogenetic tree of their Figure 3a. These two clusters may correspond to our families II and III, although this could not be ascertained in absence of detailed data. We found that these two families probably recombine very rarely if ever between each other, so that they should be considered as distinct. We did not identify the family *msg*-C including only two members described by Ma et al (4) among our set. Indeed, the *msg*-C gene larger than 1.6 kb (accession number T551_03109) that could be introduced in our tree clustered close to the *msg*-I family (Fig. S11). Consistently, BLAST analyses revealed that the smallest *msg*-C gene of 968 bps (T551_01654) showed homology to three of our *msg*-I genes, two of which being pseudogenes (Table S1). Moreover, its 968 bps were identical with part of our pseudogene *msg*38 which is 2754 bps long. Analysis of the region surrounding this *msg*-C gene revealed that our pseudogene was present and 100% identical in the Ma et al (4) assembly, except five single bp polymorphisms. The two Msg-C proteins are present in close clusters of their tree which are poorly supported with bootstrap values of only 0 and 15 (intriguingly, many other such low values are present in their tree which includes also *P. carinii* and *P. murina* proteins). The proposal of the existence of family Msg-C might be linked to the fact that they did not describe any pseudogenes in their work. This could result from the use of automated gene prediction and annotation, although this is not stated. Consistently, the proportions of the corresponding families were roughly similar in the two studies (see our Table 1 and their supplementary Table 3).

The arrangement of the different *msg* families within the *P. jirovecii* subtelomeres that Ma et al (4) reported corresponds almost fully to ours, *i.e.* with the *msg*-VI genes distal and the *msg*-I genes proximal to the telomeres. One exception is a *msg*-A3 gene closer to the telomere than a *msg*-A1 gene in the subtelomere of chromosome 5, a situation that we never observed. Some of their as well as our subtelomeres do not include *msg*-I genes at their tip, a fact that could result from incomplete assembly. Similarly, incomplete assembly could explain that seven of the 20 chromosomes miss one subtelomere in Ma et al (4).

Comparison of the number and identity of the *msg* genes present on the same chromosomes revealed that they differed significantly between the *P. jirovecii* isolates analyzed in the two studies (for example, compare contigs 18 and 55 of Fig. 3 with the two subtelomeres of chromosome 7 in their supplementary Figure 1c). On the other hand, the single copy UCS was linked in both assemblies to chromosome 1, which, interestingly, is the largest one.

The conserved domains we identified correspond well with those described by Ma et al (4) (their Fig. 3b). Their domains N1, M1, M2, M3, M4, M6, C1, and C2 correspond respectively to our domains 1, 2, 3+4, 5+6, 7+8, 9, 10, and 11. Their domain M5 corresponds to the second copy our domain 2 present in our families I to III. They did not report domains corresponding to our domains 12 and 13 which include respectively the signal peptide and PE-rich region. The distribution of the domains among the families present some differences between the two studies. The domain N1 present at the N-terminus of all their families (our domain 1) was not conserved in our families IV, V, and VI. The domain M1 (our domain 2) was not present in our family IV (their family B). The domain C2 including the GPI-anchor signal (our domain 11) present only in their family A was also present in our families V and VI. These differences may result from the use of different settings and/or set of proteins for the analyses with MEME.

Ma et al (4) reported introns only in *P. carinii* specific *msr* genes (subfamily *msg*-A2), but not in *P. jirovecii* *msg* families II to VI as we demonstrated here. Moreover, they did not report the conserved motifs responsible for transcription as well as protein secretion and glycosylation that we evidenced, preventing interpretation concerning expression and function.

**7. Analysis of the Msg proteins using an adhesin predictor**

To investigate the nature of the *P. jirovecii* Msg proteins, we used the online Fungal Adhesins and Adhesin-like proteins predictor (62) with the default threshold score of -0.8 and the hybrid classifier model ACHM (Faapred, <http://bioinfo.icgeb.res.in/faap/>). This predictor uses a dataset made of 341 fungal non-adhesins proteins and 75 fungal adhesins, including two *P. carinii* Msg glycoproteins. Out of the 55 full-length *P. jirovecii* Msg proteins we identified in the present study, including the outliers, 47 were predicted to be fungal adhesins (Table S4 available on [institutional website](http://www.chuv.ch/microbiologie/en/imu_home/imu-recherche/imu-research-groups/imu-research-phauser/imu-supplementary_data.htm)). The results of the six members of family IV with scores close to the threshold of prediction and three members not predicted as adhesins suggested that these might be the only *P. jirovecii* Msg glycoproteins with another function.

**8. Control PCRs**

The accuracy of the PacBio assembly nucleotide sequence was checked by PCR amplification of subtelomeric fragments of 400 to 1600 bps using specific primers from the DNA of the selected specimen (randomly amplified, but not enriched, see Methods). Both strands of 34 different PCR products corresponding to fragments of 18 different PacBio contigs were sequenced. A total of 18 single bp differences were considered as errors in the PacBio assembly because their correction eliminated a stop codon and increased the size of ORF. These errors were all within homopolymers, 14 times missing A/T and four times a G/C. These errors were observed in only seven different PCR products, suggesting that they were not evenly distributed among the contigs. A maximum of five errors was detected in a single amplified fragment. These errors were manually corrected for the extracted and translated CDS. These observations suggested that many errors could be present in a given *msg* gene (ca. 3000 bps), but that most of them had no errors. These residual errors are likely due to alignment uncertainties of PacBio reads onto repetitive *msg* regions during the genome polishing step.
