# Supplementary figures and images for: Mechanisms of Surface Antigenic Variation in the Human Pathogenic Fungus Pneumocystis jirovecii
Source: mBio. 2017 Nov 7;8(6):e01470-17. doi: 10.1128/mBio.01470-17 (PMC5676039; doi:10.1128/mBio.01470-17)

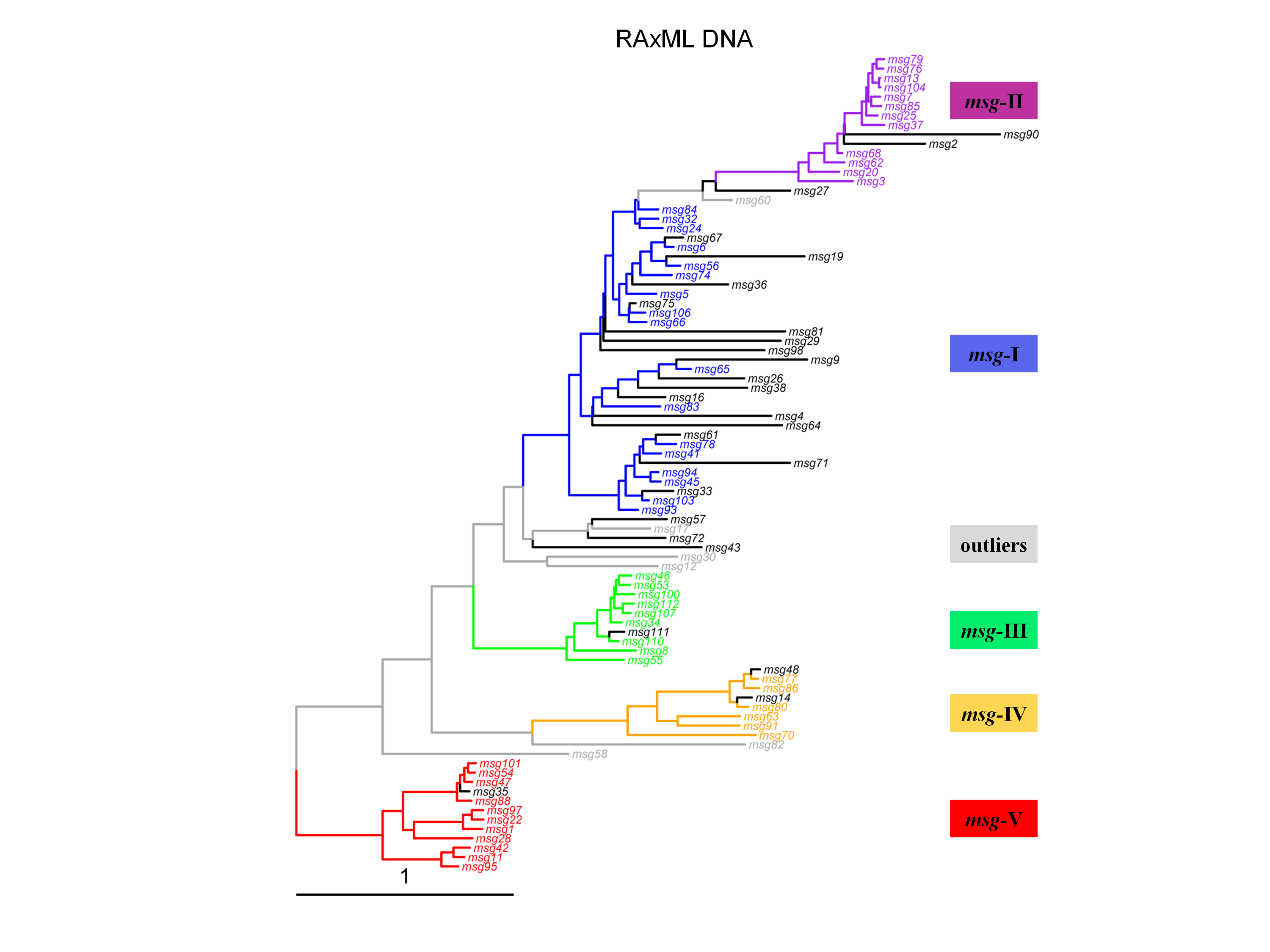

Supplement: FIG S1 [file mbo005173568sf1.tif]

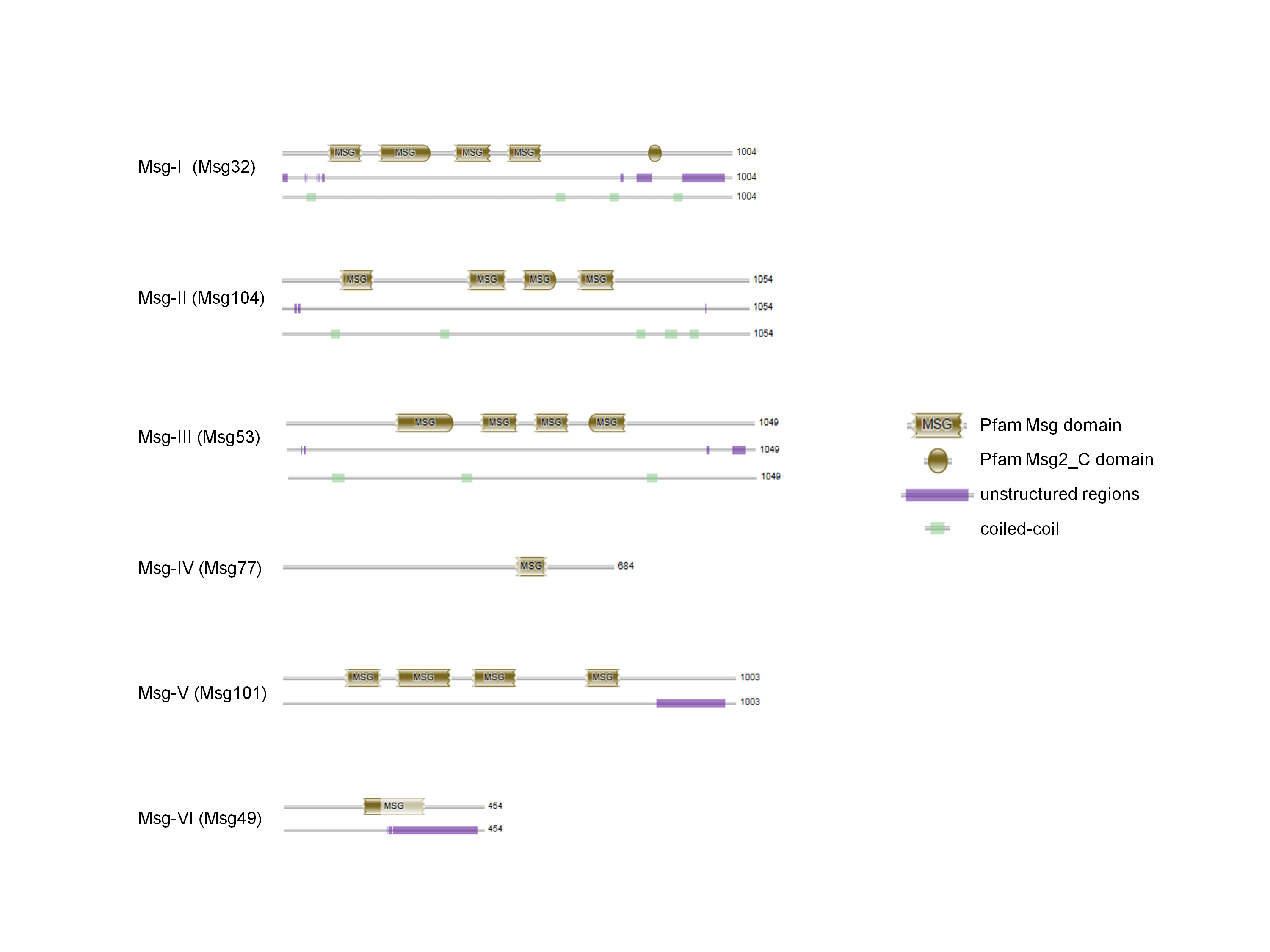

Supplement: FIG S5 [file mbo005173568sf5.tif]
