# Supplementary material for: Mechanisms of Surface Antigenic Variation in the Human Pathogenic Fungus Pneumocystis jirovecii
Source: mBio. 2017 Nov 7;8(6):e01470-17. doi: 10.1128/mBio.01470-17 (PMC5676039; doi:10.1128/mBio.01470-17)
Supplement: FIG S2 [file mbo005173568sf2.docx]

Fig. S2 (85 pages)

***msg*-I** **upstream potential TATA box**

*msg*32 ATTT-------AT---TCA-ATTTT-ACT--------ATTA----C---- 22

*msg*24 ACATTA----TAT---TC--ATTAT-CCA--------AACAT--TT---- 26

*msg*84 TTTCCAGAGTTTT---TTACAT**TATATTC**ATTATCCAAACAT--TT---- 41

*msg*56 TAAAA----CTAT---AAA-AAATCATCA-GAAAAAAATTATTAAT---- 37

*msg*6 TTTCAT---CTAT---TAA-GTTTTACTT-T--TATGATTA---CC---- 33

*msg*94 ACTTGT----TAAGATTTACGAT-TAC-C-C--CACATTTA---CC---- 34

*msg*45 AATTCA----TTT---TCATTT--TTC-T-C--AACAATTA---CTATTT 34

*msg*41 ATATT------TT---TCAC-TT-AAGAT-T--TGAGTTTA---CC---- 29

*msg*103 TTCATC----TAT---TTAAGTT-T--------TACTTTTA--------- 25

*msg*93 CTACTT----TTT---ATCTATTCAAGTT-T--TACGATTA---CC---- 33

*msg*78 CAAACA----TTT---TTGCGTT-TAC-C-C--TGCGTTTA---CC---- 31

*

*msg*32 --CCACATTTACCCCGCATTTACCCCACACAAATTTAAAT-A-CGTGCA- 67

*msg*24 --TTGCGTTTACCCCGCGTTTACCCCACACAAATTTTAACTATCACGAA- 73

*msg*84 --TTGCATTTACCCCGCATTTACCCCACATAAATTTAAAT-A---CGTA- 84

*msg*56 --TAATATTAA--ATACTTTTATAGAACATTTAG-TAAATAA-TTTTTA- 80

*msg*6 --CCGCATTTACCCCGCATTTACCCCACATAAATTTAAAT----ACGTA- 76

*msg*94 --CCGGATTTACCCCGGATTTACCCCATACAAATTTAAAGCA-T-CTCA- 79

*msg*45 GCTAGAATATG---CAATCTTATCGAATTCAAATGCAAACCT-CAATTT- 79

*msg*41 --CCGAATTTACCCCACATTTACCCCACACAAATTTAAAT-A-CGTGTAG 75

*msg*103 -----TGATTACCCCACATTTACCCCACACGAATTTTAACTATCACGAA- 69

*msg*93 --CCGCATTTACCCCGCGTTTACCCCACACAAATTTAAATCG-T--ATA- 77

*msg*78 --CCGCGTTTACCCCGCGTTTACCCCACACAAATTTAAAGTATCGCGAA- 78

*** * * **

*msg*32 GTAAAGCA-GTAT-CACAATA--ATTG-TATGGACACACTAAAGATATAC 112

*msg*24 AC-AACCAGATACACACCAAAACAGAC-AGCGGGCACCTTCTAAA**TATAT** 121

*msg*84 CT-AAGCACGTAT-CATAATA--ATTC-TATGGACACACTAAAGATATAT 129

*msg*56 AT-AAATATTTAA-TTAAATA--AAAA-ATCCTATTTTTTAATTAGAAAC 125

*msg*6 CT-AAGCACGTAT-CATAATA--ATTC-TATGGACACACTAAAGATATAT 121

*msg*94 CG-AAGAAA-T---CAAGATACACTTGTAATGGACACACTAAGAATACGC 124

*msg*45 TT-AAGTAT-TTC-CAAATTATACTCATA-TT-ATTAATTGAAAAGACAC 124

*msg*41 TA-AAGCACGTAT-CACAAT--AATTG-TATGGACACACTAAAGATATAC 120

*msg*103 AT-AATTAA-C---TACATG-TTAGAC-AGCGGGCACTCCTTAAATATAC 112

*msg*93 CT-AAGCACGTAT-CACAAT---ATTG-TATGGACACACTAAAGATATAC 121

*msg*78 GA-A-GCCC-AAC-TACGCA--GAGAC-AGCGGGCACCTCTTAAA**TATAT** 121

* * *

*msg*32 TTCAGCACACACAGTGCTTCTTTG--ACA-AGTTTTGTCACTGAATTTCC 159

*msg*24 **TT**-GGTACACATAATGCCTGTCTA--TCACAGTTATCTCAC--TTTTTAC 166

*msg*84 TC-TGCACACA--TTGCTTGTCTG--ACA-AGTTTTGTCACTGAATTTAC 173

*msg*56 TT--------A--AAACTTAAACAATTCA-TTATTTATCATAGAATTTAC 164

*msg*6 TC-TGCACACA--TTGCTTGTCTG--ATA-AGTTTTGTCACTGAATTTAC 165

*msg*94 TT-AGCACACG--CTGCTTGTCTG--ACA-AGTTTTGTCATTGAATTTAC 168

*msg*45 TT-TAAACATC--GTCA----TTA--ACACAGAAATGGCACAGGAT-TAC 164

*msg*41 TT-TGCACACA--CAGCTTCTCTG--ACA-AGTTTTGTCACTAAGTTTAC 164

*msg*103 TT-AGCACACA--CTGCTCATCTG--GCA-AGTTTTATCACAGAATTTAC 156

*msg*93 TTCAGCACACA--CTATACCTCT--------GTTTCATCACAGAATTTAC 161

*msg*78 **T--T**GCACACA--TTACTTATCTG--ACA-AATTTTACCACAGAATTCAG 164

* ** *

*msg*32 ATAAGAA-TCTT--AATTAAA----AAGAAT-TAATA-TTAATTTC-ATA 199

*msg*24 TTGAACACCCTTAAAATTAATTAAAAATAATTTAATAGTTGA-----CTT 211

*msg*84 TTGAACACCCTTAAAATTAATTAAAAATAATTTAATACTTAA-----ATT 218

*msg*56 ATAGA-A-TCT-GAAATTAACAAAGGAAAATACAACACTTAATTTCAGTA 211

*msg*6 TTGAACACCCTTAAAATTAATTAAAAATAATTTAATACTTAAA-----TT 210

*msg*94 TTGAACTCCCTTAAAATTAATTAAGGATAATTTAATATTCAA-----ATT 213

*msg*45 TTTTTTTCTCAAAAAACTACCTTTAAAACTTTCAATATTCAA-------- 206

*msg*41 TTGAACACCCTTAAAATTA----AGAATAATTTAATATTCGA-----ATT 205

*msg*103 ATAAGAA-TCTTGAAATTAAC---AGAGAATCCAACACTTAATTTCAGTA 202

*msg*93 TTGAAAATTC-TATAATTAATTAGAAATAATTTAATATTCAA-----ATT 205

*msg*78 TTGAGCTTCCTTAAAATTA----AAAATAATTTAATACTTGA-----CTT 205

* * ** ** * * ** * * *

*msg*32 --TGAA-ACATAACTC-TACATCTCACATGTAGTCAAAACACATTAA-CT 244

*msg*24 TATGAAAACAAGCCTC-ACCAACTT--CTACAGTCAAAACACACTAG-CG 257

*msg*84 --TGAAGACATGGTTCTAACATCTCACATGAAATCAAAACACATTAA-CG 265

*msg*56 --TGAAAACATAACTCTAACATTTTACATGAAATAAAAATACATTAA--G 257

*msg*6 --TGAAGACATGGTTCTAAAATCTCACATGCAACCAAAACACGTTAA-TG 257

*msg*94 --TGAAGACATGGCTTTAACATCTCACGTGCAATGAAAACACGTTAA-CG 260

*msg*45 --------CATCTTAATGA-AGACAA**TATAAAAT**AGAAACAGCTTTAAAA 247

*msg*41 --TGAAGACATGACTCTAACATCTCACGTTCAATCAAAATACGCTAA-TG 252

*msg*103 --TGAAAACATAACTCTAATATCCCACGTAAAATTAAAACACATTAA-CG 249

*msg*93 --TGAAGACATGGCTTTAACATCTCACGTGCAATGAAAACACATTAA-CG 252

*msg*78 TATGAGAACAAGACCC-ACCAACTT--TTGCGATCAAAACACATTAA-CG 251

** * * *** * *

*msg*32 C-AAACT--CAAACTCAG--CC-ACACACCACAACGCAA-TCCACCCACT 287

*msg*24 G-AAACT--CAAACTCAA--AC--TACACCACAGCGCG--CCCACT--TC 296

*msg*84 G-AAACT--CAAACTCAA--CC-ACACACCACAGCGAAAATCCACC--CC 307

*msg*56 G-AAAC---CAAACTCAAG-CT-ACACACTACAGCGCA-GCCCAC----C 296

*msg*6 G-AAACT--CAAACTCAG--CC-ACACACCACAGCGCAAACCCACC--CC 299

*msg*94 G-AAGCT--CAAACTCAA--AC--CACGCCACAGCGCAAACCCACC--TC 301

*msg*45 GTCTGCCCACACACCCAACAAG--TACGCTTTCTCTCA----CATT--AA 289

*msg*41 G-AAACC--CAAACTCAA--AC--CACACCACAGCGCG--CCCACC--TC 291

*msg*103 A-AAACT--CAAACTCAA--ACCACACAGAATAACGCAAACCTACC--TC 292

*msg*93 G-AAACT--CAAACTCAG--CC-ACACACCACAACGTAAATCCACC--CC 294

*msg*78 G-AAACT--CAAACTCAG--CC-ACACGCCACAGTGCAAACCCACC--CC 293

* ** ** ** ** *

**<--------------CRJE------------->**

*msg*32 CGTCGGCCACAGCTTGGCGCGGGCGGTGGCGCGGGCGGTTAAGCGGCGGG 337

*msg*24 CGTCAGGCACAGCTTGGCGCGGGCGGTGGCGCGGGCGGTCAAGCGGAGGG 346

*msg*84 CATCTGGCACGGCTTGGCGCGGGCGGTGGCGCGGGCGGTTAAGCGGCGGG 357

*msg*56 CGTCAGGCACAGCTTGGCGCGGGCGGTGGCGCGGGCGGTTAAGCGGCAGG 346

*msg*6 CGTCAGGCACAGCTTGGCGCGGGCGGTGGCGCGGGCGGTTAAGCGGAGGG 349

*msg*94 CCTCAAGCACATCTTGGCGCGGGCGGTGGCGCGGGCGGTTAAGCGGCAGG 351

*msg*45 TGTCCTCTAGATTTTGGCGCGGGCGGTGGCGCGGGCGGTTAAGCGGCAGG 339

*msg*41 CGTCAGGCACAGCTTGGCGCGGGCGGTGGCGCGGGCGGTTAAGCGGCAGG 341

*msg*103 TGTCAAGCATGGCTTGGCGCGGGCGGTGGCGCGGGCGGTCAAGCGGCAGG 342

*msg*93 CATC-GGCACGGCTTGGCGCGGGCGGTGGCGCGGGCGGTTAAGCGGCAGA 343

*msg*78 CGTCAGGCACAGCTTGGCGCGGGCGGTGGCGCGGGCGGTTAAGCGGCGGG 343

** * ************************** ****** *

**imperfect inverted repeat**

*msg*32 CA---CAAG---CAC-A-GG----AA-CGCAA---GATGAGGAAGAACTG 371

*msg*24 CT---GCAGCAGCGC-A-AA----AAGCTAGTGTATATGAAGATGAAGAA 387

*msg*84 CTGCACAAGGAGCA----GG----AA-CAAAT---GATGAGGAAGAAGTG 395

*msg*56 TA---AAAGGTGCAC-A-G-----AA-T-AG------CATCGATGAGGAG 378

*msg*6 CT---GCAGGGGCAGGA-GT----AA-A-TGT--ATATGAAGATGAGGAT 387

*msg*94 CA---AAAGGTGCAC-A-G-----AA-T-AGCA------TTGATGAGGAG 383

*msg*45 CA---AAAGGTGCAC-A-G-----AA-T-AGCA------TTGATGAGGAG 371

*msg*41 TA---AAAGGTGCAC-A-G-----AA-T-AGCA------TTGATGAGGAG 373

*msg*103 CA---CAAAA---AA-A-T-----GA-T-GAGA------TTGGAGAGGAA 371

*msg*93 CA---GCAGGGGTAA-A-G-----AA-T-AATG------AAGCTGAAGAA 375

*msg*78 CA---CAAGCGGCTC-AAGTAGTGCA-A-AGTATATATGATGATGATGGA 387

* * * **

*msg*32 CGTCTTT-TACTTTGATTGTAGAA-ATGATTATAACAATGAACCAAAATG 419

*msg*24 ATTCTTTTTGCTTTAATTGCAGGAAAAGATTATAATGATGACAGTAAATG 437

*msg*84 CGTCTCTTGGCTTTGATTGCAAAAGAAAACTATAAAAATACTGATCAATG 445

*msg*56 CATGTTTTAGCTTTAATTCTAAAGGAAAGT---TTGGAAAAAACAGAATG 425

*msg*6 TATCTTTTGGCTTTAATTTTAAAAGAAGGTGCAATGGATGAATCACAATG 437

*msg*94 CATGTTTTAGCTTTAATTCTAAAAGAAAATGATTTGGATGAAACAAAATG 433

*msg*45 CATGTTTTAGCTTTAATTCTAAAAGAAAATGATTTGGATGAAACAAAATG 421

*msg*41 CATGTTTTAGCTTTAATTCTAAAGGAAGATGGACTAGGTGAACAAGAATG 423

*msg*103 CATGTTTTAGCTTTAATTCTAAATAAAAACGATTTAGAAAAAGAAGAATG 421

*msg*93 CGTCTTTTTGCTTTGATTACAAAAGAGGATTATAGCGATACTGGAAAATG 425

*msg*78 ATTCTTTTGGCTTTAATTGCAGGAAAATATTATAAGGATGA---TGAATG 434

* * * **** *** * * ****

*msg*32 CAAAGAAAAGTT-AAAAAACACTGTGAAGAA-TAACCAAAGCAA-ATTAA 466

*msg*24 CAAAAAAAAATTAGAAGAATATTGTCAAGGGCTGGAAAATGCTTCATTAA 487

*msg*84 CAAAGACAAAGTGGAAAAATATTGCAAAACGTTAACAGATGCAGGATTAA 495

*msg*56 TATAAAAAAATTGAAAGAATATTGTGAAAACTTAAAGAAAGTTG-ATGAA 474

*msg*6 CAAAGAAAAACTTAAAGAATACTGTGAAGATATAAAAAGAGTAG-ATGAA 486

*msg*94 TATAAAAAAATTGAAAGAATATTGTCAAGAACTGAAAGAAGCAAAACTGA 483

*msg*45 TATAAAAAAATTGAAAGAATATTGTCAAGAACTGAAAGAAGCAAAACTGA 471

*msg*41 CAAAGAAAAACTAAAAAAATATTGCCAAGAATTAACTGAAGCAAAACTAA 473

*msg*103 CAAAAAAAGACTGAAAGAATACTGCAAAGGCTTAAAAAATATAAATCCTA 471

*msg*93 TAAAAATAAAATAAAAGAATATTGCTATGGGCTGAAAAATGCTTCACTAA 475

*msg*78 CAAAAAAGAATTAGAAAAATATTGTAAGGCATTAACGGATGCAGAATTAA 484

* * * * ** ** * ** * * *

*msg*32 AAATAGAACAAATACACGAAAAATTAAAGGACTTATGTAATAA---TC-A 512

*msg*24 AAAAAAAAGAAATACATGGGAAACTTGAAAGCTTTTGTAAAAA---TGGT 534

*msg*84 ATCCAGAAAAGGTTCACGAAAAATTAAAAGATTTCTGTAATAA---CGGG 542

*msg*56 AATTA--TAGTGTCTATTCAAAATTAAAAGAAATTTGTAAAGA---TA-- 517

*msg*6 AATTT--TAAAGTTTATACAAAACTAAAAGAAATTTGTAAAGA---TA-- 529

*msg*94 CAACGGAAAAAGTTCATGAAAAACTAAAGGATATCTGTGATAA---CACA 530

*msg*45 CAACGGAAAAAGTTCATGAAAAACTAAAGGATATCTGTGATAA---CACA 518

*msg*41 ATATAGAACAAGTACACAAAAAACTTGAAGGTTTTTGCAAAGA---TGGA 520

*msg*103 AATTAGATAAAGTGGATCAAAAATTACAAAAAGTGTGTAAAGAAGATAGA 521

*msg*93 CATCAGAAAAAGTACATAAAGAATTAAAAGATTTTTGCAAAGA---TGGA 522

*msg*78 AACCAGAAAAAGTTCACAAAAAACTTAAGGAGTTTTGCGAAAA---TAAA 531

* * ** * * * ** * *

*msg*32 AAACAAGAGAATAAATGCAAACA-TTAAAAAGCAAAGTAAAA--TGAATG 559

*msg*24 GAAGCAGCAAAAAAATGCACAGAATTAAAGGGCAAAGTTCAGGGAAAATG 584

*msg*84 AAACAAAATAAAAAATGTCAAGATTTACAAACCAAAGTTACGGGGAAATG 592

*msg*56 -ATGGTGCAAAAAAATGTACAGAGTTAAAGAACAATGTTGATACAAAGAA 566

*msg*6 -ACGGTGCAGCAAAATGTACAGGACTGAAAGCTAAGATTGATACGAAGAA 578

*msg*94 AAACGAGATGAAAAATGTAAAAAACTAAAAACTAAAGTTACTGAAAAGTG 580

*msg*45 AAACGAGATGAAAAATGTAAAAAACTAAAAACTAAAGTTACTGAAAAGTG 568

*msg*41 AAAGCAGATGAAAAATGCAAAGAACTGAAAGCCAATATTGAGAAAAAATG 570

*msg*103 ACAGCTGAAGAAAAGTGCACAGGATTAAAAGACAAAGTCACTCAAAAATG 571

*msg*93 AATGAAGAAAAAAAATGCGGAGAACTAAAAACCAAAATTGAAGAAAAATG 572

*msg*78 AAAGCAGATTCAAAATGCAAAGAATTGAAAGAAAAACTCACTCAAAAATG 581

** ** * * * ** * *

*msg*32 CGACACATTTAAAACA--G--CGTAGGACTCTA-GTAGCAAAG-----TC 599

*msg*24 CACTACATTTAAAACAAAA--CTTCAAACAGCAGCTGGAAAAGGAATTTC 632

*msg*84 CACATCTTTTCAACAAAAA--CTAAAAACTGCT-TTAACAAAT------- 632

*msg*56 AACTGCTTTCAAAGCAAAA--CTTGATGATGCATCTAAAAAAGAAATTTC 614

*msg*6 AATTGCTTTCAAAACAAAA--CTTGATGAAGCATCAGCAAAACAAATTTC 626

*msg*94 CAATGAATTTAAAACAAAAAAGTTAGAA---CAAGCGTTAAAGAAA---- 623

*msg*45 CAATGAATTTAAAACAAAAAAGTTAGAA---CAAGCGTTAAAGAAA---- 611

*msg*41 TACTACAATCAAAGGAAAA--CTTAAAGAAGCAATTAAAAAAAAAAATTC 618

*msg*103 CAATGATTTTAAAAATAAA--CTTCAAATAGCAGCTGTAAAAGAAATTTC 619

*msg*93 TAAAACATTCAAAGGAAAC--CTCGAAACAGCTGTTAAGAAAAACATTAA 620

*msg*78 TACTGCAATCAAAGGAAAA--CTTACAGAAGCAATTAAAAAAAAAAAATC 629

* ** ***

*msg*32 A-ATTC--AAGATGATGAATGTACGAAAAATGAACGACAATGCCTATTTT 646

*msg*24 AGATTTAACGGATGACGAATGTAAAGAGAATGAACAACAATGCCTATTTT 682

*msg*84 ----CCATCAGATGATAATTGTAAAGAAAATGAACAACAATGCTTATTTT 678

*msg*56 ACAATTAACAGAAACGGATTGTT---CGAATCAAAAAGAATGTTTATTTT 661

*msg*6 GCAATTGAAAGATATAGACTGTT---TAAATCAGAAAGAATGTTTATTTT 673

*msg*94 ----CTATCTGATAATGATTGCAAAGAAAATGAGCGGCAATGCCTATTTT 669

*msg*45 ----CTATCTGATAATGATTGCAAAGAAAATGAGCGGCAATGCCTATTTT 657

*msg*41 AGATTTAACGGATAAGGATTGCAAAGAGAATGAACAACAATGCCTATTTT 668

*msg*103 AAAACTGACAGATGGCGATTGTAAAGAGAATGAACGACAATGCCTATTTT 669

*msg*93 AAATTTGGAAGATACAGATTGTG---CAAATGAACAACAATGTCTATTTT 667

*msg*78 AGATTTAACGGATGAGGATTGCAAAAAAAATGAACAACAATGCCTATTTT 679

** * ** *** * **** ******

*msg*32 TGGAGGGAGCGTGTCCAACAGATCTTAAAGAAGATTGCAATAC--T-AGA 693

*msg*24 TGGAGGAAGCATGCTCT---GATCTTACAGAGAATTGCAATAAATTAAGG 729

*msg*84 TGGAGGGAGCGTGTCCAAAAGAGCTTAAAGACGATTGCAATACTTTGAGA 728

*msg*56 TAGAAGAGGCGTACTCTAATGATCTTAAAGAAAACTGTAACACATTGAGA 711

*msg*6 TAGAGGAAGCGTACTCTAATGAACTTAAAGAAAAGTGTAATACATTAAGA 723

*msg*94 TGGAGGGGGCATGCCCTAGTGTTCTTATAGAGGATTGCAACAAACTAAGG 719

*msg*45 TGGAGGGGGCATGCCCTAGTGTTCTTATAGAGGATTGCAACAAACTAAGG 707

*msg*41 TGGAGGGAGTATGTTCAAAAGAACTTAAAGATGATTGCAATACTTTGAGA 718

*msg*103 TGGAGGGAGCATGTCCTAGTGATCTTACAGAGAATTGTAGCAAACTAAGG 719

*msg*93 TTGAGGGAGCATGTCCTAGTGATCTTACAGAGAATTGCAACAAACTAAGG 717

*msg*78 TGGAGGGAGCATGCCCTA---ATCTTGTAGAGGATTGTAACAAACTAAGA 726

* ** * * * * *** *** * ** * * * **

*msg*32 AATAAATGTTATAAGAAGAAGCGTGAGGAAGTGGCAAATGAAGT---TTT 740

*msg*24 AATAACTGTTATCAAAAGAAACGAAATGAAGTAGCAGAAAAAGTTCTTTT 779

*msg*84 AATAAGTGCTATCAAAAGAAGCGTGATAAAGTGGCGGAAGAAGCTCTTTT 778

*msg*56 ACTAATTGTTATCAAAAGAAGCGTGATGAAGTGGCAGAAAAAGCTCTTTT 761

*msg*6 ACTAATTGTTATCAAAAGAAGCGAGACGAAGTGGCAGAAAAAGCTCTTTT 773

*msg*94 AATCTATGTTACCAGAAAAAACGTGATAGAGTAGCAGAAGAAGTCCTTTT 769

*msg*45 AATCTATGTTACCAGAAAAAACGTGATAGAGTAGCAGAAGAAGTCCTTTT 757

*msg*41 AATAAGTGCTATCAAAAGAAGCGTGATAAAGTTGCGGAAGAAGTTCTTTT 768

*msg*103 AATCTATGTTACCAGAGAAAACGTGACAGAGTAGCAGAAGATGTCCTTTT 769

*msg*93 AATCTATGTTACCAGAGAAAACGTGACGGAGTAGCAGAAGAAGTCCTTTT 767

*msg*78 AATCTATGTTATCAGAGAAAACGTGACGGAGTAGCAGAAGAAATCCTTTT 776

* * ** ** * * ** ** * *** ** * * ***

*msg*32 TAGAGCACTTAGTGATGATCTTAAAGATACAAACACATGTAAAGGAAAAA 790

*msg*24 AAGGGCACTCAGTGATGATCTTAAAGATGCAAAGACATGTAAAGAAAAAA 829

*msg*84 AAGAGCGCTCAGTAATGATCTTGAAAATGAGACTAAATGTAAAGAGAAAG 828

*msg*56 AAGGGCACTTAAAGGAAGTCTTGAAGATAAAAATAAGTGTAAAAAGAAAC 811

*msg*6 AAGGGCACTTAAAGGAAGTCTTGAAAATGATGATAAATGCAAAGAGAAAC 823

*msg*94 GAGGGCGCTTCGCGGAAATCTAACTAATGAAACTACATGTCAAGAAAATC 819

*msg*45 GAGGGCGCTTCGCGGAAATCTAACTAATGAAACTACATGTCAAGAAAATC 807

*msg*41 AAGAGCACTTCGTAGTGATCTTAATGGATCAGTCATATGTGAAAAAAAAC 818

*msg*103 GAGGGCACTTCGTGGGGATTTGGGGGACAATTCTGAATGCAAAAAGAAAA 819

*msg*93 GAGGGCACTTCGTGATGATCTTGGAAATGATACCGCTTGTAAAAAGAAGA 817

*msg*78 GAGGGCACTTCGTGGAGATTTGGAGGAAGAAGCTGGATGCAAAAAGAAAA 826

** ** ** * * ** ** **

*msg*32 TTAAGGATATTTGTCTTGCATTAGGCCAAGAAAGTGATGAGTTAATGCAA 840

*msg*24 TTAAGGATGTTTGTCGTACATTGGGTCAAGAAAGTAACGAGTTGATGCAA 879

*msg*84 TTAAAAATGTTTGTTTTGCATTAGCTGAAGAAAGTGATGAGTTAATGCAA 878

*msg*56 TTGAGGAAATTTGTCCTCTGTTGGGTCAAGAAAGTAATGAGTTAATACAA 861

*msg*6 TCAAAGTGGTTTGTCTTGAGTTGGGCCAAGAAAGTAATGAGTTGATGCAA 873

*msg*94 TCAAAGAGGTTTGCCCAATCTTGGGGAGGGAAAGTGACGAGTTAACAAGC 869

*msg*45 TCAAAGAGGTTTGCCCAATCTTGGGGAGGGAAAGTGACGAGTTAACAAGC 857

*msg*41 TTAAAGAGATTTGCCCTGTCTTGGG-AGGGAAAGTGATGAGTA--CAAAC 865

*msg*103 TAAAGGATGTTTGCCCAAAAATAGGCCAAGAAAGTGATGAGTTGACAATG 869

*msg*93 TTGAAAATGTTTGCCTAAAAATAGGCCAAGAAAGTGATGAGTTGACAATG 867

*msg*78 TCAAGAACGTTTGCTCAAAAATAGGCCAAGAAAGTGATGAGTTGACAATG 876

* * **** * * ****** * ****

*msg*32 AAGTGTTTCAATACAGAGTCTCTATGTACATCTCTAGTAGCATTAGCAAA 890

*msg*24 AGATGTTTCGATACAGACTCTTTATGCACATCTTTAGTAAAAACAGCAGA 929

*msg*84 AAGTGTTTCAATACAGACTCTCTATGCACATCTCTAGTAGCATTAGCAAG 928

*msg*56 AAGTGTTTAAATCTAGACTCTACATGTCCACCTCTAGTGCAAGCAGCAAA 911

*msg*6 AGGTGTTTTAATACGGATTCTACATGCACAACTTTAGTAAAAGCAGCAGA 923

*msg*94 TTGTGTCTGAACCAGGAAAATACGTGCAAAAATATTATAAAGAAAAGAGA 919

*msg*45 TTGTGTCTGAACCAGGAAAATACGTGCAAAAATATTATAAAGAAAAGAGA 907

*msg*41 TTGTGTCTGAACCAGAAAGAGACATGTAAGAATATTTTAATTGAAAAAGA 915

*msg*103 TTATGTCTTGATCAAGAGAAAACATGCGTAAGTCTTGTAGCAAAAGGAAA 919

*msg*93 TTATGTCTTGATCAACAAGCAACATGCGTAAGTCTTGTAGCAAAAGGAAA 917

*msg*78 TTATGTTTTGATCAGAAGAAAACATGCAAAAAATTTGTGTCAGAAAGAGA 926

*** * * * ** * * * *

*msg*32 AGAAAAGTGTACTCCTCTGAAAACAGAGATAGAGAAAGTGCTAAATCCTG 940

*msg*24 AGAAAAGTGTAAATTTTTGAAGAAAGAAATAGAGAATGTATTAAAACCTA 979

*msg*84 AGAAAAGTGTGAATCTCTGAAGACAATGATAAAAAATGTGCTAACTATTG 978

*msg*56 AAATAAGTGTGA--CTT-TAAGACAGAGATAGAGAAAGTAATAAGTAATA 958

*msg*6 AGAAAAGTGTACTCCTCTGAAAACAGAGATAGAGAACGTACTAAATCCTG 973

*msg*94 TAATAAATGCAATACTCTTAAAACAAGTGTTGCAGCAGCACTTG------ 963

*msg*45 TAATAAATGCAATACTCTTAAAACAAGTGTTGCAGCAGCACTTG------ 951

*msg*41 TAAGAAATGCGGTACTCTTAAAACAGATGTTTCAGCAGCACTTG------ 959

*msg*103 AAGTAAGTGTAGTGCTCTAAAACAAGAAGTCGAAGAAGCACTTAAGAAGA 969

*msg*93 AAGTAAGTGTAGTGTTTTGAAACAAAAAGTCGAAGAAGCACTTAAGGAGA 967

*msg*78 AGGAAAATGTGATGCTCTTGAAAAAATTGTTAAAGAAGCACTTAAAAAGA 976

** ** * * * * * *

*msg*32 GTGGGAAGTTG-AA---AAAAGAAGGGTATTCTTTACTTGAAAAATGCTA 986

*msg*24 ATGGGGAGTTG-CA---AAAAAAAGGGCATTCTTTGCTTGAGAAATGCTA 1025

*msg*84 ATGGGGAGTTG-CA---AAAGAAAGGGCATTCTTTACTTGAGAAATGCTA 1024

*msg*56 AAGAAGAGTTA-CA---AAAAAGAGGGCGTTTTTTACTTGAAGAATGCCA 1004

*msg*6 GTGGGGAGTTA-AA---AAAAGAAGGACACTTTTTACTTGAGAAATGCTA 1019

*msg*94 ----GAAGCTTTAAAAAAGAAGAATGCCTATCATTACTTGAAGAATGTTA 1009

*msg*45 ----GAAGCTTTAAAAAAGAAGAATGTTTATCATTACTTGAAGAATGTTA 997

*msg*41 ----GAAGTTTTAAAAAAGAAACATGTCTTGAATTGCTTGAACAATGCTA 1005

*msg*103 AGAATGAATTA-CG---AGGAAAATGTCTACCATTACTTGAGCAATGTTA 1015

*msg*93 AAAATGAACTA-CG---AGGAAAATGTCTACCATTACTTGAGCAATGTTA 1013

*msg*78 ATAGTGAATTA-CG---AGGAAAATGTCTATCATTACTTGAGCAATGTTA 1022

* * * * * ** ***** **** *

*msg*32 CTTTTATGGACCAAGTTGTAAAGAT---A------------AT------- 1014

*msg*24 CTTTTACGAAGAAAATTGCAAAGACCCAA------------AT------- 1056

*msg*84 CTTTTATGGACCAAGCTGTAAAGGT---A------------GT------- 1052

*msg*56 CTTTTACGGACCAAATTGTAAAAAT---A------------AA------- 1032

*msg*6 CTTTTATGGGCCAAATTGTAAAGGT---A--------------------- 1045

*msg*94 TTTTTACGTTGGAAACTGCCAAGAAGACG------------AT------- 1040

*msg*45 TTTTTACGTTGGAAACTGCAAAGAATAC---------------------- 1025

*msg*41 TTTTTACATTGGAAATTGCGGAGACGACG------------AT------- 1036

*msg*103 CTTTCACAGAGGGAACTGTAAAAAAGATGCATCACAATGCAA---ACCTC 1062

*msg*93 CTTTCACAGAGGGAACTGCAAAAAAGATGCATCACAATGCAA---ACCTC 1060

*msg*78 CTTTCACAGAGGGAACTGTGAAGGAGACAAATCAAAGTGCAATAAACTTA 1072

*** * * ** *

*msg*32 -----------------------------AAATGTAGTGATTTGAAGGAA 1035

*msg*24 -----------------------AAACCGAAATGTGAAAAACTCGTAAAT 1083

*msg*84 -----------------------------AAATGTGGTGATTTGAAGGAA 1073

*msg*56 -----------------------GAATCAAAATGTAATAATTTGCAAAAA 1059

*msg*6 --------------------------GT-AGATGTGGTGATTTGAAGGAA 1068

*msg*94 -----------------------ATAATTGAATGTATTAAATTGGGAGAG 1067

*msg*45 -----------------------ATATCAGAATGTGATACATTGGCAGAG 1052

*msg*41 -----------------------ATAATTAAATGTATTGAATTGGGAGGG 1063

*msg*103 AAAATAAAGACTGCGAGGAATATCTACCAAAGTGTGATGAATTAGCAGAA 1112

*msg*93 AAAATAAAGACTGCGAGGACTATCTACCAAAATGTGATGAATTAGCAAAA 1110

*msg*78 CTAATCAGGACTGTAAAGAATATATACCAGATTGCAATAAACTAGAAGAA 1122

** *

*msg*32 AAATGTAAGGAAGAAAAAATCATTTATATACCACCAGGTTCGGATTTTGA 1085

*msg*24 GAAGCTAAAAAAAAAAAGATTGTCTACAAAGGACCAAGTTCAGATTTTGA 1133

*msg*84 AAATGCAAGAAAGAAGAAATCATTTATCTACCTCCAGACTCTGATTTTGA 1123

*msg*56 AAATGCAAAGAAGAAGAAATCTTTTATGTACCACCAGGTTCAGATTTTAA 1109

*msg*6 AAATGTAAGAAAGAAGGAATCATTTATATACCACCAGGTTCAGATTTTGA 1118

*msg*94 AAATGCCAGGAACAAAACATTGTTTATATACCACCAGGACCCGATTTTGA 1117

*msg*45 AAATGTGAAAAAGAAAATATTGTTTATATGCATCCGGGACCCGATTTTGA 1102

*msg*41 AAATGCCAAGAACAAAACATTGTTTATATACCACCAGGACCCGATTTTGA 1113

*msg*103 GAATGTGAAGAAAGAAGCGTCATTTATATACATCCAGGACCCGATTTCGA 1162

*msg*93 GAATGTGGAAAAAAGGGCGTCATTTATATACATCCAGGGCCCGATTTCGA 1160

*msg*78 GAATGTGAAAAACAAAATATCATTTACACACATCCGGGACCCGATTTTGA 1172

** ** * * ** ** * ***** *

*msg*32 TCCTACAAAACCAAAAACTACAGTTGCGGAAAAAATAGGTTTGGAAGAAC 1135

*msg*24 TCCTACAAGACCAGAGGCCACGCTAGCAGAAAAAATAGGTCTGAAAGAGC 1183

*msg*84 TCCTACAAGGCCAGAGGCTACGCTAGCAGAAAAAATAGGTCTAGAAAAGC 1173

*msg*56 TCCCACAAAACCAGAACCTACGCTGGCAGAAAAAATAGGTTTGGAAGAGC 1159

*msg*6 TCCTACAAGACCAGAGGCTACGCTGGCAGAAAAAATAGGTTTGGAAGAAC 1168

*msg*94 TCCAACTAGGCCAGAGGCTACACTAGCAGAAGACATAGGGCTGGAAGAGC 1167

*msg*45 TCCAACTAATCCAGAGCCTACAATAGCAGAGGACATAGGGCTGGAAGAAT 1152

*msg*41 TCCAACTAGGCCAGAGGCTACAATAGCAGAGGATATAGGGCTGGAAGAGC 1163

*msg*103 TCCAACTAAGCCGGAGCCTACAGTAGCAGAGGACATAGGGCTGGAAGAGC 1212

*msg*93 TCCAACTAAGCCAGAGCCTACAGTAGCAGAGGACATAGGGCTAGAAGAGC 1210

*msg*78 TCCAACTAAGCCAGAGCCTACACTAGCAGAGGATATAGGGCTGGAGGAGC 1222

*** ** * ** * * ** * ** ** * ***** * * *

*msg*32 TTTATAAGAAAGCAGCAGCACAAGGAGTTCTGATTGGAAGAATACTAAAA 1185

*msg*24 TTTATGAGGAAGCAGCAACACAAGGAGTTTTGATTGGAAGAGCACTAAAG 1233

*msg*84 TTTATAAGGAAACAGTAACACAAGGAGTTGTAATTGAAAGAGCACCAGAA 1223

*msg*56 TTTACAAAGAAGCGGCGACGCAGGGAATTCTATTTGGAAAACCACCAGAA 1209

*msg*6 TTTATGAGGAAGCGGCGGCACAAGGAGTTTTGATTGGGAAAGCACTAGAA 1218

*msg*94 TTTATAAAGAGGCTGAGAAGGATGGAATTTTTATTGGAAAGAATCATCTA 1217

*msg*45 TTTATAAGAAGGCAGAGGAGGATGGAGTTTTTATTGGAAGACAACAAGTA 1202

*msg*41 TTTATAAAGAGGCTGAGAAGGATGGAATTTTTATTGGAAAGAATCATCTA 1213

*msg*103 TTTATAGGAGGGCAGAGGAGGATGGAGTTTTTATTGGAAAGAATCATCTA 1262

*msg*93 TTTATAAGAGGGCAGAAGAGGATGGAGTTTTTATTGGAAAACAACATCTG 1260

*msg*78 TTTATAAAGAGGCTGAGAAGGATGGAGTTCATATTGGAAGGCCTCCTATA 1272

**** * * * *** ** *** * *

*msg*32 GAAGATATTGTTGACATATTAGTGTTTTTATCAGAAAACG---ATGATTT 1232

*msg*24 GAAGATATTGTTGACATATTAGTGTTTTTATCAGAAGACA---ATTT--- 1277

*msg*84 AGAGATATTGTTGATCTATTAGTGTTTCTGTCAGAGAGCA---AGCCTTT 1270

*msg*56 AGGGATGTTGTTGATTTATTAGTGTTTCTGTCAAAAAGCA---ATCTTTT 1256

*msg*6 GGAGATGTTCTTGATTTATTAGTGTTTTTGTCAGAGAAAA---ATTCTTT 1265

*msg*94 AGAGATGCAACGGCTTTGTTGGCGTTGTTGGTTGGAAAAGATAATACCGG 1267

*msg*45 AGAGATGCGACAGCTTTGTTGGCGTTGTTGGTTAAAAAAGATAATACCGG 1252

*msg*41 AGAGATGCGACAGCTTTGTTGGTGTTGTTAATTAAAGATTCTAATTCTAA 1263

*msg*103 AGAGATGCGACAGCTTTGTTAACTTTGTTGACTGGAAAAG---ATAGTGA 1309

*msg*93 AGAGATGCAACAGCTTTGTTGGCATTATTTGTTGAAAAAA---ATGCAAA 1307

*msg*78 AGAGATGCAACTGCTCTGCTGGCACTTTTGATTCAGAA-TCCAATTCTTA 1321

*** * * * * * *

*msg*32 ---T------------AAAAAAGATAAATGCGTAGCAGTACTCACAGATA 1267

*msg*24 ---T------------GATAAAGATAAATGTAAGAAAGTACTCACTGATA 1312

*msg*84 ---T------------AATGAAGCTCAATGCAAGGAAGTACTCACTAATA 1305

*msg*56 ---T------------AATGAAAATAAATGCAAGGATGTACTCAATACTA 1291

*msg*6 ---T------------GATGAAACTCAATGCCAGAGTGTACTCAGTAACA 1300

*msg*94 AAGAA---A--TA-ATGATGGAGAAAAATGCAATAAGATTCTCGAAGATA 1311

*msg*45 AAATA---A--TG-ATATCGGAAAAAAATGTAATGAGATTCTCGAAAATA 1296

*msg*41 ---AA---A--AG-ACGACAAAGAGAAATGCAAAGAAGCCCTTCAAAAAA 1304

*msg*103 ---T------------ATGGAAAAAAAATGCACTAAAGTTCTTGGTGAAA 1344

*msg*93 ---T------------GTTGAAACAAAATGTAATCAAGTCCTTGCAGATA 1342

*msg*78 --ATACTCAACTGAGCGAAAAAGAAAAATGTGGAAAAGTCCTTAAAGATA 1369

* **** ** *

*msg*32 AATGTAATT---CTATTAAATATTTGGCAGAAAGTATAAAAGAATTATGT 1314

*msg*24 AATGTGATT---CTATTAAATATTTGGCAAAAAGTATAAAGGAATTATGT 1359

*msg*84 AATGTGATT---CTATTCAATATCTAGCAAAAGATTTAAAAGATTTATGT 1352

*msg*56 AATGTGACT---CCTTTAAGCATTTAGCAAAAGAATTAAAAGATTTATGT 1338

*msg*6 AATGTAAAT---TTATTGGATATCTAACAGATGATTTAAAAGAATTATGT 1347

*msg*94 AGTGCAAAAACTCACATGAACATG------AGGCCTTGGAAAAACTATGT 1355

*msg*45 AATGCAAAAACTCTCATGAACATG------AGGCCTTGGAAAAACTATGT 1340

*msg*41 GCTGCAAAAATCCTCATGAACATG------AGGCTTTAGAAAGTTTATGT 1348

*msg*103 AGTGCAAAAATTCTCAACAGCATG------AAGCTCTAGAAGATTTATGT 1388

*msg*93 AATGCAAAAACTCTCATGAACATG------AGGCCTTGGAAAAACTATGT 1386

*msg*78 AGTGTAAAGAGTTAAAAGAACATG------AGGTTTTGGGATATCTATGT 1413

** * ** * * *****

*msg*32 AAAAATA---------CAGGTAATT---AT---ACAGATAAATGTAAAGA 1349

*msg*24 GAAAATA---------CAAGCAGTC---AT---GAAAATCAATGTGAAGA 1394

*msg*84 AAAGATA---------AAAATAATT---AT---ACAGATAAATGTAAAGA 1387

*msg*56 GAAAATA---------AAAACAATC---AT---ACAGATAAATGTAAAGA 1373

*msg*6 GGAAATA---------AAACCATTC---AT---AAAGATAAATGTAAAGA 1382

*msg*94 AATAAAACTAA---TCCAAGTGATT---ATAAA-AAAAGAAATGCGAAGA 1398

*msg*45 AATAAAACTAA---TCCAAGTGATT---ATAAAAAAAAGAAATGCGAAGA 1384

*msg*41 AAGAAAAATGG---TTTAAGTAATG---ATGGAACGAAAAAATGTGAAGA 1392

*msg*103 AATAATAAAGGTATTCCTAATACAA---ACGGAACAGAAAAATGTAAAGA 1435

*msg*93 ACGAAAAATGC---TGTAAATAACA---TTGGAAAGGAAAAGTGCAAAGA 1430

*msg*78 GATGACAGTGA---CTCGACTAGTCAAAATGGAACTGAAAAATGTAACGT 1460

* * * * ** * *

*msg*32 ATTTGAAGAGGAATTTAAAAAA-AAAAGAA-TA--CATACTGCAAAACTT 1395

*msg*24 ATTTAAAAAAGAATTTGGCAGAAAAAAAGA-CGCTCTAACTAAAAAGCTT 1443

*msg*84 ATTTAAAAATGAGTTTGAAGAAATAAAGCT-CGCTTTGACTGCAAAATTT 1436

*msg*56 GTTTGAAAAAGAATTTAAAGGAATAAAGGC-CTCTTTGACTGCAAAATTT 1422

*msg*6 ATTTAAGAATGAATTTGACAAAAAAAAGAA-CGCTTTAAATACAAAGTTT 1431

*msg*94 ATGA--GAAAGATATCAGAAAAACATGTGA-TATTCTCACGTCAAAGCTT 1445

*msg*45 ATTAGAGAAAGATATCAGAAAAACATGTGA-TATTCTCACGTCAAAGCTT 1433

*msg*41 ATTGCAAAATGATATTAACAAAACTTGCAA-AATTTTCACTTCAAAAGTC 1441

*msg*103 ATTAA---AAGATGTTAAAGAAAGGTGCAA-AATTCTCACTTTAAAAGTT 1481

*msg*93 ATTGGAGGAAGATATTAAAAAAACATGTAA-TATTTTCGCGTCAAAGCTT 1479

*msg*78 ATTAGGAGAGGA-ATTAGCAAAACGTAGCTTAATTGTTTCTGAAAAAATA 1509

* * ** * * *** *

*msg*32 GGAAACAGTCA---ATTTGAA----------------GATGAGATTGCAT 1426

*msg*24 GAAAGCAAACA---ATTTGAA----------------AATAAAATTGAAT 1474

*msg*84 AAAATCAGACA---ATTTAAA----------------GATGAGATTGCAT 1467

*msg*56 AAAA--AA-------TTTGGAA----------------ACGAAATTGAAT 1447

*msg*6 GAAA-GAAACT-C-ATTTAAAAGC-------------AATGAAATTAAAT 1465

*msg*94 ACAAATAATCATCTTTTTGA-CTCAAAAAAAGGAAGTAATGGAATTATTG 1494

*msg*45 ACAAATAATCATCTTTTTGA-CTCAAAAAAAGGAAGTAATGGAATTATTG 1482

*msg*41 ACTAATAATCGTCTTTTTGA-CCCAACAAAAGGAAATAATGAAATTGTTG 1490

*msg*103 ATTAATAATCGTTTTTTTGA-CCCAACAAAAGGAAATAATGAAATTGTTA 1530

*msg*93 ATAAATAATCGTCTTTTTGA-TCCAAAAAAAGGAAATAATGGAATTATTG 1528

*msg*78 AAGAACAAACACCTCTCTGGATCCG------G-------GAAAACCATTC 1546

* * * * *

*msg*32 TATGGGATGGATTACCAACTTTCCTTACTGAAAATGAGTGCACAAGATTA 1476

*msg*24 TATGGAGCGAATTACCAAGATTCCTTACTGAAAATGACTGCACGAGATTC 1524

*msg*84 TATGGGATGAATTACCAAGTCTCCTTACTGAAAATGACTGCACAAGATTA 1517

*msg*56 TATGGAACGAATTGCCGAACTTTCTTACTGAAGATGAATGTGCAGAATTA 1497

*msg*6 TATGGAGTGAATTGCCGAATTTCCTTAATGAAGATGAATGTGTAGAACTA 1515

*msg*94 GATGGGGAGGATTACCAACATTTCTTAGCAACGAAGATTGTGCAAAATTA 1544

*msg*45 GATGGGGAGGATTACCAACATTTCTTAGCAACGAAGATTGTGCAAAATTA 1532

*msg*41 GATGGGAAGGGTTGCCAACATTTCTTAGCAACGAAGAATGTGCGAAATTA 1540

*msg*103 AATGGGGCAAGTTGCCAACATTTCTTAGCAAAGAAGAGTGTACAAGACTA 1580

*msg*93 GATGGGGAGGATTGCCAACATTCCTTAGCAACGAAGATTGTACAAAATTA 1578

*msg*78 CATGGTATAAGTTATCGACATTTCTTAGTGACAGTGACTGTGCAAGATTA 1596

**** ** * * * **** * ** ** * *

*msg*32 CAGTCAGATTGTTTTTATTTTGG---AGGTCAAACAT----CTCTTAAAA 1519

*msg*24 CAATCAGATTGCTTTTATTTTAA---AAGCCAAACAT----CTCTTGACA 1567

*msg*84 CAATCAGACTGTTTTTATTTTGA---AAATCAACAGT----CTTTCGAGA 1560

*msg*56 GAATCAGACTGTTTTTATTTCGA---AAAACA---GT----CTTTCGAGA 1537

*msg*6 GAATCAGACTGTTTTTATTTCGA---AAATCA---GT----CTTTCGAGA 1555

*msg*94 GAGTCCTATTGTTTCTATTTTGA---AAAAAAA-TGCCAAGATGGCGAAA 1590

*msg*45 GAGTCCTATTGTTTCTATTTTGA---AAAAAAA-TGCCAAGATGGCGAAA 1578

*msg*41 GAGTCCTATTGTTTCTATTTTGA---AAAAAAA-TGTCCAGATGGAGAAA 1586

*msg*103 GAATCTTATTGCTTTTATTTTAA---AGAAAGC-TGTCCAGATGCTGAAA 1626

*msg*93 GAGTCCTATTGTTTCTATTTTGA---AAAAAAA-TGTCAAGATGGCGAAA 1624

*msg*78 GAGTCAGACTGTTTTTATTTTGCTCAAAATAAA-GATC---CTCTTGAAA 1642

* ** * ** ** ***** * * * *

*msg*32 AATGGTGTAAGAATGTTAAGGCAGCGTGTTATAAAAGAGGTCTTGATGCA 1569

*msg*24 AATGGTGCAAAAGTGTTAAGGCAGCGTGTTATAAAAGGGGTCTTGATGCA 1617

*msg*84 AACAATGTAAGAATGTTAAGGCAGCGTGCTATAAAAGAGGGCTCTATGCA 1610

*msg*56 AGCAATGTAAGAATGTTAAGGCAGCATGCTACAAAAGAGGACTTTATAAG 1587

*msg*6 AACAATGTAAGAATGTTAAGGCAGCGTGCTACAAAAGAGGACTCTATGCA 1605

*msg*94 AATCATGTGCGAACGTAAGGGCAGCGTGTTATAAGAGAGGGCTTGATGCA 1640

*msg*45 AATCATGTGCGAACGTAAGGGCAGCGTGTTATAAGAGAGGGCTTGATGCA 1628

*msg*41 ATGCATGTAAAAATATAAGAGCAACATGTTACAAAAGAGGACTTGATGCA 1636

*msg*103 AAGCATGTATGAATGTGAAAGCGGCATGTTATAAAAGAGGACTTGATGCA 1676

*msg*93 AATCATGTGCGAATGTAAGGGCAGCGTGTTATAAGAGAGGGCTTGATGCA 1674

*msg*78 AAGAATGCAAGAATGTTAAAGCAGCATGTTATAAAAAAGGGCTTGAAGCA 1692

* ** * * * ** * ** ** ** * ** ** *

*msg*32 TTAGCAAATCAAGCATTACAAGACAAAATGAGAGGAAAGTTTCATGACAG 1619

*msg*24 TTAGCAAATCAAGTATTACAAGACAGAATGAGAGGGAAGTTTCATGACAG 1667

*msg*84 TTAGCAAACCAAGCATTACAGGACAAGCTGCGAGGAAAGTTTTATGATAC 1660

*msg*56 TTAGCAAACCAAGCATTTCAGGACAAGCTTCGAGGAAAGTTTCATGATAC 1637

*msg*6 TTAGCAAACCAAGTATTACAGGACAAGCTTCGAGGGAAATTTCATGATAC 1655

*msg*94 CGGGCAAACAATATATTGCAAAAAAATATGCGAGGGTTATTGCATGGCTC 1690

*msg*45 CGGGCAAACAATATATTGCAAAAAAATATGCGAGGGTTATTGCATGGCTC 1678

*msg*41 CGAGCAAATAAAGTGCTGCAAGAAAATATGCGAGGAATGTTACATGGTTC 1686

*msg*103 CGGGCAAATAAAGTGCTGCAAGAAAATATGCGTGGGTTATTACGTGGTTC 1726

*msg*93 CGGGCAAATAAAGTGCTGCAAGAAAATATGCGAGGAATGTTACATGGTTC 1724

*msg*78 CTAGCCAATGAAGCATTTCAAAGCAAGATGTACGGATTGTTCCGTGGTTC 1742

** ** * * ** * * ** ** **

*msg*32 AAATGACCCATTGTTTAAAAAGCTTCAAAAAGAGTTAGTAAAGGAGTGTG 1669

*msg*24 AAACGATAAATGGTCTGAGATGCTTCAAAAAGAGCTGGTAAAAGTATGTA 1717

*msg*84 AAATGACCCATTGTTTAGAAAGCTTCAAAAAGAGTTAGTAAAGGAATGTG 1710

*msg*56 AAATGACCCATTCTTTGAAAAGCTTCAAAAAGAATTAGTAAAGGTGTGTC 1687

*msg*6 AAATGATACATCGTTTAAAAAGCTTCAAAAAAAGTTAGTAAAAGTGTGTG 1705

*msg*94 AAATAAAGATTGGCTTAAGAAATTTCAACAAGAATTAGTAAAAGTATGTG 1740

*msg*45 AAATAAAGATTGGCTTAAGAAATTTCAACAAGAATTAGTAAAAGTATGTG 1728

*msg*41 AAACAAAAGCTGGCTTGAAAAATTTCAACAAGAATTAGTAAAAGTATGTG 1736

*msg*103 AAATCAAAGTTGGCTTAAGGAGTTTCAACAAAGATTAGTAAAAGTATGTA 1776

*msg*93 AAACAAAAGCTGGCTTGAAAAGTTTCAACAAGAATTAGTAAAAGTATGCA 1774

*msg*78 AGGCGAAAAATGGTTTAAGGGACTATTGGACAAAATAATGGAAGAATGTT 1792

* * * * * * * * * * **

*msg*32 CGAATCTGAAA---GGAAA---------------AAGTGACGAGTTATTT 1701

*msg*24 TGGATCTGAAA---GAAAA---------------GAGTGATGAGTTATTT 1749

*msg*84 CGAATCTGAAA---GGAGA---------------GAGTGACGAGTTATTT 1742

*msg*56 TGGGTCTGAAA---AAAAA---------------GAGCAATGAGTTATTT 1719

*msg*6 TGGACCTGAAA---AAAAA---------------GAGCAATGAGTTATTT 1737

*msg*94 AGAAACTGAAA---GGAAATAAAGGAAGTTTCTCAAACGATGAATTGTTT 1787

*msg*45 AGAAACTGAAA---GGAAATAAAGGAAGTTTCTCAAACGATGAATTGTTT 1775

*msg*41 AGAAACTGAAAAAAGAAAACAAAGGAAGTTTCTC-AACGATGAATTATTT 1785

*msg*103 AGGAGCTAAAA---GAAAATAAAGGAAGTTTCCCAAACGATGAAATATTT 1823

*msg*93 AGGAACTAAAA---GAAAATAAAGAAAATTTCCCAAACGATGAACTATTT 1821

*msg*78 CGGGACTTAAA---ACAAC------A---------AGCGATGAGTTGTTT 1824

* ** *** * * * ** * ***

*msg*32 GTGTTGTGTGTGCAACCAACAAATGCAGTTTTTATATTGTTAGCGGATTT 1751

*msg*24 GCATTGTGTGTGCAACCAACAGAAGCAATTTTTACATTATTAGATGATTT 1799

*msg*84 GTATTGTGTGTACAACCAACAGAAGCAATTTTTATATTATTAAACGATTT 1792

*msg*56 GTGTTTTGTGTCCAGCCAGATAGTGCTGTTTCTATACTTTTAGGGGATTT 1769

*msg*6 GTGTTTTGTGTCCAGCCAAATAACGCTGTTTCTATACTTTTAAGAGATTT 1787

*msg*94 GTTCTGTGTATACAACCAGCAAAGGCAGCACGATTACTTACACATGACCA 1837

*msg*45 GTTCTGTGTATACAACCAACAAAGGCAGCACGATTACTTACACATGACCA 1825

*msg*41 GTTCTGTGTGTACAGCCAGCAAAAGCAGCCCGGTTGCTTACACATGATCT 1835

*msg*103 GTTCTGTGTGTACAGCCAGCAAAAGCTGCACGATTACTTACACACGATCA 1873

*msg*93 ATTCTGTGTGTACAGCCAGCAAAAGCAGCCCGGTTAATTACACACGATCA 1871

*msg*78 TTACTATGTATTGATCCACTTAAAGCAGTTAGAATACTTGCAGCTGATAT 1874

* *** * * *** ** * * **

*msg*32 ACATATGAAGGCAGATTTATTACAGAAAGATTTGAACAAGAAGCGGGATT 1801

*msg*24 ATATATAAAGACAGATTTATTACGGGAAGATTTGAATAAAAAACGTGATT 1849

*msg*84 GCGTATAAAAACGGAATTATTACACGAAGATTTGAACGAAAAACGGGATT 1842

*msg*56 GCATTTTAAAGTGGATCTATTACAGGAATATTTGAATGCAAGACGAGATC 1819

*msg*6 GCATTTTAAACTAGACCTATTACAAGAATATTTGAATGCAAGGCGAGATC 1837

*msg*94 TCAAATGAGAGTTATCTTTTTACGACAACAGTTGGATAAAAAACGAGATT 1887

*msg*45 TCAAATGAGAGTTATCTTTTTACGACAACAGTTGGATAAAAAACGAGATT 1875

*msg*41 TCGAA--AGAACTATCTTTTTACGACAACAATTGGATCAAAAGCGAGATT 1883

*msg*103 TCAAATGAGGGTTATCTTTTTACGACAACAATTGGATCAAAAGAGAGATT 1923

*msg*93 TCAAATGAGAGTTATCTTTTTACGACAACAGTTGGATCAAAAGCGAGATT 1921

*msg*78 CCAAACAAGAGCGATCTTTTTGCGGAAACAATTGGATCAAAAGCGAGATT 1924

* * ** * ** * *** * * * ***

*msg*32 TTCCTACGAAGTGGGATTGCAGGGAATTGCAAAAGAAATGTAATGATCTA 1851

*msg*24 TCCCTACGAAGCAGGATTGCAAGGAATTGCAAAAGAAATGCAATGATTTA 1899

*msg*84 TTCCTACCAAGCAGGATTGCAGGGAATTACAAAAGAAATGCGATAATCTA 1892

*msg*56 TTCCTACGAAACGAGATTGCAAGATATTATTGAAAAAATGTAAAGATCTA 1869

*msg*6 TTCCTACGAAACGAGATTGCAGGATATTATTGAAAAAATGTGAAGATCTA 1887

*msg*94 TTCCAACGGATAAAGATTGCAAAGAATTAGGGAGAAAATGCCAAGATTTA 1937

*msg*45 TTCCAACGGACAAAGATTGCAAAGAATTAGGGAGAAAATGCCAAGATTTA 1925

*msg*41 TTCCAACGGACAAAGACTGCAAGGAATTAGGAAGAAAGTGTGAAACTTTA 1933

*msg*103 TTCCGACAGATAAAGACTGCAAGGAATTAGGGAAAAAATGCCAAGATTTA 1973

*msg*93 TTCCGACAGATAAAGACTGCAAGGAATTGGGGAGAAAGTGTGAAGCTTTA 1971

*msg*78 TTCCAACAGACAAAGACTGCAAGGAATTGGGGAGAAAGTGTGAAGCTTTA 1974

* ** ** * ** **** *** * ** ** * * **

*msg*32 AAACAAGACTTTGAGGAACTTGAGTGGCCTTGTCATACGCTGAAACACCA 1901

*msg*24 AAACAAGACTTTAAAGGACTTGAATGGCCCTGTCATACGCTGGAGTATCA 1949

*msg*84 AAACAAGACTTTAAAGGACTTGAATGGCCCTGTCATACGCTGGAGCATCA 1942

*msg*56 GTACAAGATTCTGAAGAAATTGAGTGGCCTTGTCATACACTTAAACAGAA 1919

*msg*6 AAACAAGACTTTGAGGAACTTGAATGGCCCTGTCGCACGCTGGAACATCA 1937

*msg*94 GGAAAGGATTCAAAAGAAATTACGTGGCCATGTCATACACTGGAGCAGCA 1987

*msg*45 GGAAAGGATTCAAAAGAAATTACGTGGCCATGTCATACACTGGAGCAGCA 1975

*msg*41 GGAAAGGATTCAAATCAGATTCAATGGCCATGTCATACGCTAAAGCAACA 1983

*msg*103 GGAAAGGATTCAAAAGAAATTACATGGCCATGTCATACACTGGAGCAGCA 2023

*msg*93 GGAAAGGATTCAAATCAGATTCAATGGCCATGTCATACACTGGAGCAGCA 2021

*msg*78 GGAAAGGATTCAAATCAGGTTCAATGGCCATGTCATACGCTAAAACAACA 2024

* * ** * * ** ***** **** ** ** * * *

*msg*32 TTGCAATAGGCTGGAGATTGTGGAACAATTGGAAGAGAGATTGTTGGAAG 1951

*msg*24 TTGTAATAGACTAAATGTTGTAGGACAATTAGAAGAGAGGCTATTGGAAG 1999

*msg*84 TTGTAATAGACTAAATGTTGTAGGACAATTAGAAGAGAGGCTATTGGAAG 1992

*msg*56 TTGTGATCGTTTAAAGGTTGTAGAGCAATTGGAAGAAAAATTTTTGGAAG 1969

*msg*6 TTGCAATAGATTAGATATTGCAGAACAATTAGAAGAAAAGTTCTTGGAAG 1987

*msg*94 ATGCAATCGCTTGGGGACTACAGAAATTTTAAAGCAGGTTTTATTGGATG 2037

*msg*45 ATGCAATCGCTTGGGGACTACAGAAATTTTGAAGCAGGTTTTACTAAATG 2025

*msg*41 GTGTGATCGCTTG-GGACTACAGAAAT-TT-AAGCAGGTTTTACTAGGTG 2030

*msg*103 ATGCAATCGCTTGGGGACTACAGAAATTTTAAAGCAGGTTTTATTGGATG 2073

*msg*93 ATGCAATCGCTTGGGGACTACAGAAATTTTGAAGCAGGTTTTATTGGATG 2071

*msg*78 GTGTGATCGCTTGGGGACTACAGAAATCTTGAAGCAGGTTTTACTGGATG 2074

** ** * * * * ** * * * * *

*msg*32 AAAACGTGAAGGATTTAAAAAATGAAACCGTTTGTAAAAAAAGTGTTAAG 2001

*msg*24 GAAAAATAAAAGATTTAAAAAATGAAACTGTCTGTAAAGAGAATGTCGAA 2049

*msg*84 GAAAAATAAAAGATTTAAAAAATAAAACCGTCTGTAAAGAGAATGTCGAA 2042

*msg*56 AAAAAATGGAAAAATTGGATGACTCGAATTCATGTATAGAAAAGATTGGT 2019

*msg*6 AAAAGGTGAAAGATTTGGACAAGTTCGATTTATGTTTAAAAAGTCTGAAA 2037

*msg*94 AACACAAGGATACTTTGAAAACTCATGAAAACTGTGTAACATATTTAAAG 2087

*msg*45 AACACAAGGATACTTTGAAAACTCATGAAAACTGTGTAACATATTTAAAG 2075

*msg*41 AACACAAGGATACTTTGAAAGACCAAGAAAGTTGTGTAAAATATCTAAAA 2080

*msg*103 AACACAAAGATACTTTGAAAGACCAAGAAAGTTGTGTAAAATACCTAAAA 2123

*msg*93 AACACAAGGATACTTTGAAAGATGAAGAAAGTTGTCTAAAATATCTAAAA 2121

*msg*78 AACATAAGGATACTTTGAAAGATGAAGAAAGTTGTGTAAAATATCTAAAA 2124

* * * ** * *** * *

*msg*32 GAGCAGTGCAATCATTGGGTTGAAAAAAGAAAAACA-CAGTTTGCTCTTG 2050

*msg*24 GAACAATGCAATCATTGGGTTAAAAAAAGGAAAACA-CAGTTTGCTCTTG 2098

*msg*84 GAACAATGCAATCATTGGGTTAAAAAAAGGAAAATA-CTGTTTGCTCTTG 2091

*msg*56 CAGCGATGTCGTGAATGGAATAGAAGAGGAAGAACA-CACTTTGATCTCG 2068

*msg*6 GAACAATGTCATGAGTGGAATAGAAGAGGAAGAGTG-CAGTTTGCTCTCG 2086

*msg*94 GAAAAGTGTAATAAATGGTCTAGAAGG-GC-GATGACCGTTTCTCTTTTG 2135

*msg*45 GAAAAGTGTAATAAATGGTCTAGAAGGGGT-AATAATCATTTTTCTCTTG 2124

*msg*41 GAAAAGTGTAATAAATGGTCTAGAAGGGGT-GATGATCGTTTCTCTTTTG 2129

*msg*103 GAAAAGTGTAATAAATGGTCTAGAAGAGGA-GATGACCGTTTCTCTTTTG 2172

*msg*93 GAAAAGTGTAATAAATGGTCTAGAAGAGGC-GATGACCGTTTCTCTTTTG 2170

*msg*78 GAAAAGTGTAATAAATGGTCTAGAAGAGGA-GATGATCGTTTCTCTTTTG 2173

* ** * * *** * ** * * * ** * * *

*msg*32 GATGTCTAGCACATAATGTTACTTGCAAGATCATCACAGAAAGTCTTGTG 2100

*msg*24 GATGTGTAGCACAGAATGTTACATGCAAGATCATCACAGAAAGTCTTATA 2148

*msg*84 GATGTGTAGCACAGAATGCTACTTGCAAGATCATCACAGAAAGCGTAGAC 2141

*msg*56 CATGTGTAACACAGAATACTAGTTGCAAGATTCTTACAGAAAGCATAGGG 2118

*msg*6 CATGCGTAGCACAGAATATTACATGCAAGATTCTTACAGAAAGCGTAAAC 2136

*msg*94 TATGTGTTTTCCAAAACGCTACGTGTAAGC--ATGGTAAAAGACGTACAA 2183

*msg*45 TATGTGTCTTCCAAAATGCTACGTGTAAGCTGATGGTAGATGATGTGAAA 2174

*msg*41 TATGTGTTTTCCAAAACGCTACGTGTGAGCTGATGGTAAAAGACGTACAA 2179

*msg*103 TATGTGTCTTCCAAAACGCTACGTGTGAGCTGATGGTAAAAGACGTGAAA 2222

*msg*93 TATGTGTCTTCCAAAATGCTACGTGTGAGCTGATGGTAAAAGACGTACAA 2220

*msg*78 TATGTGTTTTCCAAAACGCTACATGTGAGCTGATGGTAAAAGACGTGCAA 2223

*** * ** ** ** ** ** * * * *

*msg*32 CTTAAATGTAGTTCATTAGAAAAACATATAGAGACTCTAAAAGTTGTAGA 2150

*msg*24 CTTAAATGTAGTTCATTGGAAAATCATATAGAGACTCTAAAAGTTGTAGA 2198

*msg*84 TCTAAATGCATTGCATTGAAAGAAAATATGAATACTCTAAAAGTTGTAGA 2191

*msg*56 TCTAAGTGTACTACATTGAAGGCACGTATGAAAACAAGTGATGTTATAAA 2168

*msg*6 TTTAAGTGTATTACGTTGGGTGTGCGTATCGAAGCAAGTAATGTTATAAA 2186

*msg*94 GATAGGTGCAAAATATTCAAAGAAAATATAAAAGTTTCAGAAATTGTTGA 2233

*msg*45 GATAGATGTGAAGTATTCGAAAAAAATATAAAAGCATCAGAGATTGTTGG 2224

*msg*41 GATAGGTGCAAAGTATTTAAGGAAAATATACAAGTATCAGAGATCGTTGA 2229

*msg*103 GACAGGTGTGAAGTATTCAAAAAAAATATAAAAGCTTCATATATTATTGA 2272

*msg*93 GATAGGTGCAAAGTATTCAAAGAAAATATAGAGAATTCAAAAATCATTGG 2270

*msg*78 GATAGGTGTAAAGTATTCAAAGAAAATATAAAAGCTTCAGAGATTGTTGA 2273

* ** ** *** * * * *

*msg*32 AGA------------TGCAAAA-AAGAAG-ACCA-AAAAGAAAAAACTTG 2185

*msg*24 AGA------------TGCAAAAAAAGAAG-ACCA-AAAAGAAAAAACTTG 2234

*msg*84 CAG------------AGCAGAAAAAAAAC-AAGA-AAGAGAATCAACCTG 2227

*msg*56 GAC------------TGCAAAA-GAAG-AAACAACAATGGAAGAAACATG 2204

*msg*6 TCA------------AGCAAAG-AGTAATAACCA-AAAGGAATCAACCTG 2222

*msg*94 TTTTCTTAAAAATAATACGAAT-AAC-ATAACAACACTGGAAAGAAACTG 2281

*msg*45 ATTTCTTAAAAATAATACAAAT-AAC-ATAACAACATTAGGAAATGTTTG 2272

*msg*41 GTTTCTTAAAAATAACACGAAT-AAC-ATAACAACATTAGGAAATGTTTG 2277

*msg*103 ATTTCTTGAAAATAATACAAAT-AAA-ATAACAACACTGGAAAGAAATTG 2320

*msg*93 ATTTCTTAAAAATAACACGGAT-AAA-ATAACAAGATTAGCAAATGTTTG 2318

*msg*78 TTTTCTTAAAAATAACACGAAT-AAC-ATAACAACACTGGAAAGAAACTG 2321

* * * * * * * **

*msg*32 CAGTTTTTGGGAACCGTATTGTGACAAATTCATGTTTAGCTGTGATAATT 2235

*msg*24 CAGTTTTTGGGAACCGTATTGTGACAAATTCATGTTTAGCTGCGATAATT 2284

*msg*84 TAATTTTTGGGAACCGTATTGTGACAAATTCATGTTTAGCTGCGATAACT 2277

*msg*56 TGATTCTTGGATACCCTATTGCAGCAAATTTGTATCAAGTTGTCATAATC 2254

*msg*6 CAACTCATGGACACCTTATTGCAGCAAGTTTATGTCAAGTTGTTATAATT 2272

*msg*94 TCCCTCCTGGCATACGTACTGCAATAGATTTTCATCTAATTGTCCAGATT 2331

*msg*45 CCCATTTTGGGACCCATATTGTGACAAATTTTCACCTAATTGTCTAGATT 2322

*msg*41 CCCATTTTGGGACCCATATTGTGACAAATTTTCGCCTAATTGTCCAGATC 2327

*msg*103 TCCCTCTTGGCATACGTATTGCAATAGATTTTCACCTAATTGTCCAGGTC 2370

*msg*93 CCCATTTTGGGACCCATATTGTGATAAATTTTCGCCTAATTGTCTAGATC 2368

*msg*78 TCCCTCCTGGCATACGTACTGCAATAGATTTTCACCTAATTGTCCAGATC 2371

* *** * ** ** * ** * **

*msg*32 --ATGAATGATGGCAATAAAGTGTGTAA-AAACTTAAAAAGAATTGCAAG 2282

*msg*24 TAATAAATGATAACAATAAAGTATGCAAAAAACTTAAAAAGAATTGCAAG 2334

*msg*84 TAATAGCTAATGGTAATGGAGAGTGTGAGAAACTTAAAAAGAATTGCGAG 2327

*msg*56 TAACGACTTCTGGTGGTGGAGAGTGTGAAGAACTTAACAAGGAGTGTAAA 2304

*msg*6 TAACGACTGCTGGTGGTGGAAAGTGTGAAGAACTTAACAAGGAATGCGAA 2322

*msg*94 TTTCAAAAAA---GAATC---CTTGTACAAAAATTAAAAACAATTGTAAG 2375

*msg*45 TTTCAAAAAA---GAATC---CTTGTACAAAAATTAAAAACAATTGTAAG 2366

*msg*41 TTACGAAAAA---AAATACTCTTTGTACAAAGATCAAAGACCATTGTAAG 2374

*msg*103 TTACGAAAGA---GAATA---GTTGTACAAAAATCAAGAAGCATTGTGAG 2414

*msg*93 TTTTAAAAAA---GGATA---CTTGTACAAAAATTAAAAAACATTGTAAA 2412

*msg*78 TTACGAAAGA---GGATA---GTTGTACAAAAGTCAAGAAACATTGTGAG 2415

* ** * * ** * * ** *

*msg*32 CCATACAGAGAAAGATATAACCTGGAAACACAAGTTATGTACGAGTTTAG 2332

*msg*24 CCATACAGAGAAAGATATAATCTGGAAACACATGTTATGTATGAATTTAG 2384

*msg*84 TCATACAGAGAAAGATATAACCTGGAAACACAAGTTATGTACGAGTTTAG 2377

*msg*56 TCATTCATTGAAAGGAAAGAATTGGAGGAAAAAGTGATCGATGAGTTGAA 2354

*msg*6 ACGTTCATCAAAAAGAAAGAGTTGGAATTAAAACTAGTTGACCAATTGAA 2372

*msg*94 CCATTTTATGAAAGAAAAGCCTTGGAAGACGCTCTCAAAGTAGAGCTTCG 2425

*msg*45 CCATTTTATGAAAGAAAAGCCTTGGAAGACGCTCTCAAAGTTGAACTTCG 2416

*msg*41 CCATTTTATGAAAGAAAGGCCTTAGAAGATGCTCTCAAAGTAAAGCTTCG 2424

*msg*103 CCGTTCTATAAAAGAAAGGCCTTGGAAGATGCTCTCAAAGTAGAGCTTCA 2464

*msg*93 CCATTTTATGAAAGAAAAGCCTTGGAAGACTCTCTCAAGGTTGAACTTCG 2462

*msg*78 CCGTTCTATAAAAGAAAGGCCTTGGAAGATGCTCTCAAAGTAGAGCTTCG 2465

* * *** * * ** * * *

*msg*32 AGGGAAGCTTAAAG-----AAGAGCTGTAGAACAACTCTTGACCAATATT 2377

*msg*24 AGGGAAGCTTAAAGAAGAAGTGAGCTGTAGAACAACTCTTGACCAATATT 2434

*msg*84 AGGAAAGCTTAA-GAAAAAAAGAACTGTAGAACAACTCTTGATCAATATT 2426

*msg*56 AGGTAGCTTAAAAACAGAACAAACATGTAAGGAGACACTTAATAAATATT 2404

*msg*6 GGGTCATTTGAACACAAAAGAAAAATGTAAGGGGGAGCTTGACAAATACT 2422

*msg*94 AGGAAAGCTGAGTGATGAAAACAAATGTACTGCAGCATTGAAGGGATATT 2475

*msg*45 AGGAAAGTTGAGTGAGAAAAATGGATGTGCTACAGCATTAGAAAGATATT 2466

*msg*41 AGGAAAGCTGAGTGATGAAAACAAATGTACTACAGAATTA-AAGGATATT 2473

*msg*103 AGGAAAATTGACTGATAAATCTAAATGTGAACCTGCATTGAAAAGATATT 2514

*msg*93 AGGAAAGTTGAATAAGAAAAATGAATGTACTACAGCATTAGAAGGATATT 2512

*msg*78 AGGAAAGCTGAGTGATAAAAACAAATGTACTACAGAATTAAAGGGATATT 2515

** * * *** * * *** *

*msg*32 GTACACAGTGGAGTAAGACAAAAAACAGTACATTGGAGAGTCTTTGCACT 2427

*msg*24 GTACACAGTGGAGTAAGACAAAAAATAAAACATTGGAGAGTCTATGCAAT 2484

*msg*84 GTACACAGTGGAGTAAGACAAAAAACAGTACACTGGAAAGTCTATGTACT 2476

*msg*56 GTACGCAATGGGAGAATGCAACCAATCAG---CTTAGCACTTTGTGCACA 2451

*msg*6 GTACACAATGGGCGAATGCAAGTAATGGA---CTTGAAACTTTCTGCACA 2469

*msg*94 GTACACTAGCGGGAAATGTAAATAACGCATCAGTCAGAAGTTTATGCAAA 2525

*msg*45 GTACAGTAGCGGGAAACGCAAATAATGCGTCAATCAGAAGTTTATGTAAA 2516

*msg*41 GTACAA-AGCG-AAAACGTAAATAATGCGTCAATCAGTGGTTTATGCAAA 2521

*msg*103 GTACAGTAGCGGGAAACGTAAATAATGCGTCAATCAGTGGCTTATGCAAA 2564

*msg*93 GTACAATAGCAGGAAACGTAAATAATGCGTCAATCAAAAGTTTATGTAAA 2562

*msg*78 GTACAATAGCGAAAAACGTAAATAATGCGTCAATCAATGGTTTATGCAAA 2565

**** ** ** ** * * ** *

*msg*32 GACA------AAAATGGCAA------TAATGA---TACGATTAGGGATAA 2462

*msg*24 AGTA---CTAATGGTGTTGA------TAATGA---TAAGATTAGAGATGA 2522

*msg*84 GACA------AGGGTGGCAA------TAATGA---TACAATTAGGGATGA 2511

*msg*56 GACA------AAACTAATAAA---A---ATGG---AAATGTCAAGAAAAA 2486

*msg*6 AACA------AGAAAAAAAAAAGCAAGCAAGA---TGATCTTAGAAAAGA 2510

*msg*94 GATAACACTCAAGGTAGCAATAAAAAGACTGACGAAAAAGTGGTAGAAGA 2575

*msg*45 GATAACACCAAGGGTAACCCTAAAAAGGATGA---TGAAGTTAGAAAGGA 2563

*msg*41 GATAGCACTCAAGGTAATAATAAAAAGCCTGACGACAAAGTGGTAGAAGA 2571

*msg*103 GCTAACACCAAGGATAACTCTGGAAAGAGTGATGAGGATGCTAGAAAGGA 2614

*msg*93 GATACTACTGATAACAAGTCTAAAAAGGATGACAATAAAGTTAGAGAAGA 2612

*msg*78 GATAACACTCAAGGTAATAATAAAAAGCCTGACGACAAAGTGGTAGAAGA 2615

* * * * *

*msg*32 TTTATGTAAAAAATTATTAAAACGAGTAAAGGCAAGATGTACAGAATTAT 2512

*msg*24 TTTATGTAAAAAACTAGTAGAACGAGTAAAGACAAGATGTACAGAATTGT 2572

*msg*84 TTTATGTAAAAAAC--GTGAAACGAGTAGAGGCAAGATGTGCAGAATTGT 2559

*msg*56 ACTCTGCAAAAAACTAGTAGAACGAGTAAAAAAGCAATGTCCTGTATTAA 2536

*msg*6 ACTTTGCGAGAAATTGGTAGAACAAGTAAAAAAGCGATGTCCTGGGTTAA 2560

*msg*94 GCTTTGTAAGAAATTGATGGAAGAAGTAAAAGAGCAATGCGAGACATTAC 2625

*msg*45 ACTCTGTGAGAAATTAGTGAAAGAAGTGGAAGAACAGTGCAAAGCATTAC 2613

*msg*41 GCTTTGTAAGAAATTGATGGAAGAAATAAAAGAGCAATGTGAGACATTAC 2621

*msg*103 ACTCTGTGAGAAATTAGTGAAAGAAGTGGAAGAACAGTGCAAAGCATTAC 2664

*msg*93 GCTCTGTGAGAAATTAATGGAAGAAGTGAAAGAACAATGTAAAACATTAC 2662

*msg*78 GCTTTGTAAGAAATTGATGGAAGAAATAAAAGAGCAATGTGAGACATTAC 2665

* ** * *** * ** * * * ** **

*msg*32 CTACGGAATTAAATACAGCAAAA-AA--GAGAAAAACAAAAGTAGAAGAA 2559

*msg*24 CTACAAAATTAAATACAGCGAAAGAAGGGATAAAAACAAAAGTAGAAGAA 2622

*msg*84 TTACGAAATTGAAGACAGCGAAAGAAGAGATAAAAATAAAAGTAGAAGAA 2609

*msg*56 AAGTAAAACTCACAAAGGCAAGTGAGGAACTATTTAAAAAAAAAGAAGA- 2585

*msg*6 AAAAAGAACTCACAGAGGCCAGTAAAGAGTTGGAGAAAAAAGC-TAACAA 2609

*msg*94 CAGCAGAATTAAAACAACCGGCAGACGATCTAGAAAAAGATG-TTAAGAC 2674

*msg*45 CAACAGAATTAGGGCAACCGGCTGCTGATCTAAAAAAAGATT-ATAAGAC 2662

*msg*41 CAGCAGAATTAACGGAGCTGGAAAAAAGCCTAGAAAAAGATG-TTAAAAC 2670

*msg*103 CAACAGAATTAGGACAACCGGCAGCTGATCTAAAAAAAGATT-ATAAGAC 2713

*msg*93 CAACAGAATTAGAACAACCAGAAAAAGATTTACAAGAAGATT-ATAAGAC 2711

*msg*78 CAGCAGAATTAACGGAGCTGGAAAAAAGCCTAGAAAAAGATG-TTAAAAC 2714

** * * * ** *

*msg*32 -GTTGGAAAATTGAATAAAGAGGCAAAAAAAGCATCAGAAGGAGCAAAAC 2608

*msg*24 -GTCAAAAAATTGAATGAAGGTGCAAAAAAAGCATCAGAAGGAGCAAAAC 2671

*msg*84 -GTCAAAAAATTGAATAAAGAAGCAAAAAAAGCATCAGAAGATGCAAAAC 2658

*msg*56 GTACAAGAAGCTTAAAGCTGAAGCAGTGAACGCAATGGATGATGCAAATC 2635

*msg*6 ATATGAGGATATCAA-AAAGAAGCAAAAGAAGCAATGGAAAAAGCAAATC 2658

*msg*94 ATATGAGGAACTTAAGGAAGAGGCAAAGAAAGCAATGAACAAGTCCAGCC 2724

*msg*45 ATATGAGGAACTTAAGAAACGTGCAGAGGAAGCAATGAACAAATCCAGTC 2712

*msg*41 ATATAAAGAACTTAAGAAAGAGGCAAAGAAAGCAATGAACAAGTCTAATC 2720

*msg*103 ATATGAGGAACTTAAGAAACGTGCAGAGGAAGCAATGAACAAGTCCAGTC 2763

*msg*93 ATATAAGGAACTTAAGAAACAGGCAGAAGAAGCAATGAACAAGTCCAATC 2761

*msg*78 ATATAAAGAACTTAAGAAAGAGGCAAAGAAAGCAATGAACAAGTCTAATC 2764

* * ** *** * *** * * * *

*msg*32 TTGTTTTATCATCATTAAAGAAAAATGAAGTGA-A--TGCCAAAAATACA 2655

*msg*24 TTGTTTTATCATCATTAAAGAAAAATGAAGTGA-A--TGCTAAAAATACA 2718

*msg*84 TTGTTTTATCATCATTAAAGAAAAATGAAGTGA-A--TTC---------A 2696

*msg*56 TCGTTTTATCAAAAGCAAAAGTAATGGATGACAAATCTGC---------A 2676

*msg*6 TTGTTTTATCAAAAGCAAAAGCAACGGATGACAAATCTGC---------A 2699

*msg*94 TTGTTTTATCATTCGTTAAGAAAGACGGAAATA-A--TAC---------A 2762

*msg*45 TTGTTTTGTCACTCATTAAGAAAAACGAAAGTA-A--TGT---------A 2750

*msg*41 TTGTTTTATCACTCGTTAAGAAAAACGAAAGTA-A--TAC---------A 2758

*msg*103 TTGTTTTGTCACTCATTAAGAAAAACGAAAGTA-A--TGT---------A 2801

*msg*93 TTGTATTATCACTCGTTAAGAAAAACGAAAGTA-A--TGC---------A 2799

*msg*78 TTGTTTTATCACTCGTTAAGAAAAACGAAAGTA-A--TAC---------A 2802

* ** ** *** ** * * * * * *

*msg*32 GCTGACAATGTG---------GCAGTGAATTCAGGGA----A---AAATG 2689

*msg*24 GCTGACAATGTG---------GCAGTGGATTCAGGGA----A---AAATG 2752

*msg*84 GAGA--AAAACG---------CAAGTAACAAT------------------ 2717

*msg*56 GGTAAAACAGCA---------CCATCAGTACCAGCACCAGCAGCACCATC 2717

*msg*6 GGTAAAACAGTA---------CCATCAGAATCAGCAC---CAGC------ 2731

*msg*94 CCGA--AAAATA---------ATAGCAAAAGCGAAGA----T---AAG-- 2792

*msg*45 TCAA--AAAGTA---------ATAGCAAAAACAAGGA----T---AAG-- 2780

*msg*41 TCAA--AAAATAATAGAAATAATAGCAAAAACAAGGA----T---AAG-- 2797

*msg*103 TCAA--AAAGTA---------ATAGCAAAAACAAGGA----T---AAG-- 2831

*msg*93 TCAA--AAGGTA---------ATAGTAAGGATAAG----------AAG-- 2826

*msg*78 TCAA--AAAATAATAGAAATAATAGCAAAAACAAGGA----T---AAG-- 2841

* *

*msg*32 CAACTAAC-GATCAAAATAAATCAGTAGGAGGAACAAATAGGACTCAAAA 2738

*msg*24 CAACTAAC-GATCAAAATAAATCAGTAGGAGGAACAAATAGGACTCAAAA 2801

*msg*84 ------------CAAAATAAACCAGTAGGAGGAGCAAATACGACACAAAA 2755

*msg*56 -A-GCACCAGCACCAAACACACCACCAACAGCACCAAATGGAACACAAAA 2765

*msg*6 -----ACCAGCACCGAACACACCACCAACAGCACCAAATGGAACACAAAA 2776

*msg*94 --AATGTC-GTTTCAAATGAAA----A--------AGATAC--CATAAA- 2824

*msg*45 --AATGCC-GTTTCAAACGGATT-TCA--------AGATAC--CACAGA- 2815

*msg*41 --AATGCC-GTTTCAAATGGACT-TCA--------AGATAC--CACAGA- 2832

*msg*103 --AATGCC-GTTTCAAACGGACT-TCA--------AGATAC--CACAAA- 2866

*msg*93 --AATGCT-GTTTCAAACGAG----CA--------AGATAC--CACAAA- 2858

*msg*78 --AATGCC-GTTTCAAACGGATT-TCA--------AGATAT--CACCGA- 2876

** * * ** * *

*msg*32 AACAC---AAGTCAAGCTTGTTCG--------------AAGAGGA-ACAG 2770

*msg*24 AACAC---AAGTCAAGCTTGTTCG--------------AAGAGGA-ACAG 2833

*msg*84 GACAC---AAGTCAGACTTGTTCG--------------GAGAGGA-ACAG 2787

*msg*56 CACAGTATTATTTAAACTTGTAAGAAGGAATATAAATACACCTGTGACAG 2815

*msg*6 CACAGTATTATTTAAACTTGAGAAGA--AATATAAACGCACCCGTGACAG 2824

*msg*94 ---AC---ATGTGAAAATACTACG--------------GAGAGGA-GTTA 2853

*msg*45 ---AC---ATATGAAAATACTACG--------------GAGAGGA-GTTA 2844

*msg*41 ---AC---ATGTGAAAATACTACG--------------GAG-GGA-GTTA 2860

*msg*103 ---AC---ATGTGAAAATACTACG--------------GAGAGGA-GTTA 2895

*msg*93 ---AC---ATGCGAAAATACTACG--------------GAGAGAA-GTTA 2887

*msg*78 ---AC---ATGTGAAAATACTACG--------------GAGAGGA-GTTA 2905

* * * *

*msg*32 TCGAAGTACTTGTCACAGAGGCAGAAGTTGAAGCCTTCGATGCAGTATCA 2820

*msg*24 TTGAAGTGCTTGTCACCGAGGCAGAAGTTGAAGCCTTCGATGCAGTATCA 2883

*msg*84 TCGAAGTACTTGTCACCGAGGCAGAAGTTGAAGCCTTCGATGCAGTATCA 2837

*msg*56 AAAAAACATATGTTACAGAAAAGGAGTTAAAAGCATTTGATTTGGTATCT 2865

*msg*6 AAAAAACATATGTTACAGAAAAGGAGTTAAAAGCATTTGATTTGGTATCT 2874

*msg*94 AGGATGTATTAGTAACAGAATTAGAAGCCAAGGCGTTTGACTTAGCAGCA 2903

*msg*45 AGGATGTACTTGTAACAGAATTGGAAGCCAAAGCATTTGATTTGGCAGCA 2894

*msg*41 AGGAGGCACTTG--ACAGAATCTGAAGCCAAGGCATTTGATTTAGCTGCA 2908

*msg*103 AGGATGTATCCGTAACAGAATTAGAAGCTAAAGCATTTGATTTGGCAGCA 2945

*msg*93 AGGATGTATCGGTAACGGAATCGGAAGTTAAAGCATTTGATTTGACAGCA 2937

*msg*78 AGGAGGCACTTGTAACAGAATCTGAAGCCAAGGCATTTGACCTAGCAGCA 2955

* * ** ** ** * ** ** ** *

*msg*32 AGAGCACTGGAAGCATATGTGGAAGTCAATGAAGAATGTAAAGTTTTGAA 2870

*msg*24 AGAGCACTGGAGGCGTATGTGGAAGTCAATGAAGAATGTAAAGTTTTGAA 2933

*msg*84 AGAGCACTGGAGGCGTATGTGGAAGTCAATGAAGAATGTAAAGTTTTGAA 2887

*msg*56 CAAGCATTTAGCTTGTATGTAGAATTAAAAGAATTGTGTCATGATTCAGA 2915

*msg*6 CAAGCATTTAGCTTGTATGTAGAGTTAAAAAAATTATGTCATGATTCAGA 2924

*msg*94 GAAGTGTTTGGAAGATATGTAGATTTAAAGGAAAGATGCGAGAAATTGAC 2953

*msg*45 GAAGTATTTGGAAGATATGTAGATTTGAAGGAAAGATGTGAGAAATTGGA 2944

*msg*41 GAAGTATTTGGAAGATATGTAGACTTGAAAGAAAGATGTGAGAAATTGAC 2958

*msg*103 GAAGTATTTGGAAGATATGTAGACTTGAAAGAAAGATGTAATAAATTGGA 2995

*msg*93 GAAGTATTAGGAAGATATATAGATTTAAAGGAAAAATGTAATAAATTGAC 2987

*msg*78 GATGTGTTTGGAAGATATGTAGATTTGAAGGAAAGATGTGAGAAATTAAA 3005

* * *** * ** * ** ** ** * *

*msg*32 ATTAGAATGTGGGTTTAAAGAAGAATGTTCGACATTGAAAGATGCATGCA 2920

*msg*24 ATTAGAATGTGGGTTTAAAGAAGAGTGCCCGACATTACAAGATGCATGCA 2983

*msg*84 ATTAGAATGTGGGTTTAAAGAAGAATGCCCAACATTAAAAAATGCATGCA 2937

*msg*56 GAAAGGATGTGGATTTAAGGAAGAATGTGAAGACATTAAAGAAGCATGCA 2965

*msg*6 GAAAGGATGTGGATTTAAGGAGGAATGCAAAGACATTAAGGACGCATGCA 2974

*msg*94 CTTGGATTGTGGGATTAAAGACGATTGCGATGGTTTAAAAGGTGTGTGTG 3003

*msg*45 ATCGGATTGCGGGATTAAAGAGGATTGCAAAGATTTAAAAGATGTGTGTG 2994

*msg*41 CTCAGGTTGCGGGATTAAAGACGATTGTAAAGATCTAGAAAATGTATGCA 3008

*msg*103 ATCAGATTGCGGAATTAAGGAGGATTGCAAAGACTTAGAAGAAGTATGCA 3045

*msg*93 CTCAGATTGCGGGATTAAAGACGATTGCGATGCTTTAAAAAGTGTGTGTA 3037

*msg*78 ATCGGATTGCGGGATTAAAGACGATTGTAAAGATCTAGAAAATGTATGCA 3055

* ** ** **** ** ** ** * * * **

*msg*32 GTGAAATTGAGAAGGCATGCAACGAATTGAAGTCATTGGAAGTGAAACCA 2970

*msg*24 GTAAAATTGAGAAGGCATGCAACGAATTGAAGTCATTGGAAGTGAAACCA 3033

*msg*84 GTGAAATTGAGAAGGCATGCAACGAATTGAAGTCATTGGAAGTGAAACCA 2987

*msg*56 CCAAAATTAAAAAAGCATGTGATGGATTGAAACCATTAGAAATAAAGCCA 3015

*msg*6 CTAAAATTGAAAAAGCATGTGGTGGATTGAAACCACTAGAAATAAAGCCA 3024

*msg*94 GAAAGATTAAGAAGAAATGTCGCGATCTGAAGCCTCTGGAGGTGAAGTCG 3053

*msg*45 GAAAGATTGAGAAGATATGTCGCGATCTGAAGCCTCTGGAGGTGAAGTCG 3044

*msg*41 AAAAGATCGGGAAAACATGTAGCGATCTGAAGCCTCTGGAGGTGAAGTCG 3058

*msg*103 AAAAGATTAATAAGGCTTGTCGCAATCTGAAGCCTCTGGAGGTGAAGCCG 3095

*msg*93 AAAAGATACAAGGAGTATGTTCGAAATTAGAACCACTGAAAGTGAAGCTA 3087

*msg*78 AAAAGATCGAGAAAACATGTAGCGATCTGAAGCCTCTGGAGGTGAAGCCA 3105

* ** ** * * * * * * **

*msg*32 TACGAAGTAGAAACAACGACGACAACCACCACAACGACAACCACCACTAC 3020

*msg*24 TACGAAGTAGAAACAACGACAACAACCACCACAACCACAACCACCACTAC 3083

*msg*84 TACGAAGTAGAAACAACGACAACAACCACCACAACCACAACCACCACTAC 3037

*msg*56 CACGAAATAGTAACTAAAAACGT------AACAACCACAACCACAACGAC 3059

*msg*6 CACGAAATAGAAACAACGACAAC------AACAACCACAACAACAACAAC 3068

*msg*94 CACGAAATAGTCACAGAAAG---------CACAACGACGACCACGACG-- 3092

*msg*45 CACGAAATAGTCACAGAAAG---------CACAACGACGACCACAACGAC 3085

*msg*41 CACGAAATAGTCACAGAAAG---------CACAACAACGACCACAACGAC 3099

*msg*103 CACGAAATAGTCACAGAAAG---------CACAACGACGACCACAACGAC 3136

*msg*93 CACGAAACAGTAACAAAAAT------AAATATAACCACGGTCACAGAAAC 3131

*msg*78 CACGAAATAGTTACAGAAAG---------CACAACGACGACCACGACGAC 3146

***** ** ** * * *** ** **

*msg*32 AACAAC--AAAGACAGAAGGAGAAGGAAAGACGGC--AGAGTGCCAAACT 3066

*msg*24 AACAAC--AAAGACAGAGGGAGAAGGGAAGACGGT--AGACTGCCAAACT 3129

*msg*84 AACAAC--AAAGACAGAGGGAGAAGGAAAGGCAAC--AGACTGCCAGTCT 3083

*msg*56 GACAAC--AGAAACCGTTAAAGACGCAAAGGCAAC--AGACTGCCAGTCT 3105

*msg*6 AACAAC--AAAGACAGAGGGAGAAGGAAAGACGGC--AGAGTGCCAGTCT 3114

*msg*94 -ACAAC--A---ACCGTTACCGATCCGAAGGCAAC--AGAATGCAAATCC 3134

*msg*45 CACAAC--A---ACCGTTACCGATCCGAAGGCAAC--GGAATGCAAATCC 3128

*msg*41 AACAAC--A---ACCGTTACCGATCCGAAGGCAAC--AGAATGCAAATCC 3142

*msg*103 AACAAC--G---ACCGTTGCCGATCCGAAGGCAAC--AGAATGCAAATCT 3179

*msg*93 AGTCAAAGA--AGCAGAAAA--AACAGGAGATAGCGAAAAATGCAAATCT 3177

*msg*78 AACAAC--A---ACCGTTACCGATCCGAAGGCAAC--AGAATGCAAATCC 3189

* * * * ** * *** * *

*msg*32 CTGCAGACAACAGACACGTGGGTCACAA-AACATCAACACATACCAGCAC 3115

*msg*24 CTGCAGACGACAGACACGTGGGTCACAAAGACGTCGACCCATACTAGCAC 3179

*msg*84 CTACAGACGACAGACACATGGGTCACAAAGACGTCGACTCATACCAGCAC 3133

*msg*56 CTGCAGACGACAGACACGTGGGTCACAAAGACGTCAACGCACACTAGCAC 3155

*msg*6 CTGCAGACAACAGACACATGGGTCACAAAGACGTCGACCCATACTAGCAC 3164

*msg*94 TTACAGACAACAGACACATGGGTTACACAGACATCGACACACACAAGCAC 3184

*msg*45 TTACAGACAACAGACACATGGGTTACACAGACATCGACACACACAAGCAC 3178

*msg*41 TTACAGACAACAGACACATGGGTTACACAGACATCGACACACACGAGTAC 3192

*msg*103 TTACAGACAACAGATACATGGGTTACACAGACTTCGACACACACAAGCAC 3229

*msg*93 CTCAGCACAACAGACACATGGGTTACACAGACATCGACACACACAAGCAC 3227

*msg*78 TTACAGACAACAGATACATGGGTTACACAGACATCGACACACACAAGCAC 3239

* ** ***** ** ***** *** ** ** ** ** ** ** **

*msg*32 ATCTACGACCACATCCACAGTCACCTCAAGA--AACACTCACATCAACAA 3163

*msg*24 TTCTACGACTACGTCCACAGTCACATCAAGAATAACGTTGACCTCGACGA 3229

*msg*84 ATCTACGACTACGTCCACAGTCACATCAAGAATAACGTTGACCTCGACGA 3183

*msg*56 TTCTACGACTACATCCACAGTCACGTCAAGAATAACACTCACTTCGACAA 3205

*msg*6 ATCCACAACCACATCTACAGTCACGTCAAGAATAACGTTGACCTCGACAA 3214

*msg*94 GTCTACCATCACATCTACAATCACATCAAAAATAACATTGACATCAACGA 3234

*msg*45 GTCTACCATCACATCTACAATCACATCAAAAATAACATTGACATCAACAA 3228

*msg*41 ATCTACCATCACATCTACGATTACATCAAAAATAACACTCACATCAACGA 3242

*msg*103 GTCTACCATCACATCTACAATTACATCAAAA---ACACTCACATCAACAA 3276

*msg*93 GTCTACCATCACATCTACGATTACATCAAAAATAACACTCACATCAACGA 3277

*msg*78 GTCTACCATCACATCTACGATTACATCAAAAATAACACTCACATCAACGA 3289

** ** * ** ** ** * ** **** * ** * ** ** ** *

*msg*32 GGCGGTGTAAGCCTACGAAGTGTACGACAGGAGAGGAAGATGATGCAGGA 3213

*msg*24 GGCGGTGTAAGCCTACGAAGTGCACCACCGGA------GACGATCCAGAA 3273

*msg*84 GGCGGTGTAAGCCTACGAAGTGTACGACAGGAGAGGAAGATGAAGCAGGA 3233

*msg*56 GAAGATGTAAACCGACCAAGTGTACGACAGGAGAGGAAGATGAAGCAGGA 3255

*msg*6 GGCGGTGTAAGCCTACGAAGTGTACGACAGGAGAGGAAGATGAAGCAGGA 3264

*msg*94 GGCGATGCAAACCAACCAAGTGTACCACAGGG------GATAATGCAGAG 3278

*msg*45 GGCGGTGTAAGCCTACAAAGTGTACCACAGGG------GATGATGCAGAG 3272

*msg*41 GGCGGTGCAAACCAACCAAGTGTACGACAGGG------GATGATGCAGAA 3286

*msg*103 GGCGGTGTAAGCCTACAAAGTGTACCACAGGG------GATGATGCAGAG 3320

*msg*93 GGCGTTGCAAACCAACCAAGTGTACGACAGGG------GATGATGCAGAG 3321

*msg*78 GGCGGTGCAAACCAACCAAGTGTACGACAGGG------GATGATGCAGAG 3333

* * ** ** ** ** ***** ** ** ** ** * ***

*msg*32 GACGTGAAACCGAGTGAGGGGCTGAGGATGAGTGGGTGGAGTGTGATGAG 3263

*msg*24 GATGTCAAGCCAAGTGAAGGTTTGAGAATGAATGGATGGAGTATAATGAA 3323

*msg*84 GAGGTGAAGCCGAGTGAGGGGCTGAGGATGAGTGGGTGGAGTGTGATGAG 3283

*msg*56 GACGTGAAGCCGAGTGAGGGATTAAGGGTGAGCGGGTGGAATGTGATGAG 3305

*msg*6 GACGTGAAACCGAGTGAAGGGTTGAGGATGAGTGGGTGGAGTGTGATGAA 3314

*msg*94 GACGTGAAACCGAATGAGGGATTGAAGATGAGTGGGTGGAGCGTGATGAG 3328

*msg*45 GACGTGAAACCGAGTGAAGGCTTGAGGGTGAGCGGGTGGAATGTAATAAA 3322

*msg*41 GACGTGAAGCCGAGTGAGGGATTGAAGATGAGTGGGTGGAGCGTGATGAG 3336

*msg*103 GACGTGAAACCGAGTGAGGGATTGAAGATGAGTGGGTGGAATGTGATGAG 3370

*msg*93 GACGTGAAGCCGAGTAAGGGATTGAAGATGGGTGGGTGGAGCGTGATGAG 3371

*msg*78 GACGTGAAACCGAGT--AGGCTTGAGGGTGAGCGGGTGGAATGTGATGAG 3381

** ** ** ** * * ** * * ** ** **** * ** *

*msg*32 GGGGGTGTTATTAGCAATGATGATTTCATTCATGATT**TGA** 3303

*msg*24 GGGGGTGTTACTAGCAATGATGATTTCATTCATGATT**TAA** 3363

*msg*84 GGGGGTGCTAGTGGCGATGATGATTTCATTCATGATT**TAA** 3323

*msg*56 GGGGGTGTTATTAGCAATGATGATTTCGTTTATGATT**TAG** 3345

*msg*6 GGGGGTGTTATTAGCAATGATGATTTCATTCATGATT**TAG** 3354

*msg*94 GGGGGTGATATTAGCAATGATGATTTCAATAATGATT**TAA** 3368

*msg*45 AGGGGTGATAGTAGCAATGGTTATTTCGTTTATGATT**TGA** 3362

*msg*41 GGGGGTGATATTAGCAATGATGATTTCGTTCATGATT**TAG** 3376

*msg*103 GGGGGTGATAGTAGCAATGGTTATTTCGTTTATGATT**TAG** 3410

*msg*93 GGGGGTGATATTAGCAATGATGATTTCGTTCATGATT**TAG** 3411

*msg*78 GG----GATAGTAGCAATGGTTATTTCGTTCATGATT**TAG** 3417

* * ** * ** *** * ***** * *******

***msg*-II**

**upstream potential TATA box**

*msg*104 AGAG-----**TAT-----T-------AG-A**A-------------------- 12

*msg*76 GTAA-----AAG----AT-------AG-AG-------------------- 13

*msg*79 AATA-----AAG----AG-------AGTAT-------------------- 14

*msg*85 **TATA-----AAA**----AT-------GA-AG-------------------- 13

*msg*7 **TATA-----AAG**-G--GTTTTAATTAA-AC-------------------- 21

*msg*25 AGCA-----AAA-G--AT-------AA-AT-------------------- 14

*msg*37 CCAA-----AAC----AT-----TTAC-CC-------------------- 15

*msg*68 AAACACACAAATACAAAT-------AA-ATTTTAAAAGTCATAATAATTA 42

*msg*62 ACAT----AAAG----AT-------AG-AA--CAA-------AA----TA 21

*msg*20 AAT-----AA-------T-------A----------------------TA 9

*msg*3 AAAA-----ATC----GT-------GA-AA-------------------- 13

*msg*104 -------------------------------------------------- 12

*msg*76 -------------------------------------------------- 13

*msg*79 -------------------------------------------------- 14

*msg*85 -------------------------------------------------- 13

*msg*7 -------------------------------------------------- 21

*msg*25 -------------------------------------------------- 14

*msg*37 -------------------------------------------------- 15

*msg*68 T-TATTTAAAAAACATTTTTACAACTTGAATTACTCTAGA----ATAAGG 87

*msg*62 T-AAG-----AAATAATAA-------TAAAAAATAAAAAAAGTACTAA-- 56

*msg*20 TTTAA-TA-AAAATAGCAT------------------------------- 26

*msg*3 -------------------------------------------------- 13

*msg*104 -------------------------------------------------- 12

*msg*76 -------------------------------------------------- 13

*msg*79 -------------------------------------------------- 14

*msg*85 -------------------------------------------------- 13

*msg*7 -------------------------------------------------- 21

*msg*25 -------------------------------------------------- 14

*msg*37 -------------------------------------------------- 15

*msg*68 A-AA---ATCAACAGAAAGTTTAAAAAAACAAAAAAATGCCA**TATAAAT**A 133

*msg*62 AAAACGGATCGTAAAAAAACTTAGGA------------------------ 82

*msg*20 --------TT---------------------------------------- 28

*msg*3 -------------------------------------------------- 13

*msg*104 -------------------------------------------------- 12

*msg*76 -------------------------------------------------- 13

*msg*79 -------------------------------------------------- 14

*msg*85 -------------------------------------------------- 13

*msg*7 -------------------------------------------------- 21

*msg*25 -------------------------------------------------- 14

*msg*37 -------------------------------------------------- 15

*msg*68 ATTTAAAATTTGTATTTCGATTCTAAAACCAAAAGCAAAAAATAAATGAA 183

*msg*62 -----TTA------------------------------------------ 85

*msg*20 -----ATATC---------------------------------------- 33

*msg*3 -------------------------------------------------- 13

*msg*104 -------------------------------------------------- 12

*msg*76 -------------------------------------------------- 13

*msg*79 -------------------------------------------------- 14

*msg*85 -------------------------------------------------- 13

*msg*7 -------------------------------------------------- 21

*msg*25 -------------------------------------------------- 14

*msg*37 -------------------------------------------------- 15

*msg*68 AAATAAATACAGAAAATTTTAATTATATTTCAAGAAAAATTC**TATAAAAG** 233

*msg*62 -------------------------------------------------- 85

*msg*20 -------------------------------------------------- 33

*msg*3 -------------------------------------------------- 13

*msg*104 ---------------------------------------A-G--TAAAAT 20

*msg*76 ---------------------------------------A-A--TA--AT 19

*msg*79 ------------------------------TA------GA-A-------- 19

*msg*85 ------------------------------TA------AA-A--GATAGA 24

*msg*7 ------------------------------TAGATTTAAA-AAATAGAAT 40

*msg*25 -------------------------------A------AATA--TA--TT 23

*msg*37 ------------------------------CA------AA-T--TA--CT 24

*msg*68 GTTTTAATTGTAAAAGGTTTTAAATTAAACTAGATTTAAA-AAATAGAAT 282

*msg*62 -------------------------------------------------- 85

*msg*20 -------------------------------------------------- 33

*msg*3 ------------------------------GA------GA-A--GAAAAT 24

*msg*104 CAT---AAAAAAT------------A--T--------------------- 32

*msg*76 -AT---TAAAAAG---------AGTA--T-TA-GAA-AGTA---AAA-AT 47

*msg*79 -------------------------------------AGTA---AAA-AT 28

*msg*85 GAA---TAATAAT---AA----AGTA--T-TA-GAG-AGTA---AAA-AT 55

*msg*7 CAAAGCAAAAAATAAAAACAATAATA--T-T**C-ATA-TTT**ATAGAAATAC 85

*msg*25 GAT--T-AAAAAA---------AACATCTTTT-G**TATATT--------A**T 52

*msg*37 -------TGCACT---------ACCC-CA-TG-GGG-AATA---ATT-TT 50

*msg*68 CAAAGCAAAAAATCAAAACAATAATA--T-TC-ATAT-TTATAGAAATAC 327

*msg*62 -------------------------------------------------- 85

*msg*20 -------------------------------------------------- 33

*msg*3 C-T---AAAAAAT---------TAAA--A-TAAGAA-TGTT---TTT-AT 53

*msg*104 ---------------------TAATA---AAA--T-------TA------ 43

*msg*76 CA--TAAAAAA**TAT-------TAAT**A---AAA--T-------TA------ 70

*msg*79 CA--TAAAAAA**TAT-------TAAT**A---AAA--T-------TG------ 51

*msg*85 CA--TAAAAAA**TAT-------TAAT**A---AAA--T-------TG------ 78

*msg*7 AA--TAAAAAATAGGGAATAGTAGTAAAAAAGTACTAGAAAATAGAAATG 133

*msg*25 AA--AAAAAAATAT-------GAATG---ACAA---------AA------ 75

*msg*37 AAAATGAAAAATT--------CAACAA---AA----------AA------ 73

*msg*68 AA--TAAAAAATAGGGAATAATAATAAAAAAGTACTAGAAAATAGAAATG 375

*msg*62 -------------------------------------------------- 85

*msg*20 -------------------------------------------------- 33

*msg*3 -A--**TATAAAA**TAT--------AATG---AAA----------AA------ 73

*msg*104 --------TATTG-TT--------TAAAA----C---------GTTTA-- 61

*msg*76 --------TATTG-TT--------TAAAA----C---------GTTTA-- 88

*msg*79 ----------TTA-TT--------TAAAA----T---------CTTTA-- 67

*msg*85 ----------TTA-TT--------TAAAA----T---------CTTTA-- 94

*msg*7 ATAGAAAAGATTG-CTAATAAAATTATAT----C---------ATTTA-- 167

*msg*25 ----------TTCTTT--------TAATA----A---------CTCAA-- 92

*msg*37 ----------TTT-AA--------AAAAA----A---------CACAA-- 89

*msg*68 ATAGAAAAGATTG-CTAATAA-----AA-TTGTT---------ATTTA-- 407

*msg*62 --------TCTCG-TT----G-----AA----TC---------ATTTA-- 102

*msg*20 --------------------------------------------TTTA-- 37

*msg*3 -----------TA-AT--------AAAAAA---CATTAAAATACACAATA 100

*

*msg*104 ------TTATATTT---TTATTTTAA-AAATATTTTTT--TATGAAAATT 99

*msg*76 ------TTATATTT---TTATTTTAA-AAATATTTTTT--TATGAAAATT 126

*msg*79 ----------------------TTAA-AAATATTTTTT--TGTGAGAATT 92

*msg*85 ----------------------TTAA-AAATATTTTTT--TGTGAGAATT 119

*msg*7 ------TAACATTTGCTATATTTTAA-GAATATCTTTT--TATAAAAATT 208

*msg*25 ----------------------A-AA-AAACTTCATCTAGAATGAGAATT 118

*msg*37 -------AAGA------ATATCATAT-AAATAATT--------TAAAAGT 117

*msg*68 ------TAACATTAACTATATTTTAA-GAATATTTTTT--TACAAAAATT 448

*msg*62 ------AAATATTTACTATAGTTTAA-AAATAGTTTTC--**TATAAAA**ATT 143

*msg*20 ------TTATGTAT---CTATTGTCA---AT---TCGT--TAT---TGTT 67

*msg*3 GAATAAAAATATAT---CTAATAAAAACAATATTTATT--TATC------ 139

*

*msg*104 TACGT-AT------TAGAAAGCAAAAAT-----CCACCAAAAGTAAAAAT 137

*msg*76 TACGT-AT------TAGAAAGCAAAAAT-----CCACCAAAAGTAAAAAT 164

*msg*79 TACAT-ATTAATATTAAAAATTAAAAAC-----TCAACGAGAGTCAAAAT 136

*msg*85 TACAT-ATTAATATTAAAAATTAAAAAC-----CCAACGAGAGTCAAAAT 163

*msg*7 TATAG-AT------TTAAAAGTAAAAAC-----TAAATGGAAAATAAAAC 246

*msg*25 AAAAAAATTTAAATAATAT-ACTAAGAT-----TTATCAATAGAAACAGT 162

*msg*37 TGCAT-TT------TA--ACTCTAAAATTAGATGCA--AAAAAATAAAAA 156

*msg*68 TATAG-AT------TTAAAAGTAAAAAC-----TAGATGAAAAATAAAAC 486

*msg*62 TATAG-AT------ATGAAAATAAAAAC-----TAGATAAAAAATAAAAC 181

*msg*20 TATGG------------------------------------------AAT 75

*msg*3 -------------------TACT--TAT-----TTATTGTTT-------- 155

*msg*104 ATCTAAG**TA-------TA-A--TA**CA----------AAAT--AATACACA 165

*msg*76 ATCTAAG**TA-------TA-A--TA**CA----------AAAT--AATACACA 192

*msg*79 ATCTAAGTA-------CA-A--TACAGTACAATTCAAAAT--GGTACATA 174

*msg*85 ATCTAAGTA-------CA-A--TACA----------GTAC--AATTCAAA 191

*msg*7 ATGTAAA**TA-------TA-A--TA**CA----------AAATATAATACAAA 276

*msg*25 ATATAAAAAACTTATGTA-AGTAACT----------AAAA--AATATAAT 199

*msg*37 ATATAATTG-------TA-A--**TATA----------AAA**T--AATATTTA 184

*msg*68 ATA-AAATG-------TA---ATACA----------AAA**TATAATA**CAAA 515

*msg*62 ATATAGTTG-------TA---A**TATA----------AAA**T--AATATTTA 209

*msg*20 -------TT-------TC---ATA---------------------TCAGA 87

*msg*3 -----ATTA-------TATA--TTCA----------T**TAT--AATT**TATT 179

*msg*104 TT--TACC--TAT----TTATTTATTGTA-T---------TTTATT--AT 195

*msg*76 TT--TACC--TAT----TTATTTATTGTA-T---------TTTATT--AT 222

*msg*79 TT--TGCC--TAT----TTATTTATTGTA-T---------TTTATT--AT 204

*msg*85 ATGGTACA--TATTTGCCTATTTATTGTA-T---------TTTATT--AT 227

*msg*7 AT--A--A--TAT----CTATCTATTGCA-TATCG-----ATTTTT--AA 308

*msg*25 AG--TAAA--AA-----TTAATACTTGTC-A---------GTCATT---T 227

*msg*37 TC--TATT--TAC-TGCATATTTATT--A-T---------TAATTT--GT 215

*msg*68 ACAATATC--TAT----CTATTTATTGCA-TATCGATTTTTAAT----AT 554

*msg*62 ------------T----TTATTCACTGCA-TATTTATTATTAATTT--GT 240

*msg*20 ------------G----TTGTT---------------------------T 94

*msg*3 ATCGTTTATCAATTCTTTTATCAATTACAAA---------TTTATTTAAA 220

*

*msg*104 TATCGT-TTATCGCT-ATT-TTTT-AGTGAG--AACGA--TATTTTTCGA 237

*msg*76 TATCGT-TTATCGCT-ATT-TTTT-AGTGAG--AACGA--TATTTTTCGA 264

*msg*79 TATCGT-TTATCGCT-ATT-TTTTAACTGAG--AACGA--TATTTTTCGA 247

*msg*85 TATCGT-TTATCGAT-ACT-TTTT-ACTAAG--AT--A--T-TTTTTCGA 266

*msg*7 TATCGT-TTACTGGT-ACTTTTTT-AATGAG--AATGG--TATTTTTCGA 351

*msg*25 TATTGG-ATATCGATGAAT-TTGT-AATAATTTATCGA--T-TTTTTCGA 271

*msg*37 TATCGC-TTATCGGT-ATT-TTTT-AATGAA--AATGA--T-TTTCTCGA 256

*msg*68 TATTGT-TTC----T-CTT-TTTT-AGTGAG--AACGA--TATTTTTCAA 592

*msg*62 TATCGC-T----------------------T--ATCGG--TATTTTTTAG 263

*msg*20 TCTCGA-TAAC---C-ACT-T-----ATGAA------A--TATTTTT--- 124

*msg*3 AATTGTTTTCTCGAT-ATT-TTAT-ATTAAA--TATGAAACATTTTTTAT 265

* * *** *

**TATA box Cap signal**

*msg*104 TGTCAAA--TGTGAAGCACTTTT-**TATAAAA**ATTATTATTTGTTAC**CA**TT 284

*msg*76 TGTCAAA--TGTGAAGCACTTTT-**TATAAAA**ATTATTATTTGTTAC**CA**TT 311

*msg*79 TGTCAAA--TGTGAAGCACTTTT-**TACAAAA**ATTATTATTTGTTAC**CA**TT 294

*msg*85 TGTCAAA--TATGAAGTACTTTT-**TATAAAA**ATTATTATTTGTTAT**CA**TT 313

*msg*7 TGTCAAA--TATGAAACATTTTT-**TATAAGA**ATTATCATTTGTTGT**CA**TT 398

*msg*25 TGTCA------TAAAACAT-TTT-**TATAAAA**ATTATTATTTGTTAT**CA**TT 313

*msg*37 TATTTTT---TTCAGTTAAA**TATAAAT**GA-AAGCGTTTATAGTTA--AAT 300

*msg*68 TGTCAAATATATGAAGCACTTTT-**TATAAAA**ATTATTATTTATTAT**CA**TT 641

*msg*62 TG--A-AAA------TGA-TTTT-CTC**GATATTT**TTTTT**CT**GTTA--AAT 300

*msg*20 --------------------**TATAAAC**CTTTATTTATTATTATTATTATT 152

*msg*3 AATTATT--TATTCAATATTCTC-AT**TATAAAT**GA------ATTTT**CA**TT 306

** * *

*msg*104 ATAG**ATG**AAAGCATTTGCATTAGCCAGCTTTCTTGGCATAGCGTATGCTT 334

*msg*76 ATAG**ATG**AAAGCATTTGTATTAGCCAACTTCCTTGGCATAACATATGCTT 361

*msg*79 ATAG**ATG**AAAGCATTTGCATTAGCCGGCTTTCTTGGCATAGCGTATGCTT 344

*msg*85 ATAG**ATG**AAAGCATTTGCATTAGTCAACTTTTTTGGCATAGCATATGCTT 363

*msg*7 GTAG**ATG**AAAGCATTTGCATTAGCCAGCTTTCTTGGCACAGCGTATGCTT 448

*msg*25 ATTG**ATG**AAAGCATTTGTATTAGCCAACTTTCTTGGCATAACATATGCTT 363

*msg*37 ATAA**ATG**AAAGCGTTTATACTAGCTGTTTTTTTTGGAATAATACGTGCTT 350

*msg*68 ATAG**ATG**AAAGCGTTTGCATTAACCAGTTTTCTTGGCATAGCCTATGCTT 691

*msg*62 ATAA**ATG**AAAACGTTTATAATAGCTGTTTTTTTTGGAATAATACATGCTT 350

*msg*20 ATAA**ATG**AAAGCACTTACTATATCATGTTTTTTTACAATAGCGTGCGCTT 202

*msg*3 ATAA**ATG**AAAGTGTTTATTTTAACATTTTTTCTTGGAATAGTACATGCAT 356

* ****** ** ** ** ** * * ** *

**<-----------------intron 1---------**

*msg*104 TTTCAAAAAACATTG**GTATTA**TATTTTATT-TTTTCGGATTTTTAACTT- 382

*msg*76 TCTCAAAAAACATTG**GTATTA**TATTTTATT-TTTTTATATTTTTAACTT- 409

*msg*79 TTTCAAAAAACATTG**GTATTA**TATTTTATT-TTTTCGGATTTTTAACTT- 392

*msg*85 TCTCGAAAAACATTG**GTATTA**TATTTTACT-TTTTTAGACTTTTAACTT- 411

*msg*7 TCTCAAAAAACATTG**GTATTA**TATTTTACT-TTTTTATATTTTTAATTTT 497

*msg*25 TTTCAAAAAACATTG**GTATTA**TATTTTACT-TTTTTATATTTTTAACTT- 411

*msg*37 TCTCAGAAAATATTG**GTATTT**TATTTTATT-CTTTATTTTGTTTAACTT- 398

*msg*68 TCTTAAAAAATATTG**GTATTA**TATTTTACT-TTTTTAGATTTTTAACTT- 739

*msg*62 TCTTAGAAAATATTG**GTATTT**TATTTTATT-CTTTATTTTGTTTAACTT- 398

*msg*20 TCTCA---AATATTG**GTACTT**TGTTTATTTTTTTTCTCATTTTTAACTT- 248

*msg*3 CATCAAAAAACATTG**GTATTT**TGTCTTTTC-TATTTCTTATTTTAATTT- 404

* ** ******* * * * * ** ***** **

**------>**

*msg*104 --TT**TAG**AATCTATTTATAAAGAATCTTTAAATCCATTACAAAATCCACC 430

*msg*76 TTTT**TAG**ATTCTATTTATAAAGAATCTTTAAATCTATTACAAAATCCATC 459

*msg*79 --TT**TAG**AATCTATTTATAAAGAATCTTTAAATCCATTACAAAATCCACC 440

*msg*85 TTTC**TAG**AATCTACTTATAAAGAATCTTTAAATCTATTACAAAATTCGTC 461

*msg*7 TTTT**TAG**AATCTATTTATAAAGAATCTTTAAATCCATTACAAAATCCATC 547

*msg*25 TTTT**TAG**AATCTATTTATAAAGAATTTTTAAATCTATTACAAAATCCATT 461

*msg*37 TCTT**TAG**AATTTATTCATAAAAAGTCTGT---TTCATT---AAATTCATT 442

*msg*68 TTTT**TAG**AATCTACTTATAAAGAATCTTTAAATCTATTACAAAATCCATC 789

*msg*62 TTTT**TAG**AATTTATTCATAAAAGATCTGT---TTCATT---AAATTCGTT 442

*msg*20 T-TT**TAG**AACTTATTCATAAAAGATCTGT---TTTATT---AAACTCATT 291

*msg*3 --TT**TAG**AATTTATTCATAAAAAATCTA------CATTA---GATCAGTT 443

* **** ** * ***** * * *** *

*msg*104 ------------ACAAAATCCACCACAAAATCCGTCACA----------- 457

*msg*76 ------------ACAAAATCCACCGCAAAATCCATCACAAAATTCGCCTC 497

*msg*79 ------------ACAAAATCCACCACAAAATCCGTCACA----------- 467

*msg*85 ------------ACAAAACCC------------ATCACA----------- 476

*msg*7 ------------ACAGAATCCATCACAGAATCCATCACA----------- 574

*msg*25 ACAAAATCCATTACAAAATCCATTACAAAATCCATCGCA----------- 500

*msg*37 ------------ACAAAATAC------------ATCATT----------- 457

*msg*68 ------------ACAAAAACCATCACAGAATTCACCGCA----------- 816

*msg*62 ------------GCAAAATACATC------------ATT----------- 457

*msg*20 ---------------AAATACATC------------ATT----------- 303

*msg*3 ------------ATTAGATAG------------GTCATT----------- 458

*

**<---------------------------**

*msg*104 -AAATCCATTATTGTATATAAC**GTACAA**AAACCTTTAAA----TAATACT 502

*msg*76 AAAATTCATTATCCCATATAAT**GTATAA**AGACCTTTAAATAAATAATACT 547

*msg*79 -AAATCCATTATTGTATATAAC**GTACAA**AAACCTTTAAA----TAATACT 512

*msg*85 -AAATCCATTATTCCGTATAAC**GTATAA**AAGCC-TTAAA----TAATACT 520

*msg*7 -AAATTCATTATCCTATATAAT**GTATAA**AAACCTTTAAA----TAATACT 619

*msg*25 -AAATCCATTATCGTATATAAC**GTATAA**AAACCTTTAAA----CAATACT 545

*msg*37 -TAATCCA---------ATAGT**GTATGA**A-ATCTTTAAG----TAACACC 492

*msg*68 -AAATTCATTATTGCATGTAGC**GTATAA**AAACCTTTAAA----CAATACT 861

*msg*62 -TACTCCA---------ATAAC**GTATGA**A-ACTTTTAAG----TAACATT 492

*msg*20 -AGATTCA---------ATAAG**GTATAA**AGACGTTTAAA----TAGCATT 339

*msg*3 -ATATTC---------AATAAC**GTATAA**AAACCTTTAAA----TAATCTC 494

* * ** *** ** **** *

**------------intron 2------------>**

*msg*104 AGAACTTGTAAAATTAACAAATTATTTATT**TAG**TGAAGGAAATATTTTTG 552

*msg*76 AGAACTTGTAAAATTAACAAATTATTTATC**TAG**TGAAAGGAATATTTTTG 597

*msg*79 AGAACTTGTAAAATTAACAAATTATTTATT**TAG**TGAAGGAAATATTTTTG 562

*msg*85 AGAACTTGTAAAATTAACAAATTATTTATC**TAG**TGAAGGAAATATTTTTG 570

*msg*7 AGAGCTTGTAAAGTTAACAAATTATTTATC**TAG**TAAAGGAAATATTTTTG 669

*msg*25 AGAACTTGTAAAATTAACAAATTATTTATC**TAG**TAAAGAAAATATTTTTG 595

*msg*37 AAAACATTTAAAATTAACAAATTTTTTATC**TAG**TGAAGAAAGAATTTTCG 542

*msg*68 AAAACTTGTAAAATTAACAAATTATTTATC**TAG**TGAAGAAAATATTTTTG 911

*msg*62 AAAACATTTAAAATTAACAAATTTTTTATC**TAG**TGAAGAAAGAATTTCCG 542

*msg*20 AATATATATATAATTACCA--TTTTTTATA**TAG**TGAAGAGAGAGTTTTTA 387

*msg*3 AAAATATATAAAATTAACGAATTTTTTATA**CAG**TAAAGAAAGGATTTTTG 544

* * ** * *** * ** ***** *** ** * ***

*msg*104 CTTTAATTCTAAAAA---ATGCAATGAATACTGAATGTAAAGCAAGGCTA 599

*msg*76 CTTTAATTCTAAAAG---ATGCAGTGAATGATGGATGCCAAGAAAAGCTA 644

*msg*79 CTTTAATTCTAAAAA---ATGCAATGAATACTGAATGTAAAGCAAGGCTA 609

*msg*85 CTTTAATTCTAAAAG---ATACAATGAATAATGAATGTCAAGAAAAGCTA 617

*msg*7 CTTTAATTCTAAAAG---ATGCAAAGGATAATCAATGTCAAGTGAAGCTA 716

*msg*25 CTTTAATTCTAAAAG---ATGCAACGAATAATCAATGTCAAATAAAACTA 642

*msg*37 CTTTAATTTTAAAAGAAAACGCAACAAATAATCAATGTAAAATAAAACTA 592

*msg*68 CTTTAATTCTAAAAG---ATGCAGTAAATAATGGATGCCAAGAAAAGCTA 958

*msg*62 CTTTAATTTTAAAAGAAAACGCAATAAATGGTGAATGTAAAATAAAACTG 592

*msg*20 TTTTAATCTTAAAAAAAAATACAATAACTGATCAATGTCAAGTAAAACTA 437

*msg*3 CTTTAATTCTAAAAGAAAATGCAATTAATAATCATTGTCATGAAAAGCTA 594

****** ***** * ** * * ** * * **

*msg*104 AGAAAATACTGTGAAAATTTAAGAAATATGACTCAAATGCTAAAAAGCTC 649

*msg*76 AAAAAATACTGTGAAGATTTAAGAAATATGACTCAAATGCTAAAAAGCTC 694

*msg*79 AGAAAATACTGTGAAAATTTAAGAAATATGACTCAAATTCTAGAAAGCTC 659

*msg*85 AAAAAATACTGTGAAAATTTAAGAAATATGACTCAAATGCTAGAAAGCTC 667

*msg*7 AAAAAATACTGTAAAGATTTAGAAGAAATGACTCAAATACTAAAAAGCTC 766

*msg*25 AAAAAATACTGTAAAGATTTAGAAGAGAAGGCTCAAATGTTAGAAAGCTC 692

*msg*37 AAAAAATATTGCGAAAATTTTAAAAATACAAATAAAGCACTAAGTGGTGT 642

*msg*68 AAAAAATACTGTGAAGATTTAAAAAAGGTGAGTCAAATGCTAAAAAGCTC 1008

*msg*62 AAAGAATACTGCGAAAATTTTAAAAATACAAATAAAATATTAAGTAGCGT 642

*msg*20 AAAGAATATTGTGGAAATTTAAAAAATATGGACCTAGAGCCAGAAAACTT 487

*msg*3 AAAGAATATTGTGAAGATTTGGAAAAAATAGGTCTAAATTTAAACAATTA 644

* * **** ** * **** * * * *

*msg*104 CTTCAAAGCATTAGAAGAACTATGCCAAGAAACGAAACTAGATGAAAAGT 699

*msg*76 CTTTAAAGCATTAGAAGAACTATGCCAAGAAATGAAATTAGATGAAAAGT 744

*msg*79 CTTCCGTGTATTAAAAGAACTATGCGAAGAAAAAAAACTAGATGAAAAGT 709

*msg*85 TTTCAAAGCATTAGAAGAACTATGCAAAAAAACGGAACTAGATAAAAAGT 717

*msg*7 CTTCGAAACATTAAAAGAACTATGCCAGGAAACAAAACTAGATGAAAATT 816

*msg*25 CTTTAATGTATTAAAAGAACTATGCCAAGAAACGGAACTAAATAAAAAGT 742

*msg*37 TTTTCTGGAATTAAGTGAACTATGTGAAGACACTAAATTAGATGGAAAAT 692

*msg*68 CTTCAAAGCATTAGAAGAACTATGCCAAGAAACGGAACTAGATGAAAAGT 1058

*msg*62 TTTTCCACAATTAAATAAACTATGTAAAGACAGTGACTTAAACAAAAAAT 692

*msg*20 GCATTATGTATTAAAAGAACAATGTAAAGAAATGAATATACAAAAAAAGT 537

*msg*3 TCATTTTAAATTGAACGAGTTATGTGAAAAATCGAAGATGAGTGAAAAAT 694

*** * *** * * * * *** *

*msg*104 GCAATGATTTAAAAGAAAATATTATGAGAAGATGTGCTGCACTTGAACGA 749

*msg*76 GCAATGATTTAAAAGAAAATATTATGGAAAGATGTACTGCACTTGAACGA 794

*msg*79 GCGATACTTTAAAAAAAACTATTACAGAAAAATGTACTATACTTAAACAA 759

*msg*85 GCAATGATTTAAAAGAAAATATTATGGGAAAATGTACTATACTTAAACAA 767

*msg*7 GCAATGATTTAAAAGAAAGTATTACAAAAAGATGTACTGCACTTGAACAA 866

*msg*25 GCAATGATTTAGAAGAAAATATTATGGAAAGATGTACTGCACTTGAACGA 792

*msg*37 GTACTCATTTGAAAGAACGACTTGCAAAACAATATGAAGCACTTAAAATG 742

*msg*68 GCAATGATTTAAAAAAAACTATTACAGAAAGATGTACTATACTTAAAAAA 1108

*msg*62 GTACTTATCTGAAAGAACAACTTGCAGGACAATATGAAGTACTTAAACAG 742

*msg*20 GTAATGATTTAAAAGTTATGATTGAAAAAACATGTACTACACATCAAACA 587

*msg*3 GTATTAATTTAGGAGAAAATATTAAGAAAAGTTGTGATATACTTAAACAT 744

* * * * * ** * * * ** * **

*msg*104 AGTTTGCAAGATATCCAGACTAAACCATCTCCAAGTACTTTTGAATGTCT 799

*msg*76 AGTTTGAAAGATATCTGGACTAAACCATCTCCAAGTACTTTTGAATGTCT 844

*msg*79 AGTTTAAAAAGTATCCGAAGTGAACCGTCCATAAGTTC---TTATTGTTT 806

*msg*85 AGTTTACAAGGTATCCGAAATAAACAGTCCATAAGTTC---TTATTGTTT 814

*msg*7 AGTTTGAAAGATATTCAAAATAAGCCGTTTACAAGTTTTTCTGATTGTGT 916

*msg*25 AGTTTGAAAGATGTTCTGACTAAGCTGTCTAAAACTGTTTTATATTGTCT 842

*msg*37 AATTTAAAAGATATAGAAACTGAATCATCACTAAACAGCCTAAAATGTAT 792

*msg*68 AGTTTACAAGGTATCCAAAATAAACCGTCTGTAAGTTCTTCAGATTGTCT 1158

*msg*62 AATTTAAAAAATATAGAAACTAAAACATCAATAAACAACGAGCAATGTAT 792

*msg*20 ATTTTGGAAAACGTATTGCAAGAATTGTCTCTTATGAATGAAGATTGCTT 637

*msg*3 GCTTTAACAGATGCTATAAAAAAACCATCAGCAACAAATGAAGATTGTGA 794

*** * * * * * **

*msg*104 GTATCAAAATGAATGTATGCGTATAGAAGAAGCATGTTCAATAAAAGTTA 849

*msg*76 GTATCAAAATGAATGTATGCGTATAGAAGAAGCATGTCCAATAAAAGTTA 894

*msg*79 GTATGAAAAAGAATGTATGCGTATAGAAGAAGCATGTCCAATAGAAGTTA 856

*msg*85 GTATGAAAAAGAATGTATGTTTGTGGAAGAAGTATGTCCAGCGGAAGTTA 864

*msg*7 ATATCAAAATGAATGTATGTTTGTGGAAGAAGCATGTTCAATAGAGGTTA 966

*msg*25 ATATCAAAGTGAATGTATGTTTGTGGAAGAAGCATGTTCAATGGAAGTTA 892

*msg*37 TATGCAATCTAAATGCATGTTCTTAGAGGGAGTTTATCCAAATGAACTTA 842

*msg*68 GTATCAAAAAGAATGTATGCTTATAGAAGAAGCATGTCCAACGGAAGTTA 1208

*msg*62 TATGCAGTCTGAATGTATGTTCTTGGAGGAAGTTTATCCGAATGAACTTA 842

*msg*20 ATTAGAAGATGACTGTATGTTTTTAGAGGGTGTATGTTCAGTAGAATTTA 687

*msg*3 AATGGAAACTGAATGTATGTTTTGGGAGGGAGTATCTCCAGAAGAACTTA 844

* * ** *** ** * * * * * * ***

*msg*104 ATGAAAAATGCAATTATTTAAGAGCATTTTGTAGAGAAAAAAGACGAGAT 899

*msg*76 ATAAAGAATGCAATTATTTAAGAACATTATGTAGAGAAAAAAGGCGAGAT 944

*msg*79 ATGAAGAATGCAATTATTTAAGAACATCATGTAGAAGAGAAAGAAGAGAT 906

*msg*85 AAGAAGAATGTAATTATTTAAGAACATTATGTGAAGAAAAAAAACGAGAT 914

*msg*7 AAAAAGGATGCAATTATTTAAGAATATTTTGTAGAGAAGAAAGACGAGAT 1016

*msg*25 AAGAAAAATGCAATTATTTAAGAATATTTTGTAGAGGAGAAAGACGAGAT 942

*msg*37 AAAAAAAATGTAATTATTTAAGAGTATTATGTAAAGAAGAAAAACAAGAC 892

*msg*68 AAGAAAGATGCAATTATTTAAGAGCATTTTGTAGAGAAAAAAGACGAGAT 1258

*msg*62 AAAAAGAATGTAATTATTTAAGAATATTATGTAAAGAAAAGAAACAAAAT 892

*msg*20 GAAAAAAATGCAGCGAGTTAAGAGAAAGATGTAGAATAAAAAAACAAGAT 737

*msg*3 CAGAAAAATGCAATAATTTAAGAATAATTTGTAGAAAAAGAAAAAGACAT 894

** *** * * ****** * *** * * * * *

*msg*104 GATTTGAAAACAGAATTTCTTTTAAGAGCCTTATCTGGAAATTTAAAAAC 949

*msg*76 GGTTTAAAAACAGAATTTCTTTTAAGAGCCTTATCTGGCAATTTGAAGAC 994

*msg*79 AATCTGAAAACAGAATTTCTTTTAAGAGTCTTATCTGGCAATTTGAAAAC 956

*msg*85 GATTTGAAAACAAAATTTCTTTTAAGAATCTTATCTGGTAATTTGAAAAC 964

*msg*7 GATCTGAAAACAAAATTTCTTTTAAGAGCCTTATCTGGCAATTTAAAAAC 1066

*msg*25 GATCTGAAAACAAAATTTCTTTTAAGGGCCTTATCTGGCAATTTAAAAAC 992

*msg*37 AGTTTGACAACAGAATTCCTTCTAAGAGTTTTTACTAACAATTTAAAAAC 942

*msg*68 GATCTGAAAACAAAATTTCTTTTAAGAGCCTTATCTGGCAGTTTGAAAAC 1308

*msg*62 AGTTTGACAATAGAATTCCTTCTAAGAGTTTTCACTAACAATTTAAGAAA 942

*msg*20 AATATAAAAACAGAAATTCTTTTAAGAGCATTAAGAGAAAATTTGAAAAG 787

*msg*3 AATTTAACAAAAAAATTTCTTTTAAGAGTATTTAATGAAAATATAAAAAC 944

* * * ** * ** * *** **** ** * * * * *

*msg*104 TCAAGAAGAGTGTGAGAAAATAATCGACAAAAAGTGTCTTGCGTTCATGG 999

*msg*76 TGAAGAAGACTGTGAAAAAATAATTGACAAAAAATGTCTTGCATTCATGG 1044

*msg*79 TCAAGAAGAGTGTGAGAAAATAATTGACAAAAAATGTCTTGCATTCATGG 1006

*msg*85 TAAAGAAGAATGTGAGAAAATAATTGATAAAAAATGTCTTCTGTTCATGA 1014

*msg*7 TAAAGAAGAGTGCAAGAAAATAATTGACAAAAAATGTCTTGCGTTCATGG 1116

*msg*25 TAAAGAAGAGTGTGAGAAAATAATTGACAAAAAATGTCTTGCGTTCATGG 1042

*msg*37 TGAAGGAGACTGTGAAAAAATAATTGATAAGAAATGTCTTATATTTATGG 992

*msg*68 TAAAGAAGAGTGTGAGAAAATAATTGACAAAAAATGTCTTCTGTTCATGG 1358

*msg*62 TGAAGAAGACTGTGAGAAAATAATTGATAAGAAATGTCTTATATTTATGG 992

*msg*20 TAAAGAAGCCTGCGAAAAAGTTATTAATGAAAAATGCTTTTTATTGATGA 837

*msg*3 AGTAGAAACGTGTAAACAAGTTATTAATGAGAAATGTCATGTATTCATGA 994

** * ** * ** * ** * * ** ** * ** ***

*msg*104 GAGAAAGCGATGAACTGATGAAGTTTTGTTTAATCCCCTTAAATAGATGT 1049

*msg*76 GAGAAAGCGATGAATTGATGAAGTTTTGTTTAATTCCTTTTAATAGGTGT 1094

*msg*79 GAGAAAGTGATGAATTGATGGAGTTTTGTTTAATTCCTTTTAATAGGTGT 1056

*msg*85 GAGAAAGCGATGAATTAATGAAATTTTGTTTAATTCCCTTTAATAGATGT 1064

*msg*7 GAGAAAGCGATGAATTGATGAAGTTTTGTTTAATTCCCTTAAATAGATGT 1166

*msg*25 AAGAAAGCGATGAATTGATGAACTTTTGTTTAATTCCCTTTAATAAATGT 1092

*msg*37 AAGAAAGTGATAAACTAATGAAATTTTGTCTTACTCCCTCGAATAGATGT 1042

*msg*68 GAGAAAGTGATGAATTAATGAAATTTTGTCTGACTCCCTTAAATAGATGT 1408

*msg*62 AAGAAAGTGATAAACTAATGAAATTTTGTCTGACTCCTTTAAATAGATGT 1042

*msg*20 GAGAAAGTGATGAACTTATGTCTTTTTGTTTGACAGAATTAGATAAGTGC 887

*msg*3 AAGAAAGCGATGAATTAATGCAGTTTTGCTTAAGTCCTTTAAATAGATGC 1044

****** *** ** * *** ***** * * * *** **

*msg*104 AACGAACTCGTGAAATTAATGGAGACAAAATGTAATAGTTTAGAATTTGA 1099

*msg*76 AACGAACTTGTAAAATTAATAAAAGGAAAGTGTAGTGATTTAGAATTTAA 1144

*msg*79 AACGAACTTGTAAAATTAATAAAAGAAAAGTGTAGTGATTTAGAATTTAA 1106

*msg*85 GATGAGCTCATGAAATTAATGAGGGAAAAATGTATTAGTTTACAATTTCA 1114

*msg*7 GACGAACTCATGAAATCGATCAGAAGCAAATGCAGTGGTTTACAATTTCG 1216

*msg*25 GATGAACTCATAAAATTAATGAAGAGAGAATGCAGTAGTTTGCAACTTAG 1142

*msg*37 AAGGATCTCATAAAATTAATGGAAAAAAAATGCAATAATTTAGAACTTGA 1092

*msg*68 GAAGACCTCATAAAATTAATGGAAGAAAAGTGTAGTAGTTTACAACTTAA 1458

*msg*62 AAGGACCTCATAAAATCGATAGAAAGTAAATGCAGTGATTTGAAATTTAA 1092

*msg*20 GGAAAACTTGTAGATGCAATGAGGAAGCAATGTAGTAGATTGAATCGTGA 937

*msg*3 AGGGACCTTATACATTTTATGAAAGATGAATACACCGAATTAAAAATAAA 1094

* ** * * ** * * * ** *

*msg*104 GGCAAAACTTTTTATCGAAAAGACTAAAATATCAAAAGAAGAATGCATTT 1149

*msg*76 AACAAAAATTTTTATCCAAAGCAATAAAATATCAAAAGAAGAATGCACTT 1194

*msg*79 AACAAAAATTTTTATCGAAAACAATAAAATATCAAAAGAAGAATGCACTT 1156

*msg*85 TTTAAAGAAATTCCCTGGGAATACTGAAATATCAAAAGAAGAATGTAATT 1164

*msg*7 TCTAGAAAAATTCACAGAGAACACTAAAATATCAAAAGAAGAATGTAATT 1266

*msg*25 TCTAAAAAAATTACTTGGGAATACTAAAATATCAAAAGAAGAATGTAATT 1192

*msg*37 GGCACAAATTTTTATCGAAAATACTAAAATATCAAAAGAAAAATGTAATT 1142

*msg*68 TGTAAAAGAATTCCCTGGGAATACTAAAATATCAAAAGAAAAATGTAATT 1508

*msg*62 TATAAATTTATTCACTCAAGATAATAAAATATCAAAAGAAAAATGTAATT 1142

*msg*20 TATAGAATCACTGCTCTATGATTTTAATGTATATAAAACGCAATGTGATT 987

*msg*3 TATAGAACAGTTTTTTAAAAATCCTAAAATATCAGAAGAAAAATGTAATT 1144

* * * * * *** ** **** **

*msg*104 CATTATTTGAGAAATGCTATTTTCATAAATCAAATTGCAATAACACTCTT 1199

*msg*76 CATTGTTTGAGGAATGCAACTTTCATGAATCAGATTGCGATGGTATTC-- 1242

*msg*79 CATTATTTGAGGAATGCAACTTTCATGAATCAGATTGCGATGGTATT--- 1203

*msg*85 TGTTACTTGAGGAATGTTACTTTCATAACTCAAATTGCAATAACACTCTT 1214

*msg*7 TATTACTTGAGGAATGTTACTTTCATAAATCAAATTGTAATAGTATT--- 1313

*msg*25 TGTTGCTTGAAGAATGTTACTTTCATAACTCAAATTGCAATAACACTCTT 1242

*msg*37 CATTATTTGAGGAATGCTACTTTCATAAATCAAATTGCAATAATACTCTT 1192

*msg*68 TGTTACTTGAGGAATGTTATTTTCATAACTCAAATTGCAATAACGCTCTT 1558

*msg*62 TATTACTCGATGAATGTTACTTTCATAAATCAAATTGTGATAACACTCTT 1192

*msg*20 TACTACTTGAAAAATGTTATTTTTATATACTAAACTGTGATA---T---T 1031

*msg*3 TTTTGCTTGATGAGTGTTACTTTTATGGATCAAATTATAATAATAATCTT 1194

* * ** * ** * *** ** * * * **

*msg*104 GATAATAAATGTAGAGAAATAGAAAAAAAGTGTGAAAATGAAGTAGAATA 1249

*msg*76 -AAAATATGTGTAAAGAAATAAAAAAAAGATGTGAGAATAAAGTAAAATA 1291

*msg*79 CAAAATATGTGTAAAGAAATAAAAAAAAGATGTGAGAATAAAGTAAAATA 1253

*msg*85 AATGATAAATGTACAGAAATAGAAAAAACATGTGAAAAAGAAATAAAATA 1264

*msg*7 CAAAATATGTGTAAAGAAATAGAAAAAAACTGTGAAAATAAAGTAGAATA 1363

*msg*25 AATGATGAATGTAGAGAAATAGAAAAAAAATGTGAAAATGAAGTAAAATA 1292

*msg*37 AATAATAAATGTAAAGAAATAGAAAAAAAGTGTGAAAATGAAGTAGAATA 1242

*msg*68 AATGATAAATGTAGAGAAATAGAAAGAACATGTGAAAAAGAAGTAAAATA 1608

*msg*62 CATGATAAATGTAGAGAAATAGAAAAAATGTGTGAAAAGGAAGTAAAATA 1242

*msg*20 CAAAATATGTGCAAAAAGTTAAAGGAGAAATGCGGAAA---AATAGAATA 1078

*msg*3 CAAGATATATGCAAAGAACTAGAAAAAAAATGTAAAAAAAAAGTGAAATA 1244

* ** ** * * * ** * * ** ** * * ****

*msg*104 CAAATATTTTTCTTTACCATTTAATCCAGTAGGAAAAGAAATTATATTAA 1299

*msg*76 CGAAGATTTTTCTTTACCATTTAACCCAATAGGAAAAGAAATTGTATTAA 1341

*msg*79 CGAAGATTTTTCTTTACCATTTAACCCAATAGGAAAAGAAATTATATTAA 1303

*msg*85 CAAACATTCTTCTTTACCATTTAATCCAATAGGAAAAGAAATTATATTAA 1314

*msg*7 CAAACATTTTTCTTTATCATTTAACCCAATAGGAAAAGAAATTATATTAA 1413

*msg*25 CAAACATTTTCCTTTACCATTTAACCCAATAGGAAAAGAAATTGTATTAA 1342

*msg*37 CAAACATTTTTCTTTACCATTTAACCCAATAGGAAAAGAAATTGTATTAA 1292

*msg*68 CAGACATTCTTCTTTACCATTTAATCCAATAGGAAAAGAAATTATATTAA 1658

*msg*62 CACACCACCTAATCTAATATTTAATCCAATAGAAAAAAAAATTACACTAA 1292

*msg*20 TATATCACTAGATCTAACTTTTGACCCATTGGGAAAAAATCTTGCACTAA 1128

*msg*3 CATACCACCAAGTTCAACCTTTAATCCAATAGGAAAAGATTTTACATTGA 1294

* * * *** * *** * * **** * ** * * *

*msg*104 TAGAAAAAGTTGGAAAAGAAAAAATATTCAAAGACGAAGTTGGAAAACCA 1349

*msg*76 TAGAAAAAGTTGGAAAAGAAAAATTATTCAAAGATGAAGTTGGAAAACCA 1391

*msg*79 TAGAAAAAGTTGGAAAAGAAAAATTATTCAAAGATGAAGTTGGAAAACCA 1353

*msg*85 TAGAAAAAGTTGGAAAAGAAAAAATATTCAAAGACGAAATTAGAAAACCA 1364

*msg*7 TAGAAAAAGTTCAAAAAGAAAAAATATTCAAAGACGAAGTTGGGAAACCA 1463

*msg*25 TAGAAAAAATTGGGAAGGAAAAATTATTCAAAGACGAAATTGGAAAACCA 1392

*msg*37 TAGAAAAAGTTGGAAAAGAAAAATTATTCAAAGATGAAATTGGAAAACCA 1342

*msg*68 TAGAAAAAGTTGGAAAAGAAAAATTATTCAAAGACGAAATTGGAAAACCG 1708

*msg*62 TAGAGAAAGTAAGAAAAGAGAAATTATTTGGAGATGAAATTAGAAAATCA 1342

*msg*20 TAGAAAAGCTAGGAAATTTAGAACTTTTTGGCGGCGAATTTAAAAGACCA 1178

*msg*3 TGGAAAAAGTAAAAAAAAAGAAATTATTTAGAGATGAAATTGGAAAGCCG 1344

* ** ** * ** ** * ** * *** ** * *

*msg*104 GGGATAAAAGATACAATTGATCTTTTAGTATTATTATCAAATAATTATTT 1399

*msg*76 GGGATAAAAGATACAATTGATCTTTTAGTATTATTATCAAATAATTATTT 1441

*msg*79 GGGATAAGGGATACAATCGATCTTTTAGTATTGTTATCAAATAATTATTT 1403

*msg*85 GGGATAAAAGATACGATTGATCTTTTAGTATTATTATCAAATAATTATTT 1414

*msg*7 GGGATAAAAGATACAATTGATCTTTTAGTATTATTATCAAATAATTATTT 1513

*msg*25 GGGATAAAAGATACAATTGATCTTTTAATATTATTATCAAATGGTTATTT 1442

*msg*37 GGAATAAAGGATACAATTGATCTTTTAGTATTATTATCAAATAATTATTT 1392

*msg*68 GGGATAAAAGATACGATTGATCTTTTAGTATTATTATCAAATAATTATTT 1758

*msg*62 GGAATAAAAGATACAATTGATCTTTTAGTATTATTAGTAGATAATTATTT 1392

*msg*20 GAAATAAAGGATACAGTTGATCTTTTAATAATGCTAACAAATGATGATAT 1228

*msg*3 AGAATGAAAGATATGATCGACCTTTTAGTATTAATGGTAAACAATGATCT 1394

** * **** * ** ****** ** * * * * * ** *

*msg*104 AGATGATTGCAAAAATTATATTGAAAAGTGTCATAAACTTTGCAGCCTTT 1449

*msg*76 AGATAGTTGTGAAACTAATATTAAAAGTTGCCATAAACTTTGCAGCCTTT 1491

*msg*79 AGATCATTGCAAAACTAATATTGAAAGTTGTCATAAACTTTGTAGCCTTT 1453

*msg*85 AGATAATTGCAAAACTGATATTGAAAGTTGTCATAAACTTTGTAGCCTTT 1464

*msg*7 AGATGGTTGCGAAACTAATATTGAAAAGTGTCATCAACTTTGCAGTCTTT 1563

*msg*25 AAGTGATTGCGAAACTTATATTGAAGAGTGTCGTAAACTTTGCAGTCTTT 1492

*msg*37 ATATGGCTGCAAAACTAATATTGAAAGCTGTCATAAATTTTGCAGCCTTT 1442

*msg*68 AGATGATTGCAAAACTAATATTGAAAGATGTCATAAACTTTGTAGCCTTT 1808

*msg*62 AAGTGGTTGTAAAGAGAATCTTAAAATGTGTCATAAATTTTGTAGCACAT 1442

*msg*20 AACAAAATGTAAAGATCGTGTTGAAGGATGCTATAAATTTTGTAGTTTTC 1278

*msg*3 AGAAGACTGCAAAACTTATGTTGAAAGATGTTATGAATTTTGTAGCTCTC 1444

* ** ** * ** ** ** * ** **** **

*msg*104 TACCTCAATTAGAGGATTTATATGACAGTGCTAAGGAAAAAATGAATAAA 1499

*msg*76 TACCTCAATTAGAGGATTTATATAACGGTACTAAAGAAAAAATGAATAAA 1541

*msg*79 TACCTGAATTAGAGGATTTATATGACAACACTAAGAAGAAAATGGATGAA 1503

*msg*85 TACCTGAATTAGAGGATTTATATGACAACACTAAAAAGAAAATGAATGAA 1514

*msg*7 TACCTCAATTAGAGGATTTATATGACAACACTAAGAAGAAAATGGATGAA 1613

*msg*25 TACCTCAATTAGAGGATTTATATAACAATACTAAGAGAAAAATGAATAAA 1542

*msg*37 TACCTCAATTAGAGGATTTATATGACAGTACTAAAGAAAAAATGGATAAA 1492

*msg*68 TACCTCAATTAAAGGATTTATATGACGGTACTAAAGAAAAAATGAATAAA 1858

*msg*62 TACCTCAATTGAAAGATTTATATAACAGCACTAAGAAAAAAATGGATAAA 1492

*msg*20 TACCTCAACTAAAAGATTTATATAATAATATTACAAAAAAAATGAGTAAA 1328

*msg*3 TACCTCAACTAAAAAATTTATATGACAACACTAAAAAAAAAATAAGTGAA 1494

***** ** * * ******** * ** ***** * **

*msg*104 AGCAAAGAAGAAGTATGTGATACCTTAAAAGAAAAATTAAAGCCTAAATG 1549

*msg*76 AGCAA---AGAAATATGTAATACCTTAGAAAAAGAATTGAAATCTAAATG 1588

*msg*79 AGCAAAAAAGAAATATGCAATACCTTAAAAGAAAAATTTAAACCTAAATG 1553

*msg*85 AGCAAAAAAGAAATATGCAATGCCTTGGGAGAAAAATTTAAACCTAAATG 1564

*msg*7 AGCAAAAAAGAAATATGCAATACCTTAAAAGAAGAATTAAAACCTAAATG 1663

*msg*25 AGCAA---AGAAATATGTACTACCTTAAAAAAAAAATTGGAACCTAGATG 1589

*msg*37 AGCAAGGAAGAAATATGTAATACCTTAGAAGAAAAATTGAAACCTAAATG 1542

*msg*68 AGCAA---AAAAATATGTAATACCTTAAAAGAAGAATTGAAATCTAAGTG 1905

*msg*62 AGTAA---AGAAATATGTGATACTTTAGAAGAAAAATTAAAACCTAGATG 1539

*msg*20 -G--A---AGAAATGTGTACATCGCTAAAAGATAAACTGAGGACTAAATG 1372

*msg*3 AATAAGGAAGAAATATGTACTAACCTAAAAGACACTCTAAAACCTAGATG 1544

* * ** * ** * * * * *** **

*msg*104 TAGGTTTTTTAAATCAAAATTATATGATTTATCACTATCAAATACTAAAA 1599

*msg*76 CAGGTTTTTTAAATCAGAATTACATAACTTACTACTATCAAATACTAGCG 1638

*msg*79 CAGATTTCTTAAATCAAAATTATATGATTTATCACTATCAAATACTAGCG 1603

*msg*85 CAAGCTTCTTAAATCAAAATTATATGATTTATCACTATCAGATACGA--- 1611

*msg*7 CAGGTCTTTTAAATCAAAATTATATGATTTATCACTATCAGATACGA--- 1710

*msg*25 CAAGTTTTTTAAATCAAAATTATATGATTTATCGCTATCAGATACTAACG 1639

*msg*37 TAGGTTTTTTAAATCAAAATTATATAATTTGTCACTATCAAATACTAAAG 1592

*msg*68 CAAGTTTTTTAAATCAAAATTATATGATTTGTCACTATCAAATACTAA-- 1953

*msg*62 TAGGAATTTTAAATCAAAATTACATAACTTGTTACTATCAGATACTAATG 1589

*msg*20 TAAAGCTTTTAAATTAAAATTAACTAGCATGTCACTATCAAATACTAGTA 1422

*msg*3 CAAAGCTTTAAAATTAGAATTGTACAGCTTATCATTATCAGACACAACCG 1594

* * * **** * **** * ***** * ** *

*msg*104 AAGACAATGAAGATGCTACTCTTATAAAATGGACCAAGCAATCTACTGAG 1649

*msg*76 AAGACAATGAAGATTCTATGCTTATAGAATGGACTAGACAATCTACTGAG 1688

*msg*79 AAGACAATGAAGATTCTATGCTTATAGAATGGACTAGACAATCTACTGAG 1653

*msg*85 AAGACGATAAAGATGCTATGCTTATGGAATGGACCAAGCAATCTATTAAC 1661

*msg*7 AAGACGATAAAGATGCTACTCTTATAAAATGGACGAAGCAATCTACAGAG 1760

*msg*25 AAGACAATAAAGATGCTAAGCTTATAGGATGGACCAAGCAATCTATTAAC 1689

*msg*37 AAGATAATGAAGATGCTACGCTTATAGAATGGACCAAGCAATCTACTAAG 1642

*msg*68 -AGACAATGAAGATGCTGCTCTTATAGAATGGACCAGGCAATCTATTGAG 2002

*msg*62 ATGATAATAAAGATGCTAAACTTCTAGAATGGAACAAACAGTTTGCTGAA 1639

*msg*20 AAGATGATGAAGAAGTCGAATTACTAGGATCCTTTGAACAGTTTCCAGAA 1472

*msg*3 ACGATAATAAAGAAACCACACTACTGAAATGGACTGAACAATTTACAGAA 1644

** ** **** * * ** ** * * *

*msg*104 TTTAATGAAAAGCTTTGTATAAATTTAGAATCAAAATGTTTTTATTTAAG 1699

*msg*76 TTTAATGAAGAGCTTTGTATAGATTTAGAATCAAAATGTTTTTATTTAAA 1738

*msg*79 TTTAATGAAAAGCTTTGTATAGATTTAGAATCAAAATGTTTTTATTTAAA 1703

*msg*85 TTTAATGAAAAGCTTTGTATAGATTTAGGATCAAAATGTTTTTATTTAAA 1711

*msg*7 TTTAATGAAAAGCTTTGTATAAATTTAGAATCAAAATGTTTTTATTTAAG 1810

*msg*25 TTTAATGAAAAACTTTGTATAGATTTAGAATCAAAATGTTTTTATTTAAG 1739

*msg*37 TTTAATGAAAAGCTTTGTATAGATTTAGAATCAAAATGTTTTTATTTAAA 1692

*msg*68 TTTAATGAAAAGCTTTGTATAGATTTAGAATCAAAATGTTTTTATTTAAA 2052

*msg*62 GTTGATGAGAAGCTTTGTGCGAATTTAGAATCAAAGTGCTTTTATTTAGA 1689

*msg*20 CTGAATAAAAGGCTTTGCACAAATTTAGAATCCAAATGTTTTTATTTCCA 1522

*msg*3 TTTGATGAAGAGCTTTGTACAGAGTTAGAATCAAAATGTTTTTATTATAG 1694

* ** * ***** * **** *** ** ** *******

*msg*104 GAAATCTTGTAATAATGCGGGTATTAAAATGTCTAATGCATGTATCAATG 1749

*msg*76 AAAACCTTGTAGTGATGAGGGTATTAAAATGTCTAATACATGCGTCAATG 1788

*msg*79 AAAACCTTGTAGTGATGAGGGTATTAAAATGTCTAATGCATGCGTCAATG 1753

*msg*85 AAAACCTTGTAATGACAAGGATATTAAAATGTCTAATGCATGCGTTAATT 1761

*msg*7 GAAATCTTGTAATGATGCGGATATTAAAATGTCTAATGCATGTATCAATG 1860

*msg*25 GAAATCTTGTAATAATCTGGGTATTAAAATGTTTAATGCATGCATCAATG 1789

*msg*37 GAAACCTTGTAATGACGCGGGTATTAAAATGTTTGATGCATGCATCAATT 1742

*msg*68 AAAACCTTGTGGTGATGAGGGCATTAAAATGTCTAATGCATGCATCAATT 2102

*msg*62 AAGACCCTGTAATAGTATAAATATTAAAATGACTAATGCATGTATCAATG 1739

*msg*20 AAAGCCTTGTACTTCAGAGAATATTAATCTTAATGGTGCATGTAGCAATG 1572

*msg*3 GAGACCTTGTAATTTAGAAAATATTAAGCTTAACAATGCATGTGGTAATG 1744

* * *** * ***** * * **** ***

*msg*104 TAGAATCAACATGTTTAAAAACACGACTTTTCAGAAGAGAATATCAACTA 1799

*msg*76 TAGAATCAACATGTTTAAAAACACGACTTTTCAGAAGAGAATATCAACTA 1838

*msg*79 TAGAATCAACATGTTTAAAAACACGGCTTTTCAGAAGAGAATATCAACTA 1803

*msg*85 TAGAATCAACATGTTTAAAAACACGACTTTTCAGAAAAGAATATCAACTA 1811

*msg*7 TAGAATCAACATGTTTAAAAACACGACTTTTTAGAAGAGAATATCAACTA 1910

*msg*25 TAAAATCAACATGTTTAAAAACACGACTTTTCAGAAGAGAATATCAATTA 1839

*msg*37 TAGAATCAACATGTTTAAAAACACGGCTTTTCAAAAAAGAGTATCAACTA 1792

*msg*68 TAGAATCAACATGTTTAAAAACACGGCTTTTCAGAAAAGAGTATCAAGTA 2152

*msg*62 TAAGATCAACATGTTTAAAGGCATTACTTTTTAGAAGAGAGTATCGATTA 1789

*msg*20 TGAAGTTAGCATGTTTAAAGATGCGGGCTTTTAAAGAAGAATATTACCTA 1622

*msg*3 TGAATTCAGCATGCTTAAAAACACGACTTCTTAAAAGAGAATATCAACTA 1794

* * * **** ***** * * * * *** *** **

*msg*104 TTTCAGAGCACATTAAAAGGGAAACTGCATAATTTAAC---A---AAT-- 1841

*msg*76 TTTCAGGGTACATTAAAAGGAAAATTGCATAATTTAAC---A---AAG-- 1880

*msg*79 TTTCAGAGCACATTAAAAGGAAAACTGCATAATTTAAC---A---AAT-- 1845

*msg*85 TTTCAGGGTAAATTAAGAGGAAAACTGCATAATTTAAC---A---AAT-- 1853

*msg*7 TTTCAGAGCACATTAAAAGGGAAACTGCATAATTTAAC---A---AAT-- 1952

*msg*25 TTTCAGAGCACATTAAAAGGAAAACTGCATAATTTAAC---A---AAT-- 1881

*msg*37 TTTCAAGGTGTATTAAAAGGAAAACTGCATGATTTAGA---A---AAT-- 1834

*msg*68 TTTCAGGGTATATTAAAAGGAAAACTGCATAATTTAAC---A---AAG-- 2194

*msg*62 TTTCAGGACACGTTAAAGGGAAAGTTACATAATTTAAT---AGTAAAT-- 1834

*msg*20 TTCGAAAATAACTTAAGAGGGAGACTGCACAATTTAAC---A---ATTAG 1666

*msg*3 CTTCAAAATAAATTAACAGGGAAACTTCATAATTTAATGATA---AAT-- 1839

* * **** ** * * ** ***** * *

*msg*104 -AATTCGCTTAAAACGTGCATAGATGAATTATGGATTCTATGTGAGAAAA 1890

*msg*76 -AATTCACTTAAAACGTGCATAGATGAACTATGGACTCTATGTGAGAAAA 1929

*msg*79 -AATTCGCTTAAAACGTGCATGGATGAATTATGGACTCTATGTAAGAAAA 1894

*msg*85 -AATTCGCTTAAAACGTGTATAGATGAATTATGGATTCTATGTAAAGAAA 1902

*msg*7 -AATTCGCTTAAAACGTGTATAGATGAATTATGGATTCTATGTAAAGAAA 2001

*msg*25 -AATTCGCTTAAAACGTGCATAGATGAACTATGGACTCTATGTAAAAAAA 1930

*msg*37 -AATTTACTTGAAATATGTGTAAATGAACTATGGATTCTATGTAAGAAAA 1883

*msg*68 -AATTCACTTAAAATGTGCATAGATGAACTATGGACTCTATGTAAGAAAA 2243

*msg*62 -AGTGTTCTTAAAAGATGCATAGATGAACTATTAGATTTATGCAAGAAAA 1883

*msg*20 CAGCTCGCTTAAAACATGTGTAAATGAACTATTGAATTTATGTAGAAAAG 1716

*msg*3 -GATGCTCTCAAAACATGTGTAAATGAACTGTTAAATCTATGTAAGGAAA 1888

** *** ** * ***** * * * **** **

*msg*104 TAATTAGCAGTAATAATCCTATATTAATAGATCTTTGTCTACACCCATGG 1940

*msg*76 TAATTAGCAGTAATAATCCTATATTAATAGATCTTTGTCTACACCCATGG 1979

*msg*79 TAATTAGCAATAATAATCCTGTATTAATGAATCTTTGTCTACACCCATGG 1944

*msg*85 TAATTAACAATGATAATGCCATATTAATGGGTCTTTGTCTACAACCATGG 1952

*msg*7 TAATTAACAATGATAATGCCATATTAATGGGTCTTTGTCTACAACCATGG 2051

*msg*25 TAATTAGCAATGATAATCCTATATTAATGGATCTTTGTCTACACCCATGG 1980

*msg*37 TAATTAACAATAATAATCCTATATTAATGGATCTTTGTCTACACCCATGG 1933

*msg*68 TAATTAACAATGATAATGCCATATTGATGAATCTTTGTCTACACCCATGG 2293

*msg*62 TAATTGATAAAAATAATATTATATCAATGAGTTTTTGTTTACAACCACAG 1933

*msg*20 GTACAGGTATTAAGGAACCTATATTTGCAGATTTATGCTTACAACCATGG 1766

*msg*3 ATGTTGACATTAAAGAACCACTTTTAGCAGATTTATGCTTGCATCCAAAA 1938

* * * * * * * ** * ** ***

*msg*104 GATACTTGTAAGGAGCTTGCAAATGATATCGAAAGGCAGAGTAGGTGGCT 1990

*msg*76 GATACTTGTAAGGAGCTTGCAAATGATATCGAAAGGCAGAGTAGGTGGCT 2029

*msg*79 GATACTTGTAAGGAACTTGCAAATGATATCGAAAGGCAGAGTAAGTGGCT 1994

*msg*85 GTTACTTGTAAAGAACTTGCAGATGATATCGAAAGGCAGAGTAAGTGGCT 2002

*msg*7 GTTACTTGTAAGGAACTTGCAGATGATATCGAAAGGCAGAGTAAGTGGCT 2101

*msg*25 GATACTTGTAAGGAACTTGCAAATGATATCGAAAGGCAGAGTAAGTGGCT 2030

*msg*37 GATACTTGTAAAGAACTTGCAAATGATATTGAAAGGCAGAGTAAGAGACT 1983

*msg*68 GATACTTGTAAGGAACTTGCAAATAATATCGAAAGGCAGAGTAAGTGGCT 2343

*msg*62 GACACTTGCCTTGCACTTGCAAATGATATTGAAAGATTAAATCATGAATT 1983

*msg*20 AACGCATGTCTAATACTTGCAAATGATATTGAAAAATTAAGTCGAGAATT 1816

*msg*3 AATACATGCCAGATGCTTGCAAAAGATATTGAAAAACAAAGTCAAGAATT 1988

* ** ****** * **** **** * * *

*msg*104 TAGAAACGATCTGGATTGGAAAAGGGATTTTCCAGATGAAGAGGACTGTA 2040

*msg*76 TAGAAACGATCTGGATTGGAAAAGGGATTTTCCAGATGAAGAGGACTGTA 2079

*msg*79 TAGAAACGATCTGGATCGGAAAAGGGATTTTCCAGATGAAGAAGACTGTA 2044

*msg*85 TAGAAACGATCTGGATCGGAAAAGGGATTTTCCAGATGAAGAAGACTGCA 2052

*msg*7 TAGAAACGATCTGGATCGGAAAAGGGATTTTCCAGATGAAGAGGACTGTA 2151

*msg*25 TAGAAACGATCTGGATCGGAAAAGGGATTTTCCAGATGAAGAAGACTGCA 2080

*msg*37 TAGAAACGATCTGGATTGGAAAAGGGATTTTCCAGATGAAGAGGACTGCA 2033

*msg*68 TAGAAACGATCTGGATCAGAAAAGGGATTTTCCGGATGAAGAAGACTGCA 2393

*msg*62 TAGGAAGGATTTAAATTGGAAAAGAGACTCACCAAATGAAGAAGACTGTA 2033

*msg*20 TAGAAAGGATTTAAATCAAAAAAGAGACTTTCCAAATGAAGAAGACTGTA 1866

*msg*3 AAATAAAAATCTAAATAAGAAAAAAGATTCTATAAATGAAGAAGATTGCA 2038

* ** ** * ** **** ** * ******* ** ** *

*msg*104 AAAAACTAAAAGAGAAATGTGAAGTGTTAGGACATGATTCAAAAACGAAC 2090

*msg*76 AAAAACTAAAAGAGAAATGTGAAGTGTTAGGACATGATTCAAAAACGAAC 2129

*msg*79 AAAAACTAAAAGAGAAATGTGAAGTGTTAGGACATGATTCAAAAACGAAC 2094

*msg*85 AAAAACTAAAAGAGAAATGTGAAGTGTTAGGACATGATTCAAAAATGAAC 2102

*msg*7 AAAAACTAAAAGAGAAATGTCAAGTGTTAGGACATGATTCAAAAACGAAC 2201

*msg*25 AAAAACTAAAAGAGAAATGTGAAGTGTTAGGACATGATTCAAAAATGAAC 2130

*msg*37 AAAAACTAAAAGAGAAATGTGAAATGTTAGGACATGATTCAAAAATGAAC 2083

*msg*68 AAAAACTAAAAGAGAAATGTGAAGTGTTAGGACATGATTCAAAAATGAAC 2443

*msg*62 AAATTTTAGAAGAGAAATGCAGAACACTAGGTCAAGATTCAAAAATAAAC 2083

*msg*20 GAAATCTAGAAGAGAATTGTAAGATACTAGGACAAGATTCAAAAATGAAT 1916

*msg*3 TGGAACTGGAAGAAAAATGCAAGATACTAGGACAAGATTCAAGAATAAAT 2088

* **** ** ** **** ** ******* ** **

*msg*104 GACTTACCATGTTTTACACTGAAAGGGCGATGTGATCATTTGGAAAATGC 2140

*msg*76 GACTTACCATGTTTTACACTGAAAGGGCGATGTGATCATTTGGAAAATGC 2179

*msg*79 GACTTACCATGTTTTACACTGAAAGGGCGATGTGATCATTTGGAAAATGC 2144

*msg*85 GACTTACCCTGTTTTACACTGAAAGAGCGATGTGATCATTTGGAAAATGC 2152

*msg*7 GACTTACCATGTTTTACACTGAAAGAGCGATGTGATCACTTAAAGAATGC 2251

*msg*25 GACTTACCATGTTTTACACTAAAAGAGCGATGTGATCACTTGAAGAATGC 2180

*msg*37 GACTTACCATGTTTTACACTGAAGGGGCGATGTGATCACTTAAAGAATGC 2133

*msg*68 GACTTACCATGTCTTACACTAAAAGAACGATGTGGTCATTTGAAAAGTGC 2493

*msg*62 GAACTACCATGTCTTACACTAAAAGAACGATGTGATCATTTGAAAAGTGC 2133

*msg*20 GAACTACCATGTCTTACACTAAAAGAACGATGTGATCACTTGAAAAATGC 1966

*msg*3 GAATTTTTATGTCTTGAACTAAACAAGAAGTGTGATCATTTAAAAAATAC 2138

** * *** ** *** ** **** *** ** * * * *

*msg*104 TAAGGAATTAGAGGAAATTTTGTTAGAAGAAAAAGTAGAAAATTTAGGCA 2190

*msg*76 TAAGGAATTAGAGGAAATTTTGTTAGAAGAAAAAGTAGAAAATTTAGGCA 2229

*msg*79 TAAGGAATTAGAGGAAATTTTGTTAGAAGAAAAAGTAGAAAATTTAGGCA 2194

*msg*85 TAAGGAATTAGAGGAAATTTTGTTAGAAGAAAAAGTAGAAAATTTAGGCA 2202

*msg*7 GAAAGAGTTAGAAGATATTTTATTAGAGGAAAAAGCAGAGAATTTAGGCA 2301

*msg*25 AAAAGAATTAGAAGATATTTTATTAGAGGAAAAAGCAGAAAATTTAGGCA 2230

*msg*37 AAAAGAATTGGAAGATATTTTATTAGAGGAAAAAGCAGAAAATTTAGGCG 2183

*msg*68 TAAGGAATTAGAAGAAATTTTATTAAAAGAAAATGTAGAAAAATTGGATG 2543

*msg*62 TAAGGAATTAGAGGAAATTTTATTAAAAGAAAATGTAGAAAAATTGGATG 2183

*msg*20 TAAGAAATTAGAGGAAATTTTGTTAGAAGAAAAAGCAGAAAATTTAGGCA 2016

*msg*3 AAAAGAATTAGAGGAAATTTTGATAAAAGAAAAAACAGAGAAATTGTATG 2188

** * ** ** ** ***** ** * ***** *** ** **

*msg*104 ATTTAGATATATGTATAAAAAAAGTTTCAGAAAAATGTAATAAATGGTCT 2240

*msg*76 ATTTAGATACATGTATAAAAAAAGTTTCAGAAAAATGTAATAAATGGTCT 2279

*msg*79 ATTTAGATACATGTATAAAAAAAGTTTCAGAAAAATGTAATAAATGGTCT 2244

*msg*85 ATTTAGATATATGTATAAAAAAAGTTTCAGAAAAATGTAATAAATGGTCT 2252

*msg*7 ATTTAGATATATGTATAGAAAGAGTTTCAGAAAAATGTAATAAATGGTCT 2351

*msg*25 ATTTAGATATATGTATAGAAAGAGTTTCAGAAAAATGTAATAGATGGTCT 2280

*msg*37 ATTTAAATACATGTATAAAAAGAGTTTCAGAAAAATGTAATAAATGGTCT 2233

*msg*68 ATTTAGATATATGTATAAAAAAAGTTTCAGAAAAATGTAATAAATGGTCT 2593

*msg*62 ACTTAGATATATGTATAAAAAGAGTTTCAGAAAAATGTAATAAATGGTCT 2233

*msg*20 ATTTAGATATATGTATAAAAAAAGTTACAGAAAGATGCAATAATTGGTCT 2066

*msg*3 ATTTACATACATGCATAAAAAAAATGACAGAAAGATGTAATAACTGGCCT 2238

* *** *** *** *** *** * * ****** *** **** *** **

*msg*104 AAAAAAAAGAGAACAAGATTTATTTTTTCGTGTATACAATTAGTCACTAC 2290

*msg*76 AAAAAAAAGAGAACAAGATTTATTTTTTCGTGTATACAATTAGTCACTAC 2329

*msg*79 AAAAAAAAGAGAACAAGATTTATTTTTTCGTGTATACAATTAGTCACTAC 2294

*msg*85 AAAAAAAAGAGAACAAGATTTATTTTTTCGTGTATACAATTAGTCACTAC 2302

*msg*7 AAAAAAAAGAGAACAAGATTTATTTTTTCGTGTATACAATTAGTCACTAC 2401

*msg*25 AAAAGAAAGAAAACAAGATTTATTTTTTCGTGTATACAATTGGTTACTAC 2330

*msg*37 AAAAAAAAGAAAACAAAGTTTATTATTTCATGTATACAATTGAATGCTAC 2283

*msg*68 AAAAGAAAGAGATCAGAATTTATTCTTTCATGTATACAATTAAATGCTAC 2643

*msg*62 AAAAGAAAGAGATCAGAATTTATTCTTTCATGTATACAATTAAATGCTAC 2283

*msg*20 AAAAGAAAGAGAACAGAGTTTATTCTTTCATGTATACAATTAAATGTTAC 2116

*msg*3 AAAAAAACGAAAACATTATTTACTATTTCATGTATACAAGTGAACATTAC 2288

**** ** ** * ** **** * **** ********* * ***

*msg*104 TTGTCAAATCATTACTAGAGACATTAAATCTAAATGTTCTGTATTAGAAA 2340

*msg*76 TTGTCAAATCATTACTAGAGACATTAAATCTAAATGTTCTGTATTAGAAA 2379

*msg*79 TTGTCAAATCATTACTAGAGACATTAAATCTAAATGTTCTATATTAGAAA 2344

*msg*85 TTGTCAAATCATTACTAGAGACATTAAATCTAAATGTTCTGTATTAGAAA 2352

*msg*7 TTGTCAAATCATTATTAGAGATATTAAATCTAAATGTTCTATATTAGAAA 2451

*msg*25 TTGCCAGATAATTACTAGAGACATTAAATCTAAATGTTCTGTATTAGAAA 2380

*msg*37 TTGTCGAATAATTATTGAAAGTATTAAATTTAAATGTACTGCATTAGGAA 2333

*msg*68 TTGTCGAATAATTATTAAAAGTATTAAATCTAAATGTACTATATTAGAAA 2693

*msg*62 TTGTCGAATAATTATTGAAAGTATTAAATCTAAATGTACTATATTAGAAA 2333

*msg*20 TTGTCAAATAATTATTGAAAGTATTAGATCTAAATGTACTGCATTAGAGA 2166

*msg*3 TTGCAGAATAATTATTGAAGACGTTAAATTTAAATGCAATACATTAAGAA 2338

*** ** **** * * *** ** ****** * **** *

*msg*104 GGAACATAGATATTGAAGACGTTTTAGATCAAGTGAAGAGTAATAATGCA 2390

*msg*76 GGAACATAGATATTGAAGACGTTCTAGATCAAGTGAAGAGTAATAATGCA 2429

*msg*79 GGAACATAGATATTGAAGACGTTCTAGATCAAATGAAGAATGATAATGCA 2394

*msg*85 GGAACATAGATATTGAAGACGTTTTAGATCAAGTGAAGAGTAATAATGCA 2402

*msg*7 GGAACATAGATATTGAAGACGTTCTAGATCAAGTGAAGAATGATAATGCA 2501

*msg*25 AAAACATAGATATTGAAGACGTTCTAAATCAAGTGAAGAGTGATAATGCA 2430

*msg*37 AAAACATAGAAATTGAAAACGTTTTAGATCAAGTGAAGAATAATGATGTA 2383

*msg*68 GAAACATAGATATTGAGGACGTTCTAGATCAAGTGAAGAATAATGATACA 2743

*msg*62 GAAACATAGATATTGAGGACGTTCTAGATCAAGTGAAGAATAATGATACA 2383

*msg*20 AAAACATAGAAATTGAAAACGTTTTAGATCAAGTAAAGAATGATGATGTA 2216

*msg*3 AAAA---------------------------------------------- 2342

**

*msg*104 GACATAAAGGGCCCTACATGTGATTTATGGGAGCCTTATTGTGATAAGTT 2440

*msg*76 GACATAAAAGGCCCTACATGTGATTTATGGGAACCCTATTGTGATAAGTT 2479

*msg*79 GACATAAAGGGCTCTACATGTGATTTATGGGAACCCTATTGTGATAAGTT 2444

*msg*85 GACATAAAGGGCCCTACATGTGATTTATGGGAGCCTTATTGTGATAAGTT 2452

*msg*7 GACATAAAGGGCCCTACATGTGATCTATGGGAACCCTATTGTAATAAGTT 2551

*msg*25 GACATAAAGGGCTCTACATGTGATTTATGGGAACCCTATTGTGATAGGTT 2480

*msg*37 AACATGAAAAGCCCTATATGTGATTTATGGGAACCCTATTGTGATAAATT 2433

*msg*68 AATATAAAAGGAGAAGCATGCGACTTTTGGGAACCTTATTGCGATAAGCT 2793

*msg*62 AATATAAAAGGAGAAGCATGCGACTTTTGGGAACCTTATTGCGATAAGCT 2433

*msg*20 AATATAAAAGGACAAACATGCGACTTTTGGGAACCTTATTGTGACAAGTT 2266

*msg*3 -------------------------------------------------- 2342

*msg*104 TATGTTGAGCTGTGAAAAACTTGTACGAAATAATGGAAAGAATGGAAAAT 2490

*msg*76 TATGTTGAGCTGTGAAAAACTTGTACGAAATAATGGAAAGAATGGAAAAT 2529

*msg*79 TATGTTGAGCTGCGAAATACTTGTACAAAATAATGGAAAGAATGGAAAGT 2494

*msg*85 TATGTTGAGCTGCGAAATACTTGTACAAAATAATGGAAAGAATGGAAAAT 2502

*msg*7 TATGTTGAGCTGCGAAATACTTGTACAAAATAATGGAAAGAATGGAAAGT 2601

*msg*25 TATGTTGAGCTGTGAAAAACTTGTACGAAATAATGGAAAGAATGGAAAAT 2530

*msg*37 TACGTTGAGCTGTGAAAAACTTGCACAAAATAATGGAAAGAATGGAAAAT 2483

*msg*68 TCTGCTGAACTGTGAGAAACTTGTTAAAGATAATGGGAACGATGGAAAAT 2843

*msg*62 TCTGCTGAACTGTGAAAAACTTGTTCAAGATAATGGGAACGATGGAAAAT 2483

*msg*20 TCAGCTAAACTGTGAAAAACTTGTTCAAGATAATGAGAATGATGGAAAAT 2316

*msg*3 -------------------------------------------------- 2342

*msg*104 GCAAAGAACTGAAGGAGAGCTGTAAATCGTATCGTAAAACACAAGAACAA 2540

*msg*76 GCAAAGAACTAAAAGAAAGCTGTAAATCGTATCGTGAAATACAAGAACAA 2579

*msg*79 GCAAAGAACTAAAGGAGAGCTGTGAACCCTATCGTAGAATACAAGAACAA 2544

*msg*85 GCAAAGAACTAAAAGAGAGCTGTAAATCGTATCGTGAAATACAAGAACAA 2552

*msg*7 GCAAAGAACTAAAGGAGAGCTGTGAACCCTATCGTAGAATACAAGAACAA 2651

*msg*25 GCAAAGAACTGAAGGAGAGCTGTAAATCGTATCGTAAAACACAAGAACAA 2580

*msg*37 GCAAAGAACTAAAAGAGAGCTGTAAATCGTATCGTGAAATACAAGAACAA 2533

*msg*68 GTATAAAATTAAAAAAGAGCTGTATATTATACCGAACAGAAGAAAATCAA 2893

*msg*62 GTATAAAATTAAAAAAGAGCTGTATATTATACCGAACAGAAGAAAATCAA 2533

*msg*20 GTATAGAATTAAAAAAGAGCTGTAAATTACACAGAATGGGGGAAAATCAA 2366

*msg*3 -------------------------------------------------- 2342

*msg*104 GAAATGAAACTTATGTATGAATTAAGAGGAAGTCTCAATAGCGAAAATAA 2590

*msg*76 GAAGTGAGACTTATCTATGAATTAAGAGGAAGTCTTAATGACAAAAATAA 2629

*msg*79 GAAATGAAACTTATGTATGAATTAAGAGGAGGTCTCAATAGCGAAAATAA 2594

*msg*85 GAAGTGAGACTTATGTATGAATTAAGAGGAAGTCTCAATAGCGAAAATAA 2602

*msg*7 GAAATGAAACTTATGTATGAATTAAGAGGAGGTCTCAATAGCGAAAATAA 2701

*msg*25 GAAATGAAACTTATGTATGAATTAAGAGGAAGTCTCAATAGCGAAAATAA 2630

*msg*37 GAAGTGAGACTTATCTATGAATTAAGAGGAAGTCTCAATAGCGAAAATAA 2583

*msg*68 AAGATGGAACTTATGTATGAATTAAGAGGAAGTCTCGATAACAAAAATAA 2943

*msg*62 AAGATGGAACTTATGTATGAATTAAGAGGAAGTCTCGATAACAAAAATAA 2583

*msg*20 AAGATGGAACTTATGTATGAATTAAGAGGAAGTCTCGATAACAAAAATAA 2416

*msg*3 ---------------TATGAAT---------------------------- 2349

*******

*msg*104 ATGTGAATCAACCCTCAATAAACACTGTTTGCATTGGAATAAAACGAAAA 2640

*msg*76 ATGTGAATCAACTCTCAATAAACACTGTTTGCATTGGGATAAAACGAAAA 2679

*msg*79 ATGTAAATCAACCCTTAATGAACACTGTTTGCATTGGGATAAAACGAAAA 2644

*msg*85 ATGTGAATCAACTCTCAATAAACGCTGTTTGCATTGGGATAAAACGAAAA 2652

*msg*7 ATGTAAATCAACCCTTAATGAACACTGTTTGCATTGGGATAAAACGAAAA 2751

*msg*25 ATGTGAATCAACCCTCAATAAACACTGTTTGCATTGGAATAAAACGAAAA 2680

*msg*37 ATGTAAATCAACCTTTAATGAACACTGTTTGCATTGGGATAAAACGAAAA 2633

*msg*68 ATGTAAAACATCTCTTGATAGATTTTGTTTGTTCTGGGATGAAACAAAAA 2993

*msg*62 ATGTAAAACATCTCTTGATAGATTTTGTTTGTTCTGGGATGAAACAAAAA 2633

*msg*20 ATGTAAAACATCTCTTGATAGATTTTGTTTGTTCTGGGATGAAACAAAAA 2466

*msg*3 -----------------------AT---------T--------ATGAA-- 2357

* * **

*msg*104 ATAATACATTCAATAATTTCTGTAATAATAACACTGATATCAAAAATAAT 2690

*msg*76 ATGATACATTCAAGAATTTCTGCAACAATAACACTGATACCAAAAATAAT 2729

*msg*79 ATGATACATTCAAGAATTTCTGCAACAATAACACTGATACCAAAAATAAT 2694

*msg*85 ATGATACATTCAAGAATTTCTGCAACAATAACACTGATACCAAAAATAAT 2702

*msg*7 ATGATACATTCAAGAATTTCTGCAACAATAACACTGATACCAAAAATAAT 2801

*msg*25 ATAATACATTCAATAATTTCTGTAATAATAACACTGATATCAAAAATAAT 2730

*msg*37 ATGATACATTCAAGAATTTCTGTAATAATAACATTGATACCAAAAATAAT 2683

*msg*68 ATAATACATTTAAGAATCTCTGTCATGATGACAATGGTACTAAAAATGAT 3043

*msg*62 ATAATACATTTAAGAATCTCTGTCATGATGACAATGGTACTAGAAATGAT 2683

*msg*20 ATAATACATTTAAGAATTTCTGTCATGATGACAATGGTACTAAAAATGAT 2516

*msg*3 --------------AATTTCAGA------------------AAAAATAA- 2374

*** ** * * **** *

*msg*104 ATAACTAAAAATGAACTGTGCAGAAAATTATTAAAACGTCTAAAAGAAAG 2740

*msg*76 ACAACTAAAAATGAACTGTGCAAGAAATTACTAAAACATGTAAAGGAAAG 2779

*msg*79 ACAACTAAAAATGAACTGTGCAAGAAACTACTAAAACATGTAAAGGAAAG 2744

*msg*85 ACAACTAAAAATGAACTGTGCAAGAAACTACTAAAACATGTAAAGGAAAG 2752

*msg*7 ACAACTAAAAATGAACTGTGCAAGAAATTACTAAAATATGTAAAGGAAAG 2851

*msg*25 ATAACTAAAAATGAACTGTGCAGAAAATTATTAAAACGTCTAAAAGAAAG 2780

*msg*37 ACAACTAAAAGTGAACTGTGCAAAAAATTATTAGAACGTGTAAAGGAAAG 2733

*msg*68 ACAGCTAGAGATGAACTATGTGTAAAGCTCGTAGATAAAATGAAAAAAAA 3093

*msg*62 ACAGCTAGAGATGAACTATGTGTAAAGCTCGTAGATAAAATGAAAAAAAA 2733

*msg*20 ACAGCTAGAGATGAACTATGTGTAAAGCTCGTAGATAAAATGAAAAAAAA 2566

*msg*3 --------AA----AC-----------------------------CAAAG 2383

* ** ***

*msg*104 ATGTACAAAATTATTTACGAAATTAAATGACACTGCAGTAGAAATAGAAA 2790

*msg*76 ATGTACAAAATTGTTGACAAAATTAAATGACATGGCAACAGAAATAGAAA 2829

*msg*79 ATGTACAAAATTATTGGCAAAATTAAATGGCATGGCAACGGAAATAGAAG 2794

*msg*85 ATGTACAAAATTATTGGCAAAATTAAATGGCATGGCAACGGAAATAGAAG 2802

*msg*7 ATGTACAAAATTATTGGCAAAATTAAATGGCATGGCAAGGGAAATAGAAG 2901

*msg*25 ATGTACAAAATTATTCACGAAATTAAATGACACTGCAGTAGAAATAGAAA 2830

*msg*37 ATGTACAAAATTGTTGACAAAATTAAATGACATGGCTACAGAAATAGAAA 2783

*msg*68 ATGTACAGAAATGTTCATAAGATTAAATGATTCAGCAACAGAGATAGAAA 3143

*msg*62 ATGCACAGAAATGTTCATAAGATTAAATGATTCAGCAACAGAGATAGAAA 2783

*msg*20 ATGTACAGAAATGTTCATAAGATTAAATGATTCAGCAATAGAGATAGAAA 2616

*msg*3 ATAT--------------------A-----------------AATATAAA 2396

** * *** **

*msg*104 AAAATGTAAAGATTGTTGAAGAATTGAACGAAGCTGCAAAAAAAGCACTA 2840

*msg*76 AAAGCATAAAAATTGTTGAAGAATTGAACGAAGCTGCGAAAAAAGCACTA 2879

*msg*79 AAAATGTAAAGATTGTTGAAAAATTGAACGAAGCAGCGAAAAAAGCACTA 2844

*msg*85 AAAATGTAAAGATTGTTGAAAAATTGAACGAAGCAGCGAAAAAAGCACTA 2852

*msg*7 AAAATGTAAAGATTGTTGAAAAATTGAACGAAGCAGCGAAAAAAGCACTA 2951

*msg*25 AAAGTGTAAAGATTGTTGAAGAATTGAACGAAGCTGCAAAAAAAGCACTA 2880

*msg*37 AAAACATAAAAATTGTTGAAGAATTGAACGAAGCTGCAAAAAAAGCACTA 2833

*msg*68 AAAGTCTGGATATTGCTGGAAAATTAATTCAAACAGCAAAAAAAGCATTA 3193

*msg*62 AAAGTATGGATATTGCTGGAGAATTAATTCAAACAGCAAAAAAAGCATTA 2833

*msg*20 AAAGTCTGGATATTGCTGGAAAATTAATTCAAACAGCAAAAAAAGCATTA 2666

*msg*3 -------------------------------------------------- 2396

*msg*104 AAGAATACAAATCTTATTTTAACTTCAAGCAAACAAAAAACAGATCCTAA 2890

*msg*76 AAGAATACAAATCTTATTTTAACTTCAAGCAAACAAAAAACAGATCCTAA 2929

*msg*79 AAGAATACAAATCTTATTTTAACTTCAAGCAAACAAAAAACAGATTCTAA 2894

*msg*85 AAGAATACAAATCTTATTTTAACTTCAAGCAAACAAAAAACAGATCCTAA 2902

*msg*7 AAGAATACAAATCTTATTTTAACTTCAAGCAAACAAAAAACAGATTCTAA 3001

*msg*25 AAGAATACAAATCTTATTTTAACTTCAAGCAAACAAAAAACAGATCCTAA 2930

*msg*37 AAGAATATAAATCTTATTTTAACTTCAAGCAAACAAAAAACAGGTTCTAA 2883

*msg*68 AGAAGTGTAAAGCTTACTTTAATTTCAAATAGACGAAGAACATGTCTTAA 3243

*msg*62 AGAAGTGTAAAGCTTACTTTAATTTCAAATAGACGAAGAACATGTCTTAA 2883

*msg*20 AGAAGTGTAAAGCTTACTTTAATTTCAAATAGACGAAGAACATGTCTTAA 2716

*msg*3 ------------------------------------AAAACA-------- 2402

* ****

*msg*104 CATAAATAATGCAACATTAATTCTAGCTTATAATGCAAATGCTGACAGGA 2940

*msg*76 CATAAATAACGCAACATTAATTCTAGCTTATAATGCAAATGCTGACAGAA 2979

*msg*79 CATAAATAACGCAACATTAATTCTAGCTTATAATGCAAATGCTGACAGGA 2944

*msg*85 CATAAATAACGCAACATTAATTCTAGCTTATAATGCAAATGCTGACAGAA 2952

*msg*7 CATAAATAACGCAACATTAATTCTAGCTTATAATGCAAATGCTGACAGAA 3051

*msg*25 CATAAATAACGCAACATTAATTCTAGCTTATAATGCAAATGCTGACAGAA 2980

*msg*37 CATAAATAACACAATATTAATTCTAGCTTATAATGCAAATGCTGACAGGA 2933

*msg*68 TATACATAA-----------TT-------------GGAATACTAAGACAA 3269

*msg*62 CATACATAA-----------TT-------------GGAATACTAAGACAA 2909

*msg*20 TATACATAA-----------TT-------------GGAATACTAAGACAA 2742

*msg*3 ----AATA-TGCG------------------------------------- 2410

***

*msg*104 ATGTAAATTTAAAAACAGATGTAACTCTGAAAAATCAATCAAGGTATTTG 2990

*msg*76 ATGTAAATTTAAAAACAGATGTAACTCTGAAAAATCAATCAAGGTATTTG 3029

*msg*79 ATGTAAATTTAAAAACAGATGCAACTCAGAAAAATCAATCAAGGTATTTG 2994

*msg*85 ATGTAAATTTAAAAACAGATGTAACTCTGAAAAATCAATCAAGGTATTTG 3002

*msg*7 ATGTAAATTTAAAAACAGATGTAACTCTGAAAAATCAATCAAGGTATTTG 3101

*msg*25 ATGTAAATTTAAAAACAGATGTAACTCAGAAAAATCAATCAAGGTATTTG 3030

*msg*37 ATGTAAATTTAAAAACAGATGTAACTCAGAAAAATCAATCAAAGTATTTG 2983

*msg*68 ATGCAAATTTAAAAAGAGATACAACTC---AAAATCAATTAGAGTATTTG 3316

*msg*62 ATGCAAATTTAAAAAGAGATACAACTC---AAAATCAATTAGAGTATTTG 2956

*msg*20 ATGCAAATTTAAAAAGAGATACAACTC---AAAATCAATTAGAGTATTTG 2789

*msg*3 -----------------------ACT-------------------TTTGG 2418

*** ** *

*msg*104 GAACAAAA-GGAGACAAAAGTGAATATTACAGAAAAAGAAGTTGAAGCGT 3039

*msg*76 GAACAAAA-GGAGACAAAAGTGAATATTACAAAAAAAGAAGTTGAAGCGT 3078

*msg*79 GAACAAAA-GGAGACAAAAGTAAATATTACAGAAAAAGAAGTTGAAGCGT 3043

*msg*85 GAACAAAA-GGAGACAAAAGTGAATATTACAGAAAAAGAAGTTGAAGCGT 3051

*msg*7 GAACAAAA-GGAGACAAAAGTGAATATTACAGAAAAAGAAGTTGAAGCGT 3150

*msg*25 GAACAAAA-GGAGACAAAAGTGAATATTACAGAAAAAGAAGTTGAAGTGT 3079

*msg*37 GAACAAAA-GGAGACAAAAGTGAATATTACAGAAAAAGAAGTTGAAGCGT 3032

*msg*68 GAACAAAA-GGAAATAGAAGTAAATATTACAGAAAAAGAAGTTGAGGCAT 3365

*msg*62 GAACAAAA-GGAGATAGAAGTGAATATTACAGAAAAAGAAGTTGAAGCGT 3005

*msg*20 GAACAAAA-GGAAATAGAAGTAAATATTACAGAAAAAGAAGTTGAGGCAT 2838

*msg*3 GAACCATATTGTGAT--AAGTTTATGTTA----------AGTTGTA---- 2452

**** * * * * **** ** *** *****

*msg*104 TTGATGCAGCAGCAGAAGCATTAAAGGTTTATACAGAAGTCAAAGCAGAG 3089

*msg*76 TTGATGCAGCAGCAGAAGCATTAAAGGTTTATACAGAAGTCAAAGCAGAG 3128

*msg*79 TTGATGCAGCAGCAGAAGCATTAAAGGTTTATACAGAAGTCAAAGCAGAG 3093

*msg*85 TTGATGCAGCAGCAGAAGCATTAAAGGTTTATACAGAAGTCAAAGCAGAG 3101

*msg*7 TTGATGCAGCAGCAGAAGCATTAAAGGTTTATACAGAAGTCAAAGCAGAG 3200

*msg*25 TTGATACAGCAGCAGAAGCATTAAAGGTTTATACAGAAGTCAAAGCAGAG 3129

*msg*37 TTAATGCAGCAGCAGAAGCATTAAAGGTTTATACAGAAGTCAAAGCAGAG 3082

*msg*68 TTGATGCAGCAACAGAAGCGTTAAAAGTCTATAGAGAAGTGGAAGAAGAG 3415

*msg*62 TTGATGCAGCAACAGAAGCGTTAAAAGTCTATAGAGAAGTGGAAGCAGAG 3055

*msg*20 TTGATGCAGCAACAGAAGCGTTAAAAGTCTATAGAGAAGTGGAAGAAGAG 2888

*msg*3 ---------------A-------AGGACTTATACAAAA-TG--------- 2470

* * **** * ** *

*msg*104 TGCAAAGGTCTGCAATTAGAATGTGAATTTAAGGAAGATTGTTCTGAATA 3139

*msg*76 TGCAAAGGTCTGCAGTTAGAATGTGAATTTAAGGAGGATTGTTCTGAATA 3178

*msg*79 TGCAAAGGTCTGCAGTTAGAATGTGAATTTAAGGAAGATTGTTCTGAATA 3143

*msg*85 TGCAAAGGTCTGCAATTAGAATGTGAATTTAAGGAAGATTGTTCTGAATA 3151

*msg*7 TGCAAAGGTCTGCAATTAGAATGTGAATTTAAGGAAGATTGTTCTGAATA 3250

*msg*25 TGCAAAGGTCTGCAGTTAGAATGTGAATTTAAGGAGGATTGTTCTGAATA 3179

*msg*37 TGCAAAGGTCTGCAATTAGAATGTGAATTTAAGGAAGATTGTTCTGAATA 3132

*msg*68 TGCAGAAGTCTACTGTTAAAATGTAAATTTAAGGAGGATTGTTCTGAACA 3465

*msg*62 TGCAGAAGTCTACTGTTAAAATGTAAATTTAAGGAGGATTGTTCTGAACA 3105

*msg*20 TGCAGAAGTCTACTGTTAAAATGTAAATTTAAGGAGGATTGTTCTGAACA 2938

*msg*3 -------------------------------------ATA---------G 2474

**

*msg*104 CAAAGATGTATGCAAGAAAATTGAAGATGCATGCAATAAATTAAAGTCAC 3189

*msg*76 CAAAAACGTATGCAAGAAAATTGAAGATGCATGCAATAAATTAAAGTCAC 3228

*msg*79 CAAAGATGTATGCAAGAAAATTGAAGATGCATGCAATAAATTAAAGTCAC 3193

*msg*85 CAAAGATGTATGCAAGAAAATTGAAGATGCATGCAATAAATTAAAGTCAC 3201

*msg*7 CAAAGATGTATGCAAGAAAATTGAAGATGCATGCAATAAATTAAAGTCAC 3300

*msg*25 CAAAAACGTATGCAAAAAAATTGAAGATGCATGCAATAAATTAAAGTCAC 3229

*msg*37 CAAAGATGTATGCAAGAAAATCGAAGATGCATGTAATAAATTAAAGTCAT 3182

*msg*68 TAAAAACACATGCAAGAAAATCGAAGAAACATGCAGTAAATTGGGGTCAC 3515

*msg*62 CAAAAACACATGCAAGAAAATCGAAGAAACATGCAGTAAATTGGGGTCAC 3155

*msg*20 CAAAAACACATGCAAGAAAATCGAAGAAACATGCAGTAAATTGGGGTCAT 2988

*msg*3 CAAAGATG-------GAAA-------ATG-----------------T--- 2490

*** * *** * *

*msg*104 TGGAAATCAAGTCTTCAGAAACAAAAACAATTAATC-------------- 3225

*msg*76 TGGAAATCAAGTCTTCAGAAACAAAAACAATTAATC-------------- 3264

*msg*79 TGGAAATCAAGTCTTCAGAAACAAAAACAATTAATC-------------- 3229

*msg*85 TGGAAATTAAGTCTTCAGAAACAAAAACAATTAATC-------------- 3237

*msg*7 TGGAAATCAAGTCTTCAGAAACAAAAACAATTAATC-------------- 3336

*msg*25 TGGAAATCAAGTCTTCAGAAACAAAAACAATTAATC-------------- 3265

*msg*37 TGGAAATCAAGTCTTTAGAAACAAAAACAATTAATC-------------- 3218

*msg*68 TGGGAGTCAGATCTTCAGAAACAAAAATCACTACAG-------------- 3551

*msg*62 TGGGAGTCAGATCTTCAGAAACAAAAATCACTGCAG-------------- 3191

*msg*20 TGGGAGTCAGATCTTCAGAAACAAAAATCACTACTGAAACAGATAAAAAT 3038

*msg*3 -G------AA-------GA------------------------------- 2495

* * **

*msg*104 ----------AAAC-------------------------------TATAA 3234

*msg*76 ----------AAAC-------------------------------TATAA 3273

*msg*79 ----------AAAC-------------------------------TATAA 3238

*msg*85 ----------AAAC-------------------------------TATAA 3246

*msg*7 ----------AAAC-------------------------------TATAA 3345

*msg*25 ----------AAAC-------------------------------TATAA 3274

*msg*37 ----------AAAC-------------------------------TATAA 3227

*msg*68 ----------AAA----CAAA----------------------------- 3558

*msg*62 ----------AAA----CAAA----------------------------- 3198

*msg*20 ACAACGCAAGAAACAGATAAAAACACAACACAAGAAACAGAAACAGATAA 3088

*msg*3 -------------------------------------------------- 2495

*msg*104 AAACAACAAT-----------------------CATTGCAG--------- 3252

*msg*76 AAAC---AAT-----------------------TATTACAA--------- 3288

*msg*79 AGAC---CAT-----------------------CATTACAA--------- 3253

*msg*85 AGAC---CAT-----------------------CATTACAA--------- 3261

*msg*7 AGAC---CAT-----------------------CATTACAA--------- 3360

*msg*25 AAAC---AAT-----------------------CATTACAA--------- 3289

*msg*37 AAAC---AAT-----------------------TGTTACAA--------- 3242

*msg*68 -A------AT-----------------------CACTACAG--------- 3569

*msg*62 -A------AT-----------------------CACTACAG--------- 3209

*msg*20 AAAT----ACAACGCAAGAAACAGATAAAAACACAATACAAGAAACAGAA 3134

*msg*3 -------------------------------------------------- 2495

*msg*104 -------------------------------------------------- 3252

*msg*76 -------------------------------------------------- 3288

*msg*79 -------------------------------------------------- 3253

*msg*85 -------------------------------------------------- 3261

*msg*7 -------------------------------------------------- 3360

*msg*25 -------------------------------------------------- 3289

*msg*37 -------------------------------------------------- 3242

*msg*68 -------------------------------------------------- 3569

*msg*62 -------------------------------------------------- 3209

*msg*20 ACAGATAAAAATACAACGCAAGAAACAGATAAAAACACAATACAAGAAAC 3184

*msg*3 -------------------------------------------------- 2495

*msg*104 --AAACCGAGACAAATACAACGCAAAAAACACTGACGACAGGAGAACAAT 3300

*msg*76 --AAACCGAGACAAATACAACACAAAAAACACTGATGACA---GAACAAT 3333

*msg*79 --AAACCGAGACAAATACAACACAAAAAACACTGACGACAGGAGAACAAT 3301

*msg*85 --AAACCGAGACAAATACAACACAAAAAACACTGACGACAGGAGAACAAT 3309

*msg*7 --AAACCGAGACAAATACAACACAAAAAACACTGACGACAGGAGAACAAT 3408

*msg*25 --AAACTGAGACAAATACAACACAAAAAACACTGATGACA---GAACAAT 3334

*msg*37 --AAACCGAGACAAATACAACGCAAAAAACACTGACAACAGGAGAACAAT 3290

*msg*68 --AAACAGATAAAAATACAACGCAAGAAACAGCGACAACAGGAGAACAAT 3617

*msg*62 --AAACAGATAAAAATACAACGCAAGAAACAGCGACAACAGGAGAACAAT 3257

*msg*20 AGAAACAGATAAAAATACAACGCAAGAAACAGCGACAACAGGAGAACAAT 3234

*msg*3 ----AC---------T--------AAA-A------------GAGAA--TT 2509

** * * * * *** *

*msg*104 GTATGTCGCTTTCTACAACAGACAAATGGATTACGCGTACGTCAACACAC 3350

*msg*76 GTATGTCGATTTCTACAACAGACAAGTGGATTACGCGTACGTCAACACAC 3383

*msg*79 GTATGTCGATTTCTACAACAGACAAATGGATTACGCGTACATCAACACAC 3351

*msg*85 GTATGTCGATTTCTACAACAGACAAATGGATTACGCGTACATCAACACAC 3359

*msg*7 GTATGTCGATTTCTACAACAGACAAATGGATTACGCGTACATCAACACAC 3458

*msg*25 GTATGTCGATTTCTACAACGGATAAGTGGATTACGCGTACATCAACACAC 3384

*msg*37 GTATATCGATTTCTACAACAGACAAGTGGATTACGCGTACATCAACACAC 3340

*msg*68 GTACGTCGATTCCTA---GAGACAAGTGGATTACGCGTACATTAACACAT 3664

*msg*62 GTACGTCGATTCCTA---GAGACAAGTGGATTACGCGTACATTAACACAT 3304

*msg*20 GTACGTCGATTCCTA---GAGACAAGTGGATTACGCGTACATCAACACAC 3281

*msg*3 ------------------------------------GTAAATC------- 2516

*** *

*msg*104 ACACACACATCTACACACACATCCGTACTAACACTAACTGTAACATTGAC 3400

*msg*76 ACACACACATCTACACAAACATCCGTACTAACACTAACTGTAACATTAAC 3433

*msg*79 ACACACACATCTACACAAACATCCGTACTAACATTAACTGTAACATTAAC 3401

*msg*85 ACACACACATCTACACAAACATCCGTACTAACATTAACTGTAACATTAAC 3409

*msg*7 ACACACACATCTACACAAACATCCGTACTAACACTAACTGTAACATTGAC 3508

*msg*25 ACACACACATCTACACAAACACCCGTAATAACATTAACTGTAACATTAAC 3434

*msg*37 ACACACACATCTACACAAACATCCGTACTAACACTAACTGTAACATTGAC 3390

*msg*68 ACATACATACCTACAGAAATATCTATAGAAACATTAACTGTAACATTGAC 3714

*msg*62 ACATACATACCTACAGAAATATCTATAGAAACATTAACTGTAACATTGAC 3354

*msg*20 ACACACACATCTACACAAACATCCGTACTAACATTAACTGTAACATTGAC 3331

*msg*3 ------------------------------------------------AT 2518

*

*msg*104 ATCAACGAAGGGATGTAAACCAGTTAAATGCACTATTGGAAGTGGAGAAG 3450

*msg*76 ATCAACGAAGGAATGTAAACCAATGAAGTGCACTAC-GGGAGTGGAGAGG 3482

*msg*79 ATCAACGAAGGAATGTAAACCAATGAAGTGCACTACCGGGAGTGAAGAGG 3451

*msg*85 ATCAACGAAGGAATGTAAACCAATGAAGTGCACTACCGGGAGTGAAGAGG 3459

*msg*7 ATCAACGAAGGGATGTAAACCAGTTAAATGCACTATTGGAAGTGGAGAAG 3558

*msg*25 ATCGACGAAAGGATGTCAACCAGCCAAATGCACTACCGGAAGTGGAGATG 3484

*msg*37 ATCAACGAAGGGATGTCAACCAGTCAAATGTACTACCGGAAGTGAAGATG 3440

*msg*68 GTCAACGCAGGAATGTCAACCGGTCAGATGTACTACTGGAAGTGAAGATG 3764

*msg*62 GTCAACGCAGGAATGTCAACCGGTCAGATGTACTACTGGAAGTGAAGATG 3404

*msg*20 ATCAACGAAGGGATGTAAACCAGTCAAATGCACTACTGGAAGTGGAGATG 3381

*msg*3 ATC----------------------------------------------- 2521

**

*msg*104 AAGCAGGAGATGTAAAACCAAGTAAAGGGTTAAGGATGAATGGGTGGAGC 3500

*msg*76 AAGCAGGAGATG--AAATCAAGTAAAGGGTTAAGGATGAATGGGTGGAGC 3530

*msg*79 AAGCAGGAGATGTAAAATCAAGTAAAGGGTTAAGGATGAATGGGTGGAGC 3501

*msg*85 AAGCAGGAGATGTAAAATCAAGTAAAGGGTTAAGGATGAATGGATGGGGT 3509

*msg*7 AAGCAGAAGATGTAAAACCAAGTAAAGGGTTAAGGATGAATGGATGGGGT 3608

*msg*25 AAGCAGGAGATGTAAAATCAAGTAAAGGGTTAAGGATGAATGGATGGGGT 3534

*msg*37 AAACAAGGGATGTAAAGCAAAATGAAGGATTAAAGATGAATGGATGGGGT 3490

*msg*68 AAACAGGGGATGTAAAGCAAAATGATGGATTAAAGATGGATGGATGGGGT 3814

*msg*62 AAACAGGGGATGTAAAGCAAAATGAAGGATTAAAGATGAATGGATGGGGT 3454

*msg*20 AAACAGGAGATGTAAAACCAAGTAAAGGATTAAAGATGAATGGGTGGAGT 3431

*msg*3 ----AGG--------AACCAAGTGAAGGTTTAAGAATGAATGGCTGGAGT 2559

* * ** * * ** **** *** **** *** *

*msg*104 CTAATAAAAAGAGTAATATTAATAATGATTATTTCAACTATGATT**TAA** 3548

*msg*76 CTAATAAAAAGAGTAATATTAATAATGATTATTTCAACTACGATT**TAA** 3577

*msg*79 CTAATAAAAAGAGTAATATTAATAATGATTATTTCAACTATGATT**TAA** 3549

*msg*85 TTGATAAAAGGAGTAATATTAACAATGATTATTTCAACTATGATT**TAA** 3557

*msg*7 TTGATAAAAGGAGTAATATTAACAATGATTATTTCAACTATGATT**TAA** 3656

*msg*25 TTGATAAAAGGAGTAATATTAACAATGATTATTTCAACTATGATT**TAA** 3582

*msg*37 CTGATAAAAGGAGTAATGTTAACAATGATTATTTCAACTATGATT**TAA** 3538

*msg*68 CTGATGAAAGGAGTAATATTAACAATGATTATTTCAACTATGATT**TAA** 3862

*msg*62 CTGATGAAAGGAGTAATATTAACAATGATTATTTCAACTATGATT**TAA** 3502

*msg*20 CTAATAAAAGGAGTAATATTAACAATAATTATTTCAACTATGATT**TAA** 3479

*msg*3 ATTATAAAAGAAACTATACTAGGAGTAATAATTTCACTTGTAATA**TAA** 2605

* ** *** * ** ** * * ** ****** * * * *

***msg*-III**

*msg*53 TAATAAGAACGCA-AGAAAAAATAATAAAAA-ATAC-AGAAATAGGAATT 47

*msg*46 ACC--------CA-AGAAAAAATAATAAGAA-ATAC-AGAAATATGAATT 39

*msg*107 ATA------------------ATAATAAAAA-ATAC-AGAAATAGGAATT 30

*msg*34 AAA------------------ATAATAAAAA-ATAC-AGAAATAGGAATT 30

*msg*110 AAAA-----CCCATAGAAAACTAAATTGAAA-AAAT-AGAA-CA-GATAT 41

*msg*8 AGAA-----GATA--AAAAAAACAATAAAAATATATAAGCACTAAGAATC 43

*msg*55 TCA----------------------TAGAA-------------------- 8

* *

**upstream potential TATA box**

*msg*53 ATAAAAAGTATTACTAATAA-AATTATA-TCATTTAAAATATTTAA**TATA** 95

*msg*46 ATAAAAAGTATTACTAATAA-AATTATA-TCATTTAAAATATTTAA**TATA** 87

*msg*107 ATAAAAAGTATTACTAATAA-AATTATA-TCATTTAAAATATTTAA**TATA** 78

*msg*34 ATAAAAAGTATTACTAATAA-AATTATA-TCATTTAAACTATTTAA**TATA** 78

*msg*110 AAAAAAATCAAGAC-AATACTATTTATA-TCTATAAAAA--TGAAATAA- 86

*msg*8 ATAAAAATTATATTTAATAA-ACT**TATAATT**ATTTAAAACTTT---**TATA** 89

*msg*55 -----AA-GATTGCTAATAA-AATTATA-TCATTTATAACATTTAC**TATA** 50

** * **** * ***** * * * * * **

*msg*53 **TTT**TAAGAATATATTTTTATGAAAATTTATAGATTTAAAAGTAAAAACTA 145

*msg*46 **TTT**TAAGAATATT-TTTTATGAAAATT**TATAAAT**TTAAAAACAAAAACTA 136

*msg*107 **TTT**TAAAAATATTTTTTTATGAAAATTTATAGATTTAAAAACAAAAACTA 128

*msg*34 **TTT**TAAGAATATTTTTTTATAAAAATTTATAGATTTAAAAGTAAAAACTA 128

*msg*110 -----AAGATAATGAATAATAATAAAAAGAGTATTAGAAAGCAAAAATCC 131

*msg*8 **TTT**TAAAAATAATTTTTTATAAAAATTTAAAAACATAAAAATACAAACTC 139

*msg*55 **TTT**TAAGAATATTTTTTTATAAAAATT**TATAAAT**TTAAAAGTAAAAACTA 100

* *** * ** * ** * *** * ***

*msg*53 GATG-AAAAATAAAACATGTAAA------**TATAATA**CAA-AA**TA-TAATA** 186

*msg*46 AACACAAAAATAAGACATGTAAA------TACAATACAA-AAC---AATG 176

*msg*107 AACACAAAAATAAAACATGTAAA------TACAATACAA-AA**TA-TAATA** 170

*msg*34 GATG-AAAAATAAAACATGTAAA------**TATAATA**CAA-AA**TA-TAATA** 169

*msg*110 ACTA-AGAGTAAAAATATCTAAG------TACAATACAA-AAT---AATA 170

*msg*8 AGTA-AG---AAAAACATATAAT------**TATAATA**AAGTAATGTTTATG 179

*msg*55 GATG-AAAAATAAAACATGCAAATACAAA**TATAATA**CAA-AA**TA-TAATA** 147

* ** * ** ** ** **** * ** **

*msg*53 CA-AAACAAT----ATCTATCT-ATTTATCGCATATCGGT-TATTAA-TA 228

*msg*46 CC-------T----ATCTATCT-ATTTATCGCATATCAGT-TATTAA-TA 212

*msg*107 CA-AAACAAT----ATCTATCT-ATTTATCACATATCGGT-TATT----A 209

*msg*34 CA-AAACAAT----ATCTATCT-ATTTATCGCATATCGGT-TATTAA-TA 211

*msg*110 CA-------C----ATTTACCT-ATTTATT---TATTGTA-TTTTAT-TA 203

*msg*8 TTTAATCATT----TATTGTATAATTTGTTATATATCTAT-TAT----TA 220

*msg*55 CA-AAACAATACTTATCTGTCT-ATTGATTGTATATCGATTTATCGATTT 195

* * *** * *** * *

*msg*53 TTATCG-TTTATCGT----TATT--TTTTAGTGAGAACGATATTTTTTCG 271

*msg*46 TTATCG-TTTATCAC----TATT--TTTTAGTGAGAACGATATTTTTTCG 255

*msg*107 TTATCG-TTTATCGC----TACTTTTTTTAGTGAGAACGATATTTTTTCG 254

*msg*34 TTATCG-TTTATCGT----TATT--TTTTAGTGAGAACGATATTTTTTCG 254

*msg*110 TTATCG-TTTATCGC----TACT--TTTTATTGAGAACGATATTTTTTCG 246

*msg*8 TTTAGT-TATATCGG----AATT--TTTT-TTG---TCGATATTTTTCAA 259

*msg*55 TTAATATATCATCGTTTACAGTT--TTTTAATGAGAATGGTATTT-TTCG 242

** *** * **** ** * ***** *

**TATA box Cap signal**

*msg*53 ATGTCAAATATGAACCATTTTT**TATAAAA**ATT-A-----TGTTAT**CA**TTA 315

*msg*46 ACGTCAAATATGAAACATTTTT**TATAAAA**ATT-A---TTTGTTGTTATTA 301

*msg*107 ATGTCAAATATGAAACATTTTT**TATAAAA**ATC-ATTATTTGTTGT**CA**TTA 303

*msg*34 ATGTCAAATATGAAACATTTTT**TATAAAA**ATT-A---TTTGTTGTTATTA 300

*msg*110 ATATCAAATATGAAGCACTTTT**TATAAAA**ATT-ATTATTTGTTAT**CA**TTG 295

*msg*8 ATATTAAA--TGATACTTTTTT**TATAAAA**TTTTATATGTTGTTTT**CA**TTA 307

*msg*55 ATCTCAAATATGAAACACTTTT**TATAAAA**ATT-ATTATTTGTTAT**CA**TTA 291

* * *** *** * *********** * * **** * ***

*msg*53 TAG**ATG**AAAACATTTGCGTTAGCTAGTTTTCTTGGTATAGCGTATGCTTT 365

*msg*46 TAA**ATG**AAAGCATTCGTATTAGCCAGTTTTCTTGGTATAGCGTATGCTTT 351

*msg*107 TAG**ATG**AAAACATTCGTATTAGCCAGTTTTCTTGGTATAGCGTATGCTTT 353

*msg*34 TAG**ATG**AAAGCATTCGTATTAGCCAGTATTCTTGGTATAGCGTATGCTTT 350

*msg*110 TAG**ATG**AAAGTGTTTGCATTAGCCAGCTTTCTTGGAATAGCATATGCTTT 345

*msg*8 TAA**ATG**AAAAGATTTACGCTATTTTGTTTTCTTAGAATAGCATATACTTT 357

*msg*55 TAG**ATG**AAAGCATTTGCATTAGCCAGCTTTCTTGGTATAGTGTATGCTTT 341

** ****** ** ** * ***** * **** *** ****

**<-----------------intron 1----------**

*msg*53 CTCAAAAAACATTG**GTATTA**TATGTATTATATTTTATT-TTTTCACATTT 414

*msg*46 CTCGAAAAACATTG**GTATTA**TATATATTATATTTTATT-TTTTCACGTTT 400

*msg*107 CTCAAAAAACATTG**GTATTA**T---------ATTTTATT-TTTTCACGTTT 393

*msg*34 CTCAAAAAACATTG**GTATTA**T---------ATTTTATT-TTTTCACGTTT 390

*msg*110 CTCAAAAAACATTG**GTATTA**T---------ATTTTATTTTTTTCACGTTT 386

*msg*8 TTCAAAAAATAATG**GTATTT**T---------ATCTTTTT-TTTTC---TTT 394

*msg*55 CTCAAGAAACATTG**GTATTA**T---------ATTTTATT-TTTTCAGATTT 381

** * *** * ******* * ** ** ** ***** ***

**-------------->**

*msg*53 TTAACTTTTTTT**TAG**AATTCATTCATAAAGGATCTGCATCAAATCCATCA 464

*msg*46 TTAACCTTTTTT**TAG**AATTCATTCATAAAAGATCTGCATCAGATCCATCA 450

*msg*107 TTAACTT-TTTT**TAG**AATTCGTGCACGAAAGATCTGCTTCAGATCCATCA 442

*msg*34 TTGACTT-TTTT**TAG**AATTTGTTCATAAGAAATCTGCATCGGATCCATCA 439

*msg*110 TTGACTTTTTTT**TAG**AATTCATTCATAAAAAATCTACATCAAATCTATCA 436

*msg*8 TTAA-TT-TTTTTAAAACTGATTAATAACA**AAG**----------------- 424

*msg*55 TTAACTT-TTTT**TAG**AATTTATTCATAAGAGATCTGACTCAGATCCATCA 430

** * * ****** ** * * * * *

*msg*53 CAAGATCCATCAC------------------------AAAATC---CATT 487

*msg*46 CAAGATCCGTCACAAGATCCGTCACAAGATCCGTCACAAGATCCATCATT 500

*msg*107 CAAGATCCAC------------------------------------CATT 456

*msg*34 CAAAATCCATTAC------------------------AAAATC---CATT 462

*msg*110 CAAAATCCATCGC------------------------AAAATT---TATT 459

*msg*8 ----AACCG------------------------------ACCT---TATT 438

*msg*55 CAAAATCCATCACAAAATCCATCACAAAATCTATCACAGAATT---CATT 477

* ** ***

**<-------------**

*msg*53 AGACTTACCATCCGATTTATCATTAGATTCATTAAG**GTATAG**AAAATTTT 537

*msg*46 AAACTTATTACTTGATTCATCATTAGATTCATTAGG**GTATAA**AAAATTTT 550

*msg*107 AAACTTATTACTTGATTTATCATTAGATTCATTAGG**GTATAA**AAAATTTT 506

*msg*34 AGACTTATCATTCGATTTACCATTAAGTTCATTAGG**GTATAG**AAAATTTA 512

*msg*110 AGATTTGTCATTCGATTTATCATTAGATTCATTAGG**GTATAA**AAATTTTT 509

*msg*8 AGATACATCGTTAGAC---TCATTAGATTTAATAGG**GTATAA**AAACTTTT 485

*msg*55 AGACTTATCATTCGATTTATCATTAAATTCATTAAG**GTATAA**AAATTTTT 527

* * ** ***** ** * ** ****** *** ***

**<---------------intron 2---------------->**

*msg*53 AAGCAGCAACAAAACATACAAAACTAATATTT-TTATG**CAG**CGAAGAAAA 586

*msg*46 AAGCAGCAACAAAACATACAAAATTAACATTT-TTATG**CAG**CGAAGAAAA 599

*msg*107 AAGCAGCAACAAAACATACAAAATTAACATTT-TTATG**CAG**CGAAGAAAA 555

*msg*34 ---TAGCAACAAAATATGCAAAATTAACATTT-TTATG**CAG**CGAAGAAAA 558

*msg*110 AAGCAGCAACAAAACATACAAAATTAACACTT-TTATG**CAG**CGAAGAAAA 558

*msg*8 AATTAGTGGCAAAACATTCA--ATTAACATTTTTTATC**TAG**TAAAGATAA 533

*msg*55 AAACGACAACAGAACATACAAAATTAACATTT-TTATG**CAG**CGAAGAAAA 576

** ** ** ** * *** * ** **** ** **** **

*msg*53 AGTTTTATCTTTGATTCTAGGAAAAAACCCAG---AATTTCAATGTAAAA 633

*msg*46 AGTTTTAGCTTTGATTTTAGGAAAAAATGCAATAGACAATGAATGTGAGG 649

*msg*107 AGTTTTATCTTTGATTCTAGGGAAAAACGCAATAGAGTCTCAATGTGAGG 605

*msg*34 AATTTTAACTTTAATCCTAGGAAAAAATGCAATAAATGCTCAATGTGAGA 608

*msg*110 AATTTTAACTTTAATTCTAGGAAAAGACGCAATAAGTGCTCAATGCAAGA 608

*msg*8 GGTTTTAACGCTACTTCTAGGAAAAGAGGCAATAAGCGATCAATGTGTAC 583

*msg*55 AATTATAACTTTAATTCTAGGAAAAGACGCAATAGAGTCTCAATGTGAAG 626

** ** * * * **** *** * ** * ****

*msg*53 AAAAACTAAAAGAATACTGCAGCGATTTGAAAGGTCTGGCCTTGGAGCCA 683

*msg*46 AAAAACTAAAAAAATACTGTAACGATTTAGAAGGTCAAGCCTTGAAGCCA 699

*msg*107 AAAAACTACAAAAGTACTGCAGCAGTTTGAAAAGTATGGCCTTGGAACCA 655

*msg*34 AAAAACTAGAAGAGTACTGCAAGAATTTGAAAAACACTAGTTTAGTTCCA 658

*msg*110 AAAAACTAGAAGAATACTGCAGCAATTTGAAAAATAGCAACTTAGTTCCG 658

*msg*8 CAAAGCTACAAGAATACTGCAAAAATTTGAAAAATATGGCTTTAGAACCG 633

*msg*55 AAAAACTACAAAAGTACTGCAACAATTTAAAAAGTATGACCTTGGAGCCA 676

*** *** ** * ***** * *** ** ** **

*msg*53 AAGAACGTGCATCCTACATTAGAAGGACTTTGTAAA------AATACAGA 727

*msg*46 AGAAATGTACATCCTGCATTAAAAGGACTTTGTGAA------AATGCAAA 743

*msg*107 AAGAATGTACATCCTGTATTAGAGGAGCTTTGTAAGGAAGGGAAAGCAGG 705

*msg*34 GAGGATGTACATCCTGCATTAAAAGGACTTTGTGAA------AATGCAAA 702

*msg*110 AAAAATCTACATCCTGCATTAAAAGAACTTTGTGGA------AATGCAGA 702

*msg*8 AAGAATGTGCATCCAGCATTAGGGGGGCTTTGCGGT------AATGAAGA 677

*msg*55 AAGAACGTGCATCCTACATTAGAAGGACTTTGTAAA------AATGCAAA 720

* * ***** **** * ***** ** *

*msg*53 GAAGAAATGCAACGATCTTGAAACTCAAATTGATACTCTCTCGAGTAATG 777

*msg*46 ACAGAAATGCACTTATCTTAAAACTAAAATTAATGCTGTCTCAAATAATG 793

*msg*107 ACAAAAATGCACTAATCTCAAAAATGGAATTATTTCTATTTCGAATGGTA 755

*msg*34 ACAAAAATGCACTAATCTCAA---TAAAATTATTTCTACTTCGAATAATA 749

*msg*110 GAAGAAATGCAATGATCTTAAAACTCAAATTAATATTCTCATGAATAATA 752

*msg*8 TGAGGAATGTAATAATTATAAAAATAGAATTAATACTACTTTAACTGGTA 727

*msg*55 ACAGAAATGCAAAAATCTTAAAACTCAAATTGATACTTTCATATATAATA 770

* **** * ** * * **** * * * *

*msg*53 CCAAGGATTATCTTCAAGATATTATTGCGAGTAGTTCA--GGTAATAT-- 823

*msg*46 TCAAGACTCATCTTATAGATATTATGACGAATATTGCA--GATAAAAAAG 841

*msg*107 TCAAGAATCATCTTCTGGGTATTATGACAAATATTTCA--AGCAATGT-- 801

*msg*34 TCAAGGATCATCTTCAAGATATTCTGAAGAATATTTCA--AATGAATTGC 797

*msg*110 CCAATGAGTCTCTTCTAGATATTGCTTTAAATATTTCA--ACTGAAGGGT 800

*msg*8 TCAAAGAATCTCTTATGCGTATTT--ACGAAAAGCTCAATACTGACGGAT 775

*msg*55 CCGAAAATTCTCTTCTATATATTTATGCAGAACTTGTA--GATGGAAATA 818

* * **** **** *

*msg*53 -GGTTTTAGGAAAAACCCGTTGCAACTATGCTCATATCCAATGCATGATT 872

*msg*46 CTCATTTGAGAAAAAAATACTGCAACTATGCTCATATTCAATGCATGTTT 891

*msg*107 -GCTTTTAGAAAAAACCCATTGTAATTATACTCATATCCAATGCATGATT 850

*msg*34 TTCTTTTAGAAAAAACCCATTGTAACTATGTCCATATCCAATGCACGATT 847

*msg*110 TTCATCTAGAAAAAAAATACTGCAACTATGCTCATGTTCAGTGCATATTT 850

*msg*8 CTACTTTGGATCAAAATGAATGTAATTACTCTTATTTTCGATGTTTTTTT 825

*msg*55 CCGATTTATATCAAACTTATTGTAATTATGCGCATTCTCAATCTATTTTT 868

* * *** ** ** ** ** * * **

*msg*53 TTCCGAGAATTTTTTGGTTTTTCTTCTATATGCGATGAGATAGCTGAACA 922

*msg*46 TTCCGAGAAATTTCCAGTTTTTCTTCTATATGCGATGAGATAGCTGAACA 941

*msg*107 TTTTGGGAATTTTCTGGTTTTTCTTCTATATGCAATGAGATAGCTGAACA 900

*msg*34 TTCCGAGAATTTTCTGGTTTTTCTTCTATATGCAATGAGATGGCTGAACA 897

*msg*110 TTCCGAGAATTTCCTAATTTTTCTACTGTCTGCGATACTATGGCTGAACA 900

*msg*8 TTCTGGCATTTTCCTGATTTTTTAACTATCTGTAATCTTATAATTGAAAA 875

*msg*55 TTCCAAGATTTTCCTAGTTTTTTAGCTACATGTAATGGTATAGCTGACCA 918

** * ** ***** ** ** ** ** *** *

*msg*53 ATGTTATCGTCAAATAAATGAAGATTTAGCTTATGAAGTTCTTTTAAGGG 972

*msg*46 ATGTTATCGTCAAATAAATGAAGATTCAGCTTATAAAGTTCTTTTAAGGG 991

*msg*107 ATGTTATCATCAAATAAATGAAGATTTGGCTTATGAAGCTCTTTTAAGGA 950

*msg*34 ATGTTATCGTCAAATAAATGAAGATTTAGCTTATGAAGCTCTTTTAAGGG 947

*msg*110 ATGTTATCATCAGATAAATGAAGATTTTGCTTATGAAGTTCTTTTAAGAG 950

*msg*8 ATGTTATGAAAAAATGAGTCAACATCTAGCTTATAAAGTTCTTTTTAAAT 925

*msg*55 ATGTTATAGAAGAAGAAGCAAAGAAATAGCATATGAAATTCTTATAAGGG 968

******* * * ** * ** *** ** **** * *

*msg*53 CTCTTCCTAGAGATTCGAAAAATCAAGTAACATGCGAAGAAAAAATTAAA 1022

*msg*46 CTCTTCCTAGGGATTTGAAAAATCAAGTAACATGTGAAGAAAAAATTAAA 1041

*msg*107 CTCTTCCTAGGGATTTGGAAAATCAAGCAATATGTGAAA-AAAAATTAAA 999

*msg*34 CTCTTCCTAGGGATTCGAAAAGTCAAGCAGCATGTGAAGAAAAAATTAAA 997

*msg*110 CTCTTCCTAGAAATTTGGAAAATCAAGCAGCATGTGAAGAAAAAATTAAA 1000

*msg*8 CTCTTACCGGGAATTTAAAAAATCAGACTACTTGTGAAATTAATCTTAAG 975

*msg*55 CTTTTGGTAGCAATTTAAAAGGCAGAAATACATGTGAAATCATGATTAAA 1018

** ** * *** ** ** *** * ****

*msg*53 GAATCATGTTTCAAGTTAAATAGAGAAAGTTATCATTTACTCTGGTTTTG 1072

*msg*46 GAATCATGTTTCAAGTTAAATAGAGAAAGTTATCATTTACTCTGGTTTTG 1091

*msg*107 GAATCTTGCTCCAAGT--AATAGAGAAAGTTATTATTTACTCTGGTCTTG 1047

*msg*34 GAATCATGTTTCAAGTTAAATAGAGAAAGTTATCATTTACTCTGGTTTTG 1047

*msg*110 GAACCATGCTTCAAGTTAAATAGAGAAAGTTATTATTTACTCTGGTCTTG 1050

*msg*8 GAACCTTGCTCAAAATTCAGTACTGAGAGTTATTATTTAGCGTGGTATTG 1025

*msg*55 GAACTTTGCTTAAAACTAAGTACTGAAAGTTATTATTTACTATCACATTG 1068

*** ** * ** * ** ** ****** ***** * ***

*msg*53 TTTTCTCTTAAAAAAAACATGTGAGATTCTCGTTAATAAAATAAGAGACA 1122

*msg*46 TTTTCTCTTGAAGAAAACATGTGGAGTTCTCCTTAATAAAACAAAAGACA 1141

*msg*107 TTTTCTCTTGAAGGAAACATGTGGAATTCTCCTTAATAAAACAAAAGATA 1097

*msg*34 TTTTCTCTTGAAGAAAACATGTGGAGTTCTCCTTAATAGAACAAAAGACA 1097

*msg*110 TTTCCAAAAAAAATATACATGTGAAAATCTCCTTAAAAAAA---AAAGTA 1097

*msg*8 TTTCTGGAAAAGAACTACATGCATGACTCTTATCAAGAAAGCACAAGATA 1075

*msg*55 TTTCCTTCAAGAACAAACATGTGAGATTCTTGTTACAAGGGCGGCTAATG 1118

*** ***** *** * * *

*msg*53 ACTGTGGAGCTATTGA---AGA---TCTCAAAAATACATTAAAAAAGACT 1166

*msg*46 ACTGTGATGCTCTTAA---AGA---TATCAAAAATACATTAAAAAATGCT 1185

*msg*107 ACTGTGAAGATCTTAA---AAA---TCTAAAAAATACATTGAAAAATGTT 1141

*msg*34 ACTGTAATGCTCTTAA---AGA---TCTCA---ATGCATTAAAAAATACT 1138

*msg*110 ACTGCAAATCTCTTGAAAAAGA---TATCGAAAATACATTTCAAGGTGCT 1144

*msg*8 ATTGTAAAGCTCTTAA---CAATAGTATTGAAAATGCACTTAAG---GAT 1119

*msg*55 ACTGCCAAACTCTTAA---AAA---TTTTCAAAGTATATATGG---AGCT 1159

* ** * ** * * * * * * *

*msg*53 AATATATTAAAAAATGACTGTTATCCTTTACTCAGAAAATGTTATTTTCA 1216

*msg*46 AGTACATTAAAAGATGACTGTTATCCTTTACTCAGGAAATGTTATTTCCA 1235

*msg*107 AATACGTTGGGAAATGACTGTTATCCTTTACTTAGGAAATGTTATTTCCA 1191

*msg*34 AGTACATTGAAAGATGACTGTTATCCTTTACTCAGGAAATGTTATTTCCA 1188

*msg*110 CATAAATTAGAAAACGTTTGTCACTCTTTACTCGAGAAATGCTATTTCCA 1194

*msg*8 CATACGTTAGAAAAACAATGTCATTCTCTACTTCAAAGATGTTATGTTCA 1169

*msg*55 ACTTCAATAGAAAAAACCTGTTACTCTCTTTTTGAGAAATGCTATCTTCT 1209

* * * * *** * ** * * * *** *** * *

*msg*53 CTCATCAAACTGTGAAAATGAAAAGGTGCAGAACTGTGTGGAGTTTAAAA 1266

*msg*46 CTGGCCAAACTGTGAAAATGAGAAGGTGCAGAACTGTGTGGAGCTTAAAA 1285

*msg*107 CTCATCAAACTGCGATGAAGAGGATAAACAAAAATGTGCGAAACTTAAGA 1241

*msg*34 CTCGTCAAACTGCGAAAATGGAGAAGTGCAGAACTGTATGGTGCTTAAAA 1238

*msg*110 CTCGCGAGACTGTGAAAATGGAGAGATGCAGAACTGTATGGAGCTTAAAA 1244

*msg*8 TTTTCCAGATTGTGAAGA------AACACTGAATTGCGAGAACTTTAAAA 1213

*msg*55 CTCATCAATCTGTGAAGAAG---GTATACAGAGATGTAAGAATATTAAAG 1256

* * ** ** * * * ** * ****

*msg*53 AACGTTGCAAAGAAAAAAATATCACATACCCTCCCC-CCCTCCCCCCCCA 1315

*msg*46 AACGTTGCGAAGGAGAAAATATCACATATCCCT--------CCC------ 1321

*msg*107 GTCTTTGTAAGGAAAAGAACATCATATATTCCC--T-----CCCC---C- 1280

*msg*34 AACATTGCGAAAAAGAAAATATCATATACCCCC--C-----CCACACACA 1281

*msg*110 AACGTTGCGAAAAAGAAAATATCAAATATCCTT--------CCC------ 1280

*msg*8 AACGCTGTGAAGAGAAAGGCATTACATACTCTC--C-----TTTT---C- 1252

*msg*55 AACAATGCAAACAAAAAAATATAGAGTACCCTCTCTTTCCTTTCT-TCC- 1304

* ** * * ** ** *

*msg*53 CACACACAGTCTTTCTTTTAATCCATTAGACTTTCCACCTACATTGCAGG 1365

*msg*46 --CCCACAGTCTTTCTTTTAACCCATTAGACTTTCCACCTACATTGCAGG 1369

*msg*107 --ACCACGATCTTCCTTTTAACCCATTAAACTTTCCACTTACATTGCAGG 1328

*msg*34 CACACACAGTCTTCCTTTTAACCCATTAGACTTTCCACCTACATTGCAGG 1331

*msg*110 --CCCACAATCTTCCTTTTAACCCATTAGAGTTTCCGCCCACATTACAAG 1328

*msg*8 --ATGATGGTCTTT-TTTTGATCCA--AGGTTTACCGCCTACATTAGAAA 1297

*msg*55 --CTCACCGTTTTTCTTTTAACCCAATAGAATTTTCGCTTACACTACAAG 1352

* * ** **** * *** * ** * * *** * *

*msg*53 AGAAAATAGATCTTCAACAGCTTCATACAGAAGCGTTAACTTTTGGTATT 1415

*msg*46 AGAAAATAGATCTTCAACAGCTTCAAACAGAAGCGTTAACTCTTGGTATT 1419

*msg*107 AAAAAATAGATTTTCAACAGCTTCACACAGAAGCGTTAACTTTTGGTATT 1378

*msg*34 AGAAAATAGATCTTCAACAGCTTCACACAGAAGCGTTAACTCTTGGTGTT 1381

*msg*110 AAGAAATAGGTTTTTATCGGCTTTATAATGAGGCTGCATCTCATGGTGTC 1378

*msg*8 AAAGAATAGGTTTTCAACAAATTTTTGCTAAAGCAGCGGCTACTGGTATT 1347

*msg*55 AGAAAATAGGTCTTGAGCAGCTTTATGTAGATGCATTAAATTATGGTATT 1402

* ***** * ** * * ** * ** * **** *

*msg*53 TTTCTTGGAAGGTCCTTGTCGACAAAGTTTTCTCATTTTTTGTTATTTTC 1465

*msg*46 TTCCTTGAAAGGTCCTTGTCGACAAAGTTTTCTCATTTTTTATTATTTTC 1469

*msg*107 TTTCTTGGAAAACCCTTGTCGACAAAGTTTTCTCATTTTTTGTTATTTTC 1428

*msg*34 TTTCTTGGAAAATCCTTGTCGACAAAGTTTTCACATTTTTTGTTATTTTC 1431

*msg*110 TTAATTGTCAAATCTTTATCAATAGGATTCAGTCATTTTATTCTGTTAGC 1428

*msg*8 TTAGTCAGTAAACCTACAATTACGAAATCCTATCGTTTTTTATTATTATC 1397

*msg*55 TTTTTGAAAAAGTCTTTATTAACAGATCTTTCTCGTTTTTTAGTGTTTAC 1452

** * * * * * **** * * ** *

*msg*53 AAACTATTATACTGGAGACAATGCA--AGTAAATTA-GACAA-TACTCAG 1511

*msg*46 AAAACATTATGCTGGAGACAATGCA--AGCAAATTA-GACAA-TACTCAG 1515

*msg*107 AAAACATTATGCTGGAGAAAATGTA--GATAATTTA-GACAA-TGCTAAG 1474

*msg*34 AAAACATTATCTTGGAGACAATGTA--GGTAATTTA-GATAA-TGCTAAG 1477

*msg*110 ATACCATAT---TACAAAC---CCA--GCCCA---A-AACAG-AACTAAA 1465

*msg*8 ACTCCATAATA-A---------GCA--GATAAGT----GCAGTTGCT--- 1428

*msg*55 AAATTATTATGATGGATACCTTGAATCAATAAGATACAATAA-TACTCAA 1501

* ** * * * **

*msg*53 TCTTGCACTAAATCTTTGGAAAATTGCGTTTCTTTTAAATATCTGACTGA 1561

*msg*46 TCTTGCGCTAAATCTCTGGAAAGCTGTACTTCTTTTAATTATCTGACAAA 1565

*msg*107 TTATGCACTAAGTCTCTGAAAAACTGCGCTTCCTTTGAATACATAACAGA 1524

*msg*34 ATATGCACTAAGTCTCTGGAAAACTGCGCTTCTTTTGAATACCTAACAAA 1527

*msg*110 AGATGCATTAAATATCTAGAAAACTGCAC---TTTTGAATATTTAACAAA 1512

*msg*8 GGATGCGTTTATTATTTAAATAGATGTGC---TTTTAAACAATTGTCAGA 1475

*msg*55 AGATGTGTTGCTTATTTAAGGGAATGTTCTTCTTTTGGATACTTAACAAA 1551

** * * * * ** *** * * * *

*msg*53 AGAACTAGCAAATATATGCAGCGCAACTAACAAAAATGAAGCATGCAAAG 1611

*msg*46 AGAACTAGCAAATATATGCAGCACAACTAACAAAAATGAAACATGCAAAG 1615

*msg*107 GGAACTAGCAAATATCTGCAAAAAAACTGACCAAAATAAGGTCTGCGAAG 1574

*msg*34 TGAACTAGCAAATATGTGCAACGAAACTGACAAAGATAAAGCATGCAAAG 1577

*msg*110 AGAACTAGCGGATTTATGCAATGGAACTAACCGTACTGAAGTATGCGGAA 1562

*msg*8 AGAATTAGCAAATATGTGTAACGCAACTAACATTGTTGAAATTTGTGTGG 1525

*msg*55 AGAATTAACAGCTCTATGCAGTACAAAAAACAAAGACGAAACATGCAAAC 1601

*** ** * * * ** * ** ** * **

*msg*53 AGTTAAACAACGAACTAGAAAGAGAATACAAATCTCTTAAATTAATTCTC 1661

*msg*46 AGTTAAACAATGAACTGAAAAGAGAATACATGTCTCTCAAATTGCTTCTC 1665

*msg*107 AATTAAACAATGAACTAGAAGAAGAATACATGTCTCTCAAATTAGTTCTC 1624

*msg*34 AGTTAAGTAATGAATTAGAAAGAGAATACAAGTCTCTCAAATCAGCTATT 1627

*msg*110 AAATAGATAAAGAAGTCCAAAGAGAATGCAATTCTCTTCAATTAGCTTTT 1612

*msg*8 AATTAAGTAAACAAGTACCAGAGGAATGCAACTCTCTTGAATTAGCTTTG 1575

*msg*55 AATTAATAAATGAGCTACAAGATGAATGCAACACTCTCAGCTTAGACCTC 1651

* ** ** * * * **** ** **** * *

*msg*53 TATAACAGAAAGCTTTCTAACGTAAGCGATACTGCTGACTCTAAAT---- 1707

*msg*46 TATAACAAAAAGCTTTCTGACGTAAGCGATACTACTGATTCTAAAC---- 1711

*msg*107 TATAACAAAAAGCTTTCCAACGTAAGCGATGCTACTAACTCTAAAT---- 1670

*msg*34 TATCAT---AAGTTTTCCAACCCAAGCGATGCTACTAAATCTAAAT---- 1670

*msg*110 CAAGAAAAAAATCTCTTTAAGATGAAATCTGAGGCGAAATCTAAAC---- 1658

*msg*8 AAAAACAAAGGACTCTTTAATATGAGTGCTACTGATAAATCTGATC---- 1621

*msg*55 TATAAGAAAAACTTCACCTCCATAACCGTTGATGTTCAATCTATACCCTT 1701

* * * * * * ***

*msg*53 --CGTACTCATGGAGCGAACTACCAGGGACAATTTCCAAGGAAGACTGTA 1755

*msg*46 --CGTACTCATGGAGCAAACTACCAGGGACAATTTCCAAGGAAGACTGTA 1759

*msg*107 --CCTACTCATGGAGCGAATTACCAGGGACAATTTCCAAGGAAGACTGTA 1718

*msg*34 --CGTACTCATGGGATGAATTACCAGGAACAATGTCCAAAGAAGACTGTA 1718

*msg*110 --TCTATACTTTGAATGGGCTGACAAAAATTATTCATGAGGAAAGCTATG 1706

*msg*8 --TATACACTTTACATGGACTATCCAAGATAGTTCCTGAAGAAGATTATG 1669

*msg*55 AACCTACTCTTCGAATGAAACATCAGAAACAATTTCAGAAGAAGAGTGTG 1751

** * * * * * * *** * *

*msg*53 TAAACCTTAGCCATAAATGCTATTCTATGGATCCATATTCTAACAATACT 1805

*msg*46 TAAGTCTTAACCATAAATGCTATTGTATGGATCCATATTCTAATAATACT 1809

*msg*107 CAAGTCTTAACTATAAATGCTATTGTATGGATCCATATTCTAATAATACT 1768

*msg*34 TAAGCCTTAGCTACAAATGCTATCATATGGATCCTTATTCTAATAATGCT 1768

*msg*110 CCACTCTTATTTCAAAGTGCACTTACATCCAATCGTACTGCAGTGAAGAT 1756

*msg*8 CGAGTCTTGTTCTAGATTGTTATCATCTAGAGTCACTTTGCAATCAGAGT 1719

*msg*55 TAGCCCTTATCCAAAAGTGCAATTATCTAGATCCATTTTGCGATGATATT 1801

*** * ** * * * * * * *

*msg*53 CTTTACAATGCTTGTAGAAATCTAAAACTAGAGTGTTTTAAATCTGCAAC 1855

*msg*46 CTTTACAATGCTTGTAGAAATCTAAGACTAGAGTGTTTTAAATCGGCAAT 1859

*msg*107 CTTTACAATGCTTGTAGAAATCTAAGACTAGAGTGCTTTAAATCGGCAAT 1818

*msg*34 CTTTACAGTGCTTGTAAAAATATAAAACTAGAGTGTTTTAAATCGGCAAT 1818

*msg*110 CTCATAGATGCATGCCTAGCTCTAAAAGTAGCATATTATAAGGCTCAGTT 1806

*msg*8 ATTATGGATTCATGTAACAATTTAAGACAAGTATATCATAGAAGTCAATT 1769

*msg*55 ATTTTTGATGCGTGTAATAATCTAAGACGAGCATGTTCTAAGTTAGCATT 1851

* * * ** * *** * ** * **

*msg*53 TTACGGACTGGCAAGGGATGTGTTGGAGGATGGATTGTTTGGATTACTTC 1905

*msg*46 TTACGGACTGGCAAGGGATCTGTTGGAGGAAGGATTGTTTGGATTACTTC 1909

*msg*107 TTATGGACAGGCAAGGGATGTGTTGGAGGAAGGATTGTTTGGATTACTTC 1868

*msg*34 TTACGGATTGGCAAGGGATGTGTTGGAGGAAGGATTGTTTGGATTACTTC 1868

*msg*110 TTATAAATTAGCAAAAGAAGTATTGGAGGATGGATTGTTTGGATTGTTTC 1856

*msg*8 TTATAATATAGCAAAAGATAAATTAGAAAAAGAATTGTTTGGATTATTTC 1819

*msg*55 CTACAAATCAGCATACGAAGTATTAGAAGAGGGACTGTTTGGTTTGCTTC 1901

** *** ** ** ** * * * ******* ** ***

*msg*53 ACAATTTAGATTCAAACAGAACTAAAGAGTGTATAGCCAAACTAATAGAA 1955

*msg*46 ACGATTTAGATTTAGATAGAGCTAAAAAATGTGCACAGAAATTAGTAGAA 1959

*msg*107 ACAACTTAGATCCAAAAAGAACTAAAGAGTGTATAGCCAAACTAGTAGAA 1918

*msg*34 ACGACTTAGATTTAGATGGAACTAAAAAATGTATAGACAAACTAGTAGAA 1918

*msg*110 ACAACTTAGACTCAGATGGGACTAAAAAATGTATAGACAAACTAGTAGAA 1906

*msg*8 GTAATTTAAGTTCAAGCGGGGTTAAGGAATGTGCAGTCAAGTTGACACAA 1869

*msg*55 ACAATTTAACTTCAAACAGACTTAAGGAATGTGCAGCTAAATTAGTGAAA 1951

* *** * * *** * *** * ** * **

*msg*53 AGGTGCCAAATAGTCAGAAACAATAGTATAGTTATACTTTCAATGTGTTT 2005

*msg*46 AAATGCCAAATGGTCAGAAACAATAGTATAGTTATACTTTCAATGTGTTT 2009

*msg*107 AGGTGCCAAATGGTCAGAAACAATAATATAAATCTACTCTCAATGTGCTT 1968

*msg*34 AAATGCAAAGTAGTCAGAAACAACAATATAAATCTTCTCTCAATGTGTTT 1968

*msg*110 AAATGCAAAGTGGTTAGAAACAACAGTATAAATCTTCTCTCAATGTGTTT 1956

*msg*8 AAATGCCAAATAGCTAGAAACAACACTATAGATATACTTGCATTATGTTT 1919

*msg*55 AACTGTCAAGCAGTTAGATACAACAGTATAGATATACTCTCAATGTGTCT 2001

* ** ** * *** **** * **** * * ** ** * ** *

*msg*53 GAAACCAAAAGAAACATGCAAGGCACTTGCAGAGGATGTAGAAAGAAAGA 2055

*msg*46 AAGGCCAGAAAAAACATGCAAAGCACTTGCAGAGGATGTAGAAAGAAAGA 2059

*msg*107 AAGACCAAAAGAGACATGTGAAGCACTTGCAGAGGATGTAAAACGAAAGA 2018

*msg*34 AAGGCCAAAAGAGACATGTGAAGTACTTGCAGAGGATGTAGAAAGAAAGA 2018

*msg*110 AAAGCCAAAAGAGACATGCGAAGCACTTGCAGAGGATGTAGAAAGAAAGA 2006

*msg*8 GAAGCCAGAAGAAGCATGCAAAATATTTGCAGAGGATGTAAAAAGAAAGA 1969

*msg*55 AAGACCAAAAGAAACATGCAAAGCACTTGCAGAAGATGTTAAAAAGAAAA 2051

* *** ** * **** * * ******* ***** ** ** *

*msg*53 GTCATCGTCTACGGCATATTTTAGATAAAACGAGGGATTATCCCCGAGAA 2105

*msg*46 GTCTTCGTCTACGACATATTTTAGACAAAACGAGAGATTATCCCCGAGAA 2109

*msg*107 GCTATCGTCTACGGCATGTTTTAGATAAAACCAGAGACTATCCCCGAGAA 2068

*msg*34 GTCATCGTCTACGGCATATTTTAGATAAAACGAGAGATTATCCCCGAGAA 2068

*msg*110 GTCATCGTCTGCGGCATGTTTTAGATAAAACAAGAGATTATCCCCGAGAA 2056

*msg*8 GTCTTCATTTACGACATGTTTTAGATAAAGTGAGAGATTATCCACAAGAA 2019

*msg*55 GTCTCGATTTACAACATATTTTAGATAAAACAAGAGATTATCCACGAGAA 2101

* * * * *** ******* *** ** ** ***** * ****

*msg*53 AAGGATTGTCTCGTTTTGGAGAAACAGTGCGAAGATCTGACAAAGGATTT 2155

*msg*46 AAGGATTGTCTCGTTTTGGAGAAACAATGTGAAGACCTGACAAAGGATTT 2159

*msg*107 AAGGATTGTCTCGTTTTGGAGAAACAGTGCGAAGATCTGACAAAGGATTT 2118

*msg*34 AAGGATTGTCTCGTTTTGGAGAAACAATGTGAAGACCTGACAAAGGATTT 2118

*msg*110 AAAGATTGTCTCGTTTTGGAGAAACAATGCGAAGACCTGACAAAGGATTT 2106

*msg*8 AAAGATTGTCTCGTTCTTGAGGAAAAGTGTGAAGACTTAACAAAGGATTT 2069

*msg*55 AATAATTGTATCATTTTAGAAAAAAAATGCAAAGATCTAACAAAAGATTT 2151

** ***** ** ** * ** ** * ** **** * ***** *****

*msg*53 CGAAGAGCTTAATGGTCCTTGCGCCACGCTAAAGAGGAATTGTGCTCATT 2205

*msg*46 TGAAGAGCTTAATGGTCCTTGCGCTACGCTAAAGAGGAATTGTGCTCATT 2209

*msg*107 TGAAGAGCTTAATGGTCCTTGTGCCACGCTAAAGAGGAATTGTGCTCATT 2168

*msg*34 TGAAGAGCTTAATGGTCCTTGCGCTACGCTAAAAAGGAATTGTGCTCATT 2168

*msg*110 CGAAGAGCTTAATGGACCTTGTGCCACACTAAAGAGGAATTGTGCTCATT 2156

*msg*8 CGAAGAACTTAATGGTCCTTGCACCACGCTAAAGAGGAATTGTGCTCATT 2119

*msg*55 TAATGAACTTAATGCTCCTTGTAATACACTAGAAATGCATTGTGCTCATT 2201

* ** ******* ***** ** *** * * * ************

*msg*53 TGAGGAACACAAAAGAAGTAAAAGACAATTTGTTGAGTAAAAATACAGAT 2255

*msg*46 TGAGGAACACGAAAGAAGTAAAAGACAATTTGTTGAGTAGAAATACAGAT 2259

*msg*107 TGAGGAACACGAAAGAAGTAAAAGACAGTTTGTTGAGTAAAAATACAGAT 2218

*msg*34 TGAGGAATACAAAAGAAGTAAAAGACAGTTTGTTGAGTAAAAATACAGAT 2218

*msg*110 TGAGGAACACGAAAGAAGTAAAAGACAGTTTGTTGAGTAAAAATACAGAT 2206

*msg*8 TGAGGAACGCGAAAAAACTAAAAGACAGTTTGTTAAGTAAAAATGCAGAT 2169

*msg*55 TAAGGAATACAAAAGAACTAAAAAAGATTTTATTAAGTAAAAATAGTGAT 2251

* ***** * *** ** ***** * * *** ** **** **** ***

*msg*53 ATTTTGGCAAACGTCGATAATTGTACAACATATTTGAATGTGAAGTGTCC 2305

*msg*46 ATTTTGGCAAACGTTGACAATTGCACAACATATTTAAATAGGAAATGTCC 2309

*msg*107 ATTTTGGCAAACGTCGATAATTGTACAACATATTTAAATAGTAAATGTCC 2268

*msg*34 ATTTTGGCAAACGTCGATAATTGCACAACATATTTAGATATGAAGTGTCC 2268

*msg*110 ATTTTGGCAAATGTTGATAATTGTACAACATATTTAAATAGAAAATGTCC 2256

*msg*8 ATTTTAGCAAACGTCGATAATTGCACAGCATATTTAAATATGAAGTGTCC 2219

*msg*55 ATTCTGGTAAATGTTAATAAATGTACAACATATTTGAATGTGAAGTGCCC 2301

*** * * *** ** * ** ** *** ******* ** ** ** **

*msg*53 TCGGTGGTTTAGGAGGGAAATAAATCCATTCAATCTTACATGTGTAGCAC 2355

*msg*46 TCGGTGGTTTAGGAGAGAAATAAATCCATTCAATCTTACATGTGTGGCGC 2359

*msg*107 TCGGTGGTTTAGGAGGGAAATAAATCCATTCAATCTTACATGTGTAGCAC 2318

*msg*34 TCGGTGGTTTAGGAAGGAAATAAATCCATTCAATCTTACATGTGTAGCAC 2318

*msg*110 TCGGTGGTTTAGGAGGGAAATAAATCCATTCAATCTTACATGTGTGGTGC 2306

*msg*8 TCGATGGCTTAAAAGGGAAACAAATCTGTTTAATCTTATATGTATAGAAC 2269

*msg*55 TCAATGGCTTAAAAGGAAAACAAATATGTTTACTCTTACATGTGTAGCAC 2351

** *** *** * *** **** ** * ***** **** * * *

*msg*53 ACCACAAATCATGCGTTATAATGATTGAAGAGGTACAAAATCATTGTTTG 2405

*msg*46 ATCACAAATCATGCGTTATAATGATTGAAGATGTGCAAAATCATTGCTTG 2409

*msg*107 ACCACAAATCATGTGTTATAATAATTGAAGACGTGCAAAATCATTGTTTG 2368

*msg*34 TCCACAAATCATGCGTTATAATGATTGAAGAGGTACAAAATCATTGTTTG 2368

*msg*110 ATCACAAATCATGCGTTATAATGACTGAAGAGGTGCAAAATCATTGTTTG 2356

*msg*8 ACCACAAATCGTGCATTATAATGACTGAAGACATGCAAAATCATTGTTCG 2319

*msg*55 ACTATAAAACATGTGTTATAATAGTTGAAGATATAAAGAATCATTGTTCT 2401

* *** * ** ******* ****** * * ******** *

*msg*53 GCATTTAAGGAAAATATGGAAAGTCATGATGTTATTAAAAAATCAGAAGA 2455

*msg*46 GCATTTAAAGAAAATGTGGAAAGTCATAATGTTATTAAAAAATCAGAAGA 2459

*msg*107 GCATTTAAGGAAAATATGGAAAGTCATGATGTTATTAA-AAATCAGATGG 2417

*msg*34 GCATTCCAACAAAATATGGAGGATCACAAAGTTATTGAAAAATCAAAAGA 2418

*msg*110 GCATTCCAACAAAATATGGAGGATCACAAAGTTATTGAAAAATCAAAAGA 2406

*msg*8 GCATTTAAAGAAAATATGAAAAGTCAAGATGTTGTTAAAAAATCAGAAGA 2369

*msg*55 GCACTCCAACAAAATATGAAAAACTATAAAGTTATTGAACAGTCGAATAA 2451

*** * * ***** ** * * * *** ** * * ** *

*msg*53 CAATGAA---AAAGATAACATTTGTTTTCTTTGGGGTGGATACTGCGATA 2502

*msg*46 CAATGAA---AAAGATAACGTTTGTTTTCTTTGGGGTGGATACTGCGATA 2506

*msg*107 CAATGAA---AAAGATAACGTTTGTTTTCTTTGGAGTGGATACTGCGATA 2464

*msg*34 AGATGAAGAAAGAGATAATATTTGTTTTCTTTGGGATGGATACTGCAATA 2468

*msg*110 AGATGAAGAAAGAGATAATATTTGTTTTCTTTGGGATGGATACTGCGATA 2456

*msg*8 CAGTGAAAAAAGGGGTGATATTTGTTTTTTTTGGGGTGGATACTGTGAAA 2419

*msg*55 TGAAGAA---AACGATAATATTTGTTTTCTTTGGGAAAAATACTGTGATA 2498

*** * * * * ******** ***** ****** * *

*msg*53 TGCTTGTGGAAAACTGTCCTGATAAACTAAAACAGGGCAATAACGGTGAA 2552

*msg*46 TGCTTGTGGAAAACTGTCCTGATAAACTAAAGCT---CGACAATGGCAAA 2553

*msg*107 TGCTTGTGGAAAACTGTCCTGATAAAC--AAACAGGGCAATAACGGTGAA 2512

*msg*34 TGCTTACAGGGAATTGTCCTGATAAACTAAAACAGGGCAATAATGGCGAA 2518

*msg*110 TGCTTACGGGGAATTGTCCTGATAAATTAAAACAAAGCTATAACGGCAAA 2506

*msg*8 TGTTTATGAAAAGTTGTCCTCATAAATTAGAACAAAATGATACTGACATA 2469

*msg*55 TGCTTATAAAAAATTGTCTTCACATGCCAAAACAAGGCAACAATGACTTA 2548

** ** * **** * * * * * * * * *

*msg*53 AATGGCCTCTGTGTGACTCTTAAGAAGAACTGTAAGACGTTTCACAAAGA 2602

*msg*46 GAAGGGGTTTGTATGAAGCTCAAAAAAAACTGTGAGACGTTTCGCGAAAA 2603

*msg*107 AATGGCCTCTGTGTGACTCTTAAGGAGAACTGTAAGACGTTTCACAAAGA 2562

*msg*34 GATGGTCTTTGCGTGAGTCTCAAGAAGAACTGTAAGGTGTTTCGCGAAAA 2568

*msg*110 AATGGCCTCTGTGTGACTCTTAAGGAGAACTGTAAGGTGTTTCGCGAAAA 2556

*msg*8 AATGGGCTTTGTGCAGAGCTCAAAAAAAATTGTAGGACATTTTATAAAAA 2519

*msg*55 AACAGACTTTGTTCAAAGCTCAAAAAAAATTGCAGAGTGGTTCGTGAGAA 2598

* * * ** ** ** * ** ** ** * *

*msg*53 ATTGCCTCTATTAAAAGCCCTCATGTACAATATAAAAGGTTCTTTAACAA 2652

*msg*46 ATTGCCTCTATTAAAAGCCCTTATGTACAATATAAAAGGTTCTTTAAAAG 2653

*msg*107 ATTGCCTCTATTAAAAGCCCTCATGTACAATATAAAAGGTTCTTTAAAAG 2612

*msg*34 ATTGCCTCTATTAAAAGCCCTTATGTACAATTTAAAAGGTTCTTTAAAAG 2618

*msg*110 ATTGCCTCTATTAAAAGCCCTTATGTACAATATAAAAGGTTCTTTAACAG 2606

*msg*8 AGAACTCTTATTGAAAGATCTTATGTACATTATGAAAGGTTTTTTGACTG 2569

*msg*55 AGAATCCTTATTGAAAGCCCTCGTATATAATATGAATAGCTCCTTGATAC 2648

* **** **** ** * ** * * * ** * * ** *

*msg*53 AAAAGGATGCTTGCATTAAGAAACTAAATGATTATTGTGCAAAATCAACA 2702

*msg*46 AAAAAGATGCTTGCATTATGAAACTAGATGATTATTGTACAAAATCAGCA 2703

*msg*107 AAAAAAATACTTGCATTAATAAACTAAATGATTATTGTACAAAATCAACA 2662

*msg*34 AAAAAGATACTTGCGTTAAGAAACTAAATGATTATTGTACGAAATCAGCA 2668

*msg*110 AAAAAAATATTTGCATTAATAAACTAAATGATTATTGTACAAAATCAGCA 2656

*msg*8 ATAAGAATGTTTGTAGTAAACAATTAAGTAGTTATTGTTCGAACTCAACA 2619

*msg*55 AAAAAAATGCATGTGTTCAGCAGTTAAATAACTATTGCACAAAGTTGACA 2698

* ** ** ** * * ** * ***** * ** * **

*msg*53 CACTCAAACAAGACTCTTGAAGATTCATGTAAAAAATATGGTAAAGATGA 2752

*msg*46 CACTCAAATAAGACTCTTGAAGATTCATGTAAAGAGTATAGTGGGAGCAA 2753

*msg*107 CACTCAAACAAGACTCTTGAAGATTCATGTAAAAAATATGGTAAAGACGA 2712

*msg*34 CACACAAACAAGACTCTTGAAGATTCATGTAAAGAATATAA---AGATGA 2715

*msg*110 CACTCAAATAAGACTCTAGAAAATTCATGCAAAGAATATAGTGAGAACAA 2706

*msg*8 CAATCAAATAAAACTCTTAAAAATTTATGTATAGAATATAA---AGATGA 2666

*msg*55 CAATCAAACGAAACTCTTAAAAACTTATGTCAGAAATATAACAAGAATGA 2748

** **** * ***** ** * * *** * *** *

*msg*53 AAACATAAAAAGTGAAACTTGTGAAAAATTTGTCAAATGGATGGAAATAT 2802

*msg*46 AGAAACAAGGGCTAAAACATGTGATAAACTTATTAGCTGGATGAAAATAT 2803

*msg*107 AAACACAAGAAGTGAAACTTGTGAAAAACTTGTCAAATGGATGGAAATAT 2762

*msg*34 AAAGACAAAAAGCGAAACTTGTGATAAACTTATTAGCTGGATGAAAATAT 2765

*msg*110 AGAAACAAGGGCTAAAACATGTGATAAACTTATTAGCTGGATGAAAATAT 2756

*msg*8 AA------AGGATAAGACATGCGATAGACTTGTCAATCGAACAAAAATAT 2710

*msg*55 A---ATAAGGGAAAAAACTTGTAATAAGTTCATCACTTGGATAGAAAAAA 2795

* * ** ** * * * * * * * *** *

*msg*53 TGTGCAATACCTTACCAGTCAAATTGGATAAAGCTGCTAAAGACTTGGAA 2852

*msg*46 TATGCAATGCCTTACCAGTCAAACTGGATAAAGCTGCTAAAGATTTGGAA 2853

*msg*107 TATGTGATACCTTGCCAGTTAAACTGAGTAAAGCAGCTAAAGACTTGGAA 2812

*msg*34 TATGCAATACCTTACCAGTCAAATTGGATAAAGCTGCTAAAGACTTGGAA 2815

*msg*110 TATGCAATACCTTACCAGTTAAACTGGGTAAAGCTGCTAAGGACTTAAAA 2806

*msg*8 TCTGTAATATATTTCTAATTAGGTTAGATAAAACTTCTAGTGACTTAGAA 2760

*msg*55 TCTGCATTACATATTCAACTAAACTGAATAAAGCAGCTAAAGACTTAAAA 2845

* ** * * * * * **** * *** ** ** **

*msg*53 AATAGAGCAAACGAATTTAAAAAAACCAAGCAAGAAACTGAAAAAGTCAT 2902

*msg*46 AATAGAGCAAATGAATTTAAAAAAACCAAGCAAGAAACTGAAAAAGCCAT 2903

*msg*107 AATAGAGCAAACGAATTTAAAAAAACCAAGCAAGAAACTGAAAAAGCTAT 2862

*msg*34 AATAGAGCAAACGAATTTAAAAAAACCAAGCAAGAAACTGAAAAAGCCAT 2865

*msg*110 AATAGAGCAAATGAATTTAAAAAAACCAAGCAAGAAACTGAAAAAGCTAT 2856

*msg*8 AATAGAGCATATGAGTTTAAAAAAATTAAAAAGCAAGCTGTAAAAGCTGT 2810

*msg*55 GATAAACTAGATGAATTCAAAGAAACTAAAAAATTGACAGAAAAAGCTGT 2895

*** * * * ** ** *** *** ** * * * ***** *

*msg*53 TAATGATTCTGGTTTATTCTTGGCAATTTCTCAAATAGCAGATGAG---A 2949

*msg*46 TAATGATTCTGGTTTATTCTTGGCAATTTCTCAAACAGCAGATGAGAAGA 2953

*msg*107 TAATGATTCTGGTTTATTCTTGACAATTTCTCAAACAGCAGATGGGAAGC 2912

*msg*34 TAATGATTCTGGTTTATTCTTGGCAATTTCTCAAACAGCAGATAAG---A 2912

*msg*110 TAATAATTCTGGTTTATTCTTGGCCATTCCTCAAACAGCAGATGGGAAGC 2906

*msg*8 TAGTGATTCGGGTTTACTTTTAGCAATTCCTCAAACAAAAGACAAA---C 2857

*msg*55 TAATGGTTCAGGTTTATTTTTAGGAATTCCCCCAACAAGAAACAGAAAAC 2945

** * *** ****** * ** *** * * ** * * *

*msg*53 AGAATCATCATCTT------------------CACAGCAATAACACGGCT 2981

*msg*46 AGAATCATCATCTT------------------CACAGCAATAACACGGCT 2985

*msg*107 AGAATCATCACCTTAGTGTACGTAGCAATATTTACAGCAATAACACGGCT 2962

*msg*34 AGAATCATCATCTT------------------CACAGCAATAACACGGCT 2944

*msg*110 AGAATCATCACCTTAGTGTACGTAGTAATATTTACAGCAATAACACGGCT 2956

*msg*8 AAAATCATTATCCTACT---------AATACGCATAGCAATATTACGGCT 2898

*msg*55 AAAATCATCATCTTAATATA------------CGTAGCAATATTGTAACC 2983

* ****** * * * ******* *

*msg*53 TATGTTAGACTTGTACGTCGCGAAAATGCCCTAGATATAGAGCCATCAGT 3031

*msg*46 TATGTTAGACTTGTGCGTCGTGAGGATGCTCCGGACATAGAGCCATCAGT 3035

*msg*107 TATGTTAGACTTCTACGTCGCGAGGATGCCCCGGACATAGAGCCATCAGT 3012

*msg*34 TATGTTAGACTTGTACGTCGCGAGGATGACTTGGACATAGAGCCATCAGT 2994

*msg*110 TATGTTAGACTTGTACGTCGCGAGGATGTCCTAGATATAGAGCCATCAGT 3006

*msg*8 TATATTAGGCTCATGCGTCGTAAAAACATTCTAAATTTACAACCGTCTAT 2948

*msg*55 CACATCAAGCATCTATTCAATGAAAATATCCTGGATATGCAAACATCAAT 3033

* * * * * * * * * * * ** *

*msg*53 GCGTCAAGGGTTAGCGTTTGATCTGGTGTCTTTACTTCTAGAGTTGTATT 3081

*msg*46 GCGTCAAGGGTTAGCGTTTGATCTGGTGTCTTTACTTATAGAGTTGTATT 3085

*msg*107 GCGTCAAGGGTTAGCGTTTGATCTGGTGTCTTTACTTATAGAGTTGTATT 3062

*msg*34 GCGTCAAGGGTTAGCGTTTGATCTGGTGTCTTTACTTGTAGAGTTATATT 3044

*msg*110 GCGTCAAGGATTAGCATTTGATCTGATGTATTTACTTGTAGAGTTGTATT 3056

*msg*8 ACGTCAAGGACTAGCATTTGATTTGATGTCTTTAGTTATAGAGCTGTATC 2998

*msg*55 GCATTATGGATTAGCATATAGTTTAATGTCTTTACTTGTAGACTTATATT 3083

* * * ** **** * * * * *** **** ** **** * ***

*msg*53 TGGAAGCCAAGGGCATCTGTAATCATTTTATCCAAGAGTGTGTCCTTGAA 3131

*msg*46 TAGAAGCCAAGGGCATCTGTGATCATTTTATCCAAGAGTGTGTCTTTGAA 3135

*msg*107 TAGAAGCCAAGGGCATCTGCAATCATTTTATCCAAGAGTGTGTCCTTGAA 3112

*msg*34 TAGAAGCCAAGGGCATCTGCAATCATTTTATCCAAGAGTGTCTCTTTGAA 3094

*msg*110 TGGAAGCCAAGGGCATCTGCAATCATTTTATCCAAGAGTGTATCTTTGAA 3106

*msg*8 TAGAAGCTAAAGGTATCTGCGATCATTTTATCCGAATGTGTGTCTTTGAG 3048

*msg*55 TGGAAGCTAAAGCTAGTTGTGATCATTTTATTGTAGAATGCTCTTTTGAA 3133

* ***** ** * * ** ********** * ** ****

*msg*53 GATGACTGTCCTAAGTTTAAAGACTCATGTAAGAAAATACATGAATCATG 3181

*msg*46 GATGACTGTCCTAAGTTTAAAGATTCATGTGGAAAAATACGTAAGTCTTG 3185

*msg*107 GATGGTTGTCCTAAGTTTAAAGATTCATGTGACAAAATACGTAAGTCTTG 3162

*msg*34 GATGATTGTCCTGAGTTTAAAGATTCATGTGAAAAAATACGTAAGTCTTG 3144

*msg*110 GATGACTGTCCTAAGTTTAAAGACTCATGTAAGAAAATACATGAATCATG 3156

*msg*8 AATGATTGTCCTAAGTTTAAGGATTCATGCAAAAAAATACACAATTATTG 3098

*msg*55 AACGATTGTCCTACTTTTAAAGATTTATGTGACAAAATACGCAAATTCTG 3183

* * ****** ***** ** * *** ******* * * **

*msg*53 TAAGGAATTTGTGTTGCCTGATACCAAGCCTC------------ATGTAA 3219

*msg*46 TGAGGCGTTTGTGTTGCCTGATGCCAAGCCTC------------GTGTAA 3223

*msg*107 CAAGGAATTTGTGTTGCCTGATGCCAAGCTTC------------GTGCAA 3200

*msg*34 TGAGGCGTTTGTGTTGCCTGATGCCAAGCCTC------------GTGTAA 3182

*msg*110 TAAGGAATTTGTGTTGCCTAATGCCAAGCCTC------------ATGTAA 3194

*msg*8 CAAAGAATTTGAATTACCTAAAGCTACACCTT------------TTGTAA 3136

*msg*55 TAGGGAGTTTCTGTTACCTAAAGCAATTTCTCTAACATTTGCATCTGCAT 3233

* *** ** *** * * * * ** *

*msg*53 CAACGTCTATATCAACAACGACACTTACAGAGTCAACAACTGTAGCGGAT 3269

*msg*46 CAACGTCTGTATCAACAACGACGCTTACAGAGTCAACAACTGTAACGGAT 3273

*msg*107 CAACGTCTATATCAACAACGACGCTTACAGAGTCAACAACTGTAACAGAT 3250

*msg*34 CAACGTCTATATCAACAACGACGCTTACCGAGTCAACAACTATAACGGAT 3232

*msg*110 CAACGTCTATATCAACAACGACACTTACAGAGTCAACAACTGTAGCGGAT 3244

*msg*8 CAACATCTATATCAACAATAACAATGACAGAATCAATAA---TAACAGGT 3183

*msg*55 CTACGTCTACATCAACAATAACAATTACAGATTCAACGACTATAAAGG-- 3281

* ** *** ******** ** * ** ** **** * ** *

*msg*53 ACTCAATCGGAATCTACTGTAGTAACTACAATGATAGAGG---GAAAGT- 3315

*msg*46 ACTCAATCGGAATCTACTGTAGCAACTACAATGATGGAAG---GAAAGT- 3319

*msg*107 ACTCAGTCAAAATCTACTGCAACAACTACAATCATGGAAG---GAAAAT- 3296

*msg*34 ACTCAGTCAGAATCTACTATAGCAACTACAGTGATGGAGG---GAAAGT- 3278

*msg*110 ACTCAATCGGAATCTACTGTAGTAACTACAATGATAGAGG---GAAAGT- 3290

*msg*8 AA------AGGATGTATGG------------------------------- 3196

*msg*55 -CCCATATAACAACTAATATGACAAAAACAATGAATGAGAAATGCACATC 3330

* **

*msg*53 --GTGTTGCGCTTCACTCAAAGACCACGTGGGTAACAAGCAAATCAACAT 3363

*msg*46 --GTGTTGCGCTTCATTCAAAGACCACGTGGGTAACAAGCAAATCAACAT 3367

*msg*107 --GTGTTGCGCTTCATTCAAAGACCACGTGGGTAACAAGCAAATCAACAT 3344

*msg*34 --GTGTTGCGCTTCATTCAAAGACCACGTGGGTAACAAGCAAATCAACAT 3326

*msg*110 --GTGTTGCGCTTCATTCAAAGACCACATGGGTAACAAGCAAATCAATAT 3338

*msg*8 --------TATATCATGTAAGGACCAGCCTAGTGATGCATAAATCAGCAT 3238

*msg*55 CTATACAAAAATTCATACAAAAATCAACTGGATGACAACTGAATCGATAT 3380

*** ** * ** * * **** **

*msg*53 CTACAAAAACAACTACAAC---TACATCAGTAACAACACTGACA-C--AA 3407

*msg*46 CTACAAAAACAACTACAAC---TACATCAGTAACAACACTGACA-C--AA 3411

*msg*107 CTACAAAAACAACTACAAC---TACATCAGTAACAACACTGACA-C--AA 3388

*msg*34 CTACAAAAACAACTACAAC---TACATCAGTAACAACACTGACA-C--AA 3370

*msg*110 CTACAAAAATAAC---------TACATCAGTAACAACACTAACA-C--AA 3376

*msg*8 CTACAAAAACAAAAATAAA---TACATTAGTGGCAACAGTTACA-C--AA 3282

*msg*55 TTACGGAAACAACTATATCATCAATAATAGTATCAACAGTATCAGCAAAA 3430

*** *** ** * * *** ***** * ** * **

*msg*53 AAATGCAAACCAGTACCATGTACAACGGAA---GAAACACAAACAAGAAA 3454

*msg*46 AAATGCAAGCCAGTACCATGTACAACGGAA---GAAACACAAACAAGAAA 3458

*msg*107 AAATGCAAGCCAGTACCATGTACAACGGAA---GAAACACAAACAAGAAA 3435

*msg*34 AAATGCAAACCAGTACCATGTACAACGGAA---GAAACACAAACAAGAAA 3417

*msg*110 AAATGCAAGCCAGTACCATGTACAACGGAA---GAAACACAAACAAGAAA 3423

*msg*8 AAGTGCAATCCAGTACCATGCATAATGGAAAAAGAGACACAAACAAAAAA 3332

*msg*55 GAATGCGAGCCAGCACCATGCATAAGAGAAAAAAAAACACAAGCAGAAGA 3480

* *** * **** ****** * ** *** * ****** ** * *

*msg*53 GCCTGAAACTAGAAATGAAGCAGATCATACAGTGATGCCAAACGAGGGGA 3504

*msg*46 GCCTGAAACTAGAAGTGAAACAGATCATACAGTGATGCCAAACGAGGGGA 3508

*msg*107 GCCTGAAACTAGAACTGAAGCAGATCATACAGTGATGCCAAACGAGGGGA 3485

*msg*34 GCCTGAAACTAGAAGTGAAACAGATCATACAGTGATGCCAAATGAGGGGA 3467

*msg*110 GCCTGAAACTAGAACTGAAGCAGATCATACAGTGATGCCAAACGAGGGGA 3473

*msg*8 AGTTAAACCTAGTGGGGAAACAAGTAATAAAAT---ATTAAATAAGGGAA 3379

*msg*55 ATTTGAAAATGAAGATGGAGCAAATAATATAGT---ACAGAATAAAGAAA 3527

* ** * * * ** * *** * * ** * * *

*msg*53 TAAAAATATCTGGATTGGGAGCAACGAGTATTGTTA---TATGG-GTAAT 3550

*msg*46 TAAAGATATCTGGATTGGGAGCAACGAGTATTGTTA---TATGG-GTAAT 3554

*msg*107 TAAAGATATCTGGATTGGGAGTAACGAGTATTGTTA---TATGGGGTAAT 3532

*msg*34 TAAAAATATCTGGATTGGGAACAACGAGTATTGTTA---TATGG-GTAAT 3513

*msg*110 TAAAAATATCTGGATTGGGAGCAACGAGTATTGTTA---TATGG-GTAAT 3519

*msg*8 TAAAAATATCTGGACTGGGAATAATAAATATTATTATTATATGG-ATAGT 3428

*msg*55 TAAAAATATCTGAATTAAAAGTAATGAATATTATTA---TATGG-TTAAT 3573

**** ******* * * * ** * **** *** ***** ** *

*msg*53 AGGAGTTTTTATTGTGATT**TAA** 3572

*msg*46 AGGAGTTTTTATTGTGATT**TAA** 3576

*msg*107 AGGAGTTTTTATTGTGATT**TAA** 3554

*msg*34 AGGAATTTTTGTTGTAATT**TAA** 3535

*msg*110 AGGAGTTTTTATTGTAATT**TAA** 3541

*msg*8 GGGGATCTTTGCTATAATT**TAA** 3450

*msg*55 AGGAATCTTTGTTTTAATT**TAG** 3595

** * *** * * *****

***msg*-IV**

**upstream potential TATA box**

msg77 AATTGAAAT--TAAA-GAATA**T--ATAAAA**TACATAAAATACATAAA--- 42

msg86 TATT---AT--TA------------------------------------- 8

msg80 ATT----AT--TA------------------------------------- 7

msg63 GA**TATATAT**AATATATGATGAAAGAGCAAATATATCTAAATCAATTA--- 47

msg70 TATT---AT--TT------------------------------------- 8

msg91 TT**TATAAAA**--TAAA-GAAAAA--ATAAACAA**TATATAA**TAAAAAAACTA 45

* * *

msg77 AATGTTATTATTATTTGAAACAAAATGCAGTTTAAGT**TATATTT**AGTGT**T** 92

msg86 -------------TTTAAAACAAAATACAATTTAAAT**TATATTT**AGTGT**T** 45

msg80 -------------TTTGAAATAAAATACAGTTTAAAT**TATATTT**AATATC 44

msg63 CATAATATTATTATTTGAAATAAAAGGTATTCTAAATTATACTTAATAT**T** 97

msg70 -------------TT--------------TTTTGTATTATAATT------ 25

msg91 AAGAA-AACA-TATC-TAAATAAAATACATTATATGTTAGATTTAATATC 92

* * * *** * **

msg77 **ATATTT**---TTCATACTATAACTTTTGTAAAAAATCAAATT----TTTT**T** 135

msg86 **ATATTT**---TTTATACTATAACTTTTACAAAAAATCAAGTT----TTTT**T** 88

msg80 GTATTT---TTCATACTATAACTTTTGTAAAAAATCAAATT----TTTT**T** 87

msg63 **ATATTT**TTTCTCGTACTATAATTTTTATAAAA-ATTAAGTA----TGGAA 142

msg70 ----TT---**TATAAAA**ATTAACTA-TGGAAAAACTTAAA----------- 56

msg91 T-TTTT---TTCATGTCCTAA-TTTCATAAAAGATTAATTAATTATAGAA 137

** *** * **** * **

msg77 --------**ATATTT**TTATAAAA---ATA-TTGTAACTAATTAGGAAACTA 173

msg86 --------**ATATTT**TTATAAAA---ATA-TTGTAACTAATTAGGAAACCA 126

msg80 --------**ATATTT**TTATAAAA---ATA-TTGTAACTAATTAGGAAAC-- 123

msg63 AAA--TTAACCTTTT--TAAAA---ATAATTAAAATTAATC------TTA 179

msg70 ----------CTTTTTAGAGAA---ATA-TTAAAATTAACTAGT---CTT 89

msg91 AAAGCTTAACCTTTTTAGAAACAAAATA-AATAAAATAATTA--AAATTA 184

**** * * *** ** ***

msg77 AAATT--AAAGATAATACCAATA-ATTTAACATCCAAT-AATCTGTATTT 219

msg86 AAATT--GAAGATAATATCAATA-ATTTAATATTCAAT-AATTTGTATTT 172

msg80 ----T--AAAGATAATATTAATA-ATTTAACATTCAAT-AATCTGTATTT 165

msg63 A---A--AAAGATAAAATCAATAGATTTACTAATCTATTTGTGTGTGCTT 224

msg70 A---A--AACGATAATATCAATAAATTTATCACTC----------TATTT 124

msg91 GTATTAAAAAGATAATGTTAATGAATTTACCATTCCAT-AACTTGTGTTT 233

* ***** *** ***** * * * **

**TATA box**

msg77 TTATTCTAAAATCTGAAACCAAATTTAATAATATTAAT-ACTAAATT**CAT** 268

msg86 TTATTCTAAAATCTCAAACCAAATTCAATAATATTAAT-ACTAAATT**CAT** 221

msg80 TTATTCTAAAATCTGAGACCAAATTTAATAATATTAAT-ACTAAATT**CAT** 214

msg63 TCAGTTTAAATCTTGAAGCTAATTTTAATTATGTTAAT-ACTAAA**TACAA** 273

msg70 TCAAATTAACAT--TAAATTAACTTTAATAA**TATTAAT**TATTCGACTCTT 172

msg91 TTAGTTTAAATTTTAAAGCCAACCTAAATAA**TAATACT**-GCTGAATT**CA**T 282

* * *** * ** * *** ** ** * * * *

**Cap signal**

msg77 **AATG**GAGACAA**CT**GAAGCGTATCG--TCTCTC-TAA**ATG**AAGGCTTTTGT 315

msg86 **AATG**GAGACAA**CA**GAAG**CA**TGTCG--TCTTTC-TAA**ATG**AAGACTTTTGT 268

msg80 **AATG**AAGACAA**CT**GAAATGTATCG--TCTCTC-TAA**ATG**AAGGTTTTTGT 261

msg63 AA----AA**CA**AA---GG**CA**TGTCGCTACCCTC-TAA**ATG**AAAATTTTTGT 315

msg70 **CA**TGA-AA**CA**A----A--TTGTCT----TTTA-TAA**ATG**AAAACCTCTAT 210

msg91 GA---AAAGAATTAAAAGTTACCA--TTTCTCTTAA**ATG**AAAGCCTTTGT 327

* * ** * * * ******** * * *

msg77 CTTTGTAATCTTTGTGATTTTAAGCACAATATCGTATGCTTTTTCAAAAG 365

msg86 CATTGTGATCTTTGTGATTTTAAGCACAATATCGTATGTTTTTTCGAAAG 318

msg80 TTTTGTTATCTTTGTGATTTTAAGCACAATATCGTATGCTTTTTCAAAAG 311

msg63 TTTG---------ACTATTTTAAGCACTATACCATGTGTTTTTTTAAAGA 356

msg70 CATT---------CTAACTTTAAGCATCATTTCGTCTGCTTTCTCAAAGA 251

msg91 CCTT---------GCAATTTTAAGCATCATATCATATGTTTTCTCAAAAG 368

* * ******** ** * * ** *** * **

**<------------------intron----------**

msg77 GCATTAATACTTATG**GTATTT**TTATTTTT-TTTTGTTTTTT---TTTTTT 411

msg86 GTATTAATACTTATG**GTATAT**T----TTT-TTTCGTTTT------TTTTT 357

msg80 GCATTAATACTTATG**GTATTT**TTATTCTT-TTTCGTTTTTTTT-TTTTTT 359

msg63 GCATTAACATACCAG**GTATTT**TTATCCTTTTTTTGTTTTTTTTGTTTTCT 406

msg70 GTGTTCCTGTTCATG**GTATTT**TCATCCTT-TTTTGTTTT------CTTTT 294

msg91 GCATTAGGATTCCTG**GTATTT**TTACTTCT-TTTTGTTGT------TTTTT 411

* ** ***** ** * *** *** * ** *

**--------------->**

msg77 AATTTTTATCTTT**TAG**AATCAAAAGAAAACTTAAATTTATTTACTTCAAA 461

msg86 AATTTTTATCTTT**TAG**AACCAAAAGAAAACTTAA-TTTATTTACTTCAAA 406

msg80 AATTTTTATCTTT**TAG**AACCAAAAGAAAACTTAAATTTATTT----CAAA 405

msg63 TTTTTTTATTTTA**TAG**AATTAAAAGAGAGCTTAAATTTATTTAAATCGGA 456

msg70 GATTTTCATTTTT**TAG**AATCAAATGAAAAATTATTTTTTTTTAATTTAGA 344

msg91 AATTTTTATCTTT**TAG**AATTAAAAGAAAACTTAAATTTATGTAAATCAG- 460

**** ** ** ***** *** ** * *** *** * *

msg77 AA-AAATTAGGAGTTATGGCCCATCAGTACATTCTGAATCAACTGATTCC 510

msg86 AAAAAATTAGGAGTTATGACCCATCAATATATTCTGAATCAACTGATTCC 456

msg80 AA-AAATTAGGAGTTATGGCCCATCAATATATCCTAAATCAACTGATTTC 454

msg63 ----AAATGGGAATTATAGTTCACCACTACATTCTACATTGGTTGATTTT 502

msg70 ----------AAACTATAGTTCATCTGATTATTTAACATCATTTCATA-- 382

msg91 ---------GGGATTGCAATTCATCAGCAGAATATCCATTACTTAATGTA 501

* ** * * ** * **

msg77 TTATCAAAACACTTACTATCTTCATCTGCATGGTTTATGGTTTTAGACGA 560

msg86 TTATCAAAACACTTACTATTTTCATCTTCATGGTTTATGGTTATGAAAAA 506

msg80 TTATCAAAACACTTACTATCTTCATCCGCATG-TTTATTGTTTTAGACGA 503

msg63 CTGTCAAAACGCTCACTACCATCGTTTTTATGGTCTAATATTAAGCAAGA 552

msg70 -TGTCAAAGCGTTCAAGTGCTTCATATACATTGTTTTCTTCTTTAAAGGA 431

msg91 CTATCAAAACGATCACTATTTTCATCACTATGGTCTAATATTAGACATGA 551

* ***** * * * ** * ** * * * * *

msg77 TGATACTTCTATGTATAGTCTTATCTTAGAAGAATCCATCAAAACTGAAA 610

msg86 CGATGTTTCTATGTACAGTCTTATTTTAGAAAAGTCTGTTACTACTGAAA 556

msg80 TGATGTTTCTATGTATAGTCTTATCTTAGAAGATTCTATCACAACTGAAA 553

msg63 TCATATTTCTTTATTTGTTTTGATTTTAAACATTTTTACACAGGATAAAC 602

msg70 TTCTATTGATATTTCTGCTTTAGTCTTAGGAGGCAATATAAATAATAGAG 481

msg91 TCCTCTTGTTTTATCTGCTCTTAATTTAGACGCTTCTATAAAAAATGAAG 601

* * * * * * * *** * *

msg77 GACAATGTAAATTATATTTAGAATATTTATGTGAAAAACTCGTAGAAAAG 660

msg86 TAGATTGTAAATCATATTTAGGATATTTATGTAAAATATTCATAAAAAAG 606

msg80 GACAATGTAAATTATATTTAGAATATTTATGTAAAAAATTCGTAGAAAAG 603

msg63 AGGAATGTAAATCATTTTTACAAGAGATATGTAATGATTTATTAAAAAAG 652

msg70 AACAATGTATAGATTTTCTAGAAAGTATTTGCGAGATTCTTTATCCGGAG 531

msg91 AAGAATGTCTATTACTCGTAAGAGAACTATGTGAAGGCCTCTTACCTAAG 651

* *** * ** * * ** * * **

msg77 CTACCAGGAGGAGGTTTTTCTAATATACTAAAAAGACTGTGCAACTTAG- 709

msg86 CTACCAAAAGGAAATTTTACTGATATACTAAAGAAATTATGTGACAAAA- 655

msg80 CTACCAGAAGGAGGTTTTTCTAATATACTAAAAAGACTGTGCAACTTAG- 652

msg63 CTATTAGAAGGAAGCTTTGTTGATATATTAAAAACAATTTGTACTTTAA- 701

msg70 TTATCAGAAGGACCCTTTACTAATATACTCAAGAAAATATGTGAAATTGA 581

msg91 CTGCCTCGAGGAGGCTTTACTAGTTTATTAAAAACATTTTGTGAATTGG- 700

* **** *** * * ** * ** * * * **

msg77 ATA--AAAGAAATACTTATTGCAAAGGTCTCTTTACTAAGACAAGTACTA 757

msg86 ATA--AAATAAATAAATATTGCAAAGGTTTATTTACTAAGG---GTGGTG 700

msg80 ATA--AAAGAAATACTTATTGTAAAGGTCTCTTTACTAATG---GTGATA 697

msg63 AAA--AAATAGATGCTCTT---CAAGATTTAA---CTGAATCAACTATTT 743

msg70 ACAAGGATTAAGTAAGCTTTGTCAATATTTAACTGCTAAATCTCGTCATA 631

msg91 CTG--AAGCAGATGATCTTTGCACACATTTAA---CTAAGTTGAAAA-AA 744

* * * * * * * ** *

msg77 TTGGAAAGGAC--CTACTT--ATAAAATGTAATGGTATAAAGGATAAATT 803

msg86 TTAAAGATGAC--TTACGT--AAAAAATGTGATGGTATAAAGAGTAAGCT 746

msg80 TTAAAAAAGAC--TTAAAT--AAAAAATGTAATGGTATAAAGGA-AAACT 742

msg63 CT-AAGCCTACTTCTATTCAAAGAAAATGTCAAGATTTAAATAGCAGACT 792

msg70 CT-AACTTTACACTGATTGAAAAAAAATGTCATGACCTTGAAAATG---- 676

msg91 TCAGGATTTACTCCTGTTCAAAGGAAATGTAATGATTTAAAAAATGAGCT 794

** * ****** * * * *

msg77 CAATTATTATTATTATATTTTAGGTA-AAACTTTATTTACAGGAGAATGG 852

msg86 CGAATATTATTATTA---TTTAGGTA-AAACTTTATTTACAGGAGAGTGG 792

msg80 CGATTATTATTATTA---TT--GGTG-AAGATTTATTTGTGGGAGAGAAG 786

msg63 TAATGAATTTTTTAA---TTCGAATG-AAGATTTGCTTTCAGAAGGTTCA 838

msg70 CATTAAGTGTTT--A---T---AATGATAACTTTAATC-TAA-AGAATGG 716

msg91 TTCTATGTTTTTTGG---TTCAAATG-ATGAATTACTTTTAGAAAGTCAA 840

* ** * * ** * *

msg77 A-ATGCAAGAATAGAAGCAGAAGATTGCGAAAATTATGAAACGTTGTGTA 901

msg86 A-ATGCAAGAATAGAAACAGAAGATTGTGAAAATTATGAAACGTTGTGTA 841

msg80 A-ATACAAGAATAGAAGCAGAGCACTGTGAAAATTATGGAACGTTATGTG 835

msg63 A-ACAAAAGACTAGGTATTGCTGATTGTGAAAATTATAGCACATATTGTA 887

msg70 GCCCGATAAACTAA--AAGGAAGA-TGTAACGAATTAGAAATATTATGTA 763

msg91 A-ATAAAAAACTAACTATTGGAGATTGTGAAAACTATGACACATTGTGTA 889

* * ** * * ** * * * * * ***

msg77 TAGTTTTTCAAAGAGTTTGCTATGGTTTGATTAGCGATTTATGTGGCAAA 951

msg86 TAGTTTTTCAAAGAATTTGCTATGGTTCGATTGGTAATTTATGTGCTAGA 891

msg80 CAGTTTTTCAAAGAATTTGTCGTGATTTAATTGGTGATTTATGTTCTAGA 885

msg63 ATATTTATCAAAGAATTTGTGGTATTGCTACTAGTAATTTATGTGGTGAA 937

msg70 ACATCTATGGACCCATTTGTGGTAGATCCATTTCTGTCCTTTGCAGTAAA 813

msg91 ATATTTTTCATAGAATTTGCGGTGATTCTACTCGTGATTTATGTACTAGG 939

* * * **** * * * * **

msg77 TTTAATAATCTCTGTTATAAAAAATATATTAAAGATACTGAAGATAGTGT 1001

msg86 TTTAATGGGCTTTGTTATAAAAAATATACTAAAGATGCTGAAGATGGCTT 941

msg80 TTTAATGGACTTTGTTATAAAAAATATACAAAAGATAATGAAAATAATAT 935

msg63 CTTCAAAATGCCTGTTATTTAAAATATCGTGAAAACTTTAGAAATACAAT 987

msg70 ATTTCTGCTGATTGTTTTGATTTACGTTATAAAGACCATCAAAATTCACT 863

msg91 TTTAAATCTATGTGTTATTCAAAACTGCGTGAAGATTTTAAAAAGGCACT 989

** **** * * ** * * * * *

msg77 ATTATTGGAATTTATAGGAAAAGAAGCACTATCTAATGAATCGAAAAATA 1051

msg86 ATTATTGAAATTTATAGGAAAAAAGACACTATATAATGAACTGGAAGATA 991

msg80 GTTATTGAAATTTATAGGTGAAAAAAT---AAGTAGTGAATTATCACATA 982

msg63 TTTATCTTTAATTTTA-----AAAGATAGTCT-TAAAG---TCTC----- 1023

msg70 TTTACTTCAGGTCTTG-----CGGGATGATCT-TAATG------------ 895

msg91 CGTATTGGCACTAGTA-----AAAGATAGCAT-CCA-------------- 1019

** * *

msg77 ATGAACTGGAACATGA---ACATC---TATATACCCAATGTATTCACACT 1095

msg86 ATAAATCGGAACATGA---ATATA---TATATACCAAATGTATTCACTCT 1035

msg80 ATAATCTAAAACATGATGAATCTGAAGAATATAGTCAATGTTTTGACAGT 1032

msg63 -TACTCTTGAAAATCA---CCA------------AGAGTGTGTTGATGAC 1057

msg70 ---ATCCTGAG------------------------GTTTGCCGTAAATCG 918

msg91 -TAGACGAG------A------------------AGAGTGTGTTACCGAC 1044

** *

msg77 CTTCTTAAAAAATGTCATTGTATAACCTCTTTTGGGCCTACTATGGCAGA 1145

msg86 CTTCTTGAAAAATGTCATCGTATAACTTCTTTTGGGCCTACTATGGTAGA 1085

msg80 CTTCTTGACAAATGTCATTGTGTAACCTCTTTCGGGCCTACTATGGTAAA 1082

msg63 CTTTGTCGCAAATGCTTTTGTGTGATCGGTCTTGGTCCTGATATTGTTGA 1107

msg70 TTTCATAATAAATGTCTATGTCTTAGTACCCTTGCTCCTACAATGGCTGA 968

msg91 CTTCTTAAAAAATGTCATTGTCTAGTTGGTTTAGGTCCTGATATGGTGGA 1094

** * ***** ** * * * *** ** * *

msg77 ATCATGTTCTCAATTAACGGATACATGTAATCACTTTAATCGAAGTACTA 1195

msg86 ATCATGTTATGAACTAACGGATACATGTAACCGCTTTAATCAAACTACAA 1135

msg80 ATCTTGTTTTGAAATACAGGATACATGTAAACACTTTGCTCAAAGTATTA 1132

msg63 AGCATGTCTTCAAGTCTGGAGTACATGTAAATCTCTTGTAGAAGCTATTG 1157

msg70 TGTCTGTCTTCTTGTAGAGGCTACATGTGAAAATCTTATAGAACATAAAG 1018

msg91 AGCATGTCTTCAAGTATGGAGAACATGCGAAACTACCGCAAAAACTACTA 1144

*** * * * ***** * * **

msg77 CCATAACTTGTCGATATTTGGACTC--TTAT--ATTAAAAA--TTTTC-- 1237

msg86 TCTCAACTTGTCGATATTTGGGTTC--TTAT--ATTGAAGA--ATTTC-- 1177

msg80 TCTTTACTTGTCGATGTTTTGATTA--TTAT--ATTAAAGA--ATTTC-- 1174

msg63 ATGAAACTTGTCTGTATTTCAATCT--TTAT--ATCCAAAA--CTCAC-- 1199

msg70 AACAGAAATGTTCAGCTCTTAAATCTGTTATTTATGAAATAACCTCAGAT 1068

msg91 GCGAGATTTGTTCACAACTCAGCTC--TTCT--ATTAAAAA--ATCAT-- 1186

* *** * ** * ** ** * *

msg77 -TCTTAGAAAAACAGGAACATATTACAGG--TTATAAGAAAAAGATAACA 1284

msg86 -TCTCGGAGAAACAAGAGCATATCACAGG--TTATGAGGAAAAGATAACA 1224

msg80 -TCTCAGAGGATCT---CCATATTACAGG--CTATAAGAAAGATATAGCG 1218

msg63 -TTTCAAGAAATTTAAAACATACCTCAGG--TTATGGGCGCACATCATCA 1246

msg70 GGTTTAGAAAATTTAGAAGAGTCTTTGAGTGCTTCAGGCA--ACACATTG 1116

msg91 -TTTTAAAGAATTTAGAATATACTTCAGA--TTATACGCATGAATCATTA 1233

* * * * * *

msg77 AGAAATAACTGTTCGTTACTCGGTGCTTGTGAGTATTAT---TCATTACT 1331

msg86 AGAAATAACTGTTCGTTGCTCGGAGCTTGTGACTATCAT---TTATTACT 1271

msg80 AGAAATAATTGCTCATTGCTTGATGTTTGTGAGCAATATATCTTATTACA 1268

msg63 GAAAAGGAATGTTTATTGGTTACTTATTGTGATTATTAT---ACATCATT 1293

msg70 GAAACCGATTGCACGTCGTTTCCTCCTTGTATTGATTTT---TTATCATA 1163

msg91 GAAAGTAGATGTTCATTGATCAGTACTTGTGATTATTAT---ACGTTGTT 1280

** ** * * **** * * *

msg77 CTGTCATAACAAAACAACAAAGAATCTTTGCAAGCCAATAATAGAAGAAT 1381

msg86 CTGTCATAACAAAACAACAGAGGGTCTTTGCAAGCCAATAATAAAAGAAT 1321

msg80 TTGTCATACCCAGACAACAAAGAATCTTTGCAACTTAATAATAAAAGAAT 1318

msg63 TTGTGGTGACTATACAACAAAAATTCTTTGCGTTGAATTAAAACAAAGAT 1343

msg70 CTGTGATAATTTAGAAACAAGAAAGCATTGTCAACATATAAAACAAAAAT 1213

msg91 TTGTGGTAATTCTGAAACAAAGAATCTTTGTAGTACATTGAAAAAAGAAT 1330

*** * **** * *** * * * ** **

msg77 GCTCCGCAAAAGCTGATCTAGATATGGGCTTCGTAAAGAATCTTTCTTTA 1431

msg86 GCTCCGCAAAAGTTGATCTAGAGATGGACTTTGTAGAGAAATTTTTTTTA 1371

msg80 GTACTAAAAAAATTGATCCAGATATGCTGTTTATAAACAACATTTCTTTA 1368

msg63 GTACTGGAAGGGC---AGTGGATATGCCATTTGTTAAAAAAATTGAATTG 1390

msg70 GCACTCAAGA---TAATTTAACTGAGTATTCGACGACCAAAGTTAGTCTA 1260

msg91 GCATCGGAAG---TAACTCGGATATCTATTCTACAGGAATGAATCTATTA 1377

* * * * * *

msg77 GGCAAATGCAAATCAACGTTTGAAAGCGTAAATTTAACTAGATTTTTTTA 1481

msg86 GGTGAATGCAAATCAACGTTTCAAAACGTAAATTTAACTAGATTTTTTTA 1421

msg80 GGAGAATGTAAATCGACATTTGAAAACGTAAATTTGACTTTGTCTTTTTT 1418

msg63 GGTAAATGTAGTTCAGTATTTGAAAATGTAAATTTGGATTCGTTTTTTTC 1440

msg70 GGTGATTGTGTATCAGCTTATAGTAGTGTGGATTTGGATGCTTTTTTTCA 1310

msg91 GGCCAATGTGCACACGAATTCAAAGAAGTAGATTTGAGTGCGTTTTTTTC 1427

** * ** * ** **** * * ****

msg77 TGAAAAAGAAAAAAGTGGAGTTTTATTACCGCATAAAGCTCCATATCTTA 1531

msg86 TGAAAAACAAAAAAGTGGAGTTTCATTACCGCATAAAGCTCCATATCTTA 1471

msg80 TAACGAAAGAGAAAATAGCGTTTCATTACCTTATAAAAATCCATATCTTA 1468

msg63 TCAAACACAAAAAAGTGGTATTTCATTTCCTTATAAACATCCATATCTTT 1490

msg70 TAAATTACATGATAATCATCTTCTATTTCCATTTGGATATCCGAGTCTTC 1360

msg91 TAGAGAAAGAGAAAATGGTGGTTTATTTCCTTATAAACCTCCATATCTCT 1477

* * * * * * *** ** * * *** ***

msg77 CTCCTCTTTTAATGTTTTTCTCTTCCCTAGGAAGATCTACTCTTACTTTA 1581

msg86 CTCCTCTTTTAATGTTTTTTTCTTCCCTAGGAAGATCTACTTTTACTTTA 1521

msg80 CTCTTCTTATAATATTCGGGGCTTCCTTAATGAGGCGTGGTTCTAGTTTA 1518

msg63 CTACTATTATACTATTTATATCCTCTTTAATGTCACATAGGGGTGATTTA 1540

msg70 GTACTATTTTGTTATTTCTAGTTTCGTTAATGCAACATCGTGGTAATATG 1410

msg91 TTCCTCTTATAACCCTTATATCATCTTCGATGGCACATCTCACCAACTTA 1527

* * ** * * ** * *

msg77 AAGGAAAGATGCTTGA-ATTTTATTA-AAAAAAATTGTTTGGCTCTTCAG 1629

msg86 GAGAAAAGATGTTTGA-GTTTTATTA-AAGAAAATTGTATGGCTCTTTAT 1569

msg80 AACGAAAAATGTATGA-ACTTTCTTA-AACATGATTGTACTAATCATCAA 1566

msg63 CAGGAAAAATGTAGAA-TATTTCTTG-AACATTACTGTGACTATTATCAA 1588

msg70 CAAGTTAGATGCCAGTCATTTTCTTC-TAGA-TATTGTCAATATTATAGG 1458

msg91 GAGGGGAGGTGCGG-A-ATATTCTTAGAACAATCTTGCAATTATTACGAA 1575

* * ** ** ** * * ** *

msg77 GGTATGTTTCCTAATCTTCATGAATATTGTAAGAGTGGATATACTGATGA 1679

msg86 GGTATGTTTTCTAATCTTCATGAATATTGTAAGAGTAGATATACTGATGA 1619

msg80 CGTGTATTTCCTAATCTTGATGCATATTGTCAAAGTGGACAAACTGATGA 1616

msg63 ACGACGTTTCTTAATCTTGATAAATATTGTAGTGAAGGCTATAATGAAGA 1638

msg70 GGGGTATTTCCTGTTCTGCATAGTCACTGTAATAGCG---ATTTTTCGGA 1505

msg91 ACTGTGTTTCCTAATCTTGACGAATATTGTAATACAAGGCAGACTGATGA 1625

*** * *** * * *** * * **

msg77 ATGCGAACAATTGGATGAAAAGATGAATACAACATGTATCAATTTAAATA 1729

msg86 ATGTAATGATATGGATGGAAGGATGAATACAACATGTGTCAATTTAAACA 1669

msg80 GTGCGATAATCTGAATAAAAGCATCAATAAAACATGTGTCAATTTAAACA 1666

msg63 ATGTGAACATTTAAATTCTAAAACAGAAGAATCATGTAATAATCTAAGAA 1688

msg70 ATGTGACAACTTAGATACTAAAGCAACTAAAGCTTGTGCCGACTTGAAGA 1555

msg91 ATGTAGCAACTTGGATGCTAAAGCAAGTAGAGCGTGTACTCGTTTGAAAG 1675

** * * ** * * * *** * *

msg77 AAACATTTGAAGAATTAGGTCTTACTTCTACTAATGGCGTTTGGACTATA 1779

msg86 AAACATTTGAAGATTTAGGTCTTACTTCTACAAAAGGTGTTTGGACTATA 1719

msg80 GAACATTTGATGATTTAGGTCTTGTTTCTGCAAATGGTGTTTGGACTATA 1716

msg63 ATGTAGCTCAGGGGCTAGGTCTTGTTTCTGGGCAAGGCATTTCCGTTATA 1738

msg70 AAAAGCTTCATGAATTAGGGCTTGTTCGTGAAGACGGTGATTCCCCTAAA 1605

msg91 ATAGATTAGAGGATTTAGGATT---TTCAGAAAACGGGTCACCCGTTATG 1722

* * **** * * * ** **

msg77 CTATGGAACAGCACAGCAAAC---AATATCACTACACCCCAATGTCAAAT 1826

msg86 CTATGGAACAGTACAGCAAAC---ATTATCACTACACCTCAATGTCAAAT 1766

msg80 CTATGGAACAGCACTGCAAAC---ATTATCACTACACCTCAATGTCAAAT 1763

msg63 ATGCACAGTGGCATGCTAAGCGATCATATGACCGTATCTCAATGTTTATC 1788

msg70 TTATACCGTGGTGCGCATCACGATTCTATTTCTGTGGGTGAATGTGCAAC 1655

msg91 CTATCAAGCGATATAATGGATAGTCGTGTCACTGTAGCTCAATGTGTAGT 1772

* * * * ***** *

msg77 GTTAATAGAAGAATGCACTTATTTTAGACCTGGTTGTCCTGGTATTAAAA 1876

msg86 GTTTATGGAAGAATGCATTTATTTTGAACATGTTTGTTCTGGTATTAAAA 1816

msg80 GTTAATAGAAGAATGCACTTATTTTGAACATGCATGTCTTGGTATTAAAA 1813

msg63 ACTTATACATCAATGTGTTTATTTTGCACATATTTGTGAAAATATTCAAG 1838

msg70 TTTAATAGCAGAATGTCACTATTATAGCTATTTATGTCCTGAACTTAGAG 1705

msg91 ACTTATACAAGAGTGTGTCTACTTTTCACATGTATGTTCTGGTATTCGAC 1822

* ** * ** ** * * * *** ** *

msg77 GTCCATGTGAAAATGTAAAAGCTCTATGTTATACACTGAGTAAAAAAAGA 1926

msg86 GTCTATGTGAAAATGTAAAAGCTCTATGTTATACGCTGGGTGTGAAAAGA 1866

msg80 GTCCATGTGAAAATGTAAAAGCTCTATGTTATACGCTGGGTATAAAAAGA 1863

msg63 GTATATGCAAAAATGTAAGGGTTACATGTTATAATTTGGCTATACAAAAA 1888

msg70 GCCCATGCAGACATTTACATCCTCTATGTTATAATCATAATAAAGAGAAA 1755

msg91 ATCTATGTGAAGAATTGAAGGTTGTATGCCAAGAATTAGGTATACAAAGA 1872

*** * * * * *** * * * * *

msg77 TCTCACTTAAATTATTTTTGGGAAAAACTCGAAGAAAAATTATCACCTAG 1976

msg86 TTTCACTTGAATAATTTTTGGAAAAAACTCAAAGAAAAATTATCACCCAG 1916

msg80 TCTCACTTGAATAATGTTTGGAAAAAACTCAAAGAAAAAATACCATCTAC 1913

msg63 CATTATATGAGTACCTTTTGGGGAAAATTCAATAAAAATATATTATTGGC 1938

msg70 TATTATTTGGGTAGATTTTGGGATAAAATAAAAGAACACATAACATCAAA 1805

msg91 TATAGCTTAGGTCTTTTTTGGAAAGAATTTGAGAAACGCTTGCCACTTGA 1922

* * * ***** ** * * ** * *

msg77 TGATTTTAGCATTTTTAATCAATCTAATCTACCTCAATCTTCAGCTGCTT 2026

msg86 TGATTTTAGCATTTTTAATCAATCTCATCTACCTCAATCTTCTGATACTT 1966

msg80 TGATTTTAGCATTTTTAATCAATCTCATCTATCTCTGTCCTCTGATATTT 1963

msg63 TGGTCCTAATAATTTTAATCTTAG------ACTTCTATTGACTGAAAATT 1982

msg70 AGGTTTTAGGACTGCAGATCTTTTACATCCA---AAAACCTCTGGTGA-- 1850

msg91 TACTTTTAGTGCATCTAATATTAA------ACATCAGTTGTCACATGAAT 1966

* ** ** * *

msg77 CTAATTTATATCAGCATATATACAAGCATATTCTAGATATATGTGCTGAG 2076

msg86 CTAAGTTGC------------ACCAGCCTATTCTAGATATATGTGCTGAG 2004

msg80 CTAATTTAT------------ACAAGCCTATTTTAGATATATGTGCTGAG 2001

msg63 CTGGAATAC------------TTGATCATATTATGACTATCTGTTCTGAG 2020

msg70 ----ACTAT------------ATCCATATATTTTAAAATTATGCTCTACT 1884

msg91 CTAATTTAC---------------ATCATATTCTAACAGTATGTGCAGAA 2001

* **** * * ** *

msg77 CTTGGAGGAACTAACGAGGTCTTATTTAGGTGGTGTTTGCACCCTATCCC 2126

msg86 CTTGGAGGAACTAACGAGGTCTTATTTAGGTGGTGTTTGCACCCTATCCC 2054

msg80 TTTGGAGGAACTAACGAGGTCTTATTTAGATGGTGTTTGTACCCTACTCC 2051

msg63 TTAGGAGGAAGTAATGAGGTGATATTTACATGGTGTTTGTATCCTGCGAC 2070

msg70 CACGGTACCACAAATGAGTTATTTTTTCAATGGTGTTTGCATCCCTTAAA 1934

msg91 TTAGGAGGACTTAATGAAATATTATATAGATGGTGTTTACACCCTTCTAA 2051

** ** ** * * * * ******** * **

msg77 TATCACTCCCCCCGCCCCGATAATTTTCCCAGGTAACTTACTTGATAGAT 2176

msg86 TATCACTCCCCCCGCCCCGATAATTTTCCCAGGTAACTTACTTGATAGAT 2104

msg80 TAATGACCCC-----CCCGGTAACTTCCCCAGG-GACTTACTTGATAGAT 2095

msg63 TAT---TC-------CTCGAAAACTT-----------------T----A- 2088

msg70 TAT---TC-------CTCGAATGTTT-----------------T----A- 1952

msg91 GTT---TA-------CTGAGAGACTT-----------------G----A- 2069

* ** *

msg77 TACTTTATGATTTTTCAGCAAGGCGTGATGATTTAGAAAGAGGCTTATTA 2226

msg86 TACTTTATGATTTTTCAGCAAGGCATAGTGATTTAAAGAGAGGCTTACTA 2154

msg80 TATTTCATGATTTTTCAGCAAGACACGAGGATTTAAAAAGAGGCTTACTA 2145

msg63 ----TTATTATCTTGTAGAAGGATACGAGGAATTAGAAAAGGACTTACCA 2134

msg70 ----TTATTCTTTTAAATATGGTTCTATTGATTTACTAAGTAATTCATCG 1998

msg91 ----TTATTATTTATCACTAGGATATAAGGGCTTAATTCAAGACCTGCCA 2115

* ** * * * * * ***

msg77 TATGTAGGAGAACCTCCTAGTTTAGCAGAATGTGCACCTTATGTTTATGA 2276

msg86 TATGTATCAGAGCCTCCTACTTTAACGGAATGTGCATCTTATGTTTATGA 2204

msg80 TATGTATCAGAACCTCCTACTTTAACGGAATGTGCATCTTATCTTTATGA 2195

msg63 AAACCTAATGAACAGCCTTCTTTGTCTGAATGTATATATTATATTGCTGA 2184

msg70 CATGAGAATGAAAAGCTATCTTTACCTGAATGTGCGTTTTATTCTTTCCA 2048

msg91 GAAGTGACTCAAAAACCTTCTGTATCAGAATGTATGTATTATACTGAGGA 2165

* * * * * * ****** **** * *

msg77 ATGTCATAACTTATTAGAGATTTTTAAAGATCTTACTACTAATTGTACAA 2326

msg86 ATGTCACAGTTTATCAGACATTTTTAAGAATATTACT---AATTGTACAA 2251

msg80 ATGTCACAGCTTATCAAACATTTTTAAAGATCTTACT---AATTGTACAA 2242

msg63 ATGTAACGGTTTATCTAATATTTTTCCTAAACTTAAAGATTCATGTACAA 2234

msg70 ATGCA---GTCAATTTACTGTTTTTGATGATTCTAAGCATACGTGCGATA 2095

msg91 ATGTGATCTTTTAATTGCTGTTTTTGGTGATCCTAACGGTTTATGTGAAA 2215

*** * ***** * ** ** *

msg77 TACTGGAAAATGAATGTTATAATGGGTACAAAGACTATGATAAAC----- 2371

msg86 TGCTGGAAGAAGCATGTTACAGTGGTCACATAAACTATAGTAAAC----- 2296

msg80 TGTTGGAAGAAGCATGTTATAGTGGTCACAAAAATTATGGTAAAC----- 2287

msg63 GGCTCGAAAACAAATGTTTTG---GTTATAATTATAAAGATAGAT----- 2276

msg70 AGGTGAACGAAATATGTTTTCCTCAAGATGATTTTCAATATTCTTTCGGA 2145

msg91 AGTTAAAAGACTTATGTTATAA---ATATGATCTTAAAAATGATT----- 2257

* * * ***** * * *

msg77 ----TTGAAAATTCTAAGCTTATTAAGTCGATT**TGA** 2403

msg86 ----TTAAAGATTCTACGCTTAGTAAGTCGGCT**TGA** 2328

msg80 ----TTAAAGATTTTAAGTTTAGTAAGTCGACT**TGA** 2319

msg63 ----TTGAAAATATTGAGCCTGTTAAATTGACA**TGA** 2308

msg70 AATGACACAGATTTTGGATTTACTAAA---AAT**TGA** 2178

msg91 ----TTAAGGAGCTTGAGTCTATTAAA------**TAA** 2283

* * * *** * *

***msg*-V**

**upstream potential TATA box**

*msg*101 TATTAT---AGTATT------CTA-------GATAACATA---AACTATT 31

*msg*54 TAATAT---TT-----------TA-------GATAACATA---AACTATT 26

*msg*47 TTAGAT----------------------------AATATA---AACTATT 19

*msg*28 AATTAT---TTTATT-----AC**TATATTT**TGAATAAACAA---AAAAATT 39

*msg*97 TGCAAAAA-GCTATTCAAACAAAATAAA-AGTATTATTAA---AATTTTT 45

*msg*22 CTATTC-----AA-----ACAAAATAAA-AGTATTATTAA---AATTTTT 36

*msg*11 ATTTTT-----TATT-AATTATATCATTTTAAGTGAAATAAGGAATTACC 44

*msg*42 CAAAATACTTTTATT-AATCATCATATCTTCAATAAAACA---AATTATT 46

* * **

*msg*101 AAGAATTTTCTAAATAACCTTCTAAACT--AAGAAACTTAAAAGTCAATA 79

*msg*54 AAAAATTTTCTAAATAACCTTCTAAACT--AGGGAACTTAAAAATCAATA 74

*msg*47 AAAAATTTTCTAAATAACCTTCTAGACT--AGGCAACTTAAAAATTAATA 67

*msg*28 AAA-AT-TTTTATATACTGTTCCAG------GAGAAATTAAAAATCAAAA 81

*msg*97 AACTAATTTTCAAGTAGTCTTCTAAATT--AAATAACTTAAAAATTAGCA 93

*msg*22 AACTAATTTTCAAGTAGTCTTCTAAATT--AAATAACTTAAAAATTAGCA 84

*msg*11 AAAAATTTTATAACTA--ATTTT-AACT--AAATAACTTA--AATTAACA 87

*msg*42 ATGAATTTTATGATCATATTTTTAAACTATAAGTAATTTAAAAATTAGCG 96

* * ** * ** ** *** * * *

*msg*101 TTTTAAAA-TAGATTATTG----AAAATAGA---ACAA-AAGGCATATT- 119

*msg*54 TTTTAAAA-TAGATTACTG----AAAATAGA---ACAA-AAGGCATATT- 114

*msg*47 TTTTAAAA-TAGATTACTG----AAAATAGA---ACAA-AAGGCATATT- 107

*msg*28 TTTTAAAT-T----TAC-A----AGAACAAG---ATAA-TAA-TATTAC- 115

*msg*97 TTTTAAAA-TAGATTCA-T----AAAATAGATTCATAAAAATAAATATTT 137

*msg*22 TTTTAAAA-TAGATTTA-T----AAAATAGATTCATAAAAATAAATATTT 128

*msg*11 TTTTAAAAACAAATC--T-GC-AAGAATAA------AGGAATAACTATCT 127

*msg*42 TTTTAGAA-TAAATTTAATCTAGTAAATAAT---TCAAAAATAACTAGAA 142

***** * ** * * * *

*msg*101 -GGAAACTATTGAAATAAATAATCAAGT----TATAGATATATATCTATA 164

*msg*54 -GGAAACTATTGAAATAAATAATTAAGT----TATAGATATATATGTATA 159

*msg*47 -GGAAACTGTTGAAATAAATAATTAAGT----TATAGATATATATGTATA 152

*msg*28 -GGAAATTATCAAAGTAAATAATTATAATATTTATAGAAATAATCTTACC 164

*msg*97 CAAAAAATATTAAAACAAATGAA**TATATAA**----**TATATAA**AAATA-GCA 182

*msg*22 CAAAAAATATTAAAACAAATGAA**TATATAA**----**TATATAA**AAATA-GCA 173

*msg*11 CACATAA----AAAATAAACAACTATGG----**TATATTT**--------AGA 161

*msg*42 TAAATTATACTATACTAAATTATTACGC----**TATAAAA**ATAT-TATCTA 187

* * *** * * ***

*msg*101 AGAATAATATTTTTTATT---TTTTA----ACTAATA---T-------AC 197

*msg*54 AGAATAATATTTTTTATT---TTTTA----ACTAATA---T-------AT 192

*msg*47 AGAATAATATTTTTTATT---TTTTA----ACTAATA---T-------AT 185

*msg*28 TATTTACTATT-ATTATCTGTTATTA----ACTAATTATTT-------AT 202

*msg*97 TTATTTGTTTATTGTATA---TTTTA----ACTAATT---T-------TC 215

*msg*22 TTATTTGTTTATTGTATA---TTTTA----ACTAATT---T-------TC 206

*msg*11 AATAATATCTCTGTTATT---TCTTAT-CGAGTAGTT---T-------CT 197

*msg*42 ATTATTA-----AACATC---GATTATCTTAATATTT---CTGCGAATCA 226

** *** * ** *

*msg*101 TAGTA---AATAATTTTTT-TGATATCGTAA----CTTTTTATCT--ATT 237

*msg*54 TAGTA---AATAATTTTTT-TAATATCGTAA----CTTTTTATCT--ATT 232

*msg*47 TAGTA---AATAATTTTTT-TGATATCGTAA----CTTTTTATCT--ATT 225

*msg*28 TTATT---ACGAATATTTT-CAATATCGGAA----TCCTCTATCCGTAAA 244

*msg*97 TTTCTACGAATCCCCTTTT-TAATATTGCCC----CTTTTTATCCGCATT 260

*msg*22 TTTCTACGAATCCCCTTTT-TAATATTGCCC----CTTTTTATCCGCATT 251

*msg*11 TATATACAAATA----ATT-CGGTATCAGCATATCTTTTTTATTTACGTT 242

*msg*42 TTTCTGCGAATCATTTTT**TATAATG**TTT---TAGCCTTTCTGTC--TA-T 270

* * ** * * * * *

**TATA box Cap signal**

*msg*101 TTCAAATCAATTG**TATATTA**GAAAATAAGATAAATTTATTTATT**CA**TTTA 287

*msg*54 TTCAAATCAATAG**TATATTA**GAAAATAAGATAAATT**CA**TTTGTTAATTTA 282

*msg*47 TTCAAATCAATTG**TATATTA**GAAAATAAGATAAATT**CA**TTTATTAATTTA 275

*msg*28 TTTAAATC**AATAATG**CAGTTAAAAAG----**CA**AGTCTTT---------**CT** 280

*msg*97 TCTAAATCAATAA**TATAATT**CAAAATT----AGTTT**CT**T---------**CT** 296

*msg*22 TCTAAATCAATAA**TATAATT**CAAAATT----AGTTT**CT**T---------**CT** 287

*msg*11 TTTAAATCAACAA**TATAATT**CAGAACT----TAATTC**CT**----------- 277

*msg*42 TTTAAAT**CA**CTAATTC-----GAAGGG----GGATTTAT----------- 300

* ****** * * * *

*msg*101 TTATTTATT-ATTTTTTTTT**ATG**CAATTATTTTTAGGAACGTGTATTTTT 336

*msg*54 TTATTTTT--TATTTTTTTT**ATG**CAATTATTTTTAGGAACGTGTATTTTT 330

*msg*47 TTATTTTTTATTTTTTTTTT**ATG**CAATTATTTTTAGGAACGTGTATTTTT 325

*msg*28 --ATTGTC--TTTCCCATTT**ATG**CTATTTTTTACAAAGATATATGTTTTC 327

*msg*97 --GTT-TC--TTTTTTGTTT**ATG**TCACTTTTTGTAAAAGCATACATTCTT 342

*msg*22 --GTT-TC--TTTTTTGTTT**ATG**TCACTTTTTGTAAAAGCATACATTCTT 333

*msg*11 --TTAT--T-TTCCTTCTTT**ATG**TTTCTGTTTATGGAAATATGCATTTTA 322

*msg*42 --ATTCCCC-CTTCCCTCAT**ATG**CAGTTATTTACCAAAACATGCATTTTT 347

* **** * *** * ** *

**<-------intron------------**

*msg*101 TTAGTCTTTATTAGTATAAAAAGA**GTTTTT**TCAGAAGATGACGGTTTTTT 386

*msg*54 TTAATCTTTATTAGTATAAAAAGA**GTTTTT**TCAGAAGATGAGGGTTTTTT 380

*msg*47 TTAATCTTTATTAGTATAAAAAGA**GTTTTT**TCTGAGGAGGAGGGTTTTTT 375

*msg*28 CTAATCTTTATTATCATACGAAAT**GTTTTT**TCAAAGAATGAAAACTTTTT 377

*msg*97 CTGAATTTAATTGATATAAAAACA**GTTTTT**TCAAAAAACGAAGATTTTTT 392

*msg*22 CTGAATTTAATTGATATAAAAACA**GTTTTT**TCAAAAAACGAAGATTTTTT 383

*msg*11 TTACTTGTTGTTGCTATAAAAAGG**GTTTTT**TCAGAGAACGAGGATTCTTT 372

*msg*42 TTGATTTTCATCAGCATAAAAAGA**GTTTTT**TCAGAGAATAACGAAATTTT 397

* * * *** ** ******** * * * ***

----------------------------------------->

*msg*101 AGGTAGCTTTTGTTTTTTT-TTTCTTAATTTTAATTTTT**TAG**ATCTTGTC 435

*msg*54 AAGTAGTTTTTGTTTTTTTTTTTCTTAATTTTAATTTTT**TAG**ATCTTATC 430

*msg*47 AAGTAACTTTTGTTTTTTT-TTTCTTAGTTTTAATTTTT**TAG**ACCTTATC 424

*msg*28 AAGTAATTATTATT------CTA-TTAATTTTAATTTTC**TAG**ATCTTATT 420

*msg*97 GGGTAATATTTATTTAACTTAATTCCAACTTTAATCTTT**CAG**ATCTTATT 442

*msg*22 GGGTAATATTTATTTA-CTTAATTCCAACTTTAATCTTT**CAG**ATCTTATT 432

*msg*11 AGGTAACTTTTTTTTT-TT-TTATTTAATTTTAATTTCT**TAG**ATCTTATT 420

*msg*42 AGGTATTCTTTTTTTT-TT-TTTTTGATTTTCCAATTAC**TAG**ATCTTGTT 445

*** ** ** * ** * * *** *** *

*msg*101 -AATGATTATTCTCCATTATATGAAGATGATCCATTACTTTCCTCTATAC 484

*msg*54 -AATGATTATTTTCCATTATATGAAGATGATCCATTACTTTCCTTTATAC 479

*msg*47 -AATGATTATTCTCCATTATATGAAGATGATCCATTACTTTCCTCTATAC 473

*msg*28 -AATCATTATTCCCCACTATATAATGAAGATCTATTGCTTTTTCATACCC 469

*msg*97 -AA---TTATTCTCCACTAAATGAAGACGATTCATTATTTCCCCTTACTC 488

*msg*22 -AA---TTATTCTCCACTAAATGAAGACGATTCATTATTTCCCCTTACTC 478

*msg*11 CAAACATT-TTCTCCTCTGCATGATGAAGATCCATTACTTTCATACGAAG 469

*msg*42 CAAACATT-TTCTCCTCTGCATGATGAGGATCCATTACTTTCATATGAAG 494

** ** ** ** * ** * ** *** *** **

*msg*101 TTAGGAGTGATGAATACCATGTTCAATTATATGAACAATTAAAATGGATA 534

*msg*54 TTAAGAATGATGAATACCATGTTCAATTATATGAACAATTAAAATGGATA 529

*msg*47 TTAAGAGTGATGGATACCATGTTCAATTATATAAACAATTAGAATGGATG 523

*msg*28 AAAGAAGTTATATGTATTATACTAATTTACATCATAGTTTGGAAAAACTA 519

*msg*97 ATAGAAGTGCACTTTATGATCTTCGTTTAGCATATCAATTGCAAAAACTA 538

*msg*22 ATAGGAGTGCACTTTATGATCTTCGTTTAGCATATCAATTGCAAAAACTA 528

*msg*11 TTACTACTGAAGAATATCATCATCAATTAAGCAAAAAGTTGCATAATTTA 519

*msg*42 TTACTAATGAAGAATATCATTATCAATTAAACAAAAAATTGTATAAATTG 544

* * * ** ** * *** * ** * *

*msg*101 GAACACGAGAATTTAAGGCAGAATTATATAGATGACTACTATTATTTAGC 584

*msg*54 GAACACGAAAATTTAAGGCAGAATTACATAGATGACTACTATTATTTAGC 579

*msg*47 GAACACGAGAATTTAAGGCAGAATTACATAGATGACTACTATTATTTAGC 573

*msg*28 GATCTTGAGAATCTATGGTACAATTACATAGATGATTATTATTATGTTAT 569

*msg*97 GAACTTGAAAATCTCTGGTACAATTATAAACATGATTATTATTATGTTGC 588

*msg*22 GAACTTGAAAATCTCTGGTACAATTATAAACATGATTATTATTATGTTGC 578

*msg*11 GAACTTAAAGAACTAAAAAATAGTAATGAATATT---TATATTACCTTGC 566

*msg*42 GAACTTGAAAAACTGAAAAATAGTAAAGAATATTTATATTATTATCTTAC 594

** * * * * * * * * * ** ***** *

*msg*101 TATTATATTAGATAACTACTATTATTTAGCTATTATATTAGATAGCT-AC 633

*msg*54 TACTATATTAGATA------------------------------ACT-AC 598

*msg*47 TATTATATTAGATA------------------------------ACT-AC 592

*msg*28 TACTATGTTAA----------------------------------CT-CC 584

*msg*97 TGCTATGTTAG----------------------------------CT-CC 603

*msg*22 TGCTATGTTAG----------------------------------CT-CC 593

*msg*11 TACTTTTTTAA----------------------------------CTTTC 582

*msg*42 AACTTTTTTAA----------------------------------CTTCC 610

* * *** ** *

*msg*101 A--ATGATTTAGTTACTATATTAGATAACAATGATTTATACCGTTATTGT 681

*msg*54 A--ATGATTTA-------T-----AT---------------TATTATTGT 619

*msg*47 A--ATGATTTA-------T-----AT---------------CGTTATTGT 613

*msg*28 AG-ACCAGTTA-------T-----A------------------TGATTGT 603

*msg*97 AGGTCGATTAT-------------C------------------TTATTGT 622

*msg*22 AGGTCGATTGT-------------A------------------TTATTGT 612

*msg*11 A--ACGATTTTA------------AT---------------AACAATTGC 603

*msg*42 A--GCGATTTTA------------AT---------------AACAATTGT 631

* * * ****

*msg*101 GAAGCTAAGTTAAAGGAAACTTGCATTAGTAT--AGAAGAC-GTAAATAA 728

*msg*54 GAAGTTAGATTAAAGGAAGCCTGCATTAATAT--AGAAGAC-GTAAATAA 666

*msg*47 GAAGTTAAATTAAAGGAAGCTTGCATTAGTAT--AGAAGAC-GTAAATAA 660

*msg*28 AAAAGCAATCTAGTTAAAGTCTGTACTGAACT--TAAGAAT-ATCAATGA 650

*msg*97 CAAAATGACATGACTAAAATATGTACTAATAT--TAAGAAT-ATGAATGA 669

*msg*22 CAAAATGACGTGAATGAAATATGTGCTAATAT--TAAGAAT-ATGAATGA 659

*msg*11 ATAGCAAAATTAAAAAAAGTCTGCAATAGTGATGTAAAAATGTTGAATAG 653

*msg*42 ATAGCAAAACTAAAAGAAGTCTGCGATAATGATGTAAAAATGCTGAATGA 681

* * ** ** * * * * ***

*msg*101 AAGATTCCAAAAGATTTGTAAAAACCCTGAAGCATCATGCAAGGGTGCAT 778

*msg*54 AAGATTCCAAGAGATTTGTAGAAACCCTAAAGCATCATGCAATGATGCAT 716

*msg*47 AAGATTCCAAGAGATTTGTAGAAAGCCTAAAGCATCATGCAAGGATGCAT 710

*msg*28 AAAGCTTCGAAAAATTTGTGAAAAACCTAAAATGGTATGTGGTTTAGCGT 700

*msg*97 AAGAGTTCAAGGAATTTGTACA---TCTAAAACAATATGTAATCATGCAT 716

*msg*22 AAGAATTCAAGGAATTTGTGCA---CTTAAAACAATATGTAATCGTGTAT 706

*msg*11 TCATATTCATTATATTTGTAATAATCTTCAATTACTATGTACTTCTTCAA 703

*msg*42 TGATATTCATCATATTTGCAATAACCCTCAATTAGTATGTACTT---TAA 728

* * ***** * ** ***

*msg*101 ACAGTAAAATTGTCAATAAAGCAAAAGAAATAGCTATTAGCTTTTCAGAT 828

*msg*54 ACTATAAAATTACCAGTAAAGCAAAAGAAATAGCTACTAGCTTTTCAAAT 766

*msg*47 ACTATAAAATTACCAAAAAAGCAAAAGAAATAGCTACTAGCTTTCCAAAT 760

*msg*28 ACAATGAAATGATGCTTAAAATAAAAAAAACTGTTGAGGAGCTTTCGGAT 750

*msg*97 ACAATGAAATAATGACAAAGATAAAAAAAACAGCTCAAGAACTTTCGGAC 766

*msg*22 ACAATGAAATAATGATGGGGATAAAAAAAACTGATGAAGAACTTTCAGAC 756

*msg*11 ATACTCAAATGTCTAATGCGATAAAAAAAA---TTAAAGATTTTTTGGAT 750

*msg*42 ATACTCAAATATCTAATGCGATAAAAAAAA---CTAAAGATGATTTGGAT 775

* * **** **** *** * * *

*msg*101 AAAGA--------AACAAATGA-ACGTGATCAATGTAATAAATTACAAGT 869

*msg*54 AAAGA--------AACAAATGA-ACATGATCAATGTAATAAGTTACAAGT 807

*msg*47 AAAGA--------AACAAATGA-ACGTGATCAATGTAATAAATTACAAGT 801

*msg*28 GAAAG--------AATAACTGA-GCACGATAAATGTAACAAATTACAAAT 791

*msg*97 GAAAA--------AATAAGTGA-ACATGATAAGTGTAACAAATTACAAAT 807

*msg*22 AAGAG--------AATAACTGA-GCATGATAAATGTAACAAATTACAAAT 797

*msg*11 G---A--------AACAAATTA-TCATAATCGGTGTAGCAAGCTGCAAAC 788

*msg*42 AAAGAAACGGATAAAGAAACGAATTATCATCACTGTAGCAGGCTGCAAAC 825

** ** * ** **** * * ***

*msg*101 TAAATGCTTTTTCCTGGAACTTCATGGTTCATGGCATATTGGTGCTAAAT 919

*msg*54 TAAATGCTTTTTCCTAGAACGTTATGGTTCAAAGCATATTGGTACTAAAT 857

*msg*47 TAAATGCTTTTTCCTAGAGCATCATGGTTCAGGGTATATTGGTACTAAAT 851

*msg*28 GAGATGTCATTTTCTAGAACACCATAAACCATGGTATCTTAATAGCGGAT 841

*msg*97 GAGATGTTTTCTTTTCGAACGCCATGGTATGCAGTATCTTAACGAAAAGT 857

*msg*22 GAGATGTTTTCTTTTCGAACGCCATGGTATGCAGTATCTTAATGAAAAGT 847

*msg*11 AGATTGTTTTTTCTTAGAACAATATAGTCCAGAAGATCTTTCTGCAAAAT 838

*msg*42 TGAATGTTTTTTCTTAGAACAATATGGTTCAGGAGATCTTTCTGCAAAAT 875

** * * * ** * ** ** ** *

*msg*101 GTGCTACATTAAAAGAGTATTGCTACAATAAAGTCCGTATGGGAGTGGCT 969

*msg*54 GTGCCACATTAAAAGAGTATTGCTACAATAAAGTCCGTATGGGAGTAACT 907

*msg*47 GTGCTACATTAAAAGAGTATTGCTACAATAAAGTCCGTATGGGAGTGGCT 901

*msg*28 GTAAAAAATTAAAAGAGCATTGTTATAAAAAGGTCCGTATGGAAGTAGCA 891

*msg*97 GTAAAGAACTTAAAGAGAGCTGTTATAATAAAGTCCGTATGGAAGTAGCA 907

*msg*22 GTAAAAAACTTAAAGAGAGCTGTTATAATAAAGTCCGTATGGAAGTAGCA 897

*msg*11 GCAATAAACTAAAAGAAAATTGTTATAATAAGGCCCGTATGGAAGTAGCG 888

*msg*42 GCAATAAACTAAAAGAAAATTGTTATAATAAGGCCCGTATGGAAGTAGCG 925

* * * ***** ** ** ** ** * ******** *** *

*msg*101 GAAATAATTGTTTATGATTTTTTGAGAGGAACTTCAAATAATTTAGATAC 1019

*msg*54 GAAGTAATTGTTTATGATTTTTTGAGAGGAACTTCAAATAATTTAGATAC 957

*msg*47 GAAGTAATTGTTTATGATTTTCTGAGAGGGACTTCAAATGATTTAGATGC 951

*msg*28 CAAGTAATTCTTTATGATCTTTTAAGAGGAACTTCTAACGATTTTAATAA 941

*msg*97 AGAACACTTCTCTATGATATTTTAAGAGGAACTTCAAGTGATTCAAAATC 957

*msg*22 AGAACACTTCTCTATGATATTTTAAGAGGAACTTCAAGTGATTCAAAATC 947

*msg*11 GAAGGAATTATGCATAGATTTTTAAAAGGGACAC---ATAGT---AGCAC 932

*msg*42 GAAGAAATTATGCATAGATTTTTAAAAGGGACAC---ATGAT---AAGAC 969

* * ** * ** ** * * *** ** *

*msg*101 TTGCATAAGGAAACTTCAAAATGAATGTCAATTAGTTAATCAGCGAAGTC 1069

*msg*54 TTGCATAAGGAAACTTCAAAATGAATGTCAATTAGTTAATCAGCGAAGTC 1007

*msg*47 TTGCACAAGGAAACTTCAAAATGAATGTCAATTAATTAATCAGCGAAGTC 1001

*msg*28 ATGTTTAAGAAAACTCAAAAGCGAATGTCTATCACTCAATCAACGAAGTC 991

*msg*97 TTGCATAGGAAGATTAAAAAATGTATGCCAGTTACTTAGTCAACAAAGCC 1007

*msg*22 TTGCATAAGAAGACTAAAAAATGTATGCCAGTTACTTAGTCAACAAAGCC 997

*msg*11 TTGTTTACAAAAATTCAAAGAAAAGTGTCTATTATTTAGTCATCAAAGCC 982

*msg*42 TTGTTTAGGAAAACTCAAAGAAAAATGTCTATTATTTAGTCATCAAAGCC 1019

** * * * * ** ** * * * * * *** * *** *

*msg*101 CCGAATTATTTGATTTGTGTGTCAATGTTGACACAGTTTGTAGTAAATTT 1119

*msg*54 CCGAATTATTTGATTTGTGTGTCAATGTTGACACAGTTTGTAGTAAATTT 1057

*msg*47 CCGAATTATTCGATTTGTGTGTCAATGTTGACACTGTTTGTAGCAAATTT 1051

*msg*28 CAGAATTGCTCGATTTGTGTACTAATGGTGCTAATGTTTGTCTTGAATTT 1041

*msg*97 CTGAATTGCTTACTTTGTGTATGGATTATACCAGAGTTTGTAATGAATTT 1057

*msg*22 CTGAATTGCTTACTTTATGTATGGATTATACCAGAGTTTGTAATGAATTT 1047

*msg*11 CAGAGTTATTTAGTTTGTGTATTAGTGGTTCCAATGTTTGTGAAAAATTT 1032

*msg*42 CAGAGTTATTTAGTTTATGTATTAGTGGTTCCAGTGTTTGTGAAAAATTT 1069

* ** ** * *** *** * * * ****** *****

*msg*101 ATTTCGAAATCTAAAGAGGAATGTCAAAATTCTCAGGCTTTATTTTCATC 1169

*msg*54 ATTTCGAAATCTAAAGAGGAATGTCAAAATTCTCAGGCTTTATTTTCATT 1107

*msg*47 ATTTTGAAATCTGAAAAAGAGTGTCAAAGTTCTCAGGCTTTATTTTCATC 1101

*msg*28 ATTTCAAATTCTAAGAAAGAATGTCAGAATTTTCAAAATCTGTTTTTATC 1091

*msg*97 ACTTCATACTCTCAAGAAGAATGTAAAAATTTTGGAACTCAATTTTTATC 1107

*msg*22 ACTTCATATTCTCAAGAAGAATGTAAACATTTTGGAACTCAATTTTTATC 1097

*msg*11 ATTTCAGAAACTAAAACAAACTGTCAAAATTTTCAAATTCAGTTTTTATC 1082

*msg*42 ATTTCAGAAACTGAAACAAACTGTCAAAATTTTCAAACTCAGTTTTTATC 1119

* ** * ** * * *** * ** * * **** **

*msg*101 GAATCA------AATCTCAGAAAAAGAATGTAAGACTTTGCTAGCAAAAT 1213

*msg*54 GAATCA------AATCTCAGAAGAAAGATGTAAGACTTTACTAGCAAAAT 1151

*msg*47 GAATCA------AATCTCAGAAGAAAAATGTAAGACTTTGTTAGCAAAAT 1145

*msg*28 AGAACAAAAAACAATATCTGAAAAAAATTGTCTAAATTGGCTAAAAATAT 1141

*msg*97 AAAACAGGGAATAATGACAGAAGAAAATTGTGTAAAATGGTTAGAAATAT 1157

*msg*22 AAAACAGGGAATAATGACAAAAGAAAATTGTGTAAAATGGTTAGAAATAT 1147

*msg*11 AAAACATAAAGAAATAACACGAGAAAACTGCATAGTTTGGCTAAAAAAAT 1132

*msg*42 AAAACATGAAGAAATAACACAAGAAAACTGCATAATTTGGTTAAAAAAAT 1169

* ** *** * * ** ** * ** ** **

*msg*101 GCTATTCTATTCTTTCAGACTGCCCTGTTCTTCGTCCAAAATGTCGTTCA 1263

*msg*54 GCTATTCTATTCTTTCAGAGTGTCCAATTCTTCGTTTGCAATGTCGTTCA 1201

*msg*47 GCTATTCTATTCTTTCAGACTGCCCTGTTCTTCGTCCAAAATGTCGTTCA 1195

*msg*28 GCTATTACATTATTCCAGATTGTAGAGTTCTCTACCATAGCTGCCATTCA 1191

*msg*97 GTTATTATGTTATCCCTGATTGCCAAAATCTCCACAGTCTATGTCGTTCA 1207

*msg*22 GTTATTATGTTATCCCAGATTGCCAAAATCTCCACAGTCTATGTCGTTCA 1197

*msg*11 GCTATTATAACGTTTTGAATTGTCGAAATATTTATCATATGTGCCGTTTG 1182

*msg*42 GCTATTATAGCGTTTTGAATTGTCGAAATATTTATCATATGTGCCGTTTG 1219

* **** * * ** * * ** * **

*msg*101 TTAAAAATGGAATGTGCAGAAAAAGGCTTTTTTTATGATATTTCGGAAAA 1313

*msg*54 TTAAAAATGGAATGTGCAGAAAAAGGCTTTTTTTATGATATTTCGGAAAA 1251

*msg*47 TTAAAAATGGAATGTGCAGAAAAAGGCTTTTTTTATGATGTTTCGGAAAA 1245

*msg*28 TTTCGAATGAGTTGCGCAGAAAGTGGTTTTTTTTTTAAT---TCAAAAGA 1238

*msg*97 TTTCGAATGGAATGTTCTGAAAAAGGCTTTTTCTATAAT---CCAAGAGG 1254

*msg*22 TTTCGAATGGAATGTTCTGAAAAAGGCTTTTTCTATAAT---CCAAGAGG 1244

*msg*11 TTTCAAACAAATTGTGCAGAAAATGGTTTTTTTTCCAATTTTGACAGTAA 1232

*msg*42 TTTCAAACAAATTGTGCAGAAAATGGTTTTTTTTTCAATTTTGACAGTAG 1269

** ** ** * **** ** ***** * **

*msg*101 TCATAATTTTAATCTTCTTGAAAACCCGTTTATTCATATGAACAGAAATG 1363

*msg*54 TCATAATTTTAATCTTCTTGAAAACCCGTCTACTCATATAAACAAAAATG 1301

*msg*47 TCATAATTTTGCTCTTCTTGAAAACCCATTTACTCATATGAACAGAAATG 1295

*msg*28 TTACAATTTTGATCTTTTTGAAAATCCATCTACTTATATGGTAGAAAATG 1288

*msg*97 CCATAATTTTGATCTTTTTAAAAATCCATCTACCGATCTAGATGAAACCG 1304

*msg*22 CCATAATTTTGATCTTTTTAAAAATCCATCTACCGATTTAGATGAAACCG 1294

*msg*11 TTATAATCTTAATCTTCTTGAAAGCCCGTTTATCGATATTGATGAAGATA 1282

*msg*42 TTATAATCTTAATCTTCTTGAAAGCCCGTTTATCAATGTTGATGATGACA 1319

* *** ** **** ** *** ** * ** ** *

*msg*101 GTGTTGAATATGCATATGGAAAACTATATAATTCAGGCATATATGTTGCT 1413

*msg*54 GTGTTGAATATGCGTATGGAAAACTATATAATTCAGGTATATATGTTGCT 1351

*msg*47 GTGCTGAATATGCATATGGAAAACTATATAATTCAGGCATATATGTTGCT 1345

*msg*28 GGGTCCAATATGCCTATGAAAAATTGCATAATTTAGGTATTTATGTCTCT 1338

*msg*97 ATATTCAACATACCTATGAAAAATTGCATGATTTAGGTATCCATGTTACT 1354

*msg*22 GTATTCAACATACCTATGAAAAGTTGCGTGATTTAGGTATCCATGTTACT 1344

*msg*11 CAATTCAATACAGCCATAAAAAGTTAAGTCAATTAGGTATTTATGTTGAT 1332

*msg*42 CAATTCAATACAGCCATGATAAGTTAAATCAATTAGGTATTTATGTTGAT 1369

** * ** ** * * * * *** ** **** *

*msg*101 AAAATACAGAATCTTTCAGATTTATTAATTGCCGATTTTTTGATAAATA- 1462

*msg*54 AAAATACAGAATCTTTCAGATTTACTAATTGCCAATTTTTTGATAAATA- 1400

*msg*47 AAAATACAGAATCTTTCAGATTTATTAATTGCCAATTTTTTGATAAATA- 1394

*msg*28 GAAATACAGGATCTTCCAGAAATATTCGTTCTTGAGCTTTTAGTTCAAA- 1387

*msg*97 GGATTAGAGAAATATTCAGAATTGCAAGTGGCTAACTTTTTGGTTCAAGA 1404

*msg*22 GGATTAAAGAAATATTCGGAACTACAAGTGGCTAACTTTTTGGTTCAAGA 1394

*msg*11 AAAATGCCATCACTTCCGAATGAAGTAATTGCTAGCATTTTAATTCAAG- 1381

*msg*42 AAAATGCCATCACTTCCGAAGGAAGTAGTTCCTAGCATTTTAATTCGAA- 1418

* * * * * * **** *

*msg*101 ATA-------C-------AAAAGCAGTAGACAGAGATTGTAAAAAAGTGT 1498

*msg*54 ATA-------C-------AAGTGCAGGAGACACATATTGTAAAAAAGTGT 1436

*msg*47 ATA-------C-------AAGAGCAGGAGACAAAAATTGTAAAGAAGTAT 1430

*msg*28 AAA-------T-------TACTACGGGACGTAATGACTGTGTAAAAGCCT 1423

*msg*97 ATATTTGGTTCAAGAATATTATCCAGGATATCTGCAATGTAAAAAAATAT 1454

*msg*22 ACG---------------TTATCCAGGATACTTGGAATGTAAAAGTGCGT 1429

*msg*11 ATG-------T-------TTCTGCACAAATCTCTAAATGTAAAAAAGAAT 1417

*msg*42 ATG-------T-------TAATGCAAGAACCACTGAGTGCAAAGAAGAAT 1454

* * * * ** * *

*msg*101 TGGATGAAAAATGTTCTTCGATAAATTATTTAAACTACATCGAGCCTATG 1548

*msg*54 TAAATAAAAAATGTTCTTCGATAAATTATTTAAGCTACATCAAGCCTATA 1486

*msg*47 TAAATAAAAAATGTTCTTCGATAAATTATTTAAGCTACATCAAGCCTATA 1480

*msg*28 TAAATAAAAGATGTCCTTCGGCAGAATATTTAGAATTTGCTAAATCAATA 1473

*msg*97 TTGATCAGAAATGTTCATCGATACAATATTTAGAACCCATTAAACAGATG 1504

*msg*22 TAAATAAAAAATGTTCCTTAATACAATATTTAGAATCAATTAAAGACATG 1479

*msg*11 TGAATGAAAAATGTCCTTTAGTGGAATATTTAGATGTTTTTAAAGATATA 1467

*msg*42 TAAATAAAAAATGTCCTTTAGCGGAATATTTAGATGTTTTTAAAAATATA 1504

* ** * * **** * * * ****** * **

*msg*101 TGCAATGC---ATCTTC-GAATAGTGAATATAAAATTTGTAAAAGCATAT 1594

*msg*54 TGCAATGT---ATCTTC-AAATAATGAATATAAAATTTGTAGAAAGATAT 1532

*msg*47 TGCAATGT---ATCTTC-AAATGGTGAATATGAAATTTGTAAAAGTATAT 1526

*msg*28 TGTAATATCA-AATTTCAGAGTGGTATTCACTATGCTTGTATTAACTTAA 1522

*msg*97 TGCACTTCTAAGGATTC-ACGTAACCTCTATAACGTTTGTCATGATATGT 1553

*msg*22 TGCATTTCTAAAGATTG-GAAAGGCAACTATAATGTTTGTTATAATATGT 1528

*msg*11 TGCAGTAA---AACTA----ACAATAAAGATAATATTTGTAAAGGCATAT 1510

*msg*42 TGCACTAA---AACTA----GCAACGGACATAATATTTGCAAAGACATAT 1547

** * * * * * *** *

*msg*101 ATTACAAAACTAGAGATTATTGTTATTCACTTTTAGAAAAGTTTAAAGAA 1644

*msg*54 ATTACAAAACTGGACATTATTGTTATTTACTTTTAGGAAAGTTCGAAAAA 1582

*msg*47 ATTACGAAACCAAAAAGTATTGTAATTTACTTTTAAAAAAGTTTGAAGAA 1576

*msg*28 ACCATAAAGCCGAAGTTTACTGTGAACCCTTTTTGGATAAACTCAAAAAG 1572

*msg*97 ATGCTGGAACCAGAAACTATTGCTACTCCCTTCAAAGCAAACTTAAACAA 1603

*msg*22 ATGCTGGAACTAGAGATTATTGTTATTCCCTTATAAAAAAATTAAAAGAA 1578

*msg*11 ATGATAAAACTAAGCAGCATCTTGGACCTTATTCAAAGGAATTCAG---T 1557

*msg*42 ATGATAAAACTAAGCTGCATTTTGAATCTTATTCAAAGAACTTCAC---T 1594

* * * * * * *

*msg*101 AGCAATCAATTTTTGAAGTATTCAGATCGTCCTCTTTCGAAAACCGAATG 1694

*msg*54 AGTAATCAATTTTGGTGGTATCCAGATCGTCCTCTTTCAAAAATCCAATG 1632

*msg*47 AGCAATCAATTTTTGAAGTATTCAGATCGTCCTCTTTCGAAAACCGAATG 1626

*msg*28 GGTATTCAATTTTGGTGGTATCCACATCTTCCTCTTTCTGAGACAGAATG 1622

*msg*97 GGTATCTGGTTTTGGTGGTATCCAGATCGCCCTCTTTCAAAAGAAGAATG 1653

*msg*22 GGTATTAACTTTTGGTGGTATCAAGACCTCCCTCTTTCGGAAAAAAAATG 1628

*msg*11 GGTAATAA-TT-T-GTCGATTTCACAAAAATCTTTTTCAAAAGAAAAGTG 1604

*msg*42 AGTAATAA-TT-T-GTCGATTTCACAAAAATCTTTTTCAAAAAAAGAGTG 1641

* * ** * * * * * * ** **** * * **

*msg*101 TGAAGAATATCTTTCAATATGTTATTTTATTGATAAATATTTCAATTACT 1744

*msg*54 TGAAGAATATCTTTCAATATGTTATTTTATTGGTAAATATTTTCATTACT 1682

*msg*47 TGAAGAATATCTTTCAATATGTTATTTTATTGATAAATATTTCAATTACT 1676

*msg*28 TATAGAATATTTGTCAGCATGTTATTTCGTTGATAATTATTTTATTTATT 1672

*msg*97 CAAAGAATATTTATTAGTATGTTATTTTATGCATAAAGCTATCACTTATT 1703

*msg*22 CGAAGAGTATCTGACAATGTGTTATTTCATGTATAAAACTATTACTTATT 1678

*msg*11 TATAGAATATTTACCATTATGTTATTTTTTTAACGAATATTTTGATGTCT 1654

*msg*42 TATGGAATATTTATCATTATGTTATTTTTTTAATGAATATTTTAATATCT 1691

** *** * * ******** * * * * * *

*msg*101 GGAATTCTGGTTTTTGGAAGAAATACGATACTTGTAAATATGTACGAATT 1794

*msg*54 GGAG---------------GGGTTACGATACTTGTAAATATGTACGAGTT 1717

*msg*47 GGAATTCTGGTTTCTGGAAGAAATACGATACTTGTAAATATGTACGAATT 1726

*msg*28 GGAA---------------GACTTATGATATTTGCAAAGATATACGGCTT 1707

*msg*97 GGCT---------------GAGTTATAACCTTTGCAAAGATATACGACTC 1738

*msg*22 GGAC---------------AGGTTATGATATTTGTAAAAATATACGACTC 1713

*msg*11 GGGT---------------CCAAAGTGATTTATGTAAAACAATACGACTT 1689

*msg*42 GGGT---------------CCAAAGTGGTTTATGTAAAACAATACGACTT 1726

** ** *** **** *

*msg*101 GTTTGTTATCAAGAAGATTTAGATGCAGCTACAAATATGATTTTAATAAA 1844

*msg*54 ATTTGTTATCAAGAAGATTTAGATGTAGCTACAAATCTGGTTTTAATAAA 1767

*msg*47 GTTTGTTATCAAGAAGATTTAGATGCAGCTACAAATATGATTTTAATAAA 1776

*msg*28 GTTTGTTATCAAGCAGGTTTAGAAATAGCAGCCAATAAGGCTCTAATAAA 1757

*msg*97 GTTTGTTACCAAGCAGGTTTAGAAAGAGCAGCTAATATGGCTCTAATGAA 1788

*msg*22 GTTTGTTACCAAGCAGATTTGGAAAAAGCAGCTAATATGGCTCTAATGAA 1763

*msg*11 GCCTGTTATCAAGCAGATTTGGAAATGGAAGCTGATATGATTTTAATAAG 1739

*msg*42 GCCTGTTATCAAGCAGATTTAGAAACAGAAGCTGATATGATTTTAATAAA 1776

***** **** ** *** ** * * ** * * **** *

*msg*101 AAAGTTGAGTGGAAGGTTTAAATTAAAAAGAAATTTTCAAGGAATCAT-G 1893

*msg*54 AAAGTTGAGTGGAAGGTTTAAATTAAAAAGAAATGATCAAGGAATCAT-A 1816

*msg*47 AAAGTTGAGTGGAAGGTTTAAATTAAAAAGAGGTGATCAAGAAATCAT-A 1825

*msg*28 AAGGCTAAGTGGGAAATTAAGATTGGGAAAAAGTTACTCTG-AGTCTTTA 1806

*msg*97 AAAGTTGAATAGAAAATTAACATTAGAAAA---TAATCCTG-AATC---- 1830

*msg*22 AAAGTTAAATAGAAAATTAACATTAGGAAA---TAGTCCTG-AGTC---- 1805

*msg*11 GAAATTAAATGAAAAAATAAGTTCAAGAGA---TCGTT-GGTCTTCA--A 1783

*msg*42 GAAATTAAATGAAAAAATAAGTTCAAGAAA---TCGTT-GGTCTTCA--A 1820

* * * * * * * * * * * **

*msg*101 GCTTTACATCGAACTATGAGCGATTGTAAAAATGCTCTTTTAGAAGAATG 1943

*msg*54 GCTTTACATCGAACTATGAGCGATTGTAAAAATGCTCTTTTAGAAGAATG 1866

*msg*47 GCTTTACATCGAACTATGAGCGATTGTAAAAATGCTCTTTTAGAAGAATG 1875

*msg*28 ACT---GATAGAGTTCTGAATGATTGCAAAAAAGTTCTTTTAAAAGAATG 1853

*msg*97 --TTCA-AACAATCA--AAAAAACTGCGAAAAAGTTCTTATACAAGAATG 1875

*msg*22 --TTCA-AGCAATCA--AAAAAACTGCGAAAAAGTTCTTGTACAAGAATG 1850

*msg*11 GTTTTA-ACCTAACTA---AGAATTGCACAAAGCGTCTTTCTCTAGTATG 1829

*msg*42 GTTTTA-ACCTAACTA---AGAATTGCACAAAGCGTCTTTCTCTAGTATG 1866

* * * * ** *** **** ** ***

*msg*101 TGGCAATTTTATGTATCATAGTTATCATATACTATATAAATGTTTACATC 1993

*msg*54 TGGCAATTTTATGTATCATAGTTATCCTATACTATATAAATGTTTACGTC 1916

*msg*47 TGGCAATTTTATGTATCATAGTTATCATATACTATATAAATGTTTACGTC 1925

*msg*28 TGGACAATTTATGTACTACAGCTTTCATGTATTATATAAGTGTCTACATC 1903

*msg*97 CAAGCATTTTATGTATCATAGTTACTATATACTATATAGTTGTCTGCACC 1925

*msg*22 CGAACATTTTATGTATTATAGTTACTATATACTATATAGTTGTCTGCACC 1900

*msg*11 TAAAGAAGTTATGTATAGCAATTATCATGTATTATATAGATGTCTGCATC 1879

*msg*42 TAAAGATGCTATGTATTATAATTATTATGTACTATATAGATGTCTGCATC 1916

* ****** * * * ** ****** *** * * *

*msg*101 CAGAAGAAACATGCAAAAATTTAACAAATCTTCTTGATAAAAACTGCGAA 2043

*msg*54 CAAAAGAAACATGCAAAAATTTAACAAATCTTCTTGATAAAAACTGCGAA 1966

*msg*47 CAAAAGAAACATGCAAAAATTTAACAAATCTTCTTGATAAAAACAGCGAA 1975

*msg*28 CACAGGAAACATGTAAAAATTTGACAAATATTCTTTACAAGGATTTTAAA 1953

*msg*97 CAAAAGAAGCATGTAAAAATTTAACAACTTTTGTTAACAGTAACTCCAAA 1975

*msg*22 CAAAAGAAACATGTAAAAATTTAACAGCTTTTGTTAACAGCAACTCCAAA 1950

*msg*11 TACAAGAAACGTGTACAAAATTAGCATCCTTCGTTCAGGAGAAGTGCGAT 1929

*msg*42 TACAAGAAACGTGTACAAAGTTAGCATCCTTTGTTGATGAGAAATGTAGT 1966

* * *** * ** * *** ** ** * ** * *

*msg*101 AGATTAGAGAAAAATTTAAAAGCGGCAATCCCAAATCCTGAATATACTAC 2093

*msg*54 AGATTAGAGAAAAATTTAAAAGCGGCAATCCCAAATCCTGAATATACTAC 2016

*msg*47 AGATTAGAGAAAAATTTAAAAGCGGCAATCTCAAATCCTGAATATACTAC 2025

*msg*28 AGATTTGATGAAACTTTAAAGTATGTAATCATAGAACCTACTTATGATAC 2003

*msg*97 GATTTAGAAAAAAACTTAAAAGATACTCTCATGGATCCTGACTATGACAA 2025

*msg*22 GATTTAGAAAAAAACTTAAAAGATACTCTCATGGATCCTGACTATGACAA 2000

*msg*11 AAATTGCGTAATACTCTAAAAAATATAAACAATAATTTTACACTCACTTC 1979

*msg*42 AGATTAGGAGAGACTCTAAAAAAGATGAACAATAATTTTACACTCTCTTC 2016

** * * **** * * *

*msg*101 ATGTAGACAACTAAAAGAAGAATGTGATGAATTAGGGCCATATTCTGAAT 2143

*msg*54 ATGTAGACAACTAAAAGAAGAATGTGATGAATTAGGGCCATATTCTGAAT 2066

*msg*47 ATGTAGAGACCTAAAAGAAGAATGTAATGAATTAGGGCCATATTCTGAAT 2075

*msg*28 ATGTAAAGAACAGAGAGAAGAATGTCGTAGATTAGGGCCATATTTTGAGT 2053

*msg*97 GTGTAAAAAGTATAAAAAAGAATGTCATAAATTGGAGTTTTATTTTCAAT 2075

*msg*22 GTGTAAAAAGTATAAAAAAGAATGTCATAAATTGGGATTTTATTTTCAAT 2050

*msg*11 TTGTGGAAAGCAAAAAGAAGAATGCAATAGTTTGAAATTATGCTCAGGAA 2029

*msg*42 ATGTGGAAAGCAAAAAAAAGAATGCAATAATTTGAAATTATGTTCAGGAA 2066

*** * * * * ******* * ** * *

*msg*101 ATACGCTGATATTATGCAAAATTTTTAAAAAAATGTGTGAAAATGTAGAT 2193

*msg*54 ATACGCTGATATTATGCAAAATTTTTAAAAAAATGTGTGAAAATGTAGAT 2116

*msg*47 ATACACTGATGTTATGCAAAATTTTTAAAAAGATGTGTGAAAATGTGGAT 2125

*msg*28 ATACTTTGAATTTATGCAAAGACCTCGATCAATCTTGTAAAAACATAGAT 2103

*msg*97 CTACTAAAAAGTTATGCGGAGAACTTAATACTGCATGTAAAAATATAGAT 2125

*msg*22 CTACTAAAAAGTTATGTGGAGAACTTAATACTACTTGTAAAAATATAGAT 2100

*msg*11 AGACTATAAGTTTATGTCAAAACCTCACAGCATATTGCAGAAATAAAGAT 2079

*msg*42 ATGCTATAAGTTTATGTCAAAAACTCGAAAGATATTGCAGAAATATGGAT 2116

* * ***** * * ** *** ***

*msg*101 AAGTTAATGGAATTTAAATTAAGTACACTTAAGGAAAAAAATTCAGTTTT 2243

*msg*54 AAGTTAATGGAATTTAAATTAAGTACACTTAAGGAAAAAAATTCAGTTTT 2166

*msg*47 AAGTTAATGGAATTCAAATTAAGTGCACTTAAGGAAAAAAATTCAGTTTT 2175

*msg*28 GAATTAATAACTTTGAAATCAAAAATACTTAAAGAAAATGACTCAAATTT 2153

*msg*97 GAAGCAATACTATTAGGAGTAAATATACTTAAA---AAAGGCTCAGATTT 2172

*msg*22 GAGGAAACACTATTAGGAGTAAATATACTTAAA---AAAGGCTCAAATTT 2147

*msg*11 GAACTTGAAAATCTGAAACTAAAAATACTAAAAAATGATACTGTTTTTTT 2129

*msg*42 GAACTTAACAATCTGAAACTAAAAATACAAAAAAATAATACTGTTTTTTT 2166

* * * ** ** ** * ***

*msg*101 AAAAAATAACACTACTTGTTTGAATTATCTCAACAATTATTGTTCGGAAA 2293

*msg*54 AAAAAATAACACTACTTGTTTGAATTATCTCAACAATTATTGTTCGGAAA 2216

*msg*47 AAAAAATAACATTACTTGTTTGAATTATCTCAACGATTATTGTTCGAAAA 2225

*msg*28 ACTTAATGAAACTACTTGTCTTAGCTATCTCAATAACTACTGCATACAAA 2203

*msg*97 ATACAATGAATCTTCTTGTCTCAAGCATCTTGCTGAACATTGTAGACAAA 2222

*msg*22 ATACAATGAATCTTCTTGTCTCAAACATCTTACTGAACATTGCACACAAA 2197

*msg*11 ACAAAGTCAAACTGTTTGTTTTGGTTATCTTACAGACTATTGTTTAAAAA 2179

*msg*42 ACAAAGTCAACCTGTTTGTTTCAATTATCTCACAGAATATTGTTTAAAAA 2216

* * * * * **** * **** * * ** ***

*msg*101 ATGTCTCAAG-TC--ACATA-----TGTACTAATAAAAATGAATCATGTA 2335

*msg*54 ATGTCTCAAG-TC--ACATA-----TGTACTAATAAAAATGAATCATGTA 2258

*msg*47 ATTTCTCAAG-TC--ACATA-----TGTACTAATAAAAATAAATCATGTA 2267

*msg*28 ATAATATAGG-CT--ATGCA-----TGCGTTAATAAAACAATATCATGTA 2245

*msg*97 ATAGTTCATATTC--ACATACAGTCTGTCGTGATAAAATAAAAACATGCA 2270

*msg*22 ATAGTTCGTATTC--ACATACAGCCTGTCGTAATAAAACGAAAACATGCA 2245

*msg*11 ACAGCTCACA-TTATACTTATG-GGTGTACTAATGCATCAAAGATATGTA 2227

*msg*42 ACAGCTCACA-TTATACTTATG-GGTGTACTAATGCATCAAAGATATGTA 2264

* * * ** * ** * *** *

*msg*101 CACAAATGTTAGATCAAATTCCAATGCACTGCGAACAATTATCTTTTTAT 2385

*msg*54 CACAAATGTTAGATCAAATTCCAATACACTGCGAACAATTATCTTTTTAT 2308

*msg*47 CACAAATGTTAAATCAAATTCCAATGCACTGCGAACAATTATCTTTTTAT 2317

*msg*28 AACATATGTTAGATTATGTTTCAAAGCAATGTCAGCAATTATCTCGTCAT 2295

*msg*97 ATCACATACTCAAGCGTGTTTCAGCATATTGTACACAGCTCTCACACTAT 2320

*msg*22 ATCACATACTCAAGCGTGTTTCAGCATATTGTACACAGCTCTCACGCTAT 2295

*msg*11 AAGAAATACTTAGAAGTGTTGAGGATGATTGCAAAAAATTATTACAGTAT 2277

*msg*42 GAGAAATACTTAGCAGTGTTAAGGAAGATTGCAAAAAATTATTACAGTAT 2314

* ** * ** * ** * * * **

*msg*101 TTATCATATTATAAATCTTCTAATGGTCAAATAAATCATATAAATTATGG 2435

*msg*54 TTATCATATTATAAATCTTCTAATGGTCAAATAGATCATATAAATTATGG 2358

*msg*47 TTATCATATTATAAATCTTCTAATGGTCAAATAGATCATATAAATTATGG 2367

*msg*28 TTATTATATTATGGGTTTTCTAATAAACAAGTGACTCATATGGAACATAG 2345

*msg*97 CTATCACATTATGAAGTTTCCAATAACCATATAACTCATATCAGTTATAG 2370

*msg*22 CTATCACATTATGAAGTTTCCAATAACCATATAACTCATATCAGTCATAG 2345

*msg*11 GTGAATGCTTATAAAACACTTCTTAATGGAGTATCTTATATTACATATAA 2327

*msg*42 GTGAATGCTTATAAAACACTTCTTAGTGGAGCATCTTATATTACATATAA 2364

* **** * * **** **

*msg*101 TCGTTGTTCTCAATTCCTCAATTATTGTGAAATGCTTAAAGAAACATGTC 2485

*msg*54 TCGTTGTTCTCAATTCCTCAGTTACTGTAAAATGCTCGGAGAAATATGTC 2408

*msg*47 TCGTTGTTCTCAATTCCTCAGTTACTGTAAAATGCTCGAAGAAACCTGTC 2417

*msg*28 TCGTTGTTCTTATTTCATTCATTACTGCAAAATGCTTAAAAGGAATTGTC 2395

*msg*97 TCGTTGTGCTAGTTTCAAATATCACTGTAAATTGCTTAAAAGCAGTTGTC 2420

*msg*22 TCGTTGTGTTAGTTTCAAATATCACTGTAAATTGCTTAAAGGCAGTTGTC 2395

*msg*11 TCGTTGTTTTCATTTCAAATATTATTGTGTTTTATATATAGGGGGTTGTC 2377

*msg*42 TCGTTGTTCTCATTTCAAATATTATTGTGTTTTATATATAGGGGGTTGTC 2414

******* * *** * * ** * * ****

*msg*101 CTA---AT---CTAACTAGTATATGTTCTCAAGTCGAACAATCATGTAAC 2529

*msg*54 CTA---AT---TTAACTAATATATGTTCTCAAGTCAAACAATCATGTAAT 2452

*msg*47 CTA---AT---TTAACTAGTATATGTTCTCAAGTCAAACAATCATGTAAC 2461

*msg*28 CTA---ATTCTTTAGACGATATGTGTTCTGAAGTTGAAAAAATATGTGAC 2442

*msg*97 CCGGATCT---TTAAACAACATATGTGCTGAAGTTGAGAAAAAGTGTAAT 2467

*msg*22 CTGGATCT---TTAAACAACATATGTGCTGAAGTTGAGAAAAAGTGTAAT 2442

*msg*11 CTT---AT---TTGCGTGATCTCTGTAGTAATATTACTATAATATGTAGT 2421

*msg*42 CTT---CT---TTGCATGATCTCTGTAGTAATATTACTATAATCTGTAGT 2458

* * * * *** * * * * ***

*msg*101 AAAGTTTCAACTCGAAAGAAAAACGTTATCGCTTTGATCAAAGTCGTTGG 2579

*msg*54 AAAGTTTCAACTCGAAAGAAAAACGTTATCGCTTTGATCAAAGTCGTTGG 2502

*msg*47 AAAATTTCAACTGAAAAGAAAAACATTATCTCTTTGATCAAAGTCGTTGG 2511

*msg*28 AATATTTCTACTAGAAACAAAAAATTTAATGATCTAATCAAAATACTTAA 2492

*msg*97 AATACTTCAACTCAAACTAACGAACTTAATAATTTAATTAAGGCTTTTGG 2517

*msg*22 AATACTTCAGCTCAAACTAACGAACTTAATAATTTAATTAAGGCTTTTGG 2492

*msg*11 AGAATATCAAAGGCAAGTGAGAATTTTAATGCATTAGCTAAGGTACTTGG 2471

*msg*42 AGTATATCAAAGACAAGTGAAAGTTTTAATGCATTAGCTAAGGTACTTGG 2508

* ** ** * *** * ** **

*msg*101 CGAAGAAGTATCTAATCATAGTAAATGTAAAAAAAAATTACATGAAGTAT 2629

*msg*54 CGA---AGTATCTAATCATAGTAAATGTGAAAAAAAATTACATGAAGTAT 2549

*msg*47 CGA---AGTATCTAATCATAGTAAATGTGAAAAAAAGTTACGTGAAGTAT 2558

*msg*28 TGAAGAAATATCTACACATGATAGATGTAAAAAAAAAATACTTAAAGAAT 2542

*msg*97 CGAAGGAATATCCACTCATGAAAAATGTAAGAAAAAATTACATAAAATAT 2567

*msg*22 CGAAGGAATATCTAGTTATGATAAATGTTATAAAGAATTAACTCAAGAAT 2542

*msg*11 GGG---AATATCTAATGTTTCTGAATGTGAAGAAAAAATG---AAAAAAT 2515

*msg*42 GGG---AATATCTAATGTTTCTGAATGTGAAGAAAAAATG---AAAAAAT 2552

* * **** * * **** * ** * * ** **

*msg*101 GCAATAATACTATTCTGGAAAAAACAATGAATGAGCTATGTACTGATATT 2679

*msg*54 GCAATAATATTACTGTGAAAAAAACAATGAATGGGCTATGTACTGATGTT 2599

*msg*47 GCAAAAATACTACTATGAAAGAAAAAATGAATGAGCTATGTACTAAAGTT 2608

*msg*28 GCGAGAATTCTACTATAAAACAAATAATAAATATATTATGTACTAATGCA 2592

*msg*97 GTGAAAATTCTACTACAAAACAAAAAATGAATATATCATGCACTAATATT 2617

*msg*22 GTAAAAATTTTACTGTGAAAA---AAATGAATATATCATGCACTGACGTC 2589

*msg*11 GTAAAAATTCAGCAATCGAAAAGGA---AAGGCAACTATGTGACGGCTCT 2562

*msg*42 GTAAAAATTCTGCAGACCAAGAGAA---AAAAAAACTATGCAACGGCTCT 2599

* * *** ** * ***

*msg*101 AATGATACATGCAAAATTTTGAAATTACATTTAGAAGAAATATGTGATCA 2729

*msg*54 AATGATACATGTAGAATTTTGAAATTACATTTAGAAGAAATATGTGATCA 2649

*msg*47 AATGATACATGCAAAATTTTGGAATTGCATTTAGAAAAAATATGTGATCA 2658

*msg*28 GATGATACATGCAAACATTTAAGAAAACATTTAGAAAAAGGTTGTGATCA 2642

*msg*97 AATAATACATGCAAGCATCTAATAGACCATTTAGGAAAAATATGTCATGG 2667

*msg*22 AATAATACATGCAAGCATTTAGTGGATCATTTAA-GGAAATTTGTGATCA 2638

*msg*11 ATT---CAATGTGAAGCTTTAATATCATATTTAGAAAAAATGTGTGATAC 2609

*msg*42 ATT---CAATGTGGAACTTTAATATCATATTTAGAAAAAGTATGTGATGA 2646

* *** * * ***** ** *** **

*msg*101 GCTTACATTAAAAATTTTTAAATTTTTGTTTACTAATTCTAAATCTAAAA 2779

*msg*54 GCTTACATTAAAAATCTTTAAATTTTTGTTTACTAATTCTAAATCTAAAA 2699

*msg*47 GCTTGCATTAAAAATCTTTAAATTTTTGTTTACTAATTCTAAATCTGAAA 2708

*msg*28 GCTTGCATTAGAAATCTATAAGTTCTTATTTGCAAATTCTAATTCTACTA 2692

*msg*97 ACTTGCATTGAAAATTTTTAAATTTTTATCAACCCCT------TCTAATT 2711

*msg*22 TCTTGCAT--AAAATTTTTAAACTTTTATCTACTAATTTTAATTCTACTA 2686

*msg*11 ACTCGCATTAAAAGTATCTCTATATTATCATACTAAACCTCATTCTAGCA 2659

*msg*42 ACTCTTATTAAAAGTATCTCGATATTATTATACTAAACGTCATTCTAATA 2696

** ** ** * * * * * ***

*msg*101 TAGAATGTCAGAAATTAACACCTTTATGTTCATCTATAGCATCTTCTTGC 2829

*msg*54 TAGAATGTCAGAAATTAACACCTTTATGTTCATCTATAGCATCTTCTTGC 2749

*msg*47 TAGAATGTCAGAAATTAACACCTTTATGTTCATCTATAGCATCTTCTTGC 2758

*msg*28 CAGAGTGTAAAAATTTAGCATCTATATGTTCATCTATAGGTTATTCTTGT 2742

*msg*97 CAGAGTGTGAAAATTTGAAATCCTTATGCTCATCTATAGGAAATTTTTGT 2761

*msg*22 TAGAATGTCAGAAATTAATATCCTCATGTTCATTTATAGGGTCTTCTTGT 2736

*msg*11 CGGAGTGTAAAAAACTAGAATCTTTATGTTCATCTATAGGGTCTTCTTGT 2709

*msg*42 TGGAGTGTAAAAAACTAAAATCTTTATGTTCATCTATAGGGTCTTCTTGT 2746

** *** * ** * * * *** **** ***** ** ***

*msg*101 AATGAAACGAATGTTAAAATTCTTCCTTTATGTCATAACTTTACTAGTGT 2879

*msg*54 AATGAAACGAATGTTAAAATTCTTCCTTTATGTCATAACTTTACTAGTGT 2799

*msg*47 AATGAAACTAATGTTAAAATTCTTCCTTTATGTCATAACTTTACTAGTGT 2808

*msg*28 GGTAAAATTAATAATAAACTTGCTACTATATGTAGTAACTTTACTATTAA 2792

*msg*97 AATGGAATTAACGATAAAATCATTCGTATATGTAATAGCACTATGACTAA 2811

*msg*22 AATGGAATTAACGACAAAATTACTCGTATATGTAACAACACTATAACTAA 2786

*msg*11 ACTGGGGTTAACAGTAGAGTTTCTTATATATGCACTAAGCTTGCTATTAA 2759

*msg*42 ACTGGGGTTAACAATAGAGTTTCTTCTGTATGTGATAAGTTTATTCTTGA 2796

* ** * * * * * **** * * *

*msg*101 ATGCAGCA---A------------------ATTAC---TT------GAAC 2899

*msg*54 ATGCAAAT---TACTTGA---------ACCGCCAAAACCA------CCAG 2831

*msg*47 ATGCAGCAAATTACTTGA---------ACCGCCAAAACCA------CCAG 2843

*msg*28 ATGCAAGT---TATTAGAGTCAATGCTACCACCACCTCCACCATCGCCAT 2839

*msg*97 ATGCACTT---CACCAG------------GCCCTCTACCT------CCAC 2840

*msg*22 ATGTAGTT---CACCAT------------CACCACCACCA------GGAC 2815

*msg*11 ATGCAAGT---TACCACA---------GCCACCACCACCA------GTA- 2790

*msg*42 CTGCAAGT---CACCACA---------ACCACATCCACCA------TCAC 2828

** * *

*msg*101 AGCC---AAA---ACCTTCAGAATTACCAG---AATTA---------CCA 2931

*msg*54 CACCACCAGC---ACCATCAGAACCATCAG---AACCA---------CCA 2866

*msg*47 CACCACCAGC---ACCACCAGCACCACCAG---CACCA---------CCA 2878

*msg*28 TACCACCACTACCACCATCAGAACCATCAG---AACCAATACCGCCACCA 2886

*msg*97 AACCACAACC---ACCATCAGAGCCGCCAC---CACCA---------CCA 2875

*msg*22 CACCACCACC---AGTACCACGAC---CGC---AGCCA---------CCA 2847

*msg*11 --CCACCACA---ACCACCACAACCGCCGTCGCCACCA---------CTG 2826

*msg*42 CACCACCACT---ACCGCCACCACAACCAC---ATCCA---------CCA 2863

** * * ** * * *

*msg*101 GAACCATTAGAACCA---TTAGA------------------A---CCATC 2957

*msg*54 GCACCATCAGAGCCA---TCAGAAT------CACCAGAAACA---CCATC 2904

*msg*47 GCACCATCAGAACCA---TCAGAAA------CACCAGAAACA---CCATC 2916

*msg*28 AAACCAATACCGCCA---TCAGAACCAATACCACCACCAGCA---CCATC 2930

*msg*97 GAACCATTA---CCA---CCAAAAC------CGCCTATACCACCACCACC 2913

*msg*22 AAACCA------------CCACA---------ACCACAACCA---CATCC 2873

*msg*11 CCGCAACCACATCCACAACCACAGC------CACCAG---AA---CCACC 2864

*msg*42 TCGCCACCAC------AACCACATC------CACCAT---CG---CCACC 2895

* * * * * *

*msg*101 AGAACC---A---------TCAAA------A---CCATCAAAACCAGAGA 2986

*msg*54 AGAGCC---AG------TACCAAA------A---CCACCGGAACCAGAGA 2936

*msg*47 AGAACC---AG------TACCAAA------A---CCACCGGAACCAGAGA 2948

*msg*28 AGAACC---ATCAACACCACCAAAACCAATACCACCATCAGAACCAATAC 2977

*msg*97 AAAGCCATCAG------AACCAGA------A---TCATCAAAACCAATGC 2948

*msg*22 ACAGC---CAC------CACCGCC------A---CCACCGCCACCACCGC 2905

*msg*11 AGAACC---AC------CACCAAA------A---CCACCAAAACCACAAC 2896

*msg*42 ACAACC---AC---ATCCACCATC------G---CCACCACAACCACAT- 2929

* * * * * ** * ****

*msg*101 TA---------------------------CCG---CTAGAACC---AGCA 3003

*msg*54 CA---------------------------CCA---CCAGAACCACCAGCA 2956

*msg*47 CA---------------------------CCA---CCAGCACCATCAGAA 2968

*msg*28 CACCACCAGCACCACCTGCACCACCTGCACCA---CCTGCACCACCTGCA 3024

*msg*97 CA---------------------------AAA---CCAAAACCAACAAAT 2968

*msg*22 CA---------------------------CCA---CCAAAACCATCAGAA 2925

*msg*11 CG---------------------------CCACCACCATCGCCACCAGAA 2919

*msg*42 --------------------------------CCACCATCGCCACCACAA 2947

* **

*msg*101 CCAA---A------ACCATCAAAACCACC------------------AGC 3026

*msg*54 CCAG---A---GACGCCATCAGAACCAGCACC---------------AAA 2985

*msg*47 CCAT---C---AGAACCATCAGAAACACCAGA---------------AAC 2997

*msg*28 CCAT---C---AGAACCATCAACACCACCTGCACCACCTGCACCACCTGC 3068

*msg*97 TCA----------------------------------------------- 2971

*msg*22 CCAG---------AATCATCAAAACCAAAACC---------------GAA 2951

*msg*11 CCAC---AACCTCCACCACCAAAGCCACAACC---AC------------A 2951

*msg*42 CCACCGGAGCCACAACCACCGGAGCCACAACC---GCC---------GGA 2985

**

*msg*101 ACCATCAGAAC--------------------------------------- 3037

*msg*54 ACCACCAGAATCA------------------TCAACACCAGAGAC---TC 3014

*msg*47 ACCATCAGAACC---------------------AGTACCA---AA---AC 3020

*msg*28 ACCATCAGAACCA------------------TCAACACCATCAAC---AC 3097

*msg*97 -------------ACTACAAGTTTACCGACTACAAATTCATCAAC----- 3003

*msg*22 ACCGACAAACTCTACTACAAGTTTACCAACTACGAATTCATCAAC----- 2996

*msg*11 GCCGCCATCGCCA------------------CCAAAACCACTACCGCCAC 2983

*msg*42 GTCACCTGTGCCA------------------TCAACACCACCAAAACCAC 3017

*msg*101 ------------CG---CCAGGAT---------CACCAGAATCA---C-- 3058

*msg*54 ---CATCAGAACCA---TCAGAAC---------CACCAGAATCA---C-- 3044

*msg*47 ---CACCGGAACCA---GAGACAC---------CACCAGCACCA---C-- 3050

*msg*28 ---CATCAGCACCA---CCTACATCGCTACCACCGTCAAAACCA---GTT 3138

*msg*97 -------------T---GCAGCTT---------CGCCAACTACG---C-- 3023

*msg*22 -------------T---GCAGCTC---------CGCCAATTACA---T-- 3016

*msg*11 ---CACCAGAGCCA---CCAAAAC---------CATCAGAACCAGATC-- 3016

*msg*42 CTAAACCAAAGCCAAAACCAAAAC---------CGACAAATTCAACTA-- 3056

* ** *

*msg*101 -C------AGAATTGCCAG------------------------AGCCAAA 3077

*msg*54 -C------AGAATTGCCAG------------------------AGCCAAA 3063

*msg*47 -C------AGAATTGCCAG------------------------AGCCAAA 3069

*msg*28 CCATTGCCATCATCGTCACAATCTGTCTTA------AGACCAAC---AGA 3179

*msg*97 -CA------AGCTTTCCAAC---------TACACTTATACCAACTACAAA 3057

*msg*22 -CA------AGCTTGCCAAC---------TACACTTATACC--------- 3041

*msg*11 -C------ATCAAGACCAA------------------------AGCCAAA 3035

*msg*42 -C------AAGTTTACCGA------------------------CTACAGA 3075

* *

*msg*101 ACCA---------------------ACACTGACAAATT---TAAC---TG 3100

*msg*54 ACCA---------------------ACACTGACAAATT---CAAC---TA 3086

*msg*47 ACCA---------------------ACACTGACAAATT---CAAC---TA 3092

*msg*28 ATCATCAACTACATCAAGTTCATCAACTTTTTCAAATT---CATCAATTA 3226

*msg*97 CACATCA-----------------GATATATACACA-T---CAAC---TA 3083

*msg*22 ------------------------------------------AAC---TA 3046

*msg*11 ACCA---------------------AAACCGACAAATT---CAAC---TA 3058

*msg*42 TTCA---------------------TCACCAGCAGCTCCATCAAC---TA 3101

* * *

*msg*101 T---------AATATTAA---CAAGTACA---------C---------AA 3120

*msg*54 C---------AATATTAA---CAAGTACA---------C---------AA 3106

*msg*47 C---------AATATTAA---CAAGTACA---------C---------AA 3112

*msg*28 T------ATCAAGTTTAACTTCAACTACATCAGAATCATC--------GA 3262

*msg*97 CAG---CCACATCGGAAA---CAAGTATA---------TT---------A 3109

*msg*22 CAA---ACACATC---------AGATATA---------TA---------C 3066

*msg*11 CA---------AGCTTAACTACAAGTTTA---------CCGACTGCAGAT 3090

*msg*42 CATTGGATACAGACACACCTACACGTACA---------TCAG-CACATAC 3141

* * *

*msg*101 -TTG--TT---------AACAATA-------------------------- 3132

*msg*54 -TTG--TT---------AACGATA-------------------------- 3118

*msg*47 -TTG--TT---------AACGATA-------------------------- 3124

*msg*28 -TTGTGTTAGATTCATCTATTACATATAATTCATCATCTGCATCAAGTTC 3311

*msg*97 ---G--A----TA---CAAGCACA-------------------------- 3121

*msg*22 -CCA--TCAACTA---CAAGCACA-------------------------- 3084

*msg*11 -TCA--TC---------AACTGCA-------------------------- 3102

*msg*42 ATCA--TC---------ACGTACA-------------------------- 3154

*

*msg*101 -------TA---TTTACCAACTACATCAAC-------------------- 3152

*msg*54 -------TG---TTTACCAACTACATCAGCTAC---------------GC 3143

*msg*47 -------TG---TTTACCAACTACATCAGCTACACATTCATCAGCTACGC 3164

*msg*28 AATTACATCGAATTTACCAATTACATCAAATATCTC-------------- 3347

*msg*97 -------TC-----TA-CAGATACATC---TACACGCACATC---TACAC 3152

*msg*22 -------TC---TAAAACAAGTACATTAGATACAAGCACATC---TACAG 3121

*msg*11 -------GC---TCCACCAACTACATTG------------GA---TACAG 3127

*msg*42 -------T------CATCACGTACATCAACTGCACGTACGTC---TACAG 3188

* ** *****

*msg*101 ----------------------------TATGCATTCATCAGCTACACGT 3174

*msg*54 ATTCATCAGCTACGCATTCAT---CAGTTACACGTTCATCAACTACACGT 3190

*msg*47 ATTCATCAGCTACACGTTCA------ACTATACGT---TCAACTATACGT 3205

*msg*28 -TA---CATTAACAAATTCAACTATATTAACAGGCTCATTAACCACACAT 3393

*msg*97 GCACAT---CTACACGCACA------TCTAAAGGTACATCGACTACACGT 3193

*msg*22 ATACAT---CTACACGCACA------TCTAAAGATACATCGACTACACGT 3162

*msg*11 ACACAT---CTTCACGTACA------TCTTCACATACATCAACTACACGT 3168

*msg*42 ATACAT---CTACAGATACA------TCTACAGATACATCAGCTACAGGC 3229

* * * *

*msg*101 TC---AA---------CTACACGCTCA---T------------------T 3191

*msg*54 TC---AA---------CTACACGTTCA---T------------------T 3207

*msg*47 TC---AA---------CTACACGTTCA---T------------------T 3222

*msg*28 TTAACATCTACATATTTAACATCTACACGTTTGACATCTATATATTCATC 3443

*msg*97 TC---AT---------CAACATCCACGCGTT------------------C 3213

*msg*22 TC---AT---------CAACATCCACACGTT------------------C 3182

*msg*11 AC---AT---------CAACATCTACACGTT------------------C 3188

*msg*42 TC---AC---------CAACATCTACACGTT------------------T 3249

* *** * *

*msg*101 AAGGCCTAAACCAACGACATCCTCTGACGATAGACGTATAGTGGGCTTTG 3241

*msg*54 AAGGCCTAAACCAACGACATCCTCTGACGATAGACATATAGTGGGCTTTG 3257

*msg*47 AAGGCCTAAACCAACTACATCCTCTGATGATAGACGTACAGTAGGCTTTG 3272

*msg*28 AAAGTCTGTACTAACAACATCATCC--**-**GATAGCCACGAAAAAGGCTATG 3490

*msg*97 AAGACCTAGACCAACAATGTCACGG--**-**GATGACTATGAAGAAGGTTATG 3260

*msg*22 AAGACCTAGACCAACAATGTCAC-G--**-**GATGACTAT-GAGAAGGTTATG 3227

*msg*11 AAGGCCTAGACCAACAATAACACCCGGTGGTGGGCGAGGGAAAGGATATG 3238

*msg*42 AAGACCTAGACCAACAATATCACCCGGTGATGGACGAGGAAAAGGATATG 3299

** ** ** *** * * * * ** * **

*msg*101 GAGTTAGGCCAAGAAAATTAAGGGAAATAGAGTTAATCTGGATGGTTGCA 3291

*msg*54 GAGTTAGGCCAAGAAAATTAAGGGAAATAGAGTTAATCTGGATGGTTGCA 3307

*msg*47 GAATTAGGCCAAGAAAATTAAAGAGAATAGAGTTAATCTGTATGGTTGTA 3322

*msg*28 GAATTAAAAT---AGAACTACAAATGATAAAATTAATTTGGACAACCATA 3537

*msg*97 GACTCAGAGCACAGAGATTACAAATGATAAAATTGATTTTGGAAATTGCA 3310

*msg*22 GACTCAGAGCACAGGGATTACAAATGATAAAATTGATTTGGA-AATTGTA 3276

*msg*11 GAATCAGAACACAAGGACTACAGATAGTAGAATTAATTTGGACGACTATA 3288

*msg*42 GAATTAGAACACAAGGACTACAGATAATAGAATTGATTTGGATGACTATA 3349

** * * * ** ** * ** ** * *

*msg*101 GGAACAATACTGGGAATGTGGATAATTGT---T**TAA** 3324

*msg*54 GGAACAATACTGGGAATGTGGATAATTGT---T**TAA** 3340

*msg*47 GGAACAATATTGGGAATGTGGATAATTAT---T**TAA** 3355

*msg*28 GAAATACTATTAGGGTTATGGATAATTGTTATA**TAA** 3573

*msg*97 GGAATAATATTGGGACTATGGATAATTAT---T**TAA** 3343

*msg*22 GGAATAATATTGGGACTATGGATAATTAT---T**TAA** 3309

*msg*11 GGAATAATATTAGGGCTATGGATAATTAT---T**TAA** 3321

*msg*42 GGAATAATACTAGGGCTATGGATAGTTAT---T**TAA** 3382

* ** * ** * ** * ****** ** * ***

***msg*-VI**

**upstream potential TATA box**

*msg*49 TTAAA**T---ATAAAC**AT-TATTA-CAACAATATTAAAA---**TATAATA**AT 42

*msg*51 ATATTATAGTTTAGGAT-TTTTTGATAAAGTACATAAA--AACTAGTATT 47

*msg*73 TAATTG---ACTAA--T-T**TATAAAT**AAAACATTTTAA--TAAAAATTTT 42

*msg*39 AACTTT---TTAAATGT-TTTTA----AATTACTT-----------CATT 31

*msg*59 TCAAAACAACTAAAGAA-TAATA--TACAATACAA---AACAATATTAGT 44

*msg*18 TATTAA---ATAAAGTTGTAATA-TTAAAGTTTTCCATCATATTATTATA 46

* * * *

*msg*49 T---TAAAATAAAATAAT-ACCAT---TCAAAACTTTTT-AGAA-AATTA 83

*msg*51 GA-A**TATA---AAT**TAAT-ATTATTTCTTAAAAT-TTTT-ATAC-TATTC 89

*msg*73 AAAAGGTA---GA-------TTATT--TTGAGAT-T-TT-ATGT-TATTA 76

*msg*39 T---TAAA-------AAT-ATCTT---TTAAAAATATC----------TA 57

*msg*59 T--A**TATA---AAT**ATTTAAAAAT---TTAAGTTACTTTAAAAATGCTCA 86

*msg*18 T--TTATA---GATATATAGACAA---ATAAACA-**TATAAAA**AA-AATTA 86

* *

*msg*49 TTTTTAA---A**TATAAAT**TTTATCTAAAAATTCT--TTGGAATTTA**TATA** 128

*msg*51 TATTTAAT--ATGAA-ATTCGAAATAA--ATTTA--CAAAAAATGATTTA 132

*msg*73 CATTTTATACAGAAAAATGTAATATAT--A**TATATATATATA**AC--CATA 122

*msg*39 C-TTTAA---ATA-AAATCATAATTGA--ATT**TA--TAAAA**------AAA 92

*msg*59 T-TTTGG-----A-AAAGCCAAACTAA--ATCTT---ATGAAA--ATAGA 122

*msg*18 TGTTTGT-----T-AAAGAAGGAATAA--AAGGT---AGTAA**T--AT--A** 121

*** * * * * *

*msg*49 **AAA**ATCCAAATAATTCAA-TAAAGATG--AT-C-----AAAATAGTCAAT 169

*msg*51 AA-CTTATTATAAAGTATTTAAAAATC--TT-TCTAATAAAAATACT--- 175

*msg*73 GA-TTTAGT-AAAATCATTAAAAAAAT--AAAAGAACTAAATATAATCT- 167

*msg*39 AAAATCATATTAATTTGT-TAAAAATG--AT-T-----AAAATAGACAAT 133

*msg*59 AA-TATATTAGAAATTATTTAAATAAAAAAT-AAAAACAAAATTAACAT- 169

*msg*18 **AAA**GTCAAAACAATGTATTTATTTTTG--AT-T---TCATGTTTTATACT 165

* ** * *

*msg*49 AA---------------AGAA-ATAATA-------A-CAATAATTTATTA 195

*msg*51 -------------------TTATTATCT-------AATTAATATTTGTTA 199

*msg*73 CT---------------ATTTACTATTT-------ATCTATTATCTATTA 195

*msg*39 AGATATCATTAAAAATAAAAT-TTATTTATTCTTTGTTTATCATTATATG 182

*msg*59 CC---------------ACAT-TTATTT-------ATTTATTATTTGTTC 196

*msg*18 GA---------------ATAT-TTATTT-------GCAAATGATTTCTTA 192

** * ** *

*msg*49 TTTAATAT------TATTTTATT-TCACG--TGACTTTATTTTT-TTCGT 235

*msg*51 TCTATTCTTGATAACATTTTCCG--------TAATCGGG-ATAC-G-A-T 237

*msg*73 TATATTAAT-----TATTTGCTG-TCAAACCAAATCGGA-ACAG-CTC-C 236

*msg*39 CATACTTT------TTTTTAAAA-ATATATTGTATTACG-AATT-TTC-G 222

*msg*59 ---TATAT------TTTTTAATA-TTATCTAGTACTACG-ATTT-TTC-G 233

*msg*18 TTTTATTT------TAAATTACAAGGATACAGGGATAAA-ATCCTTACAG 235

* *

**TATA box** **Cap signal**

*msg*49 TGAACTTT-TTCT--AA--AAAATTAACGA--ATTT**CATAAAA**AATAA**CA** 278

*msg*51 TAAA-------**CATATTT**CGATGTCAAAGAGCTCTC-TC**CA**CGAAATA-- 277

*msg*73 TGAATT-TTTTACATCG-------GAAACACCCTTT-**TACAAAA**ATAA-- 275

*msg*39 TAAGTTTT-TTTCTTAAAAAAAATCAAGAT-----T**TATAAGA**ACATACG 266

*msg*59 TAAGTTTT-TTTTCC**TACAAAA**ATTAACAA----AC-TTAAAAATT**CA**CG 277

*msg*18 TA-ACGTT-TAAT---A--ATATTTAAAAAAGACAC**TATAAAA**ATGTA**CA** 278

* * ** * * *

*msg*49 ACTCTTTTTCTT--T**CA**--TACTG--TTACCG**ATG**AAAACTTCTGTATTT 322

*msg*51 -CTTTAATTAAT--TAA--TACTACTTCATGA**ATG**AAGGCCTCAATAATT 322

*msg*73 -**CA**TTATTCTCT--TATTCTATTGCTTAATGA**ATG**AAGCTCCCAATATTC 322

*msg*39 G-ACT**CA**TTTCCCCTAT--TA-TT--TTACTA**ATG**AGGGCCTCCGTATTT 310

*msg*59 -GGCTTGTCCCC--T----CACTT--TTATTA**ATG**AAAATCTCCGTGTTT 318

*msg*18 -CGTTAATTA**CT**--TAT--TGTTG--GAGCAA**ATG**AGAGTCTTTATATAT 321

* * * * **** *

**<------**

*msg*49 TTAGTTTTTATTGGAACAACATGCGTTTTATCAAAGGGGATTGTTACACC 372

*msg*51 GCTATACTTGTTGGAGTTACTCTCGTTTTTTCTAAAAATAATG**GTATTT**A 372

*msg*73 TCTATATTTCTGAGTATTACTTGTGTTCTTTCAAGGGCTGATG**GTATTT**T 372

*msg*39 GCATTTTTTGTAGGGGTAGTACGTGCTCTTTTAAAAGATATAAATATTGC 360

*msg*59 ATATTTTTTATTGGAATAGTACACGCCCTTTTAAGAGATATAGGTGTTTC 368

*msg*18 GGGATTTTTATTGCATTTACAAATGCTTTGTCAGAAAAA------ATTTC 365

* ** * * * *

**-------------------------intron-------------------**

*msg*49 AA**GT---AATT**TTAT-CTT--TC----C---TTTTTATTTTTTAACTTTT 409

*msg*51 AGAT-------------TC--TT----T---TTTTCAAATTTTGACCTT- 399

*msg*73 TGAT---TGTTTCTA-GTT--TT----T---TTGCTTTTTTTTAACTTT- 408

*msg*39 AG**GTATCT**TTTCTTT-TTTTTTG----T---TTTTT-----TTTTTTTTT 397

*msg*59 TG**GTATTT**ATTTTTTGTTT--TTGTTTTTTTTTTTTTTTTTTTTACAT-- 414

*msg*18 AG**GT---TATT**TTAT-TTT--TT----CT-TTTATGAATTTTTAACTC-- 402

* * * ** **

**----->**

*msg*49 TTT**TAG**GACATCAAGA-TAAATC-----TTCTACCCTCAAAAA-AA--CT 450

*msg*51 --T**TAG**AACGCGATTATGCTA------GTTCTATTTTGAAAAG-AG--AT 438

*msg*73 TTT**TAG**AATCCAAAAAT---A------ATCATATTTCAAATAG-AA--AG 446

*msg*39 TTTTAAAAT**TAG**CGTATGAAAATTCAGACAATATTTTGGAT---AA--T- 441

*msg*59 TTT**TAG**AATTTACATATGAAAATTTAAACTCTATTTTGGACAACAAAAAA 464

*msg*18 TTT**AAG**ATTCTGCAAGTCAAAATTTAAAT---ATTTTAGATAATGAAG-- 447

* * * * *

*msg*49 CAACTAAATCATGATCATATTCTTGTGGACTATGCATATGATG-ATCCG- 498

*msg*51 AATTTGAATTATTATCGCGTTACTA---------GAGATTTTT-TTCCAA 478

*msg*73 TTTTTGAATGATGACGAAGCTTTTG---------CACATTTTT-CACAAG 486

*msg*39 -------------------------------------------------- 441

*msg*59 TACATTGATGATGA--------TGATAC-CTACTCATATATCT-CTCTTA 504

*msg*18 ---AAAAATTAATA---TTTAACCATGT-ATATCCATATAAGTCATTT-G 489

*msg*49 GAAAATGGAGTGAAT-TCGGTTAATAAACTTACAAAAGA-ACTTAATGAA 546

*msg*51 GAGAATC-AAGAGAT-TCACTAAATAAGCTTACAGGAAA-TCTTAAGAAA 525

*msg*73 GGGATTC-TCCAGACTTTAAT-AAGAAGATTAAAAAAGA-CCTTTTAGAA 533

*msg*39 -------------------------------------------------- 441

*msg*59 AACAATC-ATTGTAT-TCA----CTAACTCAGGATGATAATTTATTTGAA 548

*msg*18 AGAAACGGAGTA-AT-TTT----ATGAGTTTTGATGAGTATATTAATAAA 533

*msg*49 ATTTTGGATAAAGTCAGTC--TTGTTTTAGATAATAAAATTTACTGGAAA 594

*msg*51 ATACTGGAAGAAGTTGAT--AGTCTTTTAAATGGTAAAATACAGTGGAAA 573

*msg*73 ATGAAGTCTAAATTTGATGAAATCATTTCTACTTTACAAAACCAA-CA-- 580

*msg*39 -------------------------------------------------- 441

*msg*59 TTAATAAGAGACTTTCAAGTTACATTTCAGACCAAACA--AAACCCAGAA 596

*msg*18 CAATTAAATGAAATTCAAAAT--TTCTTACATAAAAT---TAACCTCGAA 578

*msg*49 AGA--TATGATTTTTCGGATGATAGCTCCTCAA-AGACGG---AGGGATA 638

*msg*51 GAA--CATGATTCCTTAGACGAT---TCTTCAA-ATGCAG---AAAAATA 614

*msg*73 --A--CAAAATAATTTAGATGAT---TCTTTAA-AACCAC---TAG-CT- 617

*msg*39 -------------------------------------------------- 441

*msg*59 AGTGAAACTATTG---AGAAACTAGCACCTCGATCCGCACCCGCAAAAGA 643

*msg*18 AAT--CATGATTT---AT----------CTCAA-G--------GAAATTA 604

*msg*49 TG--AT-AATATGAAGAAA--CAA-----------GATATCGCATTAAAA 672

*msg*51 TA--AT-ATTTTAGAAGAA--CAGAAT----------TCTAGAATTATGA 649

*msg*73 GA--GT-ATCTAAGTGGTC--GAAGTT----------TGGAGGATGATCT 652

*msg*39 ------------------------------------------------AC 443

*msg*59 TGGAGTTGCCCAAAAAGAG-GCAGTATCAACAGAAGATGTATATTTCTTA 692

*msg*18 TC--TT-ACTTCAGAAGATTACGGATT----------GCTGGCAGTAATA 641

*msg*49 AAT----A-TACTAGAAGATTATCTTAT-GAGTTACCATAATACAGATAA 716

*msg*51 -AT----A-TTCTGCAAGGCTATTTTAT-GAATTGCTATAGTACAGATGA 692

*msg*73 -TGTTTCATCTATGCTAAGTTACTTTAA-AGGACATAATACTATACAAGA 700

*msg*39 AGG----A-TACTTGGAAAATA---------------ATAA------TAT 467

*msg*59 -TT----ACTTCTAGCAAATT-TCTTACAAAGACCTC---TTACTCATCA 733

*msg*18 -A---------TTG-CAACATATTTTAA-TCGTAAATATACTGTAATTGA 679

* * *

*msg*49 A-TGTCTACAA-ACAGTAA-ACG--CTTATC-GATCTCTTCTTGCAAC-T 759

*msg*51 A-TGTGCAAGA-ACAATAC-AAG--ATTATC-GCTCTGCTCTTAAGAC-T 735

*msg*73 A-TGCA--AAA-TTTTTCA-AAAAATATATT-CATCTGCTCTTAACAAAT 744

*msg*39 A-TATTT-----------------------------------AAATAG-T 480

*msg*59 ATTGTGTGTGACTGTATTA-ACG--AACATCTGCCCTACTATCAATAA-T 779

*msg*18 A-TGTGTAAAT-ATTTTGACTCG--ATTTT--G-TCA--ACATATGAA-T 719

* * * *

*msg*49 TT--TAAAGA---TAGAAATGAAGAAGTAG----CTAAACTGGATTCATT 800

*msg*51 TT--TGAAAA---TAAAGGGGAAGCATTTG----CTAGGCTAAATTCATT 776

*msg*73 TTGATAAGGATAATAAAGATGATGCTATGG----ATAAACTGCATTCACT 790

*msg*39 CC--CAAAAA---TCA---CGAAGAAT-------CAA-----AATTTGTT 510

*msg*59 TA--TTCTGC---CACAGGAGCTGTATCTAGTGACTTTATAACAAAAAGA 824

*msg*18 TC--TATACC---AAAAAATGAT--AATCG----CTTAAC-GAGTTTATC 757

*

*msg*49 GCT-TTCAAATAAAGATCTG---------TGCGATAAGGTATTAAAACAT 840

*msg*51 ATT-TTCAGATAAAGATATA---------TGTGACAAAATATTGAAGCAT 816

*msg*73 TCT-TTCAAAGGATGATTTG---------TGTGAAAAGTTATTAAAACAT 830

*msg*39 AATACTCACA--CCCATGTT---------GCTGATAAAACA--------- 540

*msg*59 TGT-GCCAATGTAACACTGACA-------TGTCTATATTTAGTGAAAAAA 866

*msg*18 TTT-TGCATGTAATAATCCAACAGAAACATGTAAAAAAACGATACATTTT 806

* ** * *

*msg*49 TTAGATAAATTATGTATTAGTATTAAAAAAGAACAACTTGCAATTAAAAA 890

*msg*51 CTGGATAAATTATGTACTATTATTAGAGAAGAACAAAATGCAATTAATGA 866

*msg*73 TTACAAAGTATATGTCTTAACATTAAAAATGAACATGATTCAATCGAAAA 880

*msg*39 -------GAATATGTAA------CAGAGGT--GT--TATACCA----TAC 569

*msg*59 ATGGATGAATACTGTCAAAAGATTAAAGAAGAACAAAATAACGCTCTTTC 916

*msg*18 TTTGATGAAATGTGTAGAGAACTTGAAGAATTCTTAGAAAAAACAAGCGA 856

*** *

*msg*49 TTTCACAGAAGATGTTTGTCGTACTGAACTTTCAAAATGTCAGGATCTAG 940

*msg*51 TTTTACAAAGGATGTTTGTCTTATTCAGCTTTCAAAATGTCATGAGCTAG 916

*msg*73 ATATACCCAGGAAATTTGTGATCTTCATCTTACAAAATGTCGTGATTTGC 930

*msg*39 TCATACAGAAGTG-GTATTCACAATAA-----GAAAATGTTG-----TTC 608

*msg*59 TGTTACAAAAGAGGTTTGCACTTATCATGTTGGTAACTGCAAATATTTAG 966

*msg*18 ATTTACAAATTATGTATGTCGAAAAGAAAATCTGGTATGTAAATACTTAG 906

** * * ** *

*msg*49 AGGAAAGTTGTAATGATAC-TCTAAAAG-ATTCATGTAAGACTGTAAGAA 988

*msg*51 AGGAAAATTGTAAAAATAT-TTTAAAAG-ATTCATGCAAAAATGTAAAAG 964

*msg*73 AAGATTCATGTGGTGAACC-TTTGAAAG-GATCATGTAAAAAGTTAGAAG 978

*msg*39 AGGTAAATCA------TGG-CGTAAAAG---TTGTGCAAAAG--GGAAAG 646

*msg*59 AAAAAGCATGTGGGGAGAG-CCTAAAAA-AATCGTGCACTAAAATCAAGA 1014

*msg*18 GGAAAACATGCGGAAATAAGCTTACAACTATCTGTGAAAGAC--TGGGAA 954

* ** ** * *

*msg*49 ATAACTGTAAA---------------------GATTA------TATTCC- 1010

*msg*51 AAAAATGCAAAG----AG-----------------AAAAC---TATTCT- 989

*msg*73 AAAAATGTAGAAAAACAGAACATAGTACTGAGGACAAAACTCACGTTCA- 1027

*msg*39 T-TACTTCAA-----------------------A---------AACTACT 663

*msg*59 CTCAATGCAAAAA-ACTGGAAA--------ACGACTC---TAAAGTTCA- 1051

*msg*18 CTCATTGTGAAAA-A-------------------------CCAAA-TGA- 976

* * *

*msg*49 TATGAAAGA---TGC---GTTTGTTGTAACTCATACTGTACTTATTA-TA 1053

*msg*51 TTCAGGGGA---TAT---ATTTACTATGACTCATACTGTGCTTGTTA-TA 1032

*msg*73 TCCAAAAGA---TCT---ATTTGTTAAAGTTCATACTGTGCTAACTA-CG 1070

*msg*39 TCTCAT-------------------GCA------ACTGTTTGTCCCA-CC 687

*msg*59 T---GAAGA---TACGGGTTTTGTTAATACTCACACGCAAATTGTTG-AT 1094

*msg*18 AACAGAACATAAGACA---ATTTCTACAACACAGAGTGAATATATTAGTT 1023

*

*msg*49 TCAACAACTGTAACAACAGAAGTGGCACCTGAAACATCAACTTATGTAGT 1103

*msg*51 TCAACAACAGTAACCACAGAAATAGCGCCTGAAACATCAACTTGTGTAGA 1082

*msg*73 CATACTATAGTAGCAATGGAGACTGTAATTGCCCCAGATATTTATGTAGA 1120

*msg*39 CCAACCAGAGAA---TCAGATCCAGAACCAGAACCA---ACAGATTCAGA 731

*msg*59 GAAACAGTATACGTGACAGAAACAGTATATTATACTCATACATCTGTAA- 1143

*msg*18 TTGAAAATACTCATACTATAA----TTCTTGATGAAACAATTTACATAA- 1068

* * * *

*msg*49 AACAGAAACA----------GTGGCAG--------------------AAG 1123

*msg*51 GACAATGGTA----------GTGACAG--------------------AAA 1102

*msg*73 AACGTTGATA----------ATAACAA--------------------AAA 1140

*msg*39 CTCAATGGA-----------GCCAC------------------------- 745

*msg*59 --TATTCACA-------------ACAA--------------------AAA 1158

*msg*18 --CAGAAACAGTTTATTCTACTCACACAAACGTAGTATTAAAAACAGTTG 1116

*

*msg*49 TATGTTGTACAGATA-CTCTACCTGCTACA---ACGGAGCCAGAACTTGA 1169

*msg*51 CATGTTGTATAGATA-GGCCTCCTGCTAC---CATGGAACCTGAACCTGA 1148

*msg*73 CATGTTGTTTGGGTA-GACCAGCTCCAACGCATACAGAACCGGAACCTGA 1189

*msg*39 CAAGCTCCACAGAA--GACCTTGAGGAAAC---AG--A---CGAACATGG 785

*msg*59 AATGTTGTTCAAAAAAAACTAAATACAAAACTTGTGCAA-CAAAACCAA- 1206

*msg*18 ATTGCAACTCAAAAGATACTGAAGATTGTACTTGTACAC-CGAATATGG- 1164

* * * *

*msg*49 GCCTGACGAACCAGAACCTGATGAGCCGAAGCCTGATGAGCCAGAGACAC 1219

*msg*51 G---------------CCAGA------GAAACCAGAAGAACCAACCTCA- 1176

*msg*73 G---------------CCTGAA---CCGGAACCTGAAGATCCAGAGGAA- 1220

*msg*39 GCCAAC------GGATACACCAGA---------------ACCAG------ 808

*msg*59 -------------CTACTTGTCCTGCACTA-TCTACTGGATCAGAAGC-- 1240

*msg*18 -------------CTATTTGTCCAACACC--------------------- 1180

*msg*49 CGGAGGTGCC-AAAGGAAACAGAGGGAACAGAAGAAATAGAG-------- 1260

*msg*51 ----TG-CCC-A---------------------GAA---CCTGAACCAAA 1196

*msg*73 --GGGGAACC-AGATGAAC------AACCAGGAGAA---GAGGAGCCAGA 1258

*msg*39 -------------------------AGC---------------------- 811

*msg*59 -----GTGCCCGACGGAAGCA---TCTTCAGAAGAT---CAT-------- 1271

*msg*18 --------------------------GACATTTAC--------------- 1189

*msg*49 -GAAACAGAGGAAGTAGAGGAAACAGAGGAAGTAGAGGAAACAGAGGAAA 1309

*msg*51 T---CCTGAAC--------------------------------------- 1204

*msg*73 GGAAGAAGAGC--------------------------------------- 1269

*msg*39 -------------------------------------------------- 811

*msg*59 -GATCCAGAGC--------------------------------------- 1281

*msg*18 ---TTTAGAAC--------------------------------------- 1197

*msg*49 CAGAACCAGAGCCAGAGCCAGAACCAGA--GCCAG-AACC----AGAACC 1352

*msg*51 ------------------CTGAACCTGA--ACCTG-AACCTG-A---ACC 1229

*msg*73 ------------------CAG--GAA-A--AG-AGGAGCCAG-AGGAAGA 1294

*msg*39 CGGAACCAGAGCCGGAACCAGAGCCGGA--ACCAG-AGCC----GGAACC 854

*msg*59 ------------------CTA--CAAAAACATCAG-AAGAT-GGTGATCT 1309

*msg*18 ------------------CAA--CAAAA--TCCAG-GAGCAAATCAAAGC 1224

* * *

*msg*49 AGAGCCAGAACCAG-AACC-----AGAACCAGAACCAGAA---------- 1386

*msg*51 CGAACCCGAACCTG-AACC-----TGAACCTGAACCTGAACCTGAACCCG 1273

*msg*73 AGAGCCAGGAAAAG-A--------GAAACCAGAGGAAGAAGAAAAGCCAG 1335

*msg*39 AGAGCCGGAACCAG-AGCC-----AGAACCAGAGCCAGAACC-------- 890

*msg*59 AGAACCTGTAGAAACATCTTCAGAAGATAATGATCT-------------- 1345

*msg*18 CGACTCC-AACAACTAGCTTAAAACCAACTGAATCCAAAAA--------- 1264

** * * * * *

*msg*49 --------CCAGA----------------------A-CCAGAACCAGAAC 1405

*msg*51 AACCCGAACCTGA----------------ACCCGAA-CCCGAACCTGAA- 1305

*msg*73 AAGAGGAGCCAGAGGAAGAAAAGCCGGA-AGGGGAG-CCAGAGGAAGAAA 1383

*msg*39 ----AGAGCCAGAACC----AGAGCCA------GAG-CCGGGACCAGAAC 925

*msg*59 ----TGAACCTACAGAAACATCTTCGGA----TGAG-TCAGAAACAGAAA 1386

*msg*18 ----CAAACCAAAAC-C---AAGTCCAACGT-CTAGCTTAGAAACAA--- 1302

** *

*msg*49 CAGAACC---AGAACCAGAACCAGAACCAGAACCAGAGCCAGGACCAGAG 1452

*msg*51 -----CC---CGAACCCGAACCCGAACCTGAACCTGAACCTGAACCTGAA 1347

*msg*73 AG---CC---GGAAGAGGAGCCAGAGGAAGAAGAAAAGCCAGAAGAGGAG 1427

*msg*39 CAACATC---TGATCCAA---CAGATCC---ACCTCAACCTACAGAGGAG 966

*msg*59 CGTCTTCAGAAGAACAGGAACCAGTAGAGGAACC------AGAACCAA-- 1428

*msg*18 ------CAG--AATCTAGAGACA-AATTAAAACCAAGTCTTACAAAAACA 1343

* * * * *

*msg*49 CCAGAGCCAGAGCCAGAGCCAGAGCCAGAGCC---AGAGCCAGA-ACCT- 1497

*msg*51 CCTG------AACCT------------GAGCC---TGAGCCTGA-ACCT- 1374

*msg*73 CCAGA---GGAAGAA------------AAGCCGGAAGATCAGGA-ACCT- 1460

*msg*39 CCGGC---------------------AGAGAC---CTATT--CG-ACCG- 988

*msg*59 -CAGA---AGAACCA--------------ACT---CT-TG--CAGAGCCG 1454

*msg*18 CCTGA---TAAA-----------------AAT---AT-AT--CA-ATTA- 1365

* * *

*msg*49 C-AACCAACAA---CTGTACCTTCAGAGGATGAACC---AACTA------ 1534

*msg*51 A-AAC-CAACAACAATG--CCATC-CGA-AGAT-GAACCAACTA------ 1411

*msg*73 G-A---GCCAGAGCCTGTGCCAGA-AGAACCGGATCAAGAAGAATCTGGT 1505

*msg*39 A-AAGTGATAGTGAGTGTTCATAC-ACGGAGACAGT---AACAA------ 1027

*msg*59 ACTGAAGATGAAGAATGTACGATT-ACAAAAACAATAACAATGA------ 1497

*msg*18 -TAAATGTTAAAGAATGTGAAATA-ACAACTACAATGACAATAA------ 1407

** ** *

*msg*49 ----CAGACGAT---TCGAGTTGTTCATATACAGAAATTGTAACAGTAAC 1577

*msg*51 ----CAGATGAT---TCTAGTTGTTCATATACGGAGATAGTAACAGTAAC 1454

*msg*73 GATTCAGATGAT---AAAGATTGT------ACAATTACAAAGACAACGAC 1546

*msg*39 ----TAAATGATGGATATGATG---C---AACAGTTACAGTAACAGTATC 1067

*msg*59 ----CAAACAGA---TTTGGAGAGACACTTACAACAACTGTAACAGTATC 1540

*msg*18 ----AAAATAAA---TTTAGTGAAACTATTACAATGACTGTAACAGTATC 1450

* * ** * *** *

*msg*49 ATCAGAACCTACTAATACAGATGACTCTGAAGTAGAGGATAAAGGTTATG 1627

*msg*51 ATCTAGTCCTACTAAAACAAGTGAGTCTGGAATAGAAAGTAAAAGTTATG 1504

*msg*73 AGTAACGCCTGAAGGTACAGAGAAACCTGGAATAGATGATAAAGGTTATG 1596

*msg*39 AGAAGAATC---------AGATAACAATGGAGTAAGTGATAAAGGGCACA 1108

*msg*59 AGAAAAGCCTGGGGAGACTAACAAAGATCAAATAAACAATAAAGGTGATA 1590

*msg*18 AAAAATACCAAAAACCACGGATAACGCTCAAATAGAA---AAGAGTCATG 1497

* * * * * ** ** * *

*msg*49 GGCTTCGAATTGGAGGATTTCACAGAGCAGGAATCATTTGTCTTATCATG 1677

*msg*51 GACTTCGTGTTGAAAAGTTTCATGAAATGAGGGTAATTTGTTTAATTATA 1554

*msg*73 GTGTTCGAGTACAAGGGTTTGAAAGGGCAGGAATAATTTGTTTAATTATA 1646

*msg*39 GTGTCCGAATAGAAGGATTTCAAAGAGTAGGGGTAATTTGTCTGATTATA 1158

*msg*59 GCATCAAAAAAGAAGCCTTTCAAAAAGTAGGAATATTTTGTCTAATTATA 1640

*msg*18 GGGTTCGAATAGA---ATTTCAAAAAGTATTGGTATTTCTTTTAATTATA 1544

* * *** * * ** * * ** **

*msg*49 GGGATAACAGCAGGTGTTTGGATAATTGTT**TAA** 1710

*msg*51 GGAATAACAGCAGGAATTTGGATAATTGTT**TAA** 1587

*msg*73 GGAATAACTTCTGGAGTATGGATATTTGTT**TAA** 1679

*msg*39 GGAATCATGGCAGGGATGTGGATAATTGTT**TAA** 1191

*msg*59 GGGGTAACAACAGGGGTTTGGATAATTGTT**TAA** 1673

*msg*18 AGAGTGATTGCAA---------CA---ATG**TAA** 1565

* * * * * * **
