# Supplementary material for: Mechanisms of Surface Antigenic Variation in the Human Pathogenic Fungus Pneumocystis jirovecii
Source: mBio. 2017 Nov 7;8(6):e01470-17. doi: 10.1128/mBio.01470-17 (PMC5676039; doi:10.1128/mBio.01470-17)
Supplement: FIG S3 [file mbo005173568sf3.docx]

Fig. S3 (26 pages)

**Msg-I**

**<--CRJE--->**

Msg32 VGHSLARAVARAVKRRAQAQE--R-K--MR-KNCVFYFDCRNDYNNEPKC 44

Msg24 VRHSLARAVARAVKRRAAAAQ--KASVYED-EEILFALIAGKDYNDDSKC 47

Msg84 IWHGLARAVARAVKRRAAQGA--GTN-DEE-EVRLLALIAKENYKNTDQC 46

Msg56 VRHSLARAVARAVKRQVKGAQ--NSI---D-EEHVLALILKES-LEKTEC 43

Msg6 VRHSLARAVARAVKRRAAGAG--VNV-YED-EDYLLALILKEGAMDESQC 46

Msg94 LKHILARAVARAVKRQAKGAQ--NSI---D-EEHVLALILKENDLDETKC 44

Msg45 ---ILARAVARAVKRQAKGAQ--NSI---D-EEHVLALILKENDLDETKC 41

Msg41 VRHSLARAVARAVKRQVKGAQ--NSI---D-EEHVLALILKEDGLGEQEC 44

Msg103 VKHGLARAVARAVKRQAQKN---DEI---G-EEHVLALILNKNDLEKEEC 43

Msg93 HRHGLARAVARAVKRQTAGVK--NNE---A-EERLFALITKEDYSDTGKC 44

Msg78 VRHSLARAVARAVKRRAQAAQVVQSI-Y-DDDGILLALIAGKY-YKDDEC 47

***********:. . :: : : :*

**N-glycolsylation sites (NXS/T, X≠P)**

Msg32 KEKLKNTVKN**N-QS**KLKIEQIHEKLKDLCNNQ-NKR-INANIKKQSKMNA 91

Msg24 KKKLEEYCQGLE**NAS**LKKKEIHGKLESFCKNG-EAAKKCTELKGKVQGKC 96

Msg84 KDKVEKYCKTLTDAGLNPEKVHEKLKDFCNNG-KQNKKCQDLQTKVTGKC 95

Msg56 IKKLKEYCENLKKVDE**N-YS**VYSKLKEICKDN-GA-KKCTELKNNVDTKK 90

Msg6 KEKLKEYCEDIKRVDEN-FKVYTKLKEICKDN-GA-AKCTGLKAKIDTKK 93

Msg94 IKKLKEYCQELKEAKLTTEKVHEKLKDICDNT-KRDEKCKKLKTKVTEKC 93

Msg45 IKKLKEYCQELKEAKLTTEKVHEKLKDICDNT-KRDEKCKKLKTKVTEKC 90

Msg41 KEKLKKYCQELTEAKLNIEQVHKKLEGFCKDG-KADEKCKELKANIEKKC 93

Msg103 KKRLKEYCKGLKNINPKLDKVDQKLQKVCKEDRTAEEKCTGLKDKVTQKC 93

Msg93 KNKIKEYCYGLK**NAS**LTSEKVHKELKDFCKDG-NEEKKCGELKTKIEEKC 93

Msg78 KKELEKYCKALTDAELKPEKVHKKLKEFCENK-KADSKCKELKEKLTQKC 96

..::: . .: :*: .*.: :: : :

Msg32 THLKQR-RTLV----AKSIQDDECTKNERQCLFLEGACPTDLKEDCNT-R 135

Msg24 TTFKTK-LQTAAGKGISDLTDDECKENEQQCLFLEEACS-DLTENCNKLR 144

Msg84 TSFQQK-LKTA----LT**NPS**DDNCKENEQQCLFLEGACPKELKDDCNTLR 140

Msg56 TAFKAK-LDDASKKEISQLTETDCS-NQKECLFLEEAYSNDLKENCNTLR 138

Msg6 IAFKTK-LDEASAKQISQLKDIDCL-NQKECLFLEEAYSNELKEKCNTLR 141

Msg94 NEFKTKKLEQA----LKKLSDNDCKENERQCLFLEGACPSVLIEDCNKLR 139

Msg45 NEFKTKKLEQA----LKKLSDNDCKENERQCLFLEGACPSVLIEDCNKLR 136

Msg41 TTIKGK-LKEAIKKKNSDLTDKDCKENEQQCLFLEGVCSKELKDDCNTLR 142

Msg103 NDFKNK-LQIAAVKEISKLTDGDCKENERQCLFLEGACPSDLTENCSKLR 142

Msg93 KTFKGN-LETAVKKNIKNLEDTDCA-NEQQCLFFEGACPSDLTENCNKLR 141

Msg78 TAIKGK-LTEAIKKKKSDLTDEDCKKNEQQCLFLEGACPN-LVEDCNKLR 144

:: . . .. : :* *:::***:* . . * :.*.. *

Msg32 NKCYKKKREEVANEV-FRALSDDLKDTNTCKGKIKDICLALGQESDELMQ 184

Msg24 NNCYQKKRNEVAEKVLLRALSDDLKDAKTCKEKIKDVCRTLGQESNELMQ 194

Msg84 NKCYQKKRDKVAEEALLRALSNDLE**NET**KCKEKVKNVCFALAEESDELMQ 190

Msg56 TNCYQKKRDEVAEKALLRALKGSLEDKNKCKKKLEEICPLLGQESNELIQ 188

Msg6 TNCYQKKRDEVAEKALLRALKGSLENDDKCKEKLKVVCLELGQESNELMQ 191

Msg94 NLCYQKKRDRVAEEVLLRALRGNLT**NET**TCQENLKEVCPILGRESDELTS 189

Msg45 NLCYQKKRDRVAEEVLLRALRG**NLTNET**TCQENLKEVCPILGRESDELTS 186

Msg41 NKCYQKKRDKVAEEVLLRALRSDL**NGS**VICEKKLKEICPVLGGKVM-STN 191

Msg103 NLCYQRKRDRVAEDVLLRALRGDLGDNSECKKKIKDVCPKIGQESDELTM 192

Msg93 NLCYQRKRDGVAEEVLLRALRDDLG**NDT**ACKKKIENVCLKIGQESDELTM 191

Msg78 NLCYQRKRDGVAEEILLRALRGDLEEEAGCKKKIKNVCSKIGQESDELTM 194

. **::**: **:. :*** ..* *: ::: :* :. :

Msg32 KCFNTESLCTSLVALAKEKCTPLKTEIEKVLNPGGKLKKEGYSLLEKCYF 234

Msg24 RCFDTDSLCTSLVKTAEEKCKFLKKEIENVLKPNGELQKKGHSLLEKCYF 244

Msg84 KCFNTDSLCTSLVALAREKCESLKTMIKNVLTIDGELQKKGHSLLEKCYF 240

Msg56 KCLNLDSTCPPLVQAAKNKCD-FKTEIEKVISNKEELQKRGRFLLEECHF 237

Msg6 RCFNTDSTCTTLVKAAEEKCTPLKTEIENVLNPGGELKKEGHFLLEKCYF 241

Msg94 LCLNQENTCKNIIKKRDNKCNTLKTSVAAALGS--FKKEECLSLLEECYF 237

Msg45 LCLNQENTCKNIIKKRDNKCNTLKTSVAAALGS--FKKEECLSLLEECYF 234

Msg41 LCLNQKETCKNILIEKDKKCGTLKTDVSAALGSF--KKETCLELLEQCYF 239

Msg103 LCLDQEKTCVSLVAKGKSKCSALKQEVEEALKKKNELRGKCLPLLEQCYF 242

Msg93 LCLDQQATCVSLVAKGKSKCSVLKQKVEEALKEKNELRGKCLPLLEQCYF 241

Msg78 LCFDQKKTCKKFVSEREGKCDALEKIVKEALKKNSELRGKCLSLLEQCYF 244

*:: . * :: ** :: : .: : ***:*:*

Msg32 YGPSCKD-----------------NKCSDLKEKCKEEKIIYIPPGSDFDP 267

Msg24 YEENCKDP--------------NKPKCEKLVNEAKKKKIVYKGPSSDFDP 280

Msg84 YGPSCKG-----------------SKCGDLKEKCKKEEIIYLPPDSDFDP 273

Msg56 YGPNCKNK---------------ESKCNNLQKKCKEEEIFYVPPGSDFNP 272

Msg6 YGPNCKG-----------------SRCGDLKEKCKKEGIIYIPPGSDFDP 274

Msg94 YVGNCQED--------------DIIECIKLGEKCQEQNIVYIPPGPDFDP 273

Msg45 YVGNCKE---------------YISECDTLAEKCEKENIVYMHPGPDFDP 269

Msg41 YIGNCGDD--------------DIIKCIELGGKCQEQNIVYIPPGPDFDP 275

Msg103 HRGNCKKDASQCK-PQNKDCEEYLPKCDELAEECEERSVIYIHPGPDFDP 291

Msg93 HRGNCKKDASQCK-PQNKDCEDYLPKCDELAKECGKKGVIYIHPGPDFDP 290

Msg78 HRGNCEGDKSKCNKLTNQDCKEYIPDCNKLEEECEKQNIIYTHPGPDFDP 294

: .* * * :. :. :.* *..**:*

Msg32 TKPKTTVAEKIGLEELYKKAAAQGVLIGRILKEDIVDILVFLSENDD--- 314

Msg24 TRPEATLAEKIGLKELYEEAATQGVLIGRALKEDIVDILVFLSEDN---- 326

Msg84 TRPEATLAEKIGLEKLYKETVTQGVVIERAPERDIVDLLVFLSESKP--- 320

Msg56 TKPEPTLAEKIGLEELYKEAATQGILFGKPPERDVVDLLVFLSKSNL--- 319

Msg6 TRPEATLAEKIGLEELYEEAAAQGVLIGKALEGDVLDLLVFLSEKNS--- 321

Msg94 TRPEATLAEDIGLEELYKEAEKDGIFIGKNHLRDATALLALLVGKDNTG- 322

Msg45 TNPEPTIAEDIGLEEFYKKAEEDGVFIGRQQVRDATALLALLVKKDNTG- 318

Msg41 TRPEATIAEDIGLEELYKEAEKDGIFIGKNHLRDATALLVLLIKDSNS-- 323

Msg103 TKPEPTVAEDIGLEELYRRAEEDGVFIGKNHLRDATALLTLLTGKDS--- 338

Msg93 TKPEPTVAEDIGLEELYKRAEEDGVFIGKQHLRDATALLALFVEKNA--- 337

Msg78 TKPEPTLAEDIGLEELYKEAEKDGVHIGRPPIRDATALLALLIQNPILNT 344

*.*:.*:**.***:::*..: :*: : : * :*.:: .

Msg32 --FKKDKCVAVLTDKCNSIKYLAESIKELCKNTG-----NYTDKCKEFEE 357

Msg24 --FDKDKCKKVLTDKCDSIKYLAKSIKELCE**NTS**-----SHENQCEEFKK 369

Msg84 --FNEAQCKEVLTNKCDSIQYLAKDLKDLCKDKN-----**NYT**DKCKEFKN 363

Msg56 --FNENKCKDVLNTKCDSFKHLAKELKDLCENKN-----**NHT**DKCKEFEK 362

Msg6 --FDETQCQSVLSNKCKFIGYLTDDLKELCGNKT-----IHKDKCKEFKN 364

Msg94 RNNDGEKCNKILEDKCKNS-HEHEALEKLCNKT**NP-S**-DY-KKRNAKNEK 368

Msg45 NNDIGKKCNEILENKCKNS-HEHEALEKLCNKT**NP-S**-DYKKKKCEELEK 365

Msg41 KKDDKEKCKEALQKSCKNP-HEHEALESLCKKNGL-S-NDGTKKCEELQN 370

Msg103 --DMEKKCTKVLGEKCKNS-QQHEALEDLCNNKGI-PNTNGTEKCKELK- 383

Msg93 --NVETKCNQVLADKCKNS-HEHEALEKLCTKNAV-N-NIGKEKCKELEE 382

Msg78 QLSEKEKCGKVLKDKCKEL-KEHEVLGYLCDDSDSTS-Q**NGT**EKCNVLGE 392

:* * .*. . : ** .. .:

Msg32 EFKKK-RIHTAKLGNSQ------FEDEIALWDGLPTFLTENECTRLQSDC 400

Msg24 EFGRKKDALTKKLESKQ------FENKIELWSELPRFLTENDCTRFQSDC 413

Msg84 EFEEIKLALTAKFKIRQ------FKDEIALWDELPSLLTENDCTRLQSDC 407

Msg56 EFKGIKASLTAKFK--K------FGNEIELWNELPNFLTEDECAELESDC 404

Msg6 EFDKKKNALNTKFERNSF-----KSNEIKLWSELPNFLNEDECVELESDC 409

Msg94 DIRKTCDILTSKLTNNHLFDSKKGSNGIIGWGGLPTFLSNEDCAKLESYC 418

Msg45 DIRKTCDILTSKLTNNHLFDSKKGSNGIIGWGGLPTFLSNEDCAKLESYC 415

Msg41 DINKTCKIFTSKVTNNRLFDPTKGNNEIVGWEGLPTFLSNEECAKLESYC 420

Msg103 DVKERCKILTLKVINNRFFDPTKGNNEIVKWGKLPTFLSKEECTRLESYC 433

Msg93 DIKKTCNIFASKLINNRLFDPKKGNNGIIGWGGLPTFLSNEDCTKLESYC 432

Msg78 ELAKRSLIVSEKIKNKHL----SGSGKTIPWYKLSTFLSDSDCARLESDC 438

:. *. . * *. :*...:*..::* *

Msg32 FYFGGQ-TSLKKWCKNVKAACYKRGLDALANQALQDKMRGKFHDRNDPLF 449

Msg24 FYFKSQ-TSLDKWCKSVKAACYKRGLDALANQVLQDRMRGKFHDRNDKWS 462

Msg84 FYFENQ-QSFEKQCKNVKAACYKRGLYALANQALQDKLRGKFYDTNDPLF 456

Msg56 FYFEKQ--SFEKQCKNVKAACYKRGLYKLANQAFQDKLRGKFHDTNDPFF 452

Msg6 FYFE**NQ--S**FEKQCKNVKAACYKRGLYALANQVLQDKLRGKFHDTNDTSF 457

Msg94 FYFEKKCQDGEKSCANVRAACYKRGLDARANNILQKNMRGLLHGSNKDWL 468

Msg45 FYFEKKCQDGEKSCANVRAACYKRGLDARANNILQKNMRGLLHGSNKDWL 465

Msg41 FYFEKKCPDGENACKNIRATCYKRGLDARANKVLQENMRGMLHGS**NKS**WL 470

Msg103 FYFKESCPDAEKACMNVKAACYKRGLDARANKVLQENMRGLLRGS**NQS**WL 483

Msg93 FYFEKKCQDGEKSCANVRAACYKRGLDARANKVLQENMRGMLHGS**NKS**WL 482

Msg78 FYFAQNKDPLEKECKNVKAACYKKGLEALANEAFQSKMYGLFRGSGEKWF 488

*** . .: * .::*:***:** **: :*..: * : . ..

Msg32 KKLQKELVKECANLKGK------SDELFVLCVQPTNAVFILLADLHMKAD 493

Msg24 EMLQKELVKVCMDLKEK------SDELFALCVQPTEAIFTLLDDLYIKTD 506

Msg84 RKLQKELVKECANLKGE------SDELFVLCVQPTEAIFILLNDLRIKTE 500

Msg56 EKLQKELVKVCLGLKKK------SNELFVFCVQPDSAVSILLGDLHFKVD 496

Msg6 KKLQKKLVKVCVDLKKK------SNELFVFCVQPNNAVSILLRDLHFKLD 501

Msg94 KKFQQELVKVCEKLKGNK-GSFSNDELFVLCIQPAKAARLLTHDHQMRVI 517

Msg45 KKFQQELVKVCEKLKGNK-GSFSNDELFVLCIQPTKAARLLTHDHQMRVI 514

Msg41 EKFQQELVKVCEKLKKENKGSFS-TMNYLFCVYSQQKQPGCLHMIFERTI 519

Msg103 KEFQQRLVKVCKELKENK-GSFPNDEIFVLCVQPAKAARLLTHDHQMRVI 532

Msg93 EKFQQELVKVCKELKENK-ENFPNDELFILCVQPAKAARLITHDHQMRVI 531

Msg78 KGLLDKIMEECSGLKTTS------DELFLLCIDPLKAVRILAADIQTRAI 532

. : ..::: * ** : :*: . . :

Msg32 LLQKDLNKKRDFPTKWDCRELQKKCNDLKQDFEELEWPCHTLKHHCNRLE 543

Msg24 LLREDLNKKRDFPTKQDCKELQKKCNDLKQDFKGLEWPCHTLEYHCNRLN 556

Msg84 LLHEDLNEKRDFPTKQDCRELQKKCDNLKQDFKGLEWPCHTLEHHCNRLN 550

Msg56 LLQEYLNARRDLPTKRDCKILLKKCKDLVQDSEEIEWPCHTLKQNCDRLK 546

Msg6 LLQEYLNARRDLPTKRDCRILLKKCEDLKQDFEELEWPCRTLEHHCNRLD 551

Msg94 FLRQQLDKKRDFPTDKDCKELGRKCQDLGKDSKEITWPCHTLEQQCNRLG 567

Msg45 FLRQQLDKKRDFPTDKDCKELGRKCQDLGKDSKEITWPCHTLEQQCNRLG 564

Msg41 FLRQQLDQKRDFPTDKDCKELGRKCETLGKDSNQIQWPCHTLKQQCDRLG 569

Msg103 FLRQQLDQKRDFPTDKDCKELGKKCQDLGKDSKEITWPCHTLEQQCNRLG 582

Msg93 FLRQQLDQKRDFPTDKDCKELGRKCEALGKDSNQIQWPCHTLEQQCNRLG 581

Msg78 FLRKQLDQKRDFPTDKDCKELGRKCEALGKDSNQVQWPCHTLKQQCDRLG 582

:*:: *: :**:**. **: * :**. * :* : : ***:**: :*:**

Msg32 IVEQLEERLLEENVKDLKNETVCKKSVKEQCNHWVEKRKTQFALGCLAH**N** 593

Msg24 VVGQLEERLLEGKIKDLKNETVCKENVEEQCNHWVKKRKTQFALGCVAQ**N** 606

Msg84 VVGQLEERLLEGKIKDLKNKTVCKENVEEQCNHWVKKRKILFALGCVAQ**N** 600

Msg56 VVEQLEEKFLEEKMEKLDDSNSCIEKIGQRCREWNRRGRTHFDLACVTQ**N** 596

Msg6 IAEQLEEKFLEEKVKDLDKFDLCLKSLKEQCHEWNRRGRVQFALACVAQ**N** 601

Msg94 TTEILKQVLLDEHKDTLKTHENCVTYLKEKCNKWSRRAMT-VSLLYVFSK 616

Msg45 TTEILKQVLLNEHKDTLKTHENCVTYLKEKCNKWSRRGNNHFSLVCVFQ**N** 614

Msg41 LQ-KFKQVLLGEHKDTLKDQESCVKYLKEKCNKWSRRGDDRFSFVCVFQ**N** 618

Msg103 TTEILKQVLLDEHKDTLKDQESCVKYLKEKCNKWSRRGDDRFSFVCVFQ**N** 632

Msg93 TTEILKQVLLDEHKDTLKDEESCLKYLKEKCNKWSRRGDDRFSFVCVFQ**N** 631

Msg78 TTEILKQVLLDEHKDTLKDEESCVKYLKEKCNKWSRRGDDRFSFVCVFQ**N** 632

::: :* : . *. * : ::*..* .: . : : :

Msg32 **VT**CKIITESLVLKCSSLEKHIETLKVVEDAKKKTK----KKKLA----VF 635

Msg24 **VT**CKIITESLILKCSSLENHIETLKVVEDAKKEDQ----KEKTCSFWEPY 652

Msg84 **AT**CKIITESVDSKCIALKENMNTLKVVDRAEKKQE----RESTCNFWEPY 646

Msg56 **TS**CKILTESIGSKCTTLKARMKTSDVIKTAKEETT----MEETCDSWIPY 642

Msg6 **IT**CKILTESVNFKCITLGVRIEASNVINQAKSNNQ----KESTCNSWTPY 647

Msg94 TLRVSMVKDVQDRCKIFKENIKVSEIVDFLK**NNT**N**NIT**TLERNCPSWHTY 666

Msg45 **AT**CKLMVDDVKDRCEVFEKNIKASEIVGFLK**NNT**N**NIT**TLGNVCPFWDPY 664

Msg41 **AT**CELMVKDVQDRCKVFKENIQVSEIVEFLK**NNT**N**NIT**TLGNVCPFWDPY 668

Msg103 **AT**CELMVKDVKDRCEVFKKNIKASYIIEFLE**NNT**NKITTLERNCPSWHTY 682

Msg93 **AT**CELMVKDVQDRCKVFKENIENSKIIGFLK**NNT**DKITRLANVCPFWDPY 681

Msg78 **AT**CELMVKDVQDRCKVFKENIKASEIVDFLK**NNT**N**NIT**TLERNCPSWHTY 682

:...: :* : .:: :: :.: . :

Msg32 GNRIVTNSCLAVIIMNDGNK-VCKNLKRIASHTEKDITWKHKLCTSLEGS 684

Msg24 CDKF-MFSCDNLINDN--NK-VCKKLKKNCKPYRERYNLETHVMYEFRGK 698

Msg84 CDKF-MFSCDNLIANG--NG-ECEKLKKNCESYRERYNLETQVMYEFRGK 692

Msg56 CSKF-VSSCH**NLT**TSG--GG-ECEELNKECKSFIERKELEEKVIDELKGS 688

Msg6 CSKF-MSSCY**NLT**TAG--GG-KCEELNKECETFIKKKELELKLVDQLKGH 693

Msg94 CNRF-SSNCPDFSKK----N-PCTKIKNNCKPFYERKALEDALKVELRGK 710

Msg45 CDKF-SPNCLDFSKK----N-PCTKIKNNCKPFYERKALEDALKVELRGK 708

Msg41 CDKF-SPNCPDLTKK----NTLCTKIKDHCKPFYERKALEDALKVKLRGK 713

Msg103 CNRF-SPNCPGLTKE----N-SCTKIKKHCEPFYKRKALEDALKVELQGK 726

Msg93 CDKF-SPNCLDLLKK----D-TCTKIKKHCKPFYERKALEDSLKVELRGK 725

Msg78 CNRF-SPNCPDLTKE----D-SCTKVKKHCEPFYKRKALEDALKVELRGK 726

.:: .* . * ::: .. : : : .:.*

Msg32 LK--KSCRTTLDQYCTQWSKTK**NST**LESLCTDKNG-----N**NDT**IRDNLC 727

Msg24 LKEEVSCRTTLDQYCTQWSKTK**NKT**LESLC**NST**NG----VDNDKIRDDLC 744

Msg84 LKKKRTVEQLLINIVH--SGVRQKTVHWKVYVLTR----VAIMIQLGMIY 736

Msg56 LKTEQTCKETLNKYCTQWE**NAT**NQ-LSTLCTDKTNK-----NGNVKKKLC 732

Msg6 LNTKEKCKGELDKYCTQWA**NAS**NG-LETFCTNKKKK---SKQDDLRKELC 739

Msg94 LSDENKCTAALKGYCTLAGNVN**NAS**VRSLCKDNTQGSNKKTDEKVVEELC 760

Msg45 LSEKNGCATALERYCTVAGNAN**NAS**IRSLCKDNTKGNPK-KDDEVRKELC 757

Msg41 LSDENKCTTELKDI-VQSENVN**NAS**ISGLCKDSTQGNNKKPDDKVVEELC 762

Msg103 LTDKSKCEPALKRYCTVAGNVN**NAS**ISGLCKANTKDNSGKSDEDARKELC 776

Msg93 LNKKNECTTALEGYCTIAGNVN**NAS**IKSLCKDTTDNKSKKDDNKVREELC 775

Msg78 LSDKNKCTTELKGYCTIAKNVN**NAS**INGLCKDNTQGNNKKPDDKVVEELC 776

*. * . : : . :

Msg32 KKLLKRVKARCTELSTELNTAKK-RKTKVEEVGKLNKEAKKASEGAKLVL 776

Msg24 KKLVERVKTRCTELSTKLNTAKEGIKTKVEEVKKLNEGAKKASEGAKLVL 794

Msg84 VKNVKRVEARCAELFTKLKTAKEEIKIKVEEVKKLNKEAKKASEDAKLVL 786

Msg56 KKLVERVKKQCPVLKVKLTKASEELFKKKEEYKKLKAEAVNAMDDANLVL 782

Msg6 EKLVEQVKKRCPGLKKELTEASKELEKKANKYEDIKKKQKKQWKKQILFY 789

Msg94 KKLMEEVKEQCETLPAELKQPADDLEKDVKTYEELKEEAKKAM**NKS**SLVL 810

Msg45 EKLVKEVEEQCKALPTELGQPAADLKKDYKTYEELKKRAEEAM**NKS**SLVL 807

Msg41 KKLMEEIKEQCETLPAELTELEKSLEKDVKTYKELKKEAKKAM**NKS**NLVL 812

Msg103 EKLVKEVEEQCKALPTELGQPAADLKKDYKTYEELKKRAEEAM**NKS**SLVL 826

Msg93 EKLMEEVKEQCKTLPTELEQPEKDLQEDYKTYKELKKQAEEAM**NKS**NLVL 825

Msg78 KKLMEEIKEQCETLPAELTELEKSLEKDVKTYKELKKEAKKAM**NKS**NLVL 826

* ::.:: :* * :* . : .:: : . *.

Msg32 SSLKKNEVNAKN-TADNVAVNSGK**NAT**N-DQ-NKSVGGTNRTQKTQVKLV 823

Msg24 SSLKKNEVNAKN-TADNVAVDSGK**NAT**N-DQ-**NKS**VGGT**NRT**QKTQVKLV 841

Msg84 SSLKKNEVNSEK------------**NAS**N-NQ-NKPVGGA**NTT**QKTQVRLV 822

Msg56 SKAKVMDDKSAGKTAPSVPAPAAPSAPAPNT-PPTAPNGTQ-NTVLFKLV 830

Msg6 QKQKQRMTNLQVKQ-YHQNQHQH-----QHR-THHQQHQMEHKTQYYLNL 832

Msg94 SFVKKDGNNTPK-**NN-----------S**K-SEDKNVVSNE-KDTIKHVKIL 846

Msg45 SLIKK**NESNVS**K-SN-----------SK-NKDKNAVSNGFQDTTEHMKIL 844

Msg41 SLVKK**NESNTS**K-NNR--------**NNS**K-NKDKNAVSNGLQDTTEHVKIL 852

Msg103 SLIKK**NESNVS**K-SN-----------SK-NKDKNAVSNGLQDTTKHVKIL 863

Msg93 SLVKK**NESNAS**K-GN-----------SK-DK-KNAVSN-EQDTTKHAKIL 860

Msg78 SLVKK**NESNTS**K-NNR--------**NNS**K-NKDKNAVSNGFQDITEHVKIL 866

. * : :

Msg32 RRGT-----VEVLVTEAEVEAFDAVSRALEAYVEVNEECKVLKLECGFKE 868

Msg24 RRGT-----VEVLVTEAEVEAFDAVSRALEAYVEVNEECKVLKLECGFKE 886

Msg84 RRGT-----VEVLVTEAEVEAFDAVSRALEAYVEVNEECKVLKLECGFKE 867

Msg56 RRNINTPVTEKTYVTEKELKAFDLVSQAFSLYVELKELCHDSEKGCGFKE 880

Msg6 RRNINAPVTEKTYVTEKELKAFDLVSQAFSLYVELKKLCHDSEKGCGFKE 882

Msg94 RRGV-----KDVLVTELEAKAFDLAAEVFGRYVDLKERCEKLTLDCGIKD 891

Msg45 RRGV-----KDVLVTELEAKAFDLAAEVFGRYVDLKERCEKLESDCGIKE 889

Msg41 RREL-----RR-HLTESEAKAFDLAAEVFGRYVDLKERCEKLTSGCGIKD 896

Msg103 RRGV-----KDVSVTELEAKAFDLAAEVFGRYVDLKERCNKLESDCGIKE 908

Msg93 RREV-----KDVSVTESEVKAFDLTAEVLGRYIDLKEKCNKLTSDCGIKD 905

Msg78 RRGV-----KEALVTESEAKAFDLAADVFGRYVDLKERCEKLKSDCGIKD 911

** :** * :*** .: .: *::::: *. **:*:

**<----T-rich--->**

Msg32 ECSTLKDACSEIEKACNELKSLEVKPYEVETTTTTTTTTTTTTTTKTEGE 918

Msg24 ECPTLQDACSKIEKACNELKSLEVKPYEVETTTTTTTTTTTTTTTKTEGE 936

Msg84 ECPTLKNACSEIEKACNELKSLEVKPYEVETTTTTTTTTTTTTTTKTEGE 917

Msg56 ECEDIKEACTKIKKACDGLKPLEIKPHEIVTK**NVT**TTTTTTTTE--TVKD 928

Msg6 ECKDIKDACTKIEKACGGLKPLEIKPHEIETTTTTTTTTTTTTK--TEGE 930

Msg94 DCDGLKGVCGKIKKKCRDLKPLEVKSHEIVTESTTTTTT-TTTV----TD 936

Msg45 DCKDLKDVCGKIEKICRDLKPLEVKSHEIVTESTTTTTTTTTTV----TD 935

Msg41 DCKDLENVCKKIGKTCSDLKPLEVKSHEIVTESTTTTTTTTTTV----TD 942

Msg103 DCKDLEEVCKKINKACRNLKPLEVKPHEIVTESTTTTTTTTTTV----AD 954

Msg93 DCDALKSVCKKIQGVCSKLEPLKVKLHETVTKI**NIT**TVTETVKE--AEKT 953

Msg78 DCKDLENVCKKIEKTCSDLKPLEVKPHEIVTESTTTTTTTTTTV----TD 957

:* :: .* :* * *:.*::* :* * **.* *..

**<-------------------ST-rich--------------------**

Msg32 GKTAECQTLQTTDTWVTKH-QHIPAHLRPHPQSPQETLTSTRRCKPTKCT 967

Msg24 GKTVDCQTLQTTDTWVTKTSTHTSTSTTTSTVTSRITLTSTRRCKPTKCT 986

Msg84 GKATDCQSLQTTDTWVTKTSTHTSTSTTTSTVTSRITLTSTRRCKPTKCT 967

Msg56 AKATDCQSLQTTDTWVTKTSTHTSTSTTTSTVTSRITLTSTRRCKPTKCT 978

Msg6 GKTAECQSLQTTDTWVTKTSTHTSTSTTTSTVTSRITLTSTRRCKPTKCT 980

Msg94 PKATECKSLQTTDTWVTQTSTHTSTSTITSTITSKITLTSTRRCKPTKCT 986

Msg45 PKATECKSLQTTDTWVTQTSTHTSTSTITSTITSKITLTSTRRCKPTKCT 985

Msg41 PKATECKSLQTTDTWVTQTSTHTSTSTITSTITSKITLTSTRRCKPTKCT 992

Msg103 PKATECKSLQTTDTWVTQTSTHTSTSTITSTITSK-TLTSTRRCKPTKCT 1003

Msg93 GDSEKCKSLSTTDTWVTQTSTHTSTSTITSTITSKITLTSTRRCKPTKCT 1003

Msg78 PKATECKSLQTTDTWVTQTSTHTSTSTITSTITSKITLTSTRRCKPTKCT 1007

.: .*::*.*******: * .: . . :.: **************

**> <--------GPI-anchor signal------->**

Msg32 TGEEDDAGDVKPSEGLRMSGWSVM-RGVLLAMMISFMI 1004

Msg24 TG--DDPEDVKPSEGLRMNGWSIM-KGVLLAMMISFMI 1021

Msg84 TGEEDEAGEVKPSEGLRMSGWSVM-RGVLVAMMISFMI 1004

Msg56 TGEEDEAGDVKPSEGLRVSGWNVM-RGVLLAMMISFMI 1015

Msg6 TGEEDEAGDVKPSEGLRMSGWSVM-KGVLLAMMISFMI 1017

Msg94 TG--DNAEDVKPNEGLKMSGWSVM-RGVILAMMISIMI 1021

Msg45 TG--DDAEDVKPSEGLRVSGWNVI-KGVIVAMVISFMI 1020

Msg41 TG--DDAEDVKPSEGLKMSGWSVM-RGVILAMMISFMI 1027

Msg103 TG--DDAEDVKPSEGLKMSGWNVM-RGVIVAMVISFMI 1038

Msg93 TG--DDAEDVKPSKGLKMGGWSVM-RGVILAMMISFMI 1038

Msg78 TG--DDAEDVKPSR---LEGERVECDEGIVAMVISFMI 1040

**: *:. :***.. : * : ::**:**:**

**Msg-II**

**<--signal peptide--> N-glycolsylation sites (NXS/T, X≠P)**

Msg104 **M**KAFALASFLGIAYAFSKNIESIYKESL--NPLQNPPQ----NPPQNP-- 42

Msg76 **M**KAFVLANFLGITYAFSKNIDSIYKESL--NLLQNPSQ----NPPQNPSQ 44

Msg79 **M**KAFALAGFLGIAYAFSKNIESIYKESL--NPLQNPPQ----NPPQNP-- 42

Msg85 **M**KAFALVNFFGIAYAFSKNIESTYKESL--NLLQNSSQ----NP------ 38

Msg7 **M**KAFALASFLGTAYAFSKNIESIYKESL--NPLQNPSQ----NPSQNP-- 42

Msg25 **M**KAFVLANFLGITYAFSKNIESIYKEFL--NLLQNPLQNPLQNPLQNP-- 46

Msg37 **M**KAFILAVFFGIIRAFSENIEFIHKKSVSLNSLQ**NTS**F----NP------ 40

Msg68 **M**KAFALTSFLGIAYAFLKNIESTYKESL--NLLQNPSQ----KPSQNS-- 42

Msg62 **M**KTFIIAVFFGIIHAFLENIEFIHKRSVSLNSLQ**NTS**------------- 37

Msg20 **M**KALTISCFFTIACAFS-NIELIHKRSVLLNSL----------------- 32

Msg3 **M**KVFILTFFLGIVHASSKNIEFIHKKST-LDQLLD--------------- 34

**.: : *: * **: :*. : *

Msg104 --SQNPLLY--ITEGNIFALILK-NAMNTECKARLRKYCENLR**NMT**QMLK 87

Msg76 NSPQNSLSH--IIERNIFALILK-DAVNDGCQEKLKKYCEDLR**NMT**QMLK 91

Msg79 --SQNPLLY--ITEGNIFALILK-NAMNTECKARLRKYCENLR**NMT**QILE 87

Msg85 --SQNPLFR--ITEGNIFALILK-DTMNNECQEKLKKYCENLR**NMT**QMLE 83

Msg7 --SQNSLSY--IIKGNIFALILK-DAKDNQCQVKLKKYCKDLEEMTQILK 87

Msg25 --SQNPLSY--ITKENIFALILK-DATNNQCQIKLKKYCKDLEEKAQMLE 91

Msg37 -----------IVEERIFALILKE**NAT**NNQCKIKLKKYCENFKNTNKALS 79

Msg68 --PQNSLLH--VAEENIFALILK-DAVNNGCQEKLKKYCEDLKKVSQMLK 87

Msg62 ---FTPITYETFNEERISALILKENAINGECKIKLKEYCENFKNTNKILS 84

Msg20 ---**NTS**LDS--ISEERVFILILKKNTITDQCQVKLKEYCGNLKNMDLEPE 77

Msg3 ----RSLYS--ITKERIFALILKENAINNHCHEKLKEYCEDLEKIGLNLN 78

. : .: **** :: *: :*::** ::.: .

Msg104 SSFKALEELCQETKLDEKCNDLKENIMRRCAALERSLQDIQTKPSPSTFE 137

Msg76 SSFKALEELCQEMKLDEKCNDLKENIMERCTALERSLKDIWTKPSPSTFE 141

Msg79 SSFRVLKELCEEKKLDEKCDTLKKTITEKCTILKQSLKSIRSEPSISS-Y 136

Msg85 SSFKALEELCKKTELDKKCNDLKENIMGKCTILKQSLQGIRNKQSISS-Y 132

Msg7 SSFETLKELCQETKLDENCNDLKESITKRCTALEQSLKDIQNKPFTSFSD 137

Msg25 SSFNVLKELCQETELNKKCNDLEENIMERCTALERSLKDVLTKLSKTVLY 141

Msg37 GVFLELSELCEDTKLDGKCTHLKERLAKQYEALKMNLKDIETESSLNSLK 129

Msg68 SSFKALEELCQETELDEKCNDLKKTITERCTILKKSLQGIQNKPSVSSSD 137

Msg62 SVFPQLNKLCKDSDLNKKCTYLKEQLAGQYEVLKQNLKNIETKTSINNEQ 134

Msg20 NLHYVLKEQCKEMNIQKKCNDLKVMIEKTCTTHQTILENVLQELSLMNED 127

Msg3 NYHFKLNELCEKSKMSEKCINLGENIKKSCDILKHALTDAIKKPSATNED 128

. . *.: *:. .:. :* * : : * . :

Msg104 CLYQNECMRIEEACSIKVNEKCNYLRAFCREKRRDDLKTEFLLRALSGNL 187

Msg76 CLYQNECMRIEEACPIKVNKECNYLRTLCREKRRDGLKTEFLLRALSGNL 191

Msg79 CLYEKECMRIEEACPIEVNEECNYLRTSCRRERRDNLKTEFLLRVLSGNL 186

Msg85 CLYEKECMFVEEVCPAEVKEECNYLRTLCEEKKRDDLKTKFLLRILSGNL 182

Msg7 CVYQNECMFVEEACSIEVKKGCNYLRIFCREERRDDLKTKFLLRALSGNL 187

Msg25 CLYQSECMFVEEACSMEVKEKCNYLRIFCRGERRDDLKTKFLLRALSGNL 191

Msg37 CIMQSKCMFLEGVYPNELKKKCNYLRVLCKEEKQDSLTTEFLLRVFTNNL 179

Msg68 CLYQKECMLIEEACPTEVKERCNYLRAFCREKRRDDLKTKFLLRALSGSL 187

Msg62 CIMQSECMFLEEVYPNELKKECNYLRILCKEKKQNSLTIEFLLRVFTNNL 184

Msg20 CLLEDDCMFLEGVCSVEFRKKCSELRERCRIKKQDNIKTEILLRALRENL 177

Msg3 CEMETECMFWEGVSPEELTEKCNNLRIICRKRKRH**NLT**KKFLLRVFNENI 178

* : .** * . . :. : *. ** *. .::..:. ::*** : .:

Msg104 KTQEECEKIIDKKCLAFMGESDELMKFCLIPLNRCNELVKLMETKCNSLE 237

Msg76 KTEEDCEKIIDKKCLAFMGESDELMKFCLIPFNRCNELVKLIKGKCSDLE 241

Msg79 KTQEECEKIIDKKCLAFMGESDELMEFCLIPFNRCNELVKLIKEKCSDLE 236

Msg85 KTKEECEKIIDKKCLLFMRESDELMKFCLIPFNRCDELMKLMREKCISLQ 232

Msg7 KTKEECKKIIDKKCLAFMGESDELMKFCLIPLNRCDELMKSIRSKCSGLQ 237

Msg25 KTKEECEKIIDKKCLAFMEESDELMNFCLIPFNKCDELIKLMKRECSSLQ 241

Msg37 KTEGDCEKIIDKKCLIFMEESDKLMKFCLTPSNRCKDLIKLMEKKCNNLE 229

Msg68 KTKEECEKIIDKKCLLFMGESDELMKFCLTPLNRCEDLIKLMEEKCSSLQ 237

Msg62 RNEEDCEKIIDKKCLIFMEESDKLMKFCLTPLNRCKDLIKSIESKCSDLK 234

Msg20 KSKEACEKVINEKCFLLMRESDELMSFCLTELDKCGKLVDAMRKQCSRLN 227

Msg3 KTVETCKQVINEKCHVFMKESDELMQFCLSPLNRCRDLIHFMKDEYTELK 228

:. *:::*::** :* ***:**.*** ::* .*:. :. : *:

Msg104 FEAKLFIEKTKISKEECISLFEKCYFHKSNC**NNT**LDNKCREIEKKCENEV 287

Msg76 FKTKIFIQSNKISKEECTSLFEECNFHESDCDG-IQNMCKEIKKRCENKV 290

Msg79 FKTKIFIENNKISKEECTSLFEECNFHESDCDG-IQNMCKEIKKRCENKV 285

Msg85 FHLKKFPGNTEISKEECNLLLEECYFHNSNC**NNT**LNDKCTEIEKTCEKEI 282

Msg7 FRLEKFTENTKISKEECNLLLEECYFHKSNCNS-IQNMCKEIEKNCENKV 286

Msg25 LSLKKLLGNTKISKEECNLLLEECYFHNSNC**NNT**LNDECREIEKKCENEV 291

Msg37 LEAQIFIENTKISKEKCNSLFEECYFHKSNC**NNT**LNNKCKEIEKKCENEV 279

Msg68 LNVKEFPGNTKISKEKCNLLLEECYFHNSNCNNALNDKCREIERTCEKEV 287

Msg62 FNINLFTQDNKISKEKCNLLLDECYFHKSNCDNTLHDKCREIEKMCEKEV 284

Msg20 RDIESLLYDFNVYKTQCDLLLEKCYFYILNCD--IQNMCKKLKEKCG-KI 274

Msg3 INIEQFFKNPKISEEKCNFLLDECYFYGSNYNNNLQDICKELEKKCKKKV 278

: : . :: : :* *:::* *: : : :.: * :::. * ::

Msg104 EYKYFSLPFNPVGKEIILIEKVGKEKIFKDEVGKPGIKDTIDLLVLLSNN 337

Msg76 KYEDFSLPFNPIGKEIVLIEKVGKEKLFKDEVGKPGIKDTIDLLVLLSNN 340

Msg79 KYEDFSLPFNPIGKEIILIEKVGKEKLFKDEVGKPGIRDTIDLLVLLSNN 335

Msg85 KYKHSSLPFNPIGKEIILIEKVGKEKIFKDEIRKPGIKDTIDLLVLLSNN 332

Msg7 EYKHFSLSFNPIGKEIILIEKVQKEKIFKDEVGKPGIKDTIDLLVLLSNN 336

Msg25 KYKHFPLPFNPIGKEIVLIEKIGKEKLFKDEIGKPGIKDTIDLLILLSNG 341

Msg37 EYKHFSLPFNPIGKEIVLIEKVGKEKLFKDEIGKPGIKDTIDLLVLLSNN 329

Msg68 KYRHSSLPFNPIGKEIILIEKVGKEKLFKDEIGKPGIKDTIDLLVLLSNN 337

Msg62 KYTPPNLIFNPIEKKITLIEKVRKEKLFGDEIRKSGIKDTIDLLVLLVDN 334

Msg20 EYISLDLTFDPLGKNLALIEKLGNLELFGGEFKRPEIKDTVDLLIMLTND 324

Msg3 KYIPPSSTFNPIGKDFTLMEKVKKKKLFRDEIGKPRMKDMIDLLVLMVNN 328

:* *:*: *.: *:**: : ::* .*. :. ::* :***::: :.

Msg104 YLDDCKNYIEKCHKLCSLLPQLEDLYDSAKEKM**NKS**KEEVCDTLKEKLKP 387

Msg76 YLDSCETNIKSCHKLCSLLPQLEDLYNGTKEKM**NKS**K-EICNTLEKELKS 389

Msg79 YLDHCKTNIESCHKLCSLLPELEDLYDNTKKKMDESKKEICNTLKEKFKP 385

Msg85 YLDNCKTDIESCHKLCSLLPELEDLYDNTKKKM**NES**KKEICNALGEKFKP 382

Msg7 YLDGCETNIEKCHQLCSLLPQLEDLYDNTKKKMDESKKEICNTLKEELKP 386

Msg25 YLSDCETYIEECRKLCSLLPQLEDLY**NNT**KRKM**NKS**K-EICTTLKKKLEP 390

Msg37 YLYGCKTNIESCHKFCSLLPQLEDLYDSTKEKMDKSKEEICNTLEEKLKP 379

Msg68 YLDDCKTNIERCHKLCSLLPQLKDLYDGTKEKM**NKS**K-KICNTLKEELKS 386

Msg62 YLSGCKENLKMCHKFCSTLPQLKDLY**NST**KKKMDKSK-EICDTLEEKLKP 383

Msg20 DITKCKDRVEGCYKFCSFLPQLKDLYN**NIT**KKMSK-E-EMCTSLKDKLRT 372

Msg3 DLEDCKTYVERCYEFCSSLPQLKNLYDNTKKKISENKEEICTNLKDTLKP 378

: *: :: * ::** **:*::**:. ..*:.: : ::* * . :..

Msg104 KCRFFKSKLYDLSLSNTKKDNEDATLIKWTKQSTEFNEKLCINLESKCFY 437

Msg76 KCRFFKSELHNLLLSNTSEDNEDSMLIEWTRQSTEFNEELCIDLESKCFY 439

Msg79 KCRFLKSKLYDLSLSNTSEDNEDSMLIEWTRQSTEFNEKLCIDLESKCFY 435

Msg85 KCKLLKSKLYDLSLSDTK-DDKDAMLMEWTKQSINFNEKLCIDLGSKCFY 431

Msg7 KCRSFKSKLYDLSLSDTK-DDKDATLIKWTKQSTEFNEKLCINLESKCFY 435

Msg25 RCKFFKSKLYDLSLSDTNEDNKDAKLIGWTKQSINFNEKLCIDLESKCFY 440

Msg37 KCRFFKSKLY**NLS**LSNTKEDNEDATLIEWTKQSTKFNEKLCIDLESKCFY 429

Msg68 KCKFFKSKLYDLSLSNTK-DNEDAALIEWTRQSIEFNEKLCIDLESKCFY 435

Msg62 RCRNFKSKLHNLLLSDTNDDNKDAKLLEWNKQFAEVDEKLCANLESKCFY 433

Msg20 KCKAFKLKLTSMSLS**NTS**KDDEEVELLGSFEQFPELNKRLCTNLESKCFY 422

Msg3 RCKALKLELYSLSLSDTTDDNKETTLLKWTEQFTEFDEELCTELESKCFY 428

:*: :* :* .: **:*. *::: *: .* :.::.** :* *****

Msg104 LRKSCNNAGIKMSNACINVESTCLKTRLFRREYQLFQSTLKGKLH**NLT**-**N** 486

Msg76 LKKPCSDEGIKMSNTCVNVESTCLKTRLFRREYQLFQGTLKGKLH**NLT**-K 488

Msg79 LKKPCSDEGIKMSNACVNVESTCLKTRLFRREYQLFQSTLKGKLH**NLT**-**N** 484

Msg85 LKKPCNDKDIKMSNACVNLESTCLKTRLFRKEYQLFQGKLRGKLH**NLT**-**N** 480

Msg7 LRKSCNDADIKMSNACINVESTCLKTRLFRREYQLFQSTLKGKLH**NLT**-**N** 484

Msg25 LRKSCNNLGIKMFNACINVKSTCLKTRLFRREYQLFQSTLKGKLH**NLT**-**N** 489

Msg37 LKKPCNDAGIKMFDACINLESTCLKTRLFKKEYQLFQGVLKGKLHDLE-N 478

Msg68 LKKPCGDEGIKMSNACINLESTCLKTRLFRKEYQVFQGILKGKLH**NLT**-K 484

Msg62 LERPCNSINIKMTNACINVRSTCLKALLFRREYRLFQDTLKGKLHNLI-V 482

Msg20 FQKPCTSENINLNGACSNVKLACLKMRAFKEEYYLFENNLRGRLH**NLT**-I 471

Msg3 YRRPCNLENIKLNNACGNVNSACLKTRLLKREYQLLQNKLTGKLHNLMIN 478

.:.* .*:: .:* *:. :*** ::.** :::. * *:**:*

Msg104 **-NS**LKTCIDELWILCEKIISSNNPILIDLCLHPWDTCKELANDIERQSRW 535

Msg76 -NSLKTCIDELWTLCEKIISSNNPILIDLCLHPWDTCKELANDIERQSRW 537

Msg79 **-NS**LKTCMDELWTLCKKIISNNNPVLMNLCLHPWDTCKELANDIERQSKW 533

Msg85 **-NS**LKTCIDELWILCKEIINNDNAILMGLCLQPWVTCKELADDIERQSKW 529

Msg7 **-NS**LKTCIDELWILCKEIINNDNAILMGLCLQPWVTCKELADDIERQSKW 533

Msg25 **-NS**LKTCIDELWTLCKKIISNDNPILMDLCLHPWDTCKELANDIERQSKW 538

Msg37 -NLLEICVNELWILCKKIINNNNPILMDLCLHPWDTCKELANDIERQSKR 527

Msg68 -NSLKMCIDELWTLCKKIINNDNAILMNLCLHPWDTCKELANNIERQSKW 533

Msg62 NSVLKRCIDELLDLCKKIIDKNNIISMSFCLQPQDTCLALANDIERLNHE 532

Msg20 SSSLKTCVNELLNLCRKGTGIKEPIFADLCLQPWNACLILANDIEKLSRE 521

Msg3 -DALKTCVNELLNLCKENVDIKEPLLADLCLHPKNTCQMLAKDIEKQSQE 527

. *: *::** **.: . .: : .:**:* :* **.:**: .:

Msg104 LRNDLDWKRDFPDEEDCKKLKEKCEVLGHDSKTNDLPCFTLKGRCDHLEN 585

Msg76 LRNDLDWKRDFPDEEDCKKLKEKCEVLGHDSKTNDLPCFTLKGRCDHLEN 587

Msg79 LRNDLDRKRDFPDEEDCKKLKEKCEVLGHDSKTNDLPCFTLKGRCDHLEN 583

Msg85 LRNDLDRKRDFPDEEDCKKLKEKCEVLGHDSKMNDLPCFTLKERCDHLEN 579

Msg7 LRNDLDRKRDFPDEEDCKKLKEKCQVLGHDSKTNDLPCFTLKERCDHLKN 583

Msg25 LRNDLDRKRDFPDEEDCKKLKEKCEVLGHDSKMNDLPCFTLKERCDHLKN 588

Msg37 LRNDLDWKRDFPDEEDCKKLKEKCEMLGHDSKMNDLPCFTLKGRCDHLKN 577

Msg68 LRNDLDQKRDFPDEEDCKKLKEKCEVLGHDSKMNDLPCLTLKERCGHLKS 583

Msg62 FRKDLNWKRDSPNEEDCKILEEKCRTLGQDSKINELPCLTLKERCDHLKS 582

Msg20 FRKDLNQKRDFPNEEDCRNLEENCKILGQDSKMNELPCLTLKERCDHLKN 571

Msg3 LNKNLNKKKDSINEEDCMELEEKCKILGQDSRINEFLCLELNKKCDHLKN 577

:.::*: *:* :**** *:*:*. **:**: *:: *: *: :*.**:.

Msg104 AKELEEILLEEKVENLGNLDICIKKVSEKCNKWSKKKRTRFIFSCIQLVT 635

Msg76 AKELEEILLEEKVENLGNLDTCIKKVSEKCNKWSKKKRTRFIFSCIQLVT 637

Msg79 AKELEEILLEEKVENLGNLDTCIKKVSEKCNKWSKKKRTRFIFSCIQLVT 633

Msg85 AKELEEILLEEKVENLGNLDICIKKVSEKCNKWSKKKRTRFIFSCIQLVT 629

Msg7 AKELEDILLEEKAENLGNLDICIERVSEKCNKWSKKKRTRFIFSCIQLVT 633

Msg25 AKELEDILLEEKAENLGNLDICIERVSEKCNRWSKRKKTRFIFSCIQLVT 638

Msg37 AKELEDILLEEKAENLGDLNTCIKRVSEKCNKWSKKKKTKFIISCIQLNA 627

Msg68 AKELEEILLKENVEKLDDLDICIKKVSEKCNKWSKRKRSEFILSCIQLNA 633

Msg62 AKELEEILLKENVEKLDDLDICIKRVSEKCNKWSKRKRSEFILSCIQLNA 632

Msg20 AKKLEEILLEEKAENLGNLDICIKKVTERCNNWSKRKRTEFILSCIQLNV 621

Msg3 TKELEEILIKEKTEKLYDLHTCIKKMTERCNNWPKKTKTLFTISCIQVNI 627

:*:**:**::*:.*:* :*. **::::*:**.*.*:.:: * :****:

Msg104 TCQIITRDIKSKCSVLERNIDIEDVLDQVKSNNADIKGPTCDLWEPYCDK 685

Msg76 TCQIITRDIKSKCSVLERNIDIEDVLDQVKSNNADIKGPTCDLWEPYCDK 687

Msg79 TCQIITRDIKSKCSILERNIDIEDVLDQMKNDNADIKGSTCDLWEPYCDK 683

Msg85 TCQIITRDIKSKCSVLERNIDIEDVLDQVKSNNADIKGPTCDLWEPYCDK 679

Msg7 TCQIIIRDIKSKCSILERNIDIEDVLDQVKNDNADIKGPTCDLWEPYCNK 683

Msg25 TCQIITRDIKSKCSVLEKNIDIEDVLNQVKSDNADIKGSTCDLWEPYCDR 688

Msg37 TCRIIIESIKFKCTALGKNIEIENVLDQVKNNDVNMKSPICDLWEPYCDK 677

Msg68 TCRIIIKSIKSKCTILERNIDIEDVLDQVKN**NDT**NIKGEACDFWEPYCDK 683

Msg62 TCRIIIESIKSKCTILERNIDIEDVLDQVKN**NDT**NIKGEACDFWEPYCDK 682

Msg20 TCQIIIESIRSKCTALEKNIEIENVLDQVKNDDVNIKGQTCDFWEPYCDK 671

Msg3 TCRIIIEDVKFKCNTLRKNMNIMKISEKIKTKDINIKKQICDFWEPYCDK 677

**:** ..:: **. * :*::* .: :::*..: ::* **:*****::

Msg104 FMLSCEKLVRNNGKNGKCKELKESCKSYRKTQEQEMKLMYELRGSLNSEN 735

Msg76 FMLSCEKLVRNNGKNGKCKELKESCKSYREIQEQEVRLIYELRGSLNDKN 737

Msg79 FMLSCEILVQNNGKNGKCKELKESCEPYRRIQEQEMKLMYELRGGLNSEN 733

Msg85 FMLSCEILVQNNGKNGKCKELKESCKSYREIQEQEVRLMYELRGSLNSEN 729

Msg7 FMLSCEILVQNNGKNGKCKELKESCEPYRRIQEQEMKLMYELRGGLNSEN 733

Msg25 FMLSCEKLVRNNGKNGKCKELKESCKSYRKTQEQEMKLMYELRGSLNSEN 738

Msg37 FTLSCEKLAQNNGKNGKCKELKESCKSYREIQEQEVRLIYELRGSLNSEN 727

Msg68 LLLNCEKLVKDNGNDGKCIKLKKSCILYRTEENQKMELMYELRGSLDNKN 733

Msg62 LLLNCEKLVQDNGNDGKCIKLKKSCILYRTEENQKMELMYELRGSLDNKN 732

Msg20 FQLNCEKLVQDNENDGKCIELKKSCKLHRMGENQKMELMYELRGSLDNKN 721

Msg3 FMLSCKGLIQ**NDS**KDGKCEELKENCKSYQE-------------------- 707

: *.*: * ::: ::*** :**:.* ::

Msg104 KCESTLNKHCLHWNKTK**NNT**FNNFCN**NNT**DIKN**NIT**KNELCRKLLKRLKE 785

Msg76 KCESTLNKHCLHWDKTK**NDT**FKNFCN**NNT**DTKN**NTT**KNELCKKLLKHVKE 787

Msg79 KCKSTLNEHCLHWDKTK**NDT**FKNFCN**NNT**DTKN**NTT**KNELCKKLLKHVKE 783

Msg85 KCESTLNKRCLHWDKTK**NDT**FKNFCN**NNT**DTKN**NTT**KNELCKKLLKHVKE 779

Msg7 KCKSTLNEHCLHWDKTK**NDT**FKNFCN**NNT**DTKN**NTT**KNELCKKLLKYVKE 783

Msg25 KCESTLNKHCLHWNKTK**NNT**FNNFCN**NNT**DIKN**NIT**KNELCRKLLKRLKE 788

Msg37 KCKSTFNEHCLHWDKTK**NDT**FKNFCNNNIDTKN**NTT**KSELCKKLLERVKE 777

Msg68 KCKTSLDRFCLFWDETK**NNT**FKNLCHDD**NGT**K**NDT**ARDELCVKLVDKMKK 783

Msg62 KCKTSLDRFCLFWDETK**NNT**FKNLCHDD**NGT**R**NDT**ARDELCVKLVDKMKK 782

Msg20 KCKTSLDRFCLFWDETK**NNT**FKNFCHDD**NGT**K**NDT**ARDELCVKLVDKMKK 771

Msg3 -------------------------------------------------- 707

Msg104 RCTKLFTKLNDTAVEIEKNVKIVEELNEAAKKALKNTNLILTSSKQKTDP 835

Msg76 RCTKLLTKLNDMATEIEKSIKIVEELNEAAKKALKNTNLILTSSKQKTDP 837

Msg79 RCTKLLAKLNGMATEIEENVKIVEKLNEAAKKALKNTNLILTSSKQKTDS 833

Msg85 RCTKLLAKLNGMATEIEENVKIVEKLNEAAKKALKNTNLILTSSKQKTDP 829

Msg7 RCTKLLAKLNGMAREIEENVKIVEKLNEAAKKALKNTNLILTSSKQKTDS 833

Msg25 RCTKLFTKL**NDT**AVEIEKSVKIVEELNEAAKKALKNTNLILTSSKQKTDP 838

Msg37 RCTKLLTKLNDMATEIEKNIKIVEELNEAAKKALKNINLILTSSKQKTGS 827

Msg68 KCTEMFIRL**NDS**ATEIEKSLDIAGKLIQTAKKALRSVKLTLISNRRRTCL 833

Msg62 KCTEMFIRL**NDS**ATEIEKSMDIAGELIQTAKKALRSVKLTLISNRRRTCL 832

Msg20 KCTEMFIRL**NDS**AIEIEKSLDIAGKLIQTAKKALRSVKLTLISNRRRTCL 821

Msg3 -------------------------------------------------- 707

Msg104 NIN**NAT**LILAYNANADRNVNLKTDVTLK**NQSR**YLEQKETKV**NIT**EKEVEA 885

Msg76 NIN**NAT**LILAYNANADRNVNLKTDVTLK**NQSR**YLEQKETKV**NIT**KKEVEA 887

Msg79 NIN**NAT**LILAYNANADRNVNLKTDATQK**NQSR**YLEQKETKV**NIT**EKEVEA 883

Msg85 NIN**NAT**LILAYNANADRNVNLKTDVTLK**NQSR**YLEQKETKV**NIT**EKEVEA 879

Msg7 NIN**NAT**LILAYNANADRNVNLKTDVTLK**NQSR**YLEQKETKV**NIT**EKEVEA 883

Msg25 NIN**NAT**LILAYNANADRNVNLKTDVTQK**NQSR**YLEQKETKV**NIT**EKEVEV 888

Msg37 NI**NNT**ILILAYNANADRNVNLKTDVTQK**NQSK**YLEQKETKV**NIT**EKEVEA 877

Msg68 NIHN--------WNTKTNANLKRDTT-QNQLEYLEQKEIEV**NIT**EKEVEA 874

Msg62 NIHN--------WNTKTNANLKRDTT-QNQLEYLEQKEIEV**NIT**EKEVEA 873

Msg20 NIHN--------WNTKTNANLKRDTT-QNQLEYLEQKEIEV**NIT**EKEVEA 862

Msg3 -------------------------------------------------- 707

Msg104 FDAAAEALKVYTEVKAECKGLQLECEFKEDCSEYKDVCKKIEDACNKLKS 935

Msg76 FDAAAEALKVYTEVKAECKGLQLECEFKEDCSEYKNVCKKIEDACNKLKS 937

Msg79 FDAAAEALKVYTEVKAECKGLQLECEFKEDCSEYKDVCKKIEDACNKLKS 933

Msg85 FDAAAEALKVYTEVKAECKGLQLECEFKEDCSEYKDVCKKIEDACNKLKS 929

Msg7 FDAAAEALKVYTEVKAECKGLQLECEFKEDCSEYKDVCKKIEDACNKLKS 933

Msg25 FDTAAEALKVYTEVKAECKGLQLECEFKEDCSEYKNVCKKIEDACNKLKS 938

Msg37 FNAAAEALKVYTEVKAECKGLQLECEFKEDCSEYKDVCKKIEDACNKLKS 927

Msg68 FDAATEALKVYREVEEECRSLLLKCKFKEDCSEHKNTCKKIEETCSKLGS 924

Msg62 FDAATEALKVYREVEAECRSLLLKCKFKEDCSEHKNTCKKIEETCSKLGS 923

Msg20 FDAATEALKVYREVEEECRSLLLKCKFKEDCSEHKNTCKKIEETCSKLGS 912

Msg3 -------------------------------------------------- 707

**<---------------------------T-rich------------**

Msg104 LEIKSSETKTI**NQT**IKTTII------------------------------ 955

Msg76 LEIKSSETKTI**NQT**IKT-II------------------------------ 956

Msg79 LEIKSSETKTI**NQT**IKT-II------------------------------ 952

Msg85 LEIKSSETKTI**NQT**IKT-II------------------------------ 948

Msg7 LEIKSSETKTI**NQT**IKT-II------------------------------ 952

Msg25 LEIKSSETKTI**NQT**IKT-II------------------------------ 957

Msg37 LEIKSLETKTI**NQT**IKT-IV------------------------------ 946

Msg68 LGVRSSETKITTETK---IT------------------------------ 941

Msg62 LGVRSSETKITAETK---IT------------------------------ 940

Msg20 LGVRSSETKITTETDK**N-TT**QETDK**NTT**QETETDK**NTT**QETDKNTIQETE 961

Msg3 -------------------------------------------------- 707

**---------------><-----ST-rich---------------------**

Msg104 ----------------AETET**NTT**QKTLTTGEQCMSLSTTDKWITRTSTH 989

Msg76 ----------------TKTET**NTT**QKTLMT-EQCMSISTTDKWITRTSTH 989

Msg79 ----------------TKTET**NTT**QKTLTTGEQCMSISTTDKWITRTSTH 986

Msg85 ----------------TKTET**NTT**QKTLTTGEQCMSISTTDKWITRTSTH 982

Msg7 ----------------TKTET**NTT**QKTLTTGEQCMSISTTDKWITRTSTH 986

Msg25 ----------------TKTET**NTT**QKTLMT-EQCMSISTTDKWITRTSTH 990

Msg37 ----------------TKTET**NTT**QKTLTTGEQCISISTTDKWITRTSTH 980

Msg68 ----------------TETDK**NTT**QETATTGEQCTSIP-RDKWITRTLTH 974

Msg62 ----------------TETDK**NTT**QETATTGEQCTSIP-RDKWITRTLTH 973

Msg20 TDK**NTT**QETDKNTIQETETDK**NTT**QETATTGEQCTSIP-RDKWITRTSTH 1010

Msg3 -------------------------------------------------- 707

**-------------------------------> <-------GPI-anchor**

Msg104 THTSTHTSVLTLTVTLTSTKGCKPVKCTIGSGEEAGDVKPSKGLRMNGWS 1039

Msg76 THTSTQTSVLTLTVTLTSTKECKPMKCTTGVER-KQEMKSSKGLRMNGWS 1038

Msg79 THTSTQTSVLTLTVTLTSTKECKPMKCTTGSEEEAGDVKSSKGLRMNGWS 1036

Msg85 THTSTQTSVLTLTVTLTSTKECKPMKCTTGSEEEAGDVKSSKGLRMNGWG 1032

Msg7 THTSTQTSVLTLTVTLTSTKGCKPVKCTIGSGEEAEDVKPSKGLRMNGWG 1036

Msg25 THTSTQTPVITLTVTLTSTKGCQPAKCTTGSGDEAGDVKSSKGLRMNGWG 1040

Msg37 THTSTQTSVLTLTVTLTSTKGCQPVKCTTGSEDETRDVKQNEGLKMNGWG 1030

Msg68 TYIPTEISIETLTVTLTSTQECQPVRCTTGSEDETGDVKQNDGLKMDGWG 1024

Msg62 TYIPTEISIETLTVTLTSTQECQPVRCTTGSEDETGDVKQNEGLKMNGWG 1023

Msg20 THTSTQTSVLTLTVTLTSTKGCKPVKCTTGSGDETGDVKPSKGLKMNGWS 1060

Msg3 ---------------------------------------PSEGLRMNGWS 718

..**:*:**.

**signal-------->**

Msg104 LIKRVILIMIISTMI 1054

Msg76 LIKRVILIMIISTTI 1053

Msg79 LIKRVILIMIISTMI 1051

Msg85 LIKGVILTMIISTMI 1047

Msg7 LIKGVILTMIISTMI 1051

Msg25 LIKGVILTMIISTMI 1055

Msg37 LIKGVMLTMIISTMI 1045

Msg68 LMKGVILTMIISTMI 1039

Msg62 LMKGVILTMIISTMI 1038

Msg20 LIKGVILTIIISTMI 1075

Msg3 IIKETILGVIISLVI 733

::* .:* :*** *

**Msg-III**

**<-signal peptide->**

Msg53 **M**KTFALASFLGIAYAFSKNIEFIHKGSASNPSQDPSQNP---------LD 41

Msg46 **M**KAFVLASFLGIAYAFSKNIEFIHKRSASDPSQDPSQDPSQDPSQDPSLN 50

Msg107 **M**KTFVLASFLGIAYAFSKNIEFVHERSASDPSQDPP------------LN 38

Msg34 **M**KAFVLASILGIAYAFSKNIEFVHKKSASDPSQNPLQNP---------LD 41

Msg110 **M**KVFALASFLGIAYAFSKNIEFIHKKSTSNLSQNPSQNL---------LD 41

Msg8 **M**KRFTLFCFLRIAYTFSKNNEPT---------------L---------LD 26

Msg55 **M**KAFALASFLGIVYAFSRNIEFIHKRSDSDPSQNPSQNPSQNLSQ-NSLD 49

** *.* .:* *.*:**:* * *:

Msg53 LPSDLSLDSLSEEKVLSLILGKNP-EFQCKKKLKEYCSDLKGLALEPKNV 90

Msg46 LLLDSSLDSLGEEKVLALILGKNAIDNECEEKLKKYCNDLEGQALKPRNV 100

Msg107 LLLDLSLDSLGEEKVLSLILGKNAIESQCEEKLQKYCSSLKSMALEPKNV 88

Msg34 LSFDLPLSSLGEEKILTLILGKNAINAQCEKKLEEYCKNLKNTSLVPEDV 91

Msg110 LSFDLSLDSLGEEKILTLILGKDAISAQCKKKLEEYCSNLKNSNLVPKNL 91

Msg8 TSLD-SLDLIGKDKVLTLLLGKEAISDQCVPKLQEYCKNLKNMALEPKNV 75

Msg55 LSFDLSLNSLSEEKIITLILGKDAIESQCEEKLQKYCNNLKSMTLEPKNV 99

* .*. :.::*:::*:***:. . :* **::**..*:. * *.::

**N-glycolsylation sites (NXS/T, X≠P)**

Msg53 HPTLEGLCKN--TEKKCNDLETQIDTLSSNAKDYLQDIIASSSG-NMVLG 137

Msg46 HPALKGLCEN--AKQKCTYLKTKINAVSNNVKTHLIDIMTNIADKKAHLR 148

Msg107 HPVLEELCKEGKAGQKCTNLKNGIISISNGIKNHLLGIMT**NIS**S-NVLLE 137

Msg34 HPALKGLCEN--AKQKCTNLNK-IISTSNNIKDHLQDILK**NIS**NELLLLE 138

Msg110 HPALKELCGN--AEKKCNDLKTQINILM**NNT**NESLLDIAL**NIS**TEGFHLE 139

Msg8 HPALGGLCGN--EDEECNNYKNRI**NTT**LTGIKESLMRIYEKLNTDGSTLD 123

Msg55 HPTLEGLCKN--AKQKCKNLKTQIDTFIYNTENSLLYIYAELVDGNTDLY 147

**.* ** : ::*. :. * . : * * . *

Msg53 KTRCNYAHIQCMIFREFFGFSSICDEIAEQCYRQINEDLAYEVLLRALPR 187

Msg46 KKYCNYAHIQCMFFREISSFSSICDEIAEQCYRQINEDSAYKVLLRALPR 198

Msg107 KTHCNYTHIQCMIFWEFSGFSSICNEIAEQCYHQINEDLAYEALLRTLPR 187

Msg34 KTHCNYVHIQCTIFREFSGFSSICNEMAEQCYRQINEDLAYEALLRALPR 188

Msg110 KKYCNYAHVQCIFFREFPNFSTVCDTMAEQCYHQINEDFAYEVLLRALPR 189

Msg8 QNEC**NYS**YFRCFFFWHFPDFLTICNLIIEKCYEKMSQHLAYKVLFKSLTG 173

Msg55 QTYCNYAHSQSIFFQDFPSFLATCNGIADQCYRRRSKEIAYEILIRAFGS 197

:. *** : :. :* .: .* : *: : ::**.: .:. **: *::::

Msg53 DSKNQVTCEEKIKESCFKLNRESYHLLWFCFLLKKTCEILVNKIRDNCGA 237

Msg46 DLKNQVTCEEKIKESCFKLNRESYHLLWFCFLLKKTCGVLLNKTKDNCDA 248

Msg107 DLENQAICEKKLKNLA-PSNRESYYLLWSCFLLKETCGILLNKTKDNCED 236

Msg34 DSKSQAACEEKIKESCFKLNRESYHLLWFCFLLKKTCGVLLNRTKDNCNA 238

Msg110 NLENQAACEEKIKEPCFKLNRESYYLLWSCFQKKYTCENLLKK-KSNCKS 238

Msg8 NLK**NQT**TCEINLKEPCSKFSTESYYLAWYCFWKRTTCMTLIKKAQDNCKA 223

Msg55 NLKGRNTCEIMIKELCLKLSTESYYLLSHCFLQEQTCEILVTRAANDCQT 247

: :.: ** :*: . . ***:* ** . ** *:.: .:*

Msg53 IE-D-LKNTLKKTNILKNDCYPLLRKCYFHSSNCENEKVQNCVEFKKRCK 285

Msg46 LK-D-IKNTLK**NAS**TLKDDCYPLLRKCYFHWPNCENEKVQNCVELKKRCE 296

Msg107 LK-N-LKNTLKNVNTLGNDCYPLLRKCYFHSSNCDEEDKQKCAKLKSLCK 284

Msg34 LK-D-L-NALK**NTS**TLKDDCYPLLRKCYFHSSNCENGEVQNCMVLKKHCE 285

Msg110 LEKD-IENTFQGAHKLENVCHSLLEKCYFHSRDCENGEMQNCMELKKRCE 287

Msg8 L**N-NS**IENALKD-HTLEKQCHSLLQRCYVHFPDCEE--TLNCENFKKRCE 269

Msg55 LK-N-FQS-IYGATSIEKTCYSLFEKCYLLSSICEE-GIQRCKNIKEQCK 293

:: : : . : : . *:.*:.:**. *:: .* :*. *:

Msg53 EK**NIT**YPPPLPPHTHSLSFNPLDFPPTLQEKIDLQQLHTEALTFGIFLGR 335

Msg46 GE**NIT**YPS-----PHSLSFNPLDFPPTLQEKIDLQQLQTEALTLGIFLER 341

Msg107 EKNIIYSLP----HHDLPFNPLNFPLTLQEKIDFQQLHTEALTFGIFLGK 330

Msg34 KENIIYPPH--THTHSLPFNPLDFPPTLQEKIDLQQLHTEALTLGVFLGK 333

Msg110 KENIKYPS-----PHNLPFNPLEFPPTLQEEIGFYRLYNEAASHGVLIVK 332

Msg8 EKGITYSP-----FHDGLFLIQGLPPTLEKRIGFQQIFAKAAATGILVSK 314

Msg55 QKNIEYPLF-PFFPHRFSFNPIEFSLTLQEKIGLEQLYVDALNYGIFLKK 342

:.* *. * * :. **::.*.: :: .* *::: :

Msg53 SLSTKFSHFLLFSNYYTGD**N-AS**KLDNTQSCTKSLENCVSFKYLTEELAN 384

Msg46 SLSTKFSHFLLFSKHYAGD**N-AS**KLDNTQSCAKSLESCTSFNYLTKELAN 390

Msg107 PLSTKFSHFLLFSKHYAGEN-VDNLDNAKLCTKSLKNCASFEYITEELAN 379

Msg34 SLSTKFSHFLLFSKHYLGDN-VGNLDNAKICTKSLENCASFEYLTNELAN 382

Msg110 SLSIGFSHFILLAYHITNP----AQ**NRT**KRCIKYLENC-TFEYLTKELAD 377

Msg8 PTITKSYRFLLLSLHNK------QISAVAGCVYYLNRC-AFKQLSEELAN 357

Msg55 SLLTDLSRFLVFTNYYDGYLESIRY**NNT**QRCVAYLRECSSFGYLTKELTA 392

. :*:::: : . . * *. * :* :::**:

Msg53 ICSATNKNEACKELNNELEREYKSLKLILYNRKLSNVSDTADS--KSYSW 432

Msg46 ICSTTNKNETCKELNNELKREYMSLKLLLYNKKLSDVSDTTDS--KPYSW 438

Msg107 ICKKTDQNKVCEELNNELEEEYMSLKLVLYNKKLSNVSDATNS--KSYSW 427

Msg34 MC**NET**DKDKACKELSNELEREYKSLKSAIYHK-FSNPSDATKS--KSYSW 429

Msg110 LC**NGT**NRTEVCGKIDKEVQRECNSLQLAFQEKNLFKMKSEAKS--KLYTL 425

Msg8 MC**NAT**NIVEICVELSKQVPEECNSLELALKNKGLF**NMS**ATDKS--DLYTL 405

Msg55 LCSTKNKDETCKQLINELQDECNTLSLDLYKK**NFT**SITVDVQSIPLTYSS 442

:*. .: : * :: ::: * :*. : .: : . . .* *:

Msg53 SELPGTISKEDCINLSHKCYSMDPYS**NNT**LYNACRNLKLECFKSATYGLA 482

Msg46 SKLPGTISKEDCISLNHKCYCMDPYS**NNT**LYNACRNLRLECFKSAIYGLA 488

Msg107 SELPGTISKEDCTSLNYKCYCMDPYS**NNT**LYNACRNLRLECFKSAIYGQA 477

Msg34 DELPGTMSKEDCISLSYKCYHMDPYSNNALYSACKNIKLECFKSAIYGLA 479

Msg110 NGLTKIIHEESYATLISKCTYIQSYCSEDLIDACLALKVAYYKAQFYKLA 475

Msg8 HGLSKIVPEEDYASLVLDCYHLESLCNQSIMDSCNNLRQVYHRSQFYNIA 455

Msg55 **NET**SETISEEECVALIQKCNYLDPFCDDIIFDACNNLRRACSKLAFYKSA 492

. : :*. * .* ::. ..: : .:* :: : * *

Msg53 RDVLEDGLFGLLHNLDS**NRT**KECIAKLIERCQIVR**NNS**IVILSMCLKPKE 532

Msg46 RDLLEEGLFGLLHDLDLDRAKKCAQKLVEKCQMVR**NNS**IVILSMCLRPEK 538

Msg107 RDVLEEGLFGLLHNLDPKRTKECIAKLVERCQMVRNNNINLLSMCLRPKE 527

Msg34 RDVLEEGLFGLLHDLDLDGTKKCIDKLVEKCKVVRNNNINLLSMCLRPKE 529

Msg110 KEVLEDGLFGLFHNLDSDGTKKCIDKLVEKCKVVR**NNS**INLLSMCLKPKE 525

Msg8 KDKLEKELFGLFR**NLS**SSGVKECAVKLTQKCQIAR**NNT**IDILALCLKPEE 505

Msg55 YEVLEEGLFGLLH**NLT**SNRLKECAAKLVKNCQAVRYNSIDILSMCLRPKE 542

: **. ****:::* . *:* ** :.*: .* *.* :*::**:*::

Msg53 TCKALAEDVERKSHRLRHILDKTRDYPREKDCLVLEKQCEDLTKDFEELN 582

Msg46 TCKALAEDVERKSLRLRHILDKTRDYPREKDCLVLEKQCEDLTKDFEELN 588

Msg107 TCEALAEDVKRKSYRLRHVLDKTRDYPREKDCLVLEKQCEDLTKDFEELN 577

Msg34 TCEVLAEDVERKSHRLRHILDKTRDYPREKDCLVLEKQCEDLTKDFEELN 579

Msg110 TCEALAEDVERKSHRLRHVLDKTRDYPREKDCLVLEKQCEDLTKDFEELN 575

Msg8 ACKIFAEDVKRKSLHLRHVLDKVRDYPQEKDCLVLEEKCEDLTKDFEELN 555

Msg55 TCKALAEDVKKKSLDLQHILDKTRDYPRENNCIILEKKCKDLTKDFNELN 592

:*: :****::** *:*:***.****:*::*::**::*:******:***

Msg53 GPCATLKRNCAHLRNTKEVKDNLLSKNTDILANVD**NCT**TYLNVKCPRWFR 632

Msg46 GPCATLKRNCAHLRNTKEVKDNLLSRNTDILANVD**NCT**TYLNRKCPRWFR 638

Msg107 GPCATLKRNCAHLRNTKEVKDSLLSKNTDILANVD**NCT**TYLNSKCPRWFR 627

Msg34 GPCATLKRNCAHLRNTKEVKDSLLSKNTDILANVD**NCT**TYLDMKCPRWFR 629

Msg110 GPCATLKRNCAHLRNTKEVKDSLLSKNTDILANVD**NCT**TYLNRKCPRWFR 625

Msg8 GPCTTLKRNCAHLRNAKKLKDSLLSKNADILANVD**NCT**AYLNMKCPRWLK 605

Msg55 APCNTLEMHCAHLRNTKELKKILLSKNSDILVNVNKCTTYLNVKCPQWLK 642

.** **: :******:*::*. ***:*:***.**::**:**: ***:*::

Msg53 REINPF**NLT**CVAHHKSCVIMIEEVQNHCLAFKENMESHDVIKKSE-DNEK 681

Msg46 REINPF**NLT**CVAHHKSCVIMIEDVQNHCLAFKENVESHNVIKKSE-DNEK 687

Msg107 REINPF**NLT**CVAHHKSCVIIIEDVQNHCLAFKENMESHDVIKNQM-AMKK 676

Msg34 KEINPF**NLT**CVALHKSCVIMIEEVQNHCLAFQQNMEDHKVIEKSKEDEER 679

Msg110 REINPF**NLT**CVVHHKSCVIMTEEVQNHCLAFQQNMEDHKVIEKSKEDEER 675

Msg8 RETNLFNLICIEHHKSCIIMTEDMQNHCSAFKENMKSQDVVKKSEDSEKR 655

Msg55 RKTNMFTLTCVAHYKTCVIIVEDIKNHCSALQQNMKNYKVIEQSN-NEEN 691

:: * *.* *: :*:*:*: *:::*** *:::*::. .*:::. :.

Msg53 DNICFLWGGYCDMLVENCPDKLKQGNNGENGLCVTLKKNCKTFHKELPLL 731

Msg46 DNVCFLWGGYCDMLVENCPDKLKL-DNGKEGVCMKLKKNCETFREKLPLL 736

Msg107 ITFVFFGVDTAICLWKTVL-INKQGNNGENGLCVTLKENCKTFHKELPLL 725

Msg34 DNICFLWDGYCNMLTGNCPDKLKQGNNGEDGLCVSLKKNCKVFREKLPLL 729

Msg110 DNICFLWDGYCDMLTGNCPDKLKQSYNGKNGLCVTLKENCKVFREKLPLL 725

Msg8 GDICFFWGGYCEMFMKSCPHKLEQNDTDINGLCAELKKNCRTFYKKELLL 705

Msg55 DNICFLWEKYCDMLIKNCLHMPKQGNNDLNRLCSKLKKNCRVVREKESLL 741

. *: . : . : .. : :* **:**... :: **

Msg53 KALMYNIKGSLTKKDACIKKLNDYCAKSTHS**NKT**LEDSCKKYGKDENIKS 781

Msg46 KALMYNIKGSLKEKDACIMKLDDYCTKSAHS**NKT**LEDSCKEYSGSKETRA 786

Msg107 KALMYNIKGSLKEKNTCINKLNDYCTKSTHS**NKT**LEDSCKKYGKDENTRS 775

Msg34 KALMYNLKGSLKEKDTCVKKLNDYCTKSAHT**NKT**LEDSCKEYKD-EKTKS 778

Msg110 KALMYNIKGSLTEKNICINKLNDYCTKSAHS**NKT**LENSCKEYSENKETRA 775

Msg8 KDLMYIMKGFLTDKNVCSKQLSSYCSNSTQS**NKT**LKNLCIEYKD-E--KD 752

Msg55 KALVYNM**NSS**LIQKNACVQQLNNYCTKLTQS**NET**LKNLCQKYNK-NEIRE 790

* *:* ::. * .*: * :*..**:: :::*:**:: * :* : :

Msg53 ETCEKFVKWMEILCNTLPVKLDKAAKDLENRANEFKKTKQETEKVI**NDS**G 831

Msg46 KTCDKLISWMKILCNALPVKLDKAAKDLENRANEFKKTKQETEKAI**NDS**G 836

Msg107 ETCEKLVKWMEILCDTLPVKLSKAAKDLENRANEFKKTKQETEKAI**NDS**G 825

Msg34 ETCDKLISWMKILCNTLPVKLDKAAKDLENRANEFKKTKQETEKAI**NDS**G 828

Msg110 KTCDKLISWMKILCNTLPVKLGKAAKDLKNRANEFKKTKQETEKAI**NNS**G 825

Msg8 KTCDRLVNRTKIFCNIFLIRLDKTSSDLENRAYEFKKIKKQAVKAVSDSG 802

Msg55 KTCNKFITWIEKICITYSTKLNKAAKDLKDKLDEFKETKKLTEKAV**NGS**G 840

:**::::. : :* :*.*::.**::: ***: *: : *.:..**

Msg53 LFLAISQIADE-KNHHL------HS**NNT**AYVRLVRRENALDIEPSVRQGL 874

Msg46 LFLAISQTADEKKNHHL------HS**NNT**AYVRLVRREDAPDIEPSVRQGL 880

Msg107 LFLTISQTADGKQNHHLSVRSNIYS**NNT**AYVRLLRREDAPDIEPSVRQGL 875

Msg34 LFLAISQTAD-KKNHHL------HS**NNT**AYVRLVRREDDLDIEPSVRQGL 871

Msg110 LFLAIPQTADGKQNHHLSVRSNIYS**NNT**AYVRLVRREDVLDIEPSVRQGL 875

Msg8 LLLAIPQTKD-KQNHYP---TNTHS**NIT**AYIRLMRRKNILNLQPSIRQGL 848

Msg55 LFLGIPPTRNRKQNHHLN----IRSNIVTHIKHLFNENILDMQTSMHYGL 886

*:* *. : :**: ** .:::: : .:: :::.*:: **

Msg53 AFDLVSLLLELYLEAKGICNHFIQECVLEDDCPKFKDSCKKIHESCKEFV 924

Msg46 AFDLVSLLIELYLEAKGICDHFIQECVFEDDCPKFKDSCGKIRKSCEAFV 930

Msg107 AFDLVSLLIELYLEAKGICNHFIQECVLEDGCPKFKDSCDKIRKSCKEFV 925

Msg34 AFDLVSLLVELYLEAKGICNHFIQECLFEDDCPEFKDSCEKIRKSCEAFV 921

Msg110 AFDLMYLLVELYLEAKGICNHFIQECIFEDDCPKFKDSCKKIHESCKEFV 925

Msg8 AFDLMSLVIELYLEAKGICDHFIRMCVFENDCPKFKDSCKKIHNYCKEFE 898

Msg55 AYSLMSLLVDLYLEAKASCDHFIVECSFENDCPTFKDLCDKIRKFCREFL 936

*:.*: *:::******. *:*** * :*:.** *** * **:: *. *

**<---------------------T-rich--------------------->**

Msg53 LPDTKP----HVTTSISTTTLTESTTVADTQSESTVVTTMIEGK-CV--- 966

Msg46 LPDAKP----RVTTSVSTTTLTESTTVTDTQSESTVATTMMEGK-CV--- 972

Msg107 LPDAKL----RATTSISTTTLTESTTVTDTQSKSTATTTIMEGK-CV--- 967

Msg34 LPDAKP----RVTTSISTTTLTESTTITDTQSESTIATTVMEGK-CV--- 963

Msg110 LPNAKP----HVTTSISTTTLTESTTVADTQSESTVVTTMIEGK-CV--- 967

Msg8 LPKATP----FVTTSISTITMTESI---------------ITGKGCM--- 926

Msg55 LPKAISLTFASASTSTSTITITDSTTIKAH--ITTNMTKTMNEK-CTSYT 983

**.: .:** ** *:*:* : * *

**<---------------------ST-rich---------------------**

Msg53 ALHSKTTWVTSKSTSTKTTTTTS-VTTL-TQKCKPVPCTTE-ETQTRKPE 1013

Msg46 ALHSKTTWVTSKSTSTKTTTTTS-VTTL-TQKCKPVPCTTE-ETQTRKPE 1019

Msg107 ALHSKTTWVTSKSTSTKTTTTTS-VTTL-TQKCKPVPCTTE-ETQTRKPE 1014

Msg34 ALHSKTTWVTSKSTSTKTTTTTS-VTTL-TQKCKPVPCTTE-ETQTRKPE 1010

Msg110 ALHSKTTWVTSKSISTKI--TTS-VTTL-TQKCKPVPCTTE-ETQTRKPE 1012

Msg8 VYHVRTSLVMHKSASTKTKINTL-VATV-TQKCNPVPCIMEKETQTKKVK 974

Msg55 KIHTKINWMTTESIFTETTISSIIVSTVSAKECEPAPCIREKKTQAEEFE 1033

* : . : :* *: .: *:*: :::*:*.** * :**:.: :

**> <--------GPI-anchor signal------->**

Msg53 TRNEADHTVMPNEGIKISGLGATSI-VIWVIGVFIVI 1049

Msg46 TRSETDHTVMPNEGIKISGLGATSI-VIWVIGVFIVI 1055

Msg107 TRTEADHTVMPNEGIKISGLGVTSI-VIWVIGVFIVI 1050

Msg34 TRSETDHTVMPNEGIKISGLGTTSI-VIWVIGIFVVI 1046

Msg110 TRTEADHTVMPNEGIKISGLGATSI-VIWVIGVFIVI 1048

Msg8 PSGETSNKIL-NKGIKISGLGIINIIIIWIVGIFAII 1010

Msg55 NEDGAN-NIVQNKEIKISELKVMNI-IIWLIGIFVLI 1068

:. .:: *: **** * .* :**::*:* :*

**Msg-IV**

**<-signal peptide->**

Msg77  **MK**AFVFVIFVILSTISYAFSKGINTY---------------ESKENLNLF 35

Msg86  **MK**TFVIVIFVILSTISYVFSKGINTYGIFFF--VFFNFYLLEPKENLIYL 48

Msg80  **MK**VFVFVIFVILSTISYAFQKALILM---------------NQKKNLNLF 35

Msg63  **MK**IFVL---TILSTIPCVFLKSINIP---------------ELKESLNLF 32

Msg70  **MK**TSII---LTLSIISSAFSKSVPVHGIFILFCFLLIFIFLESNEKLFFF 47

Msg91  **MK**AFVL---AILSIISYVFSKGIRIPGIFYFFLLFFNFYLLELKENLNLC 47

** :: ** *. .* *.: : ::.*

Msg77 TSKKIRSYGPSVHSESTDSLSKHL-LSSSAWFMVLDDDTSMYSLILEESI 84

Msg86 LQKKIRSYDPSIYSESTDSLSKHL-LFSSSWFMVMKNDVSMYSLILEKSV 97

Msg80 QKK-LGVMAHQ--YILNQLISYQNTYYLHPHVYCLDDDVSMYSLILEDSI 82

Msg63 KSE-NGNYSSPLHSTLVDFLSKRS-LPSFLWSNIKQDHISLFVLILNIFT 80

Msg70 NLE-N--YSSSDYL-TSFHMSKRS-SASYTLFSSLKDSIDISALVLGGNI 92

Msg91 KSG-DCNSS--AEYPLLNVLSKRS-LFSSLWSNIRHDPLVLSALNLDASI 93

:* : .: : * *

Msg77 KTERQCKLYLEYLCEKLVEKLPGGGFSNILKRLCNLDK-RNTYCKGLFTK 133

Msg86 TTEIDCKSYLGYLCKIFIKKLPKGNFTDILKKLCDKNK-INKYCKGLFTK 146

Msg80 TTERQCKLYLEYLCKKFVEKLPEGGFSNILKRLCNLDK-RNTYCKGLFTN 131

Msg63 QDKQECKSFLQEICNDLLKKLLEGSFVDILKTICTLKK-IDAL-QDLTES 128

Msg70 NNREQCIDFLESICEILYPELSEGPFTNILKKICEIEQGLSKLCQYLTAK 142

Msg91 KNEEECLLLVRELCEGLLPKLPRGGFTSLLKTFCELAE-ADDLCTHLTKL 142

. :* : :*: : :* * * .:** :* : . *

Msg77 TST-IGKDLLIKCNGIKDKFNYYYYILGKTLFTGEWNARIEAEDCENYET 182

Msg86 G-G-VKDDLRKKCDGIKSKLEYYYY-LGKTLFTGEWNARIETEDCENYET 193

Msg80 G-D-IKKDLNKKCNGIKEN-SIIII-IGEDLFVGEKNTRIEAEHCENYGT 177

Msg63 TIS-KPTSIQRKCQDLNSRLNEFFN-SNEDLLSEGSNKRLGIADCENYST 176

Msg70 SRHTNFTLIEKKCHDLENALSVY-N---DNFNLKNGPDKL-KGRCNELEI 187

Msg91 KKS-GFTPVQRKCNDLKNELSMFFG-SNDELLLESQNKKLTIGDCENYDT 190

: **..::. . . : :: *::

**N-glycolsylation sites (NXS/T, X≠P)**

Msg77 LCIVFQRVCYGLISDLCGKFNNLCYKKYIKDTEDSVLLEFIGKEALS**NES** 232

Msg86 LCIVFQRICYGSIGNLCARFNGLCYKKYTKDAEDGLLLKFIGKKTLYNEL 243

Msg80 LCAVFQRICRDLIGDLCSRFNGLCYKKYTKDNENNMLLKFIGEK-ISSEL 226

Msg63 YCNIYQRICGIATSNLCGELQNACYLKYRENFRNTILSLILKDS-LKVST 225

Msg70 LCNIYGPICGRSISVLCSKISADCFDLRYKDHQNSLLLQVLRDD-LN--- 233

Msg91 LCNIFHRICGDSTRDLCTRFKSMCYSKLREDFKKALVLALVKDS-IH--- 236

* :: :* ** .:. *: :: .. :: .: .. :

Msg77 KNNELEHE--HLYTQCIHTLLKKCHCITSFGPTMAESCSQLTDTCNHFNR 280

Msg86 EDNKSEHE--YIYTKCIHSLLEKCHRITSFGPTMVESCYELTDTCNRFNQ 291

Msg80 SHNNLKHDESEEYSQCFDSLLDKCHCVTSFGPTMVKSCFEIQDTCKHFAQ 276

Msg63 L---------ENHQECVDDLCRKCFCVIGLGPDIVEACLQVWSTCKSLVE 266

Msg70 -----------DPEVCRKSFHNKCLCLSTLAPTMADVCLLVEATCENLIE 272

Msg91 -----------RREECVTDLLKKCHCLVGLGPDMVEACLQVWRTCETTAK 275

* : ** : :.* :.. * : **: .

Msg77 STTITCRYLDSYIKNFLLEK---QEHITGYKKKITRN**NCS**LLGACEYYS- 326

Msg86 TTISTCRYLGSYIEEFLSEK---QEHITGYEEKITRN**NCS**LLGACDYHL- 337

Msg80 SIIFTCRCFDYYIKEFLSED---L-HITGYKKDIARN**NCS**LLDVCEQYIL 322

Msg63 AIDETCLYFNLYIQNSLSRN---LKHTSGYGRTSSEKECLLVTYCDYYT- 312

Msg70 HKEQKCSALKSVIYEITSDGLENLEESLSASGNTLETDCTSFPPCIDFL- 321

Msg91 TTSEICSQLSSSIKKSFLKN---LEYTSDYTHESLESRCSLISTCDYYT- 321

* : * : . .. * . * .

Msg77 LLCH**NKT**TKNLCKPIIEECSAKADLDMGFVKNLSLGKCKSTFESVNLTRF 376

Msg86 LLCH**NKT**TEGLCKPIIKECSAKVDLEMDFVEKFFLGECKSTFQNVNLTRF 387

Msg80 LHCHTQTTKNLCNLIIKECTKKIDPDMLFINNISLGECKSTFENVNLTLS 372

Msg63 SFCGDYTTKILCVELKQRCTGRA-VDMPFVKKIELGKCSSVFENVNLDSF 361

Msg70 SYCDNLETRKHCQHIKQKCTQDN-LTEYSTTKVSLGDCVSAYSSVDLDAF 370

Msg91 LFCGNSETKNLCSTLKKECIGSN-SDIYSTGMNLLGQCAHEFKEVDLSAF 370

* *. * : :.* **.* :..*:*

Msg77 FYEKEKSGVLLPHKAPYLTPLLMFFSSLGRSTLTLKERCLNFIKKNCLAL 426

Msg86 FYEKQKSGVSLPHKAPYLTPLLMFFSSLGRSTFTLEKRCLSFIKENCMAL 437

Msg80 FFNERENSVSLPYKNPYLTLLIIFGASLMRRGSSLNEKCMNFLKHDCTNH 422

Msg63 FSQTQKSGISFPYKHPYLSTIILFISSLMSHRGDLQEKCRIFLEHYCDYY 411

Msg70 FHKLHDNHLLFPFGYPSLRTILLFLVSLMQHRGNMQVRCQSFSSRYCQYY 420

Msg91 FSRERENGGLFPYKPPYLFPLITLISSSMAHLTNLEGRCGIFLEQSCNYY 420

* . ... :*. * * :: : * :: :* * .. *

Msg77 QGMFPNLHEYCKSGYTDECEQLDEKM**NTTC**INL**NKT**FEELGLTSTNGVWT 476

Msg86 YGMFSNLHEYCKSRYTDECNDMDGRM**NTTC**VNL**NKT**FEDLGLTSTKGVWT 487

Msg80 QRVFPNLDAYCQSGQTDECDNLNKSI**NKTC**VNL**NRT**FDDLGLVSANGVWT 472

Msg63 QTTFLNLDKYCSEGYNEECEHLNSKTEESCNNLRNVAQGLGLVSGQGISV 461

Msg70 RGVFPVLHSHCNSDF-SECDNLDTKATKACADLKKKLHELGLVREDGDSP 469

Msg91 ETVFPNLDEYCNTRQTDECSNLDAKASRACTRLKDRLEDLGF-SENGSPV 469

* *. :*. .**..:: :* *. . **: .*

Msg77 ILW**NS-T**ANNITTPQCQMLIEECTYFRPGCPGIKSPCENVKALCYTLSKK 525

Msg86 ILW**NS-T**ANIITTPQCQMFMEECIYFEHVCSGIKSLCENVKALCYTLGVK 536

Msg80 ILW**NS-T**ANIITTPQCQMLIEECTYFEHACLGIKSPCENVKALCYTLGIK 521

Msg63 IMHSGMLSDHMTVSQCLSLIHQCVYFAHICENIQGICKNVRVTCYNLAIQ 511

Msg70 KLYRGAHHDSISVGECATLIAECHYYSYLCPELRGPCRHLHPLCYNHNKE 519

Msg91 MLSSDIMDSRVTVAQCVVLIQECVYFSHVCSGIRHLCEELKVVCQELGIQ 519

: . . ::. :* :: :* *: * :: *..:: * :

Msg77 RSHLNYFWEKLEEKLSPSDFSIF**NQS**NLPQSSAASNLYQHIYKHILDICA 575

Msg86 RFHLNNFWKKLKEKLSPSDFSIF**NQS**HLPQSSDTSKL----HQPILDICA 582

Msg80 RSHLNNVWKKLKEKIPSTDFSIF**NQS**HLSLSSDISNL----YKPILDICA 567

Msg63 KHYMSTFWGKFNKNILLAGPNNFNLRLL--LTENSGI----LDHIMTICS 555

Msg70 KYYLGRFWDKIKEHITSKGFRTADLLHP--KT-SGEL----YPYILKLCS 562

Msg91 RYSLGLFWKEFEKRLPLDTFSASNIKHQ--LSHESN-----LHHILTVCA 562

: :. .* ::::.: : : . *: :*:

Msg77 ELGGTNEVLFRWCLHPIPITPPAPIIFPGNLLDRLLYDFSARRDDLERGL 625

Msg86 ELGGTNEVLFRWCLHPIPITPPAPIIFPGNLLDRLLYDFSARHSDLKRGL 632

Msg80 EFGGTNEVLFRWCLYPTPNDPPG--NFPRDLLDRLFHDFSARHEDLKRGL 615

Msg63 ELGGSNEVIFTWCLYPATI------------PRKLYYYLVEGYEELEKDL 593

Msg70 THGTTNELFFQWCLHPLNI------------PRMFYYSFKYGSIDLLSNS 600

Msg91 ELGGLNEILYRWCLHPSKF------------TERLDYYLSLGYKGLIQDL 600

* **::: ***:* : : : * .

Msg77 LYVGEPPSLAECAPYVYECHNLLEIFKDLTT**NCT**ILENECYNGY-K--DY 672

Msg86 LYVSEPPTLTECASYVYECHSLSDIFK**NI-TNCT**MLEEACYSGH-I--NY 678

Msg80 LYVSEPPTLTECASYLYECHSLSNIFKDL-T**NCT**MLEEACYSGH-K--NY 661

Msg63 PKPNEQPSLSECIYYIAECNGLSNIFPKLKDSCTRLENKCFGYN-Y---K 639

Msg70 SHENEKLSLPECAFYSFQCSQFT-VFDDSKHTCDKVNEICFPQDDFQYSF 649

Msg91 PEVTQKPSVSECMYYTEECDLLIAVFGDPNGLCEKLKDLCYKYD-L---K 646

: ::.** * :* : :* . * ::: *:

Msg77 DKLENSKLIKSI 684

Msg86 SKLKDSTLSKSA 690

Msg80 GKLKDFKFSKST 673

Msg63 DRFENIEPVKLT 651

Msg70 GNDTDFGFTK-N 660

Msg91 NDFKELESI--K 656

. :

**Msg-V**

**<-signal peptide-> N-glycolsylation sites (NXS/T, X≠P)**

Msg101 **M**QLFLGTCIFLVFISIKRVFSEDDD---LVN--DYSPLYEDDPLLSSILR 45

Msg54 **M**QLFLGTCIFLIFISIKRVFSEDED---LIN--DYFPLYEDDPLLSFILK 45

Msg47 **M**QLFLGTCIFLIFISIKRVFSEEED---LIN--DYSPLYEDDPLLSSILK 45

Msg28 **M**LFFTKIYVFLIFIIIRNVFSKNED---LIN--HYSPLYNEDLLLFHTQR 45

Msg97 **M**SLFVKAYILLNLIDIKTVFSKNED---FLDLI**NYS**PLNEDDSLFPLTHR 47

Msg22 **M**SLFVKAYILLNLIDIKTVFSKNED---FLDLI**NYS**PLNEDDSLFPLTHR 47

Msg11 **M**FLFMEICILLLVVAIKRVFSENEDSLDLIQ--TFSPLHDEDPLLSYEVT 48

Msg42 **M**QLFTKTCIFLIFISIKRVFSENNEILDLVQ--TFSPLHDEDPLLSYEVT 48

* :* ::* .: *: ***:::: ::: : ** ::* *:

Msg101 SDEYHVQLYEQLKWIEHENLRQNYIDDYYYLAIILDNYYYLAIILDSYND 95

Msg54 NDEYHVQLYEQLKWIEHENLRQNYIDDYYYLATILDN------------- 82

Msg47 SDGYHVQLYKQLEWMEHENLRQNYIDDYYYLAIILDN------------- 82

Msg28 SYMYYTNLHHSLEKLDLENLWYNYIDDYYYVITML--------------- 80

Msg97 SALYDLRLAYQLQKLELENLWYNYKHDYYYVAAML--------------- 82

Msg22 SALYDLRLAYQLQKLELENLWYNYKHDYYYVAAML--------------- 82

Msg11 TEEYHHQLSKKLHNLELKELKNS-NEYLYYLATFL--------------- 82

Msg42 NEEYHYQLNKKLYKLELEKLKNSKEYLYYYLTTFL--------------- 83

. * .* .* :: ::* . **: :*

Msg101 LVTILDNNDLYRYCEAKLKETCI-SIEDVNKRFQKICKNPEASCKGAYSK 144

Msg54 ------YNDLYYYCEVRLKEACI-NIEDVNKRFQEICRNPKASCNDAYYK 125

Msg47 ------YNDLYRYCEVKLKEACI-SIEDVNKRFQEICRKPKASCKDAYYK 125

Msg28 -----TPDQ-LYDCKSNLVKVCT-ELKNINEKLRKICEKPKMVCGLAYNE 123

Msg97 -----APGR-LSYCQNDMTKICT-NIKNMNERVQGICTS-KTICNHAYNE 124

Msg22 -----APGR-LYYCQNDVNEICA-NIKNMNERIQGICAL-KTICNRVYNE 124

Msg11 -----TFNDFNNNCIAKLKKVCNSDVKMLNSHIHYICNNLQLLCTSSNTQ 127

Msg42 -----TSSDFNNNCIAKLKEVCDNDVKMLNDDIHHICNNPQLVCTL-NTQ 127

. * : : * .:: :*. .: ** : * :

Msg101 IVNKAKEIAISF---SDKETNERDQCNKLQVKCFFLELHGSWHIGAKCAT 191

Msg54 ITSKAKEIATSF---SNKETNEHDQCNKLQVKCFFLERYGSKHIGTKCAT 172

Msg47 ITKKAKEIATSF---PNKETNERDQCNKLQVKCFFLEHHGSGYIGTKCAT 172

Msg28 MMLKIKKTVEEL---SDERITEHDKCNKLQMRCHFLEHHKPWYLNSGCKK 170

Msg97 IMTKIKKTAQEL---SDEKISEHDKCNKLQMRCFLFERHGMQYLNEKCKE 171

Msg22 IMMGIKKTDEEL---SDKRITEHDKCNKLQMRCFLFERHGMQYLNEKCKK 171

Msg11 MSNAIKKIKDFL----D-ETNYHNRCSKLQTDCFFLEQYSPEDLSAKCNK 172

Msg42 ISNAIKKTKDDLDKETDKETN-YHHCSRLQTECFFLEQYGSGDLSAKCNK 176

: *: : : . . .:*.:** *.::* : :. *

Msg101 LKEYCYNKVRMGVAEIIVYDFLRGTSNNLDTCIRKLQNECQLVNQRSPEL 241

Msg54 LKEYCYNKVRMGVTEVIVYDFLRGTSNNLDTCIRKLQNECQLVNQRSPEL 222

Msg47 LKEYCYNKVRMGVAEVIVYDFLRGTSNDLDACTRKLQNECQLINQRSPEL 222

Msg28 LKEHCYKKVRMEVAQVILYDLLRGTSNDFNKCLRKLKSECLSLNQRSPEL 220

Msg97 LKESCYNKVRMEVARTLLYDILRGTSSDSKSCIGRLKNVCQLLSQQSPEL 221

Msg22 LKESCYNKVRMEVARTLLYDILRGTSSDSKSCIRRLKNVCQLLSQQSPEL 221

Msg11 LKENCYNKARMEVAEGIMHRFLKGTHS--STCLQKFKEKCLLFSHQSPEL 220

Msg42 LKENCYNKARMEVAEEIMHRFLKGTHD--KTCLGKLKEKCLLFSHQSPEL 224

*** **:*.** *:. ::: :*:** . . * :::. * ..::****

Msg101 FDLCVNVDTVCSKFISKSKEECQNSQALFSSN--QISEKECKTLLAKCYS 289

Msg54 FDLCVNVDTVCSKFISKSKEECQNSQALFSLN--QISEERCKTLLAKCYS 270

Msg47 FDLCVNVDTVCSKFILKSEKECQSSQALFSSN--QISEEKCKTLLAKCYS 270

Msg28 LDLCTNGANVCLEFISNSKKECQNFQNLFLSEQKTISEKNCLNWLKICYY 270

Msg97 LTLCMDYTRVCNEFTSYSQEECKNFGTQFLSKQGIMTEENCVKWLEICYY 271

Msg22 LTLCMDYTRVCNEFTSYSQEECKHFGTQFLSKQGIMTKENCVKWLEICYY 271

Msg11 FSLCISGSNVCEKFISETKTNCQNFQIQFLSKHKEITRENCIVWLKKCYY 270

Msg42 FSLCISGSSVCEKFISETETNCQNFQTQFLSKHEEITQENCIIWLKKCYY 274

: ** . ** :* :: :*: * : ::.:.* * **

Msg101 ILSDCPVLRPKCRSLKMECAEKGFFYDISENHNFNLLENPFIHMNRNGVE 339

Msg54 ILSECPILRLQCRSLKMECAEKGFFYDISENHNFNLLENPSTHINKNGVE 320

Msg47 ILSDCPVLRPKCRSLKMECAEKGFFYDVSENHNFALLENPFTHMNRNGAE 320

Msg28 IIPDCRVLYHSCHSFRMSCAESGFFFN-SKDYNFDLFENPSTYMVENGVQ 319

Msg97 VIPDCQNLHSLCRSFRMECSEKGFFYN-PRGHNFDLFKNPSTDLDETDIQ 320

Msg22 VIPDCQNLHSLCRSFRMECSEKGFFYN-PRGHNFDLFKNPSTDLDETGIQ 320

Msg11 NVLNCRNIYHMCRLFQTNCAENGFFSNFDSNYNLNLLESPFIDIDEDTIQ 320

Msg42 SVLNCRNIYHMCRLFQTNCAENGFFFNFDSSYNLNLLESPFINVDDDTIQ 324

: :* : *: :: .*:*.*** : .:*: *::.* : :

Msg101 YAYGKLYNSGIYVAKIQ**NLS**DLLIADFLI**NN-----T**KAVDRDCKKVLDE 384

Msg54 YAYGKLYNSGIYVAKIQ**NLS**DLLIANFLI**NN-----T**SAGDTYCKKVLNK 365

Msg47 YAYGKLYNSGIYVAKIQ**NLS**DLLIANFLI**NN-----T**RAGDKNCKEVLNK 365

Msg28 YAYEKLHNLGIYVSEIQDLPEIFVLELLVQK-----ITTGRNDCVKALNK 364

Msg97 HTYEKLHDLGIHVTGLEKYSELQVANFLVQEYLVQEYYPGYLQCKKIFDQ 370

Msg22 HTYEKLRDLGIHVTGLKKYSELQVANFLVQE-----RYPGYLECKSALNK 365

Msg11 YSHKKLSQLGIYVDKMPSLPNEVIASILIQD-----VSAQISKCKKELNE 365

Msg42 YSHDKLNQLGIYVDKMPSLPKEVVPSILIRN-----VNARTTECKEELNK 369

::: ** : **:* : . .. : .:*:.. . * . :::

Msg101 KCSSINYLNYIEPMC**NAS**S-NSEYKICKSIYYKTRDYCYSLLEKFKESNQ 433

Msg54 KCSSINYLSYIKPIC**NVS**S-NNEYKICRKIYYKTGHYCYLLLGKFEKSNQ 414

Msg47 KCSSINYLSYIKPIC**NVS**S-NGEYEICKSIYYETKKYCNLLLKKFEESNQ 414

Msg28 RCPSAEYLEFAKSICNIKFQSGIHYACINLNHKAEVYCEPFLDKLKKGIQ 414

Msg97 KCSSIQYLEPIKQMCTSKDSRNLYNVCHDMYAGTRNYCYSLQSKLKQGIW 420

Msg22 KCSLIQYLESIKDMCISKDWKGNYNVCYNMYAGTRDYCYSLIKKLKEGIN 415

Msg11 KCPLVEYLDVFKDICSKTN-N-KDNICKGIYDKTKQHLGPYSKEFSGNN- 412

Msg42 KCPLAEYLDVFKNICTKTS-N-GHNICKDIYDKTKLHFESYSKNFTSNN- 416

:*. :**. : :* . * : : : :: .

Msg101 FLKYSDRPLSKTECEEYLSICYFIDKYFNYWNSGFWKKYDTCKYVRIVCY 483

Msg54 FWWYPDRPLSKIQCEEYLSICYFIGKYFHYW-----RGYDTCKYVRVICY 459

Msg47 FLKYSDRPLSKTECEEYLSICYFIDKYFNYWNSGFWKKYDTCKYVRIVCY 464

Msg28 FWWYPHLPLSETECIEYLSACYFVDNYFIYW-----KTYDICKDIRLVCY 459

Msg97 FWWYPDRPLSKEECKEYLLVCYFMHKAITYW-----LSYNLCKDIRLVCY 465

Msg22 FWWYQDLPLSEKKCEEYLTMCYFMYKTITYW-----TGYDICKNIRLVCY 460

Msg11 -LSISQKSFSKEKCIEYLPLCYFFNEYFDVW-----VQSDLCKTIRLACY 456

Msg42 -LSISQKSFSKKECMEYLSLCYFFNEYFNIW-----VQSGLCKTIRLACY 460

. .:*: :* *** ***. : : * . ** :*: **

Msg101 QEDLDAATNMILIKKLSGRFKLKRNFQGIMALHRTMSDCKNALLEECGNF 533

Msg54 QEDLDVATNLVLIKKLSGRFKLKRNDQGIIALHRTMSDCKNALLEECGNF 509

Msg47 QEDLDAATNMILIKKLSGRFKLKRGDQEIIALHRTMSDCKNALLEECGNF 514

Msg28 QAGLEIAANKALIKRLSGKLRLGKSYSESLT-DRVLNDCKKVLLKECGQF 508

Msg97 QAGLERAANMALMKKLNRKLTLENNPESS----NNQKNCEKVLIQECKHF 511

Msg22 QADLEKAANMALMKKLNRKLTLGNSPESS----SNQKNCEKVLVQECEHF 506

Msg11 QADLEMEADMILIRKLNEKISSRDRWSSSF---**NLT**K**NCT**KRLSLVCKEV 503

Msg42 QADLETEADMILIKKLNEKISSRNRWSSSF---**NLT**K**NCT**KRLSLVCKDA 507

* .*: :: *:::*. :: . .:* : * * .

Msg101 MYHSYHILYKCLHPEETCK**NLT**NLLDKNCERLEKNLKAAIPNPEYTTCRQ 583

Msg54 MYHSYPILYKCLRPKETCK**NLT**NLLDKNCERLEKNLKAAIPNPEYTTCRQ 559

Msg47 MYHSYHILYKCLRPKETCK**NLT**NLLDKNSERLEKNLKAAISNPEYTTCRD 564

Msg28 MYYSFHVLYKCLHPQETCK**NLT**NILYKDFKRFDETLKYVIIEPTYDTCKE 558

Msg97 MYHSYYILYSCLHPKEACK**NLT**TFVNSNSKDLEKNLKDTLMDPDYDKCKK 561

Msg22 MYYSYYILYSCLHPKETCK**NLT**AFVNSNSKDLEKNLKDTLMDPDYDKCKK 556

Msg11 MYSNYHVLYRCLHLQETCTKLASFVQEKCDKLRNTLKNINNNFTLTSCGK 553

Msg42 MYYNYYVLYRCLHLQETCTKLASFVDEKCSRLGETLKKMNNNFTLSSCGK 557

** .: :** **: :*:*.:*: :: .. . : :.** : .* .

Msg101 LKEECDELGPYSEYTLILCKIFKKMCENVDKLMEFKLSTLKEKNSVLKNN 633

Msg54 LKEECDELGPYSEYTLILCKIFKKMCENVDKLMEFKLSTLKEKNSVLKNN 609

Msg47 LKEECNELGPYSEYTLMLCKIFKKMCENVDKLMEFKLSALKEKNSVLKNN 614

Msg28 QREECRRLGPYFEYTLNLCKDLDQSCKNIDELITLKSKILKENDSNLLNE 608

Msg97 YKKECHKLEFYFQSTKKLCGELNTACKNIDEAILLGVNILKK-GSDLYNE 610

Msg22 YKKECHKLGFYFQSTKKLCGELNTTCKNIDEETLLGVNILKK-GSNLYNE 605

Msg11 QKEECNSLKLCSGKTISLCQNLTAYCRNKDELENLKLKILKNDTVFLQSQ 603

Msg42 QKKECNNLKLCSGNAISLCQKLERYCRNMDELNNLKLKIQKNNTVFLQSQ 607

::** * : ** : *.* *: : . *: * .:

Msg101 TTCLNYLNNYCSE**NVS**--SHICTNK**NES**CTQMLDQIPMHCEQLSFYLSYY 681

Msg54 TTCLNYLNNYCSE**NVS**--SHICTNK**NES**CTQMLDQIPIHCEQLSFYLSYY 657

Msg47 ITCLNYLNDYCSK**NFS**--SHICTNK**NKS**CTQMLNQIPMHCEQLSFYLSYY 662

Msg28 TTCLSYLNNYCIQNNI--GYACV**NKT**ISCKHMLDYVSKQCQQLSRHLLYY 656

Msg97 SSCLKHLAEHCRQ**NSS**YSHTVCRDKIKTCNHILKRVSAYCTQLSHYLSHY 660

Msg22 SSCLKHLTEHCTQ**NSS**YSHTACRNKTKTCNHILKRVSAYCTQLSRYLSHY 655

Msg11 TVCFGYLTDYCLK**NSS**HYTYGCT**NAS**KICKEILRSVEDDCKKLLQYVNAY 653

Msg42 PVCFNYLTEYCLK**NSS**HYTYGCT**NAS**KICREILSSVKEDCKKLLQYVNAY 657

*: :* ::* :* * : * .:* : * :* :: *

Msg101 KSSNGQINHINYGRCSQFLNYCEMLKETCP**N-LT**SICSQVEQSCNKVSTR 730

Msg54 KSSNGQIDHINYGRCSQFLSYCKMLGEICP**N-LT**NICSQVKQSCNKVSTR 706

Msg47 KSSNGQIDHINYGRCSQFLSYCKMLEETCP**N-LT**SICSQVKQSCNKISTE 711

Msg28 GFSNKQVTHMEHSRCSYFIHYCKMLKRNCPNSLDDMCSEVEKICDNISTR 706

Msg97 EVSNNHITHISYSRCASFKYHCKLLKSSCPGSLNNICAEVEKKC**NNT**STQ 710

Msg22 EVSNNHITHISHSRCVSFKYHCKLLKGSCPGSLNNICAEVEKKC**NNT**SAQ 705

Msg11 KTLLNGVSYITYNRCFHFKYYCVLYIGGCPY-LRDLCS**NIT**IICSRISKA 702

Msg42 KTLLSGASYITYNRCSHFKYYCVLYIGGCPS-LHDLCS**NIT**IICSSISKT 706

:: :.** * :* : ** * .:*::: *. *

Msg101 KKNVIALIKVVGEEVS**NHS**KCKKKLHEVC**NNT**ILEKTMNELCTDI**NDT**CK 780

Msg54 KKNVIALIKVVGE-VS**NHS**KCEKKLHEVCN**NIT**VKKTMNGLCTDV**NDT**CR 755

Msg47 KKNIISLIKVVGE-VS**NHS**KCEKKLREVCK**NTT**MKEKMNELCTKV**NDT**CK 760

Msg28 NKKFNDLIKILNEEISTHDRCKKKILKECE**NST**IKQIINILCTNADDTCK 756

Msg97 TNELNNLIKAFGEGISTHEKCKKKLHKICE**NST**TKQKM**NIS**CTNI**NNT**CK 760

Msg22 TNELNNLIKAFGEGISSYDKCYKELTQECK**NFT**VKK-M**NIS**CTDV**NNT**CK 754

Msg11 SENFNALAKVLGG-ISNVSECEEKMK-KCKNSAIEK-ERQLCD-GSIQCE 748

Msg42 SESFNALAKVLGG-ISNVSECEEKMK-KCKNSADQE-KKKLCN-GSIQCG 752

.:.. * * .. :*. ..* ::: *:* :: . * . *

Msg101 ILKLHLEEICDQLTLKIFKFLFTNSKSKIECQKLTPLCSSIASSC**NET**NV 830

Msg54 ILKLHLEEICDQLTLKIFKFLFTNSKSKIECQKLTPLCSSIASSC**NET**NV 805

Msg47 ILELHLEKICDQLALKIFKFLFTNSKSEIECQKLTPLCSSIASSC**NET**NV 810

Msg28 HLRKHLEKGCDQLALEIYKFLFANS**NST**TECKNLASICSSIGYSCGKINN 806

Msg97 HLIDHLGKICHGLALKIFKFLSTPSN--SECENLKSLCSSIGNFCNGIND 808

Msg22 HLVDHLRKFVI-ILHKIFKLLSTNF**NST**IECQKLISSCSFIGSSCNGIND 803

Msg11 ALISYLEKMCDTLALKVSLYYHTKPHSSTECKKLESLCSSIGSSCTGVNS 798

Msg42 TLISYLEKVCDELLLKVSRYYYTKRHSNMECKKLKSLCSSIGSSCTGVNN 802

* :* : : :: : : **::* . ** *. * *

**<---------------------------**

Msg101 KILPLCH**NFT**SVCSKLLE----QPKP-SELP-ELPEPL------------ 862

Msg54 KILPLCH**NFT**SVCKL-LE----PPKP-PAPP-APSEPS------------ 836

Msg47 KILPLCH**NFT**SVCSKLLE----PPKP-PAPP-APPAPP------------ 842

Msg28 KLATICS**NFT**IKCKL-LESMLPPPPPSPLPP-LPPSEPSEPIPPPKPIPP 854

Msg97 KIIRIC**NST**MTKCTS-PG-----PL--PPQP-QPP--------------- 834

Msg22 KITRIC**NNT**ITKCSS-PS-----PPPGPPPP-VPR--------------- 831

Msg11 RVSYICTKLAIKCKL-PQ-----PPP-PVPP-QPPQPP------------ 828

Msg42 RVSSVCDKFILDCKS-PQ-----PHP-PSPPPLPPPQP------------ 833

:: :* . *. * . *

**----------------------PE-rich---------------------**

Msg101 ----EPLEPS-EPSKPSKPEIPLEPAPK--PSKP---P---------A-P 892

Msg54 ----EPPAPS-EPSE--SPETPSEPVPK--PPEPETPP---------E-P 867

Msg47 ----APPAPS-EPSE--TPETPSEPVPK--PPEPETPP---------A-P 873

Msg28 SEPIPPPAPS-EPSTPPKPIPPSEPIPPPAPPAPPAPP---------A-P 893

Msg97 S---EPPPPP-EP------LPPKP--------PIPPPP---------K-P 856

Msg22 P---QPPKPP-QPQPHP--QPPPP------PPPPPPPP---------K-P 859

Msg11 ----SPPLPQPHPQP--QPPEPPEPPPK--PPKPQPPPPSPPEPQPPPPK 870

Msg42 ----HPPSP---PQP--HPPSPPQP---------HP--PSPPQPHPPS-P 862

* * * *

**-------------------------------------------->**

Msg101 S--------------------------EPPGSPESP--ELPEPKP----- 909

Msg54 PAPETPS--EPAPKPP--ESSTPETPSEPSEPPESP--ELPEPKP----- 906

Msg47 SEPSEPS--ET-PETP--SEPVPK-PPEPETPPAPP--ELPEPKP----- 910

Msg28 PAPSEPS--TPPAPPAPPAPSEPSTPSTPSAPPTSLPPSKPVPLPSSSQS 941

Msg97 SEPES-SKPMPKPKPTNSTTSLPTTNSSTAASPTTP--SFPTTLI----- 898

Msg22 SEPES-S--KPKPKPTNSTTSLPTTNSSTAAPPITS--SLPTTLI----- 899

Msg11 PQPQPPSP--PKPLPP-----PPE-PPKPSE-P-DP--SRPKPKP----- 903

Msg42 PQPPEPQP--PEPQ-------PPE-SPVPSTPP-KP--PKPKPKP----- 894

. . * * .

**<---------------------------------------------**

Msg101 --------------TLTNLTVILT----STQL--LTI-YLPT-------- 930

Msg54 --------------TLTNSTTILT----STQL--LTI-CLPT-------- 927

Msg47 --------------TLTNSTTILT----STQL--LTI-CLPT-------- 931

Msg28 VLRPTESSTTSSSSTFSNSSIISS----LTSTTSESSIVLDS-------- 979

Msg97 --------------PTTNTSDIYT----STTATSETS-ILDT-------- 921

Msg22 --------------PTTNTSDIYP----STTSTSKTS-TLDT-------- 922

Msg11 --------------KPTNSTTSLTTSLPTADS--STA-APPTTLD----- 931

Msg42 --------------KPTNSTTSLP----TTDS--SPA-APSTTLDTDTPT 923

:* : . : . :

**----------------------ST-rich---------------------**

Msg101 TSTMH--------------------------------------------- 935

Msg54 TSATHSSATHSSV---------T--------------------------R 942

Msg47 TSATHSSATHSSA---------T--------------------------R 946

Msg28 SITYNSSSASSSI---------TSNLPITSNISTLTNSTILTGSLTTHLT 1020

Msg97 STSTDTSTRTSTR---------TS-----------------------TRT 939

Msg22 STSTDTS----------------------------------------TRT 932

Msg11 ---TDTSS----------------------------------------RT 938

Msg42 RTSAHTSSRTSSRTSTARTSTDTS-----------------------TDT 950

.

**----------------------> <-----GPI-anchor signal---**

Msg101 ---SSATRS-TTRSLRPKPTTSSDDRRIVGFGVRPRKLREIELIWMVAGT 981

Msg54 ---SSTTRS-TTRSLRPKPTTSSDDRHIVGFGVRPRKLREIELIWMVAGT 988

Msg47 STIRSTIRS-TTRSLRPKPTTSSDDRRTVGFGIRPRKLKRIELICMVVGT 995

Msg28 STYLTSTRLTSIYSSKSVLTTSSDSH-EKGYGIKI-ELQMIKLIWTTIEI 1068

Msg97 SKGTSTTRSSTSTRSRPRPTMSRDDY-EEGYGLRAQRLQMIKLILEIAGI 988

Msg22 SKDTSTTRSSTSTRSRPRPTMSRMTM-RR-LWTQSTGITNDKIDLEIVGI 980

Msg11 SSHTSTTRTSTSTRSRPRPTITPGGGRGKGYGIRTQGLQIVELIWTTIGI 988

Msg42 STDTSATGSPTSTRLRPRPTISPGDGRGKGYGIRTQGLQIIELIWMTIGI 1000

:: : :. * : : : ::

**-------->**

Msg101 ILGMWII-V 989

Msg54 ILGMWII-V 996

Msg47 ILGMWII-I 1003

Msg28 LLGLWIIVI 1077

Msg97 ILGLWII-I 996

Msg22 ILGLWI--I 987

Msg11 ILGLWII-I 996

Msg42 ILGLWIVIL 1009

:**:** :

**Msg-VI**

**<-signal peptide->**

Msg49  **M**KTSVFLVFIGTTCVLSKGIVT--PRHQDKSST-LKKTQLNHDHILVDYA 47

Msg51  **M**KASIIAILVGVTLVFSKNNER--D---YASSI-LKRDNLNYYRVTRDFF 44

Msg73  **M**KLPIFSIFLSITCVLSRADES--K----NNHI-SNRKFLNDDEAFAHFS 42

Msg39  **M**RASVFAFFVGVVRALLKDINI--AAYENSDNILDNTGYLENNNIYL--- 45

Msg59  **M**KISVFIFFIGIVHALLRDIGVSEFTYENLNSILDNKKYIDDDDTYSYIS 50

Msg18  **M**RVFIYGIFIAFTNALSEKISD--SASQNLN-ILDNEEKLIFNHVYPYKS 47

*: : .::. . .: . . : :

Msg49 YDDPENGVNSVNKLTKELNEILDKVSLVLDNKIYWKRYDFSDDSSSK--- 94

Msg51 PRE---SRDSLNKLTGNLKKILEEVDSLLNGKIQWKEHDSLDDS-SN--- 87

Msg73 QGD---SPDFNKKIKKDLLEMKSKFDEIIS-TLQNQQQNNLDDSLKP--- 85

Msg39 -------------------------------------------------- 45

Msg59 LKQ---SL-YSLTQDDNLFELIRDFQVTFQ-TKQNPESETIEKL-APRSA 94

Msg18 FEK---RS-NFMSFDEYINKQLNEIQNFLH-KINLENHDLSQ-------- 84

Msg49 -----TEGYDNMKKQDIA-LKNILEDYLMSYHNTDKCLQTVNAYRSLLAT 138

Msg51 -----AEKYNILEEQNSR-IMNILQGYFMNCYSTDECARTIQDYRSALKT 131

Msg73 -----LAEYLSGRSLEDD-LVSSMLSYFKGHNTIQECKIFQKIYSSALNK 129

Msg39 -------------------------------------------------- 45

Msg59 PAKDGVAQKEAVSTEDVY-FLLLLANFLQRPLTHQLCVTVLTNICPTINN 143

Msg18 --------GNYLTSEDYGLLAVIIATYFNRKYTVIECVNILTRFCQHMNS 126

Msg49 FKDR--NEE-VAKLDSL-LSNKDLCDKVLKHLDKLCISIKKEQLAIKNFT 184

Msg51 FENK--GEA-FARLNSL-FSDKDICDKILKHLDKLCTIIREEQNAINDFT 177

Msg73 FDKDNKDDA-MDKLHSL-LSKDDLCEKLLKHLQSICLNIKNEHDSIEKYT 177

Msg39 -------------------------------------------------- 45

Msg59 YSAT--GAVSSDFITKRCANVTLTCLYLVKKMDEYCQKIKEEQNNALSVT 191

Msg18 IPKN--DNR-LTSLSFACNNPTETCKKTIHFFDEMCRELEEFLEKTSEFT 173

Msg49 EDVCRTELSKCQDLEESC**NDT**LKDSCKTVRNNCKD------------YIP 222

Msg51 KDVCLIQLSKCHELEENCKNILKDSCKNVKEKCKEK-----------TIL 216

Msg73 QEICDLHLTKCRDLQDSCGEPLKGSCKKLEEKCRKTEHSTEDKT---HVH 224

Msg39 -----------------------NSP---------------------KNH 51

Msg59 KEVCTYHVGNCKYLEKACGESLKKSCTKIKTQCKKLE**N----DS**---KVH 234

Msg18 NYVCRKENLVCKYLGKTCGNKLTTICERLGTHCEKP**NET**-EHKTISTTQS 222

Msg49 MKDAFVVTHTVLIISTTVTTEVAPETSTYVVTETVAEVCCTDTLP-ATTE 271

Msg51 SGDIFTMTHTVLVISTTVTTEIAPETSTCVETMVVTETCCIDRPP-ATME 265

Msg73 PKDLFVKVHTVLTTHTIVAMETVIAPDIYVETLIITKTCCLGRPAPTHTE 274

Msg39 EESKFVNTHTHVADKTEYVTEVLYHTHTEVVF--TIRKCCSGKSWRKSCA 99

Msg59 EDTGFVNTHTQIVDETVYVTETVYYTHTSVIF--TTKKCCSKKTKYKTCA 282

Msg18 EYISFENTHTIILDETIYITETVYSTHTNVVL--KTVDCNSKDTEDCTCT 270

* .** : * * . * *

**<-------------------------------------------------**

Msg49 --PELEPDE----------------PEPDEPKPDEPETPEVPKETEGTEE 303

Msg51 --PEPEPEK------------------PE-----EPTSC--P-------- 280

Msg73 --PEPEPE-----------------PEPEDPEEGEPDEQ--P----GEEE 299

Msg39 KGKVTSKTTSHATVCPTPTRESDPEPEPTDSDSMEPPS------------ 137

Msg59 TKPTTCPA-------------------LSTGSEACPTEA--S-------- 303

Msg18 PNMAICPT-------------------PTFTL--EPTKS--R-------- 289

*

**------------------------PE-rich-------------------**

Msg49 IEETEEVEETEE-VEETEETEP-EPEPEPEPEPEPEPEPEPEPE---PE- 347

Msg51 -----------E-PEPNPEPEP-EPE------PEPEPEPEPEPE---PE- 307

Msg73 PE------EEEPGKEEPEEEEPGKEK------PEEEEKPEEEPE---EEK 334

Msg39 --------STED-LEETDEHGP-TDT------PEPEPEPEPEPE---PE- 167

Msg59 ---------------------------------SEDHDPEPTKTSEDGD- 319

Msg18 ----------------------------------SKSKPTPTT-----S- 299

. .* .

**------------------------------------------------->**

Msg49 PEPEPEP--EPEPEPEPEPEPGPEPEPEPEPEPEPEPEPQPTTVPSEDEP 395

Msg51 PEPEPEP--EPEPEPEPEPEPEPEPEPEPEPEPEPEPEPKPTTMPSEDEP 355

Msg73 PEGEPEE-EKPEEEPEEEEKPEEEPEEEKPEDQEPEPEPVPEEPDQEESG 383

Msg39 PEPEPEP--EPEPEPEPEPEPEPEPEPGPEPTSDPTDPPQPTEEPAETYS 215

Msg59 LEPVETSSEDNDLEPTETSSDESETETSS-EEQEPVEEPEPTEEPTLAEP 368

Msg18 LKPT---------ESKNKPKPSPTSSLET-TESRDKLKPSLTKTPDKNIS 339

: *. . .. *

**----------ST-rich------------------> <------------**

Msg49 TTDDSSCSYTEIVTVTSE--------------PTNTDDSEVEDKGYGLRI 431

Msg51 TTDDSSCSYTEIVTVTSS--------------PTKTSESGIESKSYGLRV 391

Msg73 DSDDKDCTITKTTTVT----------------PEGTEKPGIDDKGYGVRV 417

Msg39 TESDSECSYTETVTINDGYDATV--TVTV---SEESDNNGVSDKGHSVRI 260

Msg59 T-EDEECTITKTITMTNRFGETLTTTVTVSEKPGETNKDQINNKGDSIKK 417

Msg18 IINVKECEITTTMTIKNKFSETITMTVTVSKIPKTTDNAQI-EKSHGVRI 388

. ..* * *:. . :.. : .*. .::

**--GPI-anchor signal--->**

Msg49 GGFHRAGIICLIMGITAGVWIIV 454

Msg51 EKFHEMRVICLIIGITAGIWIIV 414

Msg73 QGFERAGIICLIIGITSGVWIFV 440

Msg39 EGFQRVGVICLIIGIMAGMWIIV 283

Msg59 EAFQKVGIFCLIIGVTTGVWIIV 440

Msg18 E-FQKVLVFLLIIRVIATM---- 406

*.. :: **: : : :
