# Supplementary material for: Mechanisms of Surface Antigenic Variation in the Human Pathogenic Fungus Pneumocystis jirovecii
Source: mBio. 2017 Nov 7;8(6):e01470-17. doi: 10.1128/mBio.01470-17 (PMC5676039; doi:10.1128/mBio.01470-17)
Supplement: FIG S6 [file mbo005173568sf6.pdf]

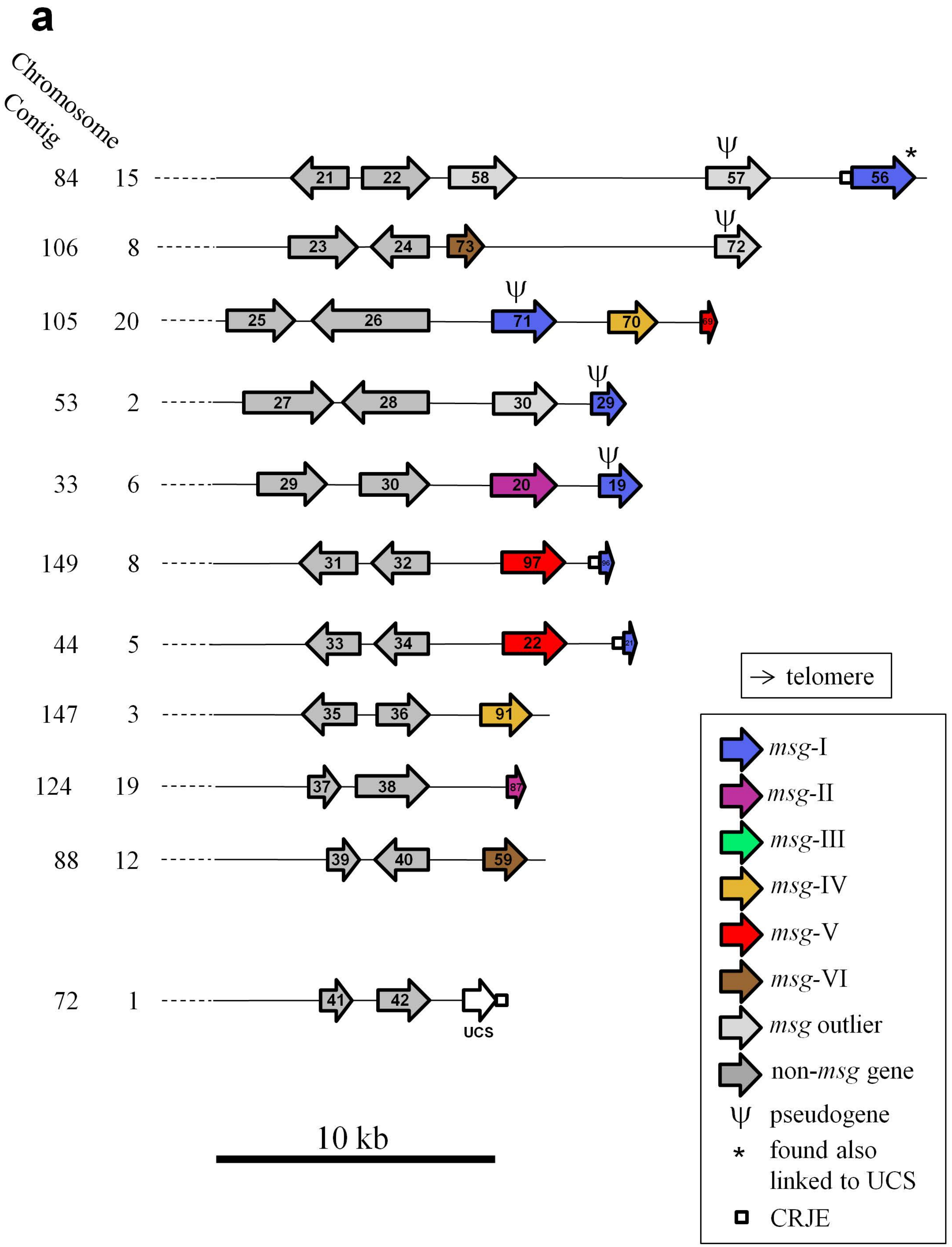

a
Chromosome
Contig
84 15
106 8
105 20
53 2
33 6
149 8
44 5
147 3
124 19
88 12
72 1
UCS
10 kb
→ telomere
msg-I
msg-II
msg-III
msg-IV
msg-V
msg-VI
msg outlier
non-msg gene
ψ pseudogene
* found also linked to UCS
CRJE

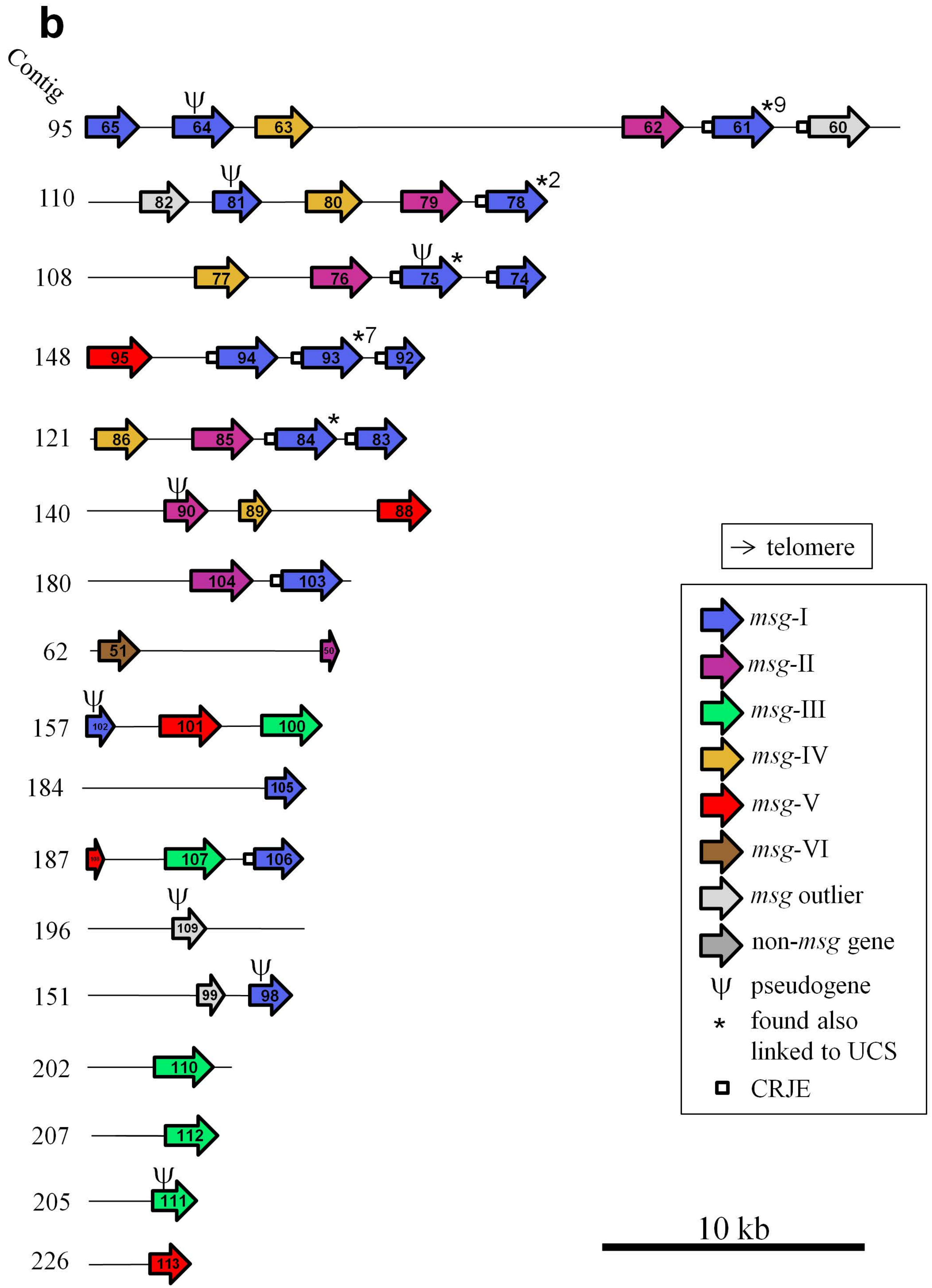

b
Contig
95
65
Ψ
64
63
62
61
*9
60
110
82
Ψ
81
80
79
78
*2
108
77
76
Ψ
75
*
74
148
95
94
93
*7
92
121
86
85
84
*
83
140
Ψ
90
89
88
180
104
103
62
51
50
157
Ψ
102
101
100
184
105
187
108
107
106
196
Ψ
109
151
99
Ψ
98
202
110
207
112
205
Ψ
111
226
113
→ telomere
msg-I
msg-II
msg-III
msg-IV
msg-V
msg-VI
msg outlier
non-msg gene
Ψ pseudogene
* found also linked to UCS
CRJE
10 kb
