# Supplementary material for: Mechanisms of Surface Antigenic Variation in the Human Pathogenic Fungus Pneumocystis jirovecii
Source: mBio. 2017 Nov 7;8(6):e01470-17. doi: 10.1128/mBio.01470-17 (PMC5676039; doi:10.1128/mBio.01470-17)
Supplement: FIG S7 [file mbo005173568sf7.docx]

Fig. S7 (2 pages)

**--------------------------------------------------**

GATTTTCGATGGTTTATAAATGTTTGTATATATGTTTATGTATATGACTT 50

**--------------------------------------------------**

TGTAGGGGAGAAAGCTATTTTTGTATTATGTATAACATGCATTAAAGATG 100

**--------------------------------------------------**

TTATATAGTTGTTTTACGTGATTATTTTGTATTATTTAGGGTTTTTATAG 150

**---------------------contig 72--------------------**

TTGTATTTTATTTGTACTATTCAAGCGTGTGTATTTGAATCTATTTGAGT 200

**--------------------------------------------------**

AAAAGTGTTTTAGAATGGGACACGTATAATATAATGAAACTAAAGTGTTA 250

**----------------------------->**

GCATGGGACAGCATACTGGTATCTTGTATTTTTTATTCTTTTACTATTTT 300

TAGTTGTTTATGTTTTTGGTCAGGGGTAAGTGAAGTGTGTGCAATTTTAT 350

CATTGACGGATCAAAATAAGAAAGGTAAAATTTGATATATTTGCGACATG 400

TCGTAAAAATGTAAATATCTGGATAAATGTTTTAACAAGTGTTTTGACAA 450

ATATTTTGAATGTCTTAAGAGTATGGGTAAAGGTTGTTTTTCAAAATAGA 500

TTTGATTGAGGATGGACTGAAAGCGGGTTGAAGATGTTAAGAATAAGTTT 550

AGGGTAGATTTTATAAATATCTAAAGTGTGTATAAAAAGTGGGTTTAAAG 600

TTTGTCTAAAAAATTTATAAAAAATGCAAAAAAAAGTAACGGTGGGATTT 650

AAAGACAGTTTGTGTTTTTATGTTTTTTTCTGAATTTTATCCTTTGGGTA 700

GGAATTGATTTGTATTTGGGTTATCGTTGTTATATAGTGTATATTTTTTT 750

TCTGTTCTTTTATCGTTATCCGTCCGTTTTCGTTTAATTCTGGGTTGGCT 800

TTAGGTTAGCTTTGGTTGGTTTATTTTTATTCGTATTTGAGGAATATTTC 850

GAGTGACTGGGTTTGCCTCTTGTTTTCGTGTTTTTAGTCTGTTTGCTGTG 900

TTTGGCATGTGTATTTTTTTACCCTCTTGGTCAGCGTAGTATTTTTCTTG 950

**supplementary T**

TGTTTGTTGATTTTTTTGCTG**T**TTTTTGTGTATAAATATATTTTCGTTCG 1000

TTACGGTTTCACGTTGTGTGTCATTTTTTAGATACGGGTGGTATAGCTTT 1050

**<--supplementary**

TATTATAGTTTGTCTAGGTGCTTGTGTTTTACTGTTTT**TTATTCATAGTT** 1100

**duplicated segment**

**segment-->**

**TTTGTTGTCTTATTCATAGTTTTTGTTG**ATCAAATGTTGATCAAGTGGGT 1150

**duplicated segment**

GTTTGTTTTTAGTAGGCTTTTATTTATAATAAATTTAATGGGTTTTTGCT 1200

GGTTTTACCAGCTTTTAAAAGTTATTTGTTTTTATTTTTTTTACTTGTTT 1250

TGTTTTTTACTTGTTTTTGGTGTTTTATTTTCTTGTATTTTTTCTATTTT 1300

ATTTCTACTGGTTTTGGGGGTTTCTTGCCTTTTATCTTTTTCCTCTTCTT 1350

GAGGGGGTCTTTGAGGGGTGCTGGCGGTGCCAGGGATGCCCTGTTTCTTT 1400

TTGCGTGTCCTGTCTTTTTTTCTCCTCTTTTGGGTGTTCCGTGGTTTCTC 1450

AAATTGTTTTTCTCGGCATGAACCCTGTATTCGGTCGTTTTTAGTGTCTT 1500

**C->T T->C**

TTCTG**T**CGAACTTT**C**CCTCTTTCGGCTGTCCTATTCTTTTTTCGTTAGTT 1550

TCATGCGCCCCCGTGCTTTTCCGTCGACATTGTTATCTCTATTCGTCGAT 1600

**14 bps critical for promoter activity**

TT**TTTCTATAACTGGT**CAATATTTTTCCCCTTGCCCATCTCCTGTTTTAG 1650

**transcription start**

**TATA box Cap signal**

TGTCTTAAGAAA**TATATTT**TTTCTTCATCGCCCGTCTTTTTCAATA**T**TT**C** 1700

**A**TTTGTGCAATC**ATG**AGAGTTGCACTTTTTGCACTTTCAGCGCAGGTTGG 1750

M R V A L F A L S A Q V G

**<------------------------------------**

**A->G**

TTGCGCTTTGGCGGCCCTTTT**G**AACGACGCATATAGGCCAGATTTTGAAG 1800

C A L A A L L N D A Y R P D F E

**-------------------signal peptide-----------------**

**<----------------------------------------**

AGGTGCGAG**GTATGT**TGTCTCTTTTTGCGCGCTTTTTTCTAGTTTTCTCT 1850

E V R

**------>**

**--------------------------------------------------**

GTTTTGCTTGCTCACGGCTTTCTGTGGATTGAGCTATTTCTTGTATCTAT 1900

**--------------------------------------------------**

GCGCTATTTTCTTGTATGGCCTGCAT**ACAGGCGCAT*ACAGGCGCAT***GCAA 1950

**Tandem repeat type : 1 *1***

**--------------------------------------------------**

GTGCTTATTTTTGCGCGGAATGTCAGCTTGCATGCTTTTATTTCTCGGGG 2000

**-----------------------intron---------------------**

AGTTTTCGAGAAGTTTCCGAGAAGTATTTTCTATTTAAGGCCGCTTAGGG 2050

**--------------------------------------------------**

CCGCTTTTCTAGCGCTTTGTTGCTTGATTTGCATGCTTTGAGTGGTTTTG 2100

**--------------------------------------------------**

TCTGGGCAGTGTTTGAACAATGTCTGGGCATTCTGTGGTTGCTCGTTTTT 2150

**--------------------------------------------------**

**G->T C->T**

TGCAGCCGCATAGGGCAGGGCTT**T**C**T**CAGCCCTGCCCTGCCTTGGCAGGG 2200

**--------------------------------------------------**

CTAGGGCTGCTGCCTGCAGGGGTGGCCTGAGTAGGCAGTGGGAATGCGCG 2250

**----------------------------->**

TTTTTCTTTTAGCTAGGGCTAACGTGG**CAG**ATCATGATGCTCTTTCGGCA 2300

D H D A L S A

TCTCTCCACAACGGGAAACAGCTTGGGGCAGGACATTTGGGGGAGCCTCG 2350

S L H N G K Q L G A G H L G E P R

GCGTTTGTATCGCCGCTCAGATGACGAATATGATGAACTGGATGCAAGGA 2400

R L Y R R S D D E Y D E L D A R

TGGACCATGAAGACGACTTGGAGCTTAGGATGCATTTGGATACGAGCTTT 2450

M D H E D D L E L R M H L D T S F

**<-**

GACAAGGATGTTGCTTTTGATGCCGCAGGACTTGAGTCGGGCCACAGC**TT** 2500

D K D V A F D A A G L E S G H S L

**-------------CRJE------------->**

**GGCGCGGGCGG**T**GGCGCGGGCGGTT**AAGCGC 2541

A R A V A R A V K R

**imperfect inverted repeat** Recognition site Kexin endonuclease
